# Asymmetric dearomative cyclopropanation of naphthalenes to construct polycyclic compounds†

**DOI:** 10.1039/d2sc04509e

**Published:** 2022-10-12

**Authors:** Fujun Guan, Rong Zhou, Xiaoyu Ren, Zhen Guo, Chengming Wang, Cong-Ying Zhou

**Affiliations:** College of Chemistry and Materials Science, Guangdong Provincial Key Laboratory of Functional Supramolecular Coordination Materials and Applications, Jinan University Guangzhou 510632 People's Republic of China cmwang2019@jnu.edu.cn zhoucy2018@jnu.edu.cn; College of Materials Science & Engineering, Key Laboratory of Interface Science and Engineering in Advanced Materials, Ministry of Education, Taiyuan University of Technology Shanxi 030024 People's Republic of China guozhen@tyut.edu.cn

## Abstract

Catalytic asymmetric dearomatization (CADA) reactions is an important synthetic method for constructing enantioenriched complex cyclic systems from simple aromatic feedstocks. However, the CADA reactions of nonactivated arenes, such as naphthalenes and benzenes, have been far less explored than those of electronically activated arenes, such as phenols, naphthols and indoles. Herein, we disclose an asymmetric dearomative cyclopropanation of naphthalenes for the rapid construction of polycyclic compounds. With chiral dirhodium carboxylate as a catalyst, the dearomative cyclopropanation proceeded smoothly under mild conditions and afforded benzonorcaradiene-containing tetracycles in good yield and high enantioselectivity (up to 99% ee). Three stereogenic centers, including two all-carbon quaternary centers, were created in the dearomatization reaction. Moreover, a variety of functional groups are well-tolerated in the reaction. The products could be readily converted into other complex polycycles while maintaining the high ee value.

## Abstract 2

An enantioselective dearomative cyclopropanation of naphthalenes has been developed, which constructed a range of tetracyclic compounds bearing multichiral centers in high enantioselectivity.

## Graphical abstract

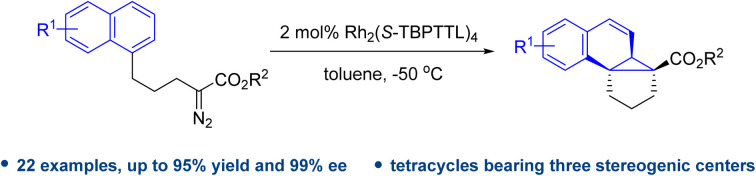

## Introduction

Catalytic asymmetric dearomatization (CADA) reactions have emerged as a unique and powerful method for constructing enantioenriched functionalized cyclic systems.^1^ This synthetic strategy enables the direct conversion of simple, planar aromatic feedstocks into complex three-dimensional molecules that often exhibit biological activity and physicochemical properties superior to those of flat molecules.^2^ Significant progress has been achieved in this field during recent decade, with a focus on electronically activated aromatic compounds such as phenols, naphthols and indoles.^1^ In contrast, more readily available nonactivated arenes, such as naphthalenes and benzenes, have rarely been applied in CADA reactions owing to their inherently low reactivity.^3^ The resulting high energy barrier often leads to the need for harsh conditions for this type of transformation, which makes stereocontrol very challenging.

**Scheme 1:**
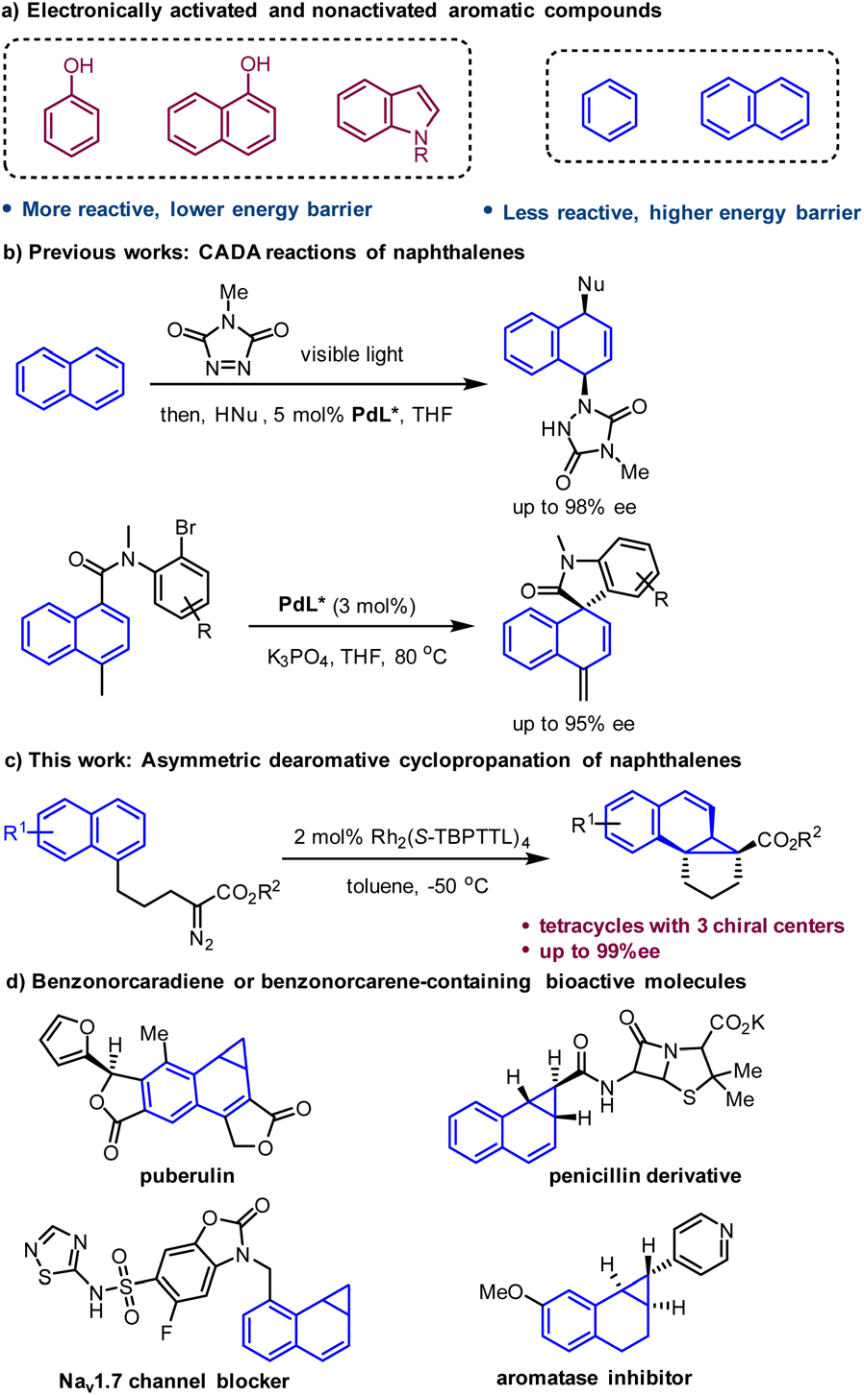
CADA reactions of naphthalenes.

Polycyclic structures are ubiquitous in natural products and pharmaceuticals. The rigidity and well-defined 3D spatial configuration of polycyclic architectures have a significant impact on their biological activities.^4^ As strenuous synthetic efforts are often necessary for the synthesis of polycyclic compounds, it is highly desirable to develop simple and efficient synthetic methods for the rapid construction of polycyclic architectures from readily available feedstocks. Dearomatization of naphthalenes has demonstrated to be an effective method for the straightforward construction of polycyclic molecules.^3,5^ However, the development of asymmetric versions of this type of transformation remains a formidable challenge because of the lack of efficient chiral catalytic systems or the requirements for harsh reaction conditions. To date, sporadic dearomatization reactions of naphthalenes with high enantioselectivity have been developed.^6–8^ Recently, an enantioselective dearomative difunctionalization of naphthalenes was achieved by Sarlah's group, which involved visible-light-mediated [4 + 2] cycloaddition of naphthalenes with an arenophile and subsequent asymmetric ring-opening of the resulting cycloadducts.^6^ In 2022, Jia, Zhang and You developed a Pd-catalyzed intramolecular dearomative Mizoroki–Heck reaction of naphthalenes to construct spirooxindole and spiroisoindolin-1-one with high enantioselectivity (Scheme 1b).^7^

The kinetic barrier of chemical reactions can be significantly reduced by using a highly reactive reagent or species, which offers a solution to the dearomatization of nonactivated arenes under mild conditions. Metal-carbenes, which are highly reactive species, are capable of reacting with nonactivated arenes at low temperature to afford the Buchner reaction product cycloheptatriene.^9^ In contrast to the well-studied Buchner reaction, arene cyclopropanation with a metal-carbene has been far less explored due to the facile electrocyclic ring opening of norcaradiene (the arene cyclopropanation product) to form the more stable tautomer cycloheptatriene.^9,10^ Herein, we reported a highly enantioselective intramolecular dearomative cyclopropanation of naphthalenes with a metal-carbene intermediate to construct benzonorcaradiene-containing tetracyclic compounds with three stereogenic centers, two of which are all-carbon quaternary centers (Scheme 1c). Benzonorcaradiene and benzonorcarene structures have been found in many biologically active molecules (Scheme 1d).^11^

## Results and discussion

We commenced our study with naphthalene-tethered diazoester 1a as the model substrate. Initially, we examined a range of chiral catalysts for the asymmetric dearomative cyclopropanation of naphthalenes (Table 1, entries 1–10). When dirhodium carboxylates were used as catalysts, the [2 + 1] cycloaddition proceeded smoothly at low temperature (+78 °C) and afforded polycyclic product 2a in good yield; no Buchner reaction product 3 was observed. The conversion of benzonorcaradiene 2a to cycloheptatriene 3 was kinetically and/or thermodynamically disfavored by concomitant dearomatization. Davies's Rh_2_[*S*-DOSP]_4_,^12^ Rh_2_[*R*-BTPCP]_4_ ^13^ and Rh_2_[*S*-PTAD]_4_,^14^ which exhibited high enantioselectivity for carbene C–H bond insertion and alkene cyclopropanation, afforded 2a in high yield but with modest enantioselectivity. The *tert*-leucine-derived catalyst Rh_2_[*S*-PTTL]_4_ ^15^ that was developed by Hashimoto exhibited good enantiocontrol for the dearomative [2 + 1] cycloaddition, delivering 2a in 66% ee.

**Table 1:** Screening of catalysts and reaction conditions^a^^,^^b^^,^^c^

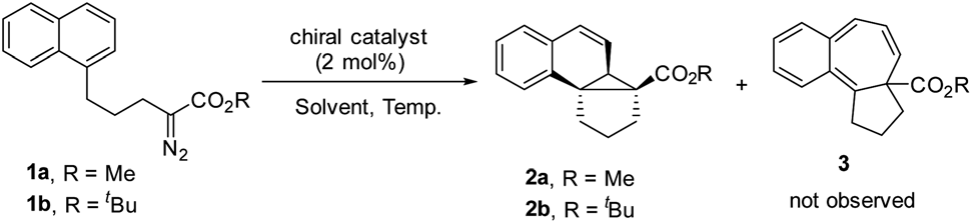						
Entry	R	Catalyst	Solvent	*T* [°C]	%Yield	ee%
1	Me	Cat. 1	DCM	−78	88	39
2	Me	Cat. 2	DCM	−78	87	42
3	Me	Cat. 3	DCM	−78	89	45
4	Me	Cat. 4	DCM	−78	94	66
5	Me	Cat. 5	DCM	−78	94	45
6	Me	Cat. 6	DCM	−78	90	67
7	Me	Cat. 7	DCM	−78	90	78
8	Me	Cat. 8	DCM	−78	90	21
9	Me	Cat. 9	DCM	0	0	
10	Me	Cat. 10	DCM	0	0	
11	^*t*^Bu	Cat. 7	DCM	−78	46	90
12	^*t*^Bu	Cat. 7	DCM	−20	56	95
13	^*t*^Bu	Cat. 7	DCM	−50	64	97
14	^*t*^Bu	Cat. 7	Hexane	−50	46	93
15	^*t*^Bu	Cat. 7	TBME	−50	53	93
**16**	^***t***^ **Bu**	**Cat. 7**	**Toluene**	**−50**	**80**	**99**
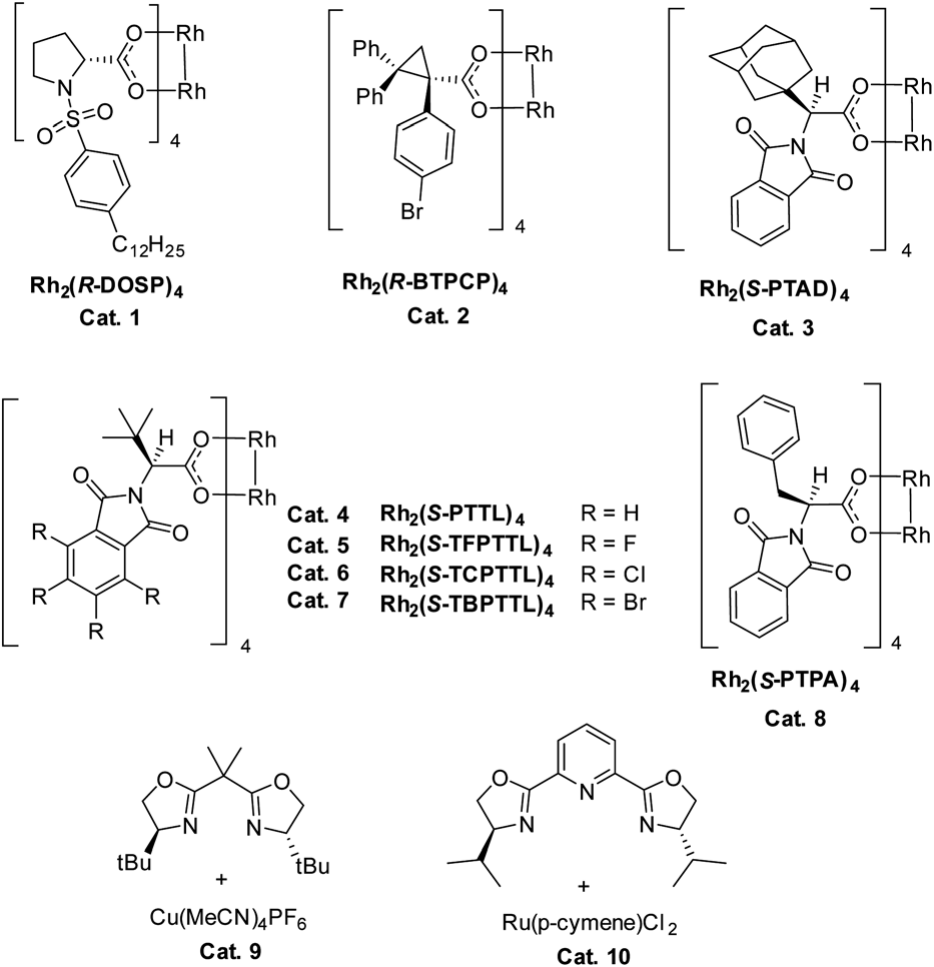						

a Reactions were conducted with 1a or 1b (0.1 mmol) and the catalyst (2 mol%) in 2 ml solvent under Ar.

b Isolated yields.

c Determined by HPLC analysis.

Halogen substituents on the ligand have obvious influence on the performance of this kind of catalyst. As shown in entries 5–7, the fluorinated, chlorinated and brominated analogs of Rh_2_[*S*-PTTL]_4_ afforded 2a in 45% ee, 67% ee and 78% ee, respectively. Compared to Rh_2_[*S*-PTTL]_4_, the phenylalanine-derived catalyst Rh_2_[*S*-PTPA]_4_ was less effective in achieving enantiocontrol for the reaction, affording 2a in 21% ee. Other metal catalysts, such as Cu(i)/Box^16^ and Ru(ii)/Pybox,^17^ which have demonstrated to be effective for enantioselective alkene cyclopropanation with diazoesters, failed to catalyze the dearomative cyclopropanation. The major side product was found to be an α,β-unsaturated ester (60–70% yields) generated *via* β-hydrogen migration.^18^ With Rh_2_[*S*-TBPTTL]_4_ as a catalyst and the use of sterically bulky *tert*-butyl ester, the enantioselectivity was significantly improved to 90% (entry 11), albeit with a lower yield than that achieved with methyl ester. Increasing the temperature from −78 °C to −50 °C improved both the yield and the enantioselectivity of the product. The effect of the solvent was also examined, and toluene proved to be the optimal solvent, affording 2b in 80% yield and with 99% ee (entry 16). No reaction between 1b and toluene was observed. The stereochemistry of 2b was assigned based on single-crystal X-ray diffraction analysis of its analogs 6 and 7.

After determining the optimized conditions, the substrate scope was examined. As depicted in Table 2, a variety of naphthalene-tethered diazoesters underwent intramolecular dearomative cyclopropanation with excellent enantioselectivity. Compared to sterically bulky *tert*-butyl ester and neopentyl ester, methyl, ethyl, propyl and benzyl esters led to a slightly lower enantioselectivity but higher yields (2a–2f). Various substituents at positions 4, 5, 6 and 8 of the naphthalene ring were well tolerated in the reaction, regardless of whether they were electron-donating or electron-withdrawing groups, affording the desired products in 84–99% ee. 2-Substituted substrate (2w) was not compatible with the reaction and failed to generate the corresponding cycloadduct, presumably due to steric hindrance, and the major product in this reaction was an α,β-unsaturated ester generated *via* β-hydrogen migration. Substrates bearing electron-donating groups (6-alkoxy) exhibited good reactivity and excellent enantioselectivity, affording the desired products in good yield and high enantioselectivity (95–99% ee, 2i–2k). An electron-withdrawing group (6-CN, 2t) made the naphthalene ring less reactive for dearomative cyclopropanation, leading to the desired products in lower yield than that achieved by its electron-donating counterparts, which is consistent with the electrophilicity of rhodium-carbene.^9*a*,*b*^ Notably, alkene and alkyne moieties, which are reactive functional groups in carbene transfer reactions, remained unaffected during rhodium catalysis (97–99% ee, 2j, 2k, 2n, and 2o). Various aryl substituents, including phenyl, naphthyl and thienyl groups, were well tolerated in the dearomative [2 + 1] cycloaddition, delivering the desired products with high enantioselectivity (92–99% ee, 2p–2s). Moreover, halide, amino, OTf and ester moieties were compatible with rhodium catalysis and afforded products with high enantioselectivity (85–99% ee, 2g, 2h, 2l, 2m, and 2p). These synthetically useful functional groups are expected to enable many further transformations, for instance, various cross-coupling reactions of aryl (pseudo)halides.^19^ When an acenaphthene-tethered diazoester was subjected to the rhodium catalysis, a fused pentacycles was obtained in good yield and excellent enantioselectivity (93% ee and 2v).

**Table 2:** Substrate scope^a^

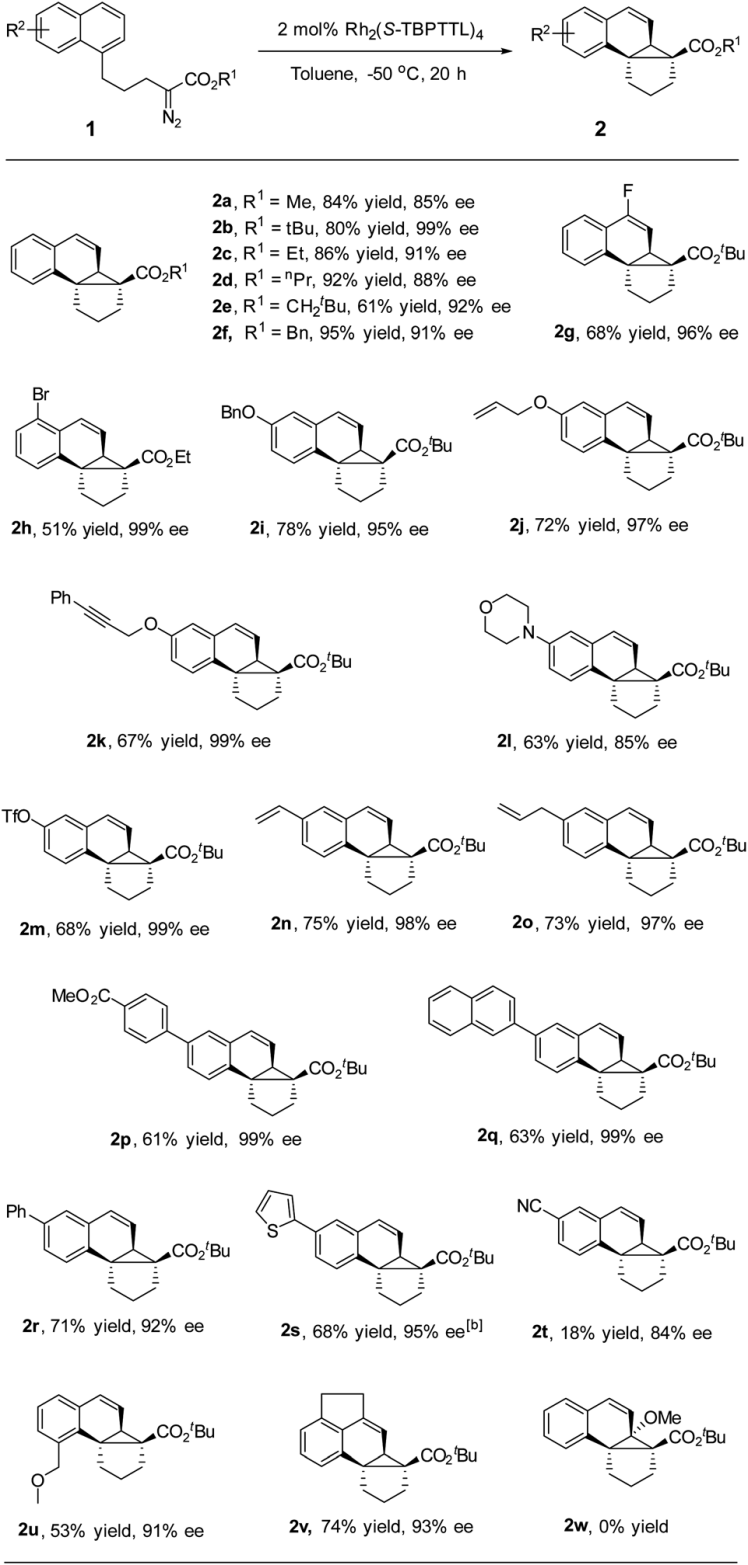

a Reactions were conducted with 1 (0.1 mmol) and catalyst (2 mol%) in 2 mL toluene at −50 °C under Ar.

b −40 °C obtained in good yield and excellent enantioselectivity (93% ee)

**Scheme 2:**
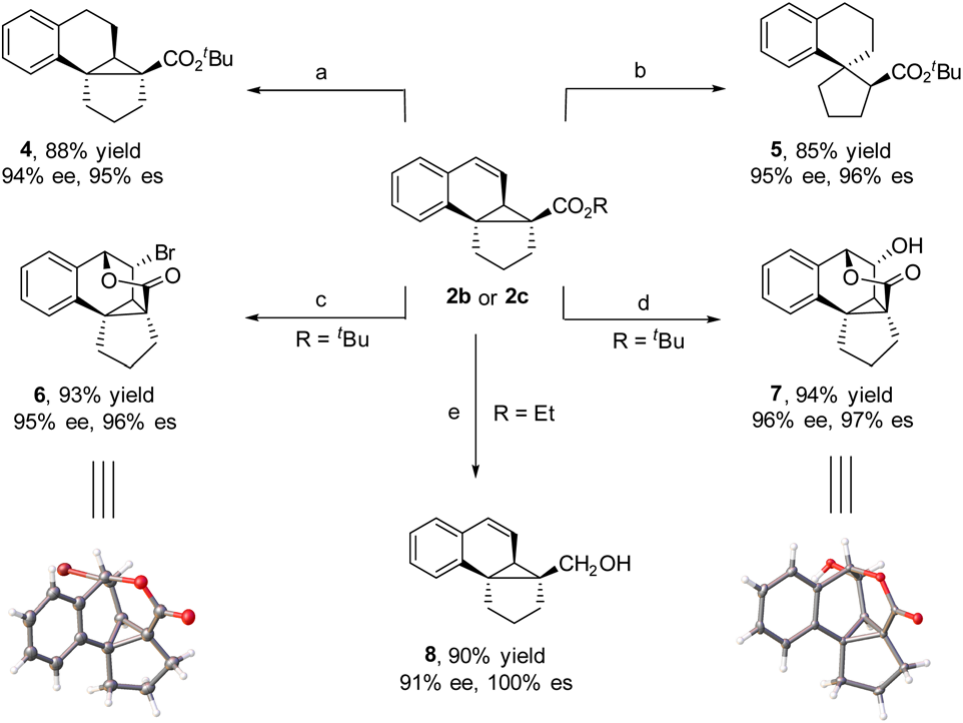
Synthetic transformation. Reaction conditions: (a) Pd(OH)_2_, EA, rt, and 4 h. (b) Pd/C (10%), EA, rt, and 6 h. (c) NBS (1.5 equiv.), CH_3_OH, 50 °C, and 2 h. (d) CH_3_O_3_Re (1.5 mol%), pyrazole (12 mol%), H_2_O_2_, MnO_2_ (8 mol%), DCM, and 0 °C–rt. (e) LiAlH_4_ (1.5 equiv.), THF, rt, and 3 h.

The obtained tetracyclic products could be readily converted to other complex polycycles. For instance, the hydrogenation of 2b with H_2_, which was catalyzed by Pd(OH)_2_ at room temperature, afforded polycycle 4 in 88% yield and 94% ee. Interestingly, when hydrogenation was performed with Pd/C as the catalyst, 2b underwent cyclopropane ring opening to generate tricyclic spiro tetrahydronaphthene 5 in 85% yield and 95% ee, where compound 4 is an intermediate in the reaction. When 2b was treated with NBS at room temperature, bridged pentacycle 6 was obtained as a single stereoisomer in 93% yield and 95% ee. The bridged pentacyclic compound contained five consecutive stereogenic centers, two of which were all-carbon quaternary centers. The cyclization reaction likely proceeded *via* NBS-mediated formation of a bromonium ion with the C=C bond of 2b and subsequent nucleophilic addition of the ester group to the bromonium ion with concomitant loss of the *tert*-butyl moiety. Similarly, when 2b was subjected to H_2_O_2_ and a catalytic amount of CH_3_O_3_Re and MnO_2_ (a protocol for the epoxidation of C=C bonds),^5*i*^ bridged pentacyclic compound 7 was obtained in 94% yield and 96% ee. The structures of 6 and 7 were determined by X-ray crystallographic analysis. Although the α-carbon is a quaternary center with high steric bulk, the ester moiety of polycyclic product 2c was readily reduced by LiAlH_4_ at room temperature to a hydroxy group in high yield and high enantioselectivity (8) (Scheme 2).

## Conclusions

In summary, we developed an enantioselective dearomative cyclopropanation of naphthalenes to construct benzonorcaradiene-containing tetracyclic compounds. Chiral dirhodium catalyst Rh_2_[*S*-TBPTTL]_4_ efficiently promoted the intramolecular dearomatization of naphthalenes in good yield and excellent enantioselectivity. Three stereogenic centers, including two all-carbon quaternary centers, were created in the dearomatization reaction. Moreover, a variety of functional groups were compatible with this rhodium catalysis. The obtained products could be readily converted to other complex polycycles in high yield while retaining the high ee value.

## Data availability

We have experimental or computational data associated with this article, and these data have been included in Electronic supplementary information (ESI) which are available free of charge at https://doi.org/10.1039/d2sc04509e.

## Author contributions

F. G., R. Z. and X. R. conceived and performed the experiments. C.-Y. Z., C. W. and Z. G. conceived and directed the project and wrote the paper. All the authors discussed the results and commented on the manuscript.

## Conflicts of interest

There are no conflicts to declare.

## Supplementary Material

SC-013-D2SC04509E-s001

SC-013-D2SC04509E-s002

## References

[cit1] Zhuo C.-X., Zhang W., You S.-L. (2012). Angew. Chem., Int. Ed..

[cit2] Lovering F., Bikker J., Humblet C. (2009). J. Med. Chem..

[cit3] Wertjes W. C., Southgate E. H., Sarlah D. (2018). Chem. Soc. Rev..

[cit4] (b) Nettekoven M. , Plancher J.-M., Richter H., Roche O., and Taylor S., PCT Int. Appl., WO *US.pat*. 2007/0135416 A1, 2007

[cit5] Liu L., Wang Z., Zhao F., Xi Z. (2007). J. Org. Chem..

[cit6] Okumura M., Shved A. S., Sarlah D. (2017). J. Am. Chem. Soc..

[cit7] Han X.-Q., Wang L., Yang P., Liu J.-Y., Xu W.-Y., Zheng C., Liang R.-X., You S.-L., Zhang J., Jia Y.-X. (2022). ACS Catal..

[cit8] Kuwano R., Morioka R., Kashiwabara M., Kameyama N. (2012). Angew. Chem., Int. Ed..

[cit9] (b) Doyle M. P. , McKervey M. A. and Ye T., Modern Catalytic Methods for Organic Synthesis with Diazo Compounds, Wiley, New York, 1998

[cit10] Reisman S. E., Nani R. R., Levin S. (2011). Synlett.

[cit11] (c) Layton M. E. , Pero J. E., Fiji H., Kelly III M. J., De Leon P., Rossi M. A., Gilbert K. F., Roecker A. J., Zhao Z., Mercer S. P., et al, International Patent No. WO2013/063459, 2013

[cit12] Davies H. M. L., Bruzinski P. R., Lake D. H., Kong N., Fall M. J. (1996). J. Am. Chem. Soc..

[cit13] Qin C., Boyarskikh V., Hansen J. H., Hardcastle K. I., Musaev D. G., Davies H. M. L. (2011). J. Am. Chem. Soc..

[cit14] Reddy R., Lee G., Davies H. M. L. (2006). Org. Lett..

[cit15] Yamawaki M., Tsutsui H., Hashimoto S. (2002). Tetrahedron Lett..

[cit16] Evans D. A., Woerpel K. A., Hinman M. M., Faul M. M. (1991). J. Am. Chem. Soc..

[cit17] Nishiyama H., Itoh Y., Matsumoto H., Park S.-B., Itoh K. (1994). J. Am. Chem. Soc..

[cit18] DeAngelis A., Panish R., Fox J. M. (2016). Acc. Chem. Res..

[cit19] Seechurn C. C. C. J., Kitching M. O., Colacot T. J., Snieckus V. (2012). *Angew. Chem., Int. Ed.*.

