# Supplementary material for: Asymmetric dearomative cyclopropanation of naphthalenes to construct polycyclic compounds
Source: Chem Sci. 2022 Oct 12;13(44):13015–9. doi: 10.1039/d2sc04509e (PMC9669881; doi:10.1039/d2sc04509e)
Supplement: SC-013-D2SC04509E-s001 [file SC-013-D2SC04509E-s001.pdf]

Electronic Supplementary Material (ESI) for Chemical Science.
This journal is © The Royal Society of Chemistry 2022

Asymmetric Dearomative Synthesis of Polycyclic Compounds via Intramolecular Cyclopropanation of Naphthalenes

Fujun Guan, [a]† Rong Zhou, [a]† Xiaoyu Ren,[b] Zhen Guo,[b]* Chengming Wang,[a]* and Cong-Ying Zhou [a]*

[a] College of Chemistry and Materials Science
Guangdong Provincial Key Laboratory of Functional Supramolecular Coordination Materials and Applications
Jinan University
Guangzhou 510632 (China)
E-mail: cmwang2019@jnu.edu.cn, zhoucy2018@jnu.edu.cn

[b] College of Materials Science & Engineering
Key Laboratory of Interface Science and Engineering in Advanced Materials, Ministry of Education
Taiyuan University of Technology
Shanxi 030024 (China)
E-mail: guozhen@tyut.edu.cn

Contents

1. Experimental Section 1

1.1 General Information 2

1.2 General procedure for the preparation of substrates **1** 2

1.3 General procedure for the intramolecular dearomative cyclopropanation of naphthalenes 3

(1) General procedure for the reaction condition screening 3

(2) General procedure for Rh_2(*S*-TBPTTL)$_2$-catalyzed intramolecular dearomative cyclopropanation of naphthalenes 3

1.4 Procedure for the synthesis of compound **4**, **5**, **6**, **7** and **8** 5

2. Characterization data of compounds 6

2.1 Characterization data of substrates **1** 6

2.2 Characterization data of products **2** 11

3. X-Ray crystallographic data of **6** and **7** 20

4. References 21

5. NMR Spectra of Compounds 22

NMR spectrum for substrates **1** 22

NMR spectrum for products **2** 44

6. HPLC spectra of products **2** 71

1. Experimental Section

1.1 General Information

All commercial reagents were used as provided without further purification. The substrates **1** were prepared according to the literature.[1-2] The reactions were monitored by thin layer chromatography (TLC) on silica gel GF254 coated 0.2 mm plates (Branch of Qingdao Haiyang Chemical plant). The product spots were visualized with UV and phosphomolybdic acid (PMA). Flash column chromatography were performed using silica gel (200-300 mesh, Branch of Qingdao Haiyang Chemical plant) and a gradient solvent system (EtOAc/n-hexane as eluent). ^{1}H and ^{13}C NMR spectra were recorded on either a Bruker Avance 300 spectrometer. Chemical shifts (δ) were measured with tetramethylsilane (TMS) as internal reference. High Resolution Mass Spectrometry (HR-MS) data were obtained on AB SCIEX TripleTOF 5600+ mass spectrometer. Enantiomeric excess (ee) was determined using Agilent 1260 Infinity II high-performance liquid chromatography (HPLC) with a UV detector (at appropriate wavelength).

1.2 General procedure for the preparation of substrates 1 [1-2]

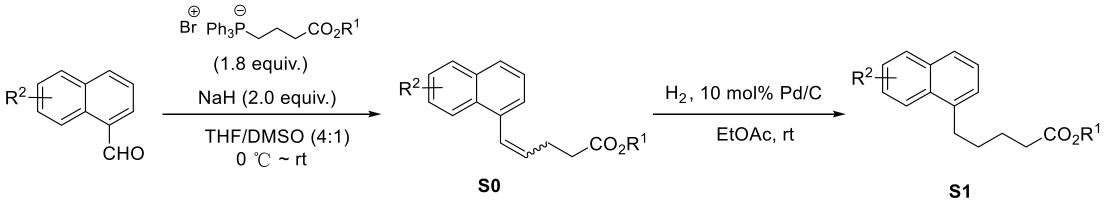

To a round-bottomed flask containing a Wittig reagent (1.8 equiv.) and sodium hydride (60%, 2.0 equiv.) was added THF/DMSO (4:1) at 0 °C. After stirred at room temperature for 30 mins, 1-naphthaldehydes (1.0 equiv.) was added to the reaction mixture at 0 °C and the reaction mixture was stirred at room temperature until the complete consumption of 1-naphthaldehydes as monitored by TLC analysis. Then the reaction was quenched with H_2O, extracted with EtOAc, washed with brine, dried with Na_2SO_4, filtered and concentrated. The residue was purified by silica gel column chromatography to afford compound **S0**.

A round-bottom flask equipped with a magnetic stir bar was charged with **S0**, Pd/C (10 mg/1 mmol substrate) and EtOAc (5 mL/mmol). The reaction was stirred for 5 hours under balloon-pressure of hydrogen. Then the reaction mixture was filtered through a celite pad with EtOAc, dried with Na_2SO_4, and concentrated under vacuo. The product **S1** was used without chromatographic purification.

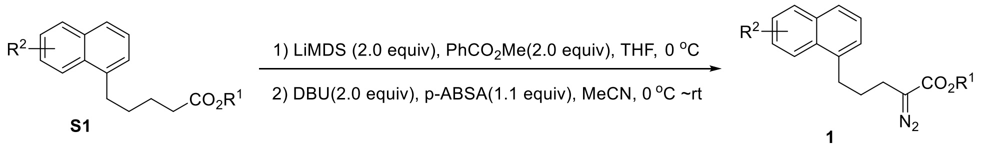

A 50-mL schlenk tube containing a magnetic stirring bar was dried with a heat-gun in vacuo and flushed with argon three times after cooling to room temperature. Ester **S1** (1.0 equiv.), methyl benzoate (2.0 equiv.) and anhydrous THF were added and the resulting solution was

stirred at 0°C for 5 minutes. Then to the solution was slowly added LiMDS (1.0 M in THF, 2.0 equiv) The solution was stirred for 2 hours and then poured into sat. NH_4Cl aq. The resulting mixture was extracted two times with EtOAc, dried over Na_2SO_4, filtrated, and concentrated in vacuo to afford a yellow oil. The yellow oil was immediately was dissolved in MeCN (0.20 M). To the resulting solution were added p-acetamidobenzenesulfonyl azide (p-ABSA, 1.1 equiv.) and 1,8-diazabicyclo[5.4.0]undec-7-ene (DBU, 2.0 equiv.) at 0 °C. After stirring the mixture for 5 hours at room temperature, the reaction was diluted with EtOAc. The organics were washed with water and brine, dried over Na_2SO_4, filtrated and concentrated in vacuo. The residue was purified by flash column chromatography on silica gel to give the corresponding product.

1.3 General procedure for the intramolecular dearomative cyclopropanation of naphthalenes

(1) General procedure for reaction condition screening

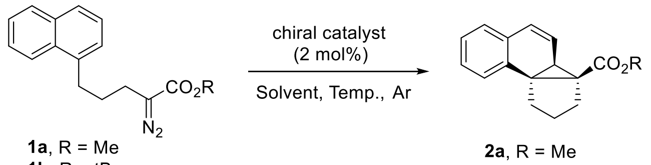

1a, R = Me
1b, R = tBu

2a, R = Me
2b, R = tBu

Catalyst (2 mol%) was added to a flame-dried 10 mL schlenk tube with a magnetic stir bar. The tube was sealed, evacuated and flushed with argon three times. Then anhydrous solvent (0.5 mL, noted in Table S1) was added and the mixture was stirred under the indicated temperature for 15 minutes. Then substrate **1a** or **1b** (0.1 mmol, 1.0 equiv.) was dissolved in the same anhydrous solvent (1.5 mL, noted in Table S1). Then the solution was injected into the tube for 30 minutes by using a syringe pump. The reaction mixture was then stirred for the indicated temperature and time in Table S1. Upon completion, the reaction was warmed to ambient temperature. The mixture was concentrated by vacuum and the residue was purified by column chromatography on silica gel to give the corresponding product.

(2) General procedure for Rh_2(*S*-TBPTTL)$_2$-catalyzed intramolecular dearomative cyclopropanation of naphthalenes

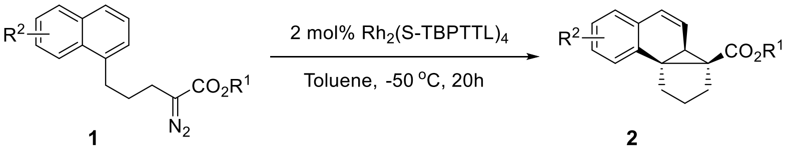

1

2

Rh_2(*S*-TBPTTL)$_4$ (2 mol%) was added to a flame-dried 10 mL Schlenk tube with a magnetic stir bar. The tube was sealed, evacuated and flushed with argon three times. Then 0.5 mL anhydrous toluene was added and the mixture was stirred under -50°C for 15 minutes. Then substrate **1** (0.1 mmol, 1.0 equiv.) was dissolved in 1.5 mL toluene. Then the solution was injected into the schlenk tube for 30 minutes by using a syringe pump. The reaction mixture was then stirred under -50°C for 20 hours. Upon completion, the reaction was warmed to

ambient temperature. The mixture was concentrated by vacuum and the residue was purified by column chromatography on silica gel to give the corresponding product.

Table S1. Screening of catalyst and reaction conditions

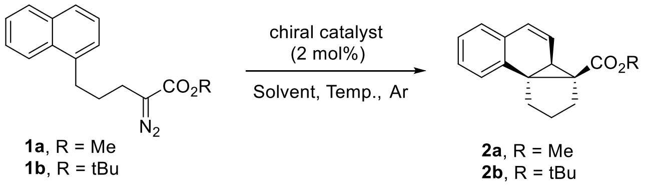

Entry	R	Catalyst	solvent	*T*[°C]	%Yield	ee%
1	Me	Cat. 1	DCM	-78	88	39
2	Me	Cat. 2	DCM	-78	87	42
3	Me	Cat. 3	DCM	-78	89	45
4	Me	Cat. 4	DCM	-78	94	66
5	Me	Cat. 5	DCM	-78	94	45
6	Me	Cat. 6	DCM	- 78	90	67
7	Me	Cat. 7	DCM	-78	90	78
8	Me	Cat. 8	DCM	-78	90	21
9	Me	Cat. 9	DCM	0	0	
10	Me	Cat. 10	DCM	0	0	
11	tBu	Cat. 7	DCM	-78	46	90
12	tBu	Cat. 7	DCM	-20	56	95
13	tBu	Cat. 7	DCM	-50	64	97
14	tBu	Cat. 7	Hexane	- 50	46	93
15	tBu	Cat. 7	TBME	-50	53	93
**16**	**tBu**	**Cat. 7**	**Toluene**	**-50**	**80**	**99**

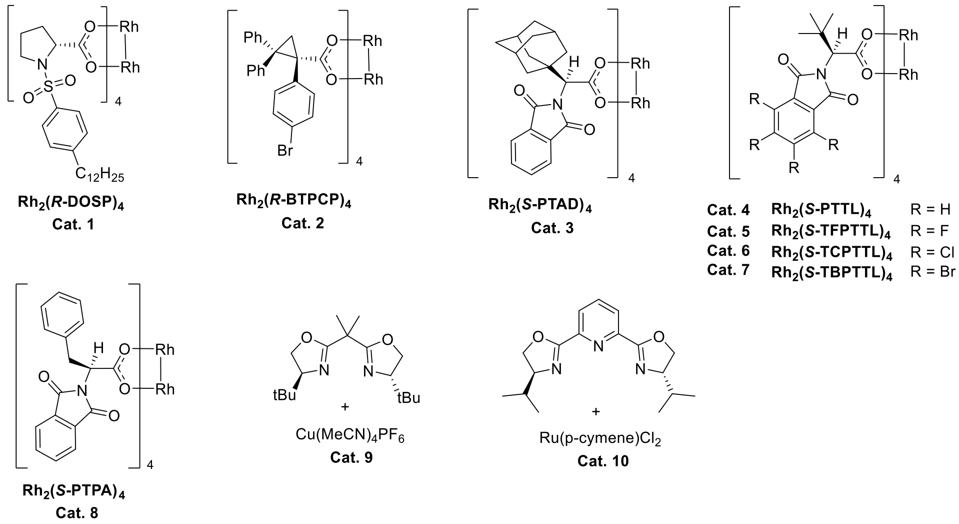

[a] Reactions were conducted with **1a** or **1b** (0.1 mmol) and catalyst (2 mol%) in 2 mL solvent under Ar. [b] Isolated yields. [c] Determined by HPLC analysis.

1.4 Procedure for the synthesis of compound 4, 5, 6, 7 and 8

Compound 4

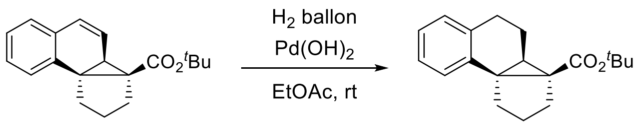

2b **4**

A dry 10 mL round-bottom flask equipped with a magnetic stir bar was charged with **2b** (30 mg, 0.11 mmol), $Pd(OH)_2$ (2.3 mg, 0.017 mmol) and EtOAc (1 mL). The reaction was stirred for 6 h under balloon-pressure of hydrogen. Then the reaction was filtered through a celite pad with EtOAc, dried with Na_2SO_4, concentrated under vacuo. The the residue was purified by column chromatography on silica gel to give the corresponding product **4** as colorless liquid (26.2 mg, 88% yield, 94% ee).

Compound 5

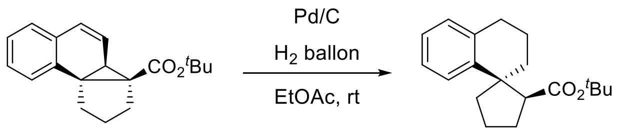

2b **5**

A dry 10 mL round-bottom flask equipped with a magnetic stir bar was charged with **2b** (30 mg, 0.11 mmol), Pd/C (5.5 mg) and EtOAc (1 mL). The reaction was stirred for 6 h under balloon-pressure of hydrogen. Then the reaction was filtered through a celite pad with EtOAc, dried with Na_2SO_4, concentrated under vacuo. The the residue was purified by column chromatography on silica gel to give the corresponding product **5** as colorless liquid (25.8 mg, 85%, 95% ee)

Compound 6

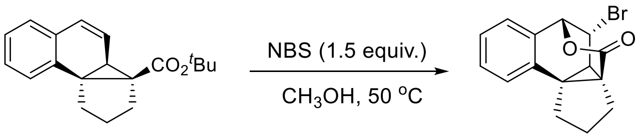

2b **6**

To a stirred solution of **2b** (28.2 mg, 0.1 mmol, 1.0 equiv.) in CH_3OH (3.0 mL) was added NBS (26.7 mg, 0.15 mmol, 1.5 equiv.) at room temperature. After stirring for 2 h at 50 °C, the reaction was extracted with ethyl acetate (2×20 mL) and then washed with H_2O and brine, dried over Na_2SO_4 and concentrated in vacuum. The crude product was purified by flash column chromatography on silica gel to yield product **6** as white solid (28.4 mg, 93%, 95% ee).

Compound 7

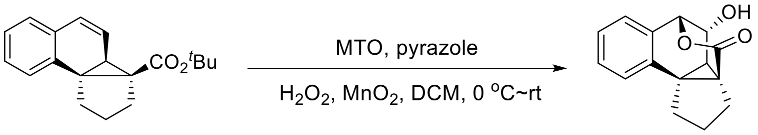

2b **7**

To a round bottom flask were added **2b** (84.6 mg, 0.3 mmol, 1.0 equiv.), MTO (CH_3O_3Re) (0.4 mg, 0.5 mol%), pyrazole (2.5 mg, 12 mol%), H_2O_2 (0.068 mL, 0.6 mmol, 2.0 equiv.) and DCM (2 mL). The resulting mixture was stirred at room temperature for 1 hour. When cooled to 0 °C, MnO_2 (2 mg) were added and the mixture was stirred at room temperature for 12 hours. Additional MTO (0.4 mg, 0.5 mol%) and H_2O_2 (0.068 mL, 0.6 mmol, 2.0 equiv) were added. After **2b** was consumed completely, the reaction mixture was quenched with Sat. aqueous $NaHCO_3$ and was extracted with EtOAc (10 mL × 3). The combined organic layers were washed with brine, dried over anhydrous Na_2SO_4 and concentrated in vacuo. The residue was purified by column chromatography on silica gel to afford product **7** as white solid (68.3 mg, 94%, 96% ee).

Compound 8

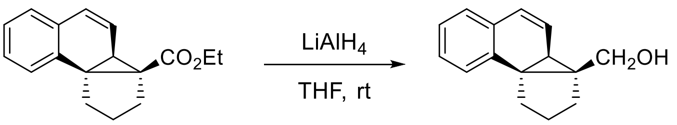

2c **8**

In a round-bottom flask, 25.4 mg of **2c** (0.1 mmol, 1.0 equiv.) was dissolved in 4 mL of dry THF. Then 5.7 mg of $LiAlH_4$ (0.15 mmol, 1.5 equiv.) was added in portion at 0 °C. The mixture was stirred at room temperature for 3 hours inert atmosphere. The reaction was quenched by addition of 2 mL of 1 M HCl solution and was extracted with 2x10 mL of ethyl acetate. The organic phase was dried with anhydrous $MgSO_4$ and filtered. After removal of the solvent under reduced pressure, the residue was purified by column chromatography on silica gel to afford product **8** as white solid (19.1 mg, 90%, 91% ee).

2. Characterization Data of Compounds

2.1 Characterization data of substrates 1

methyl 2-diazo-5-(naphthalen-1-yl)pentanoate (1a)

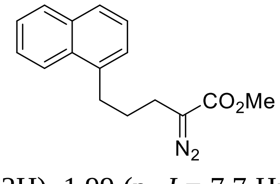

Yellow oil, 87% yield, ^{1}H NMR (300 MHz, Chloroform-*d*) δ 8.01 (d, *J* = 8.1 Hz, 1H), 7.87 (d, *J* = 7.5 Hz, 1H), 7.73 (d, *J* = 8.0 Hz, 1H), 7.50 (p, *J* = 6.7 Hz, 2H), 7.40 (t, *J* = 7.5 Hz, 1H), 7.33 (d, *J* = 6.9 Hz, 1H), 3.77 (s, 3H), 3.19 – 3.09 (t, *J* = 7.5 Hz, 2H), 2.45 (t, *J* = 7.5 Hz, 2H), 1.99 (p, *J* = 7.7 Hz, 2H); ^{13}C NMR (75 MHz, $CDCl_3$) δ 168.10, 137.48, 134.03, 131.83,

128.96, 126.98, 126.16, 126.01, 125.63, 123.69, 52.04, 32.16, 28.63, 23.25; HRMS (ESI+): Calculated for $[C_{16}H_{16}N_2O_2Na]^+$ ($[M+Na]^+$): 291.1104, Found: 291.1100.

tert-butyl 2-diazo-5-(naphthalen-1-yl)pentanoate (1b)

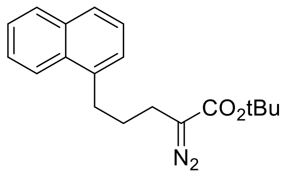

Yellow oil, 90% yield, ^{1}H NMR (300 MHz, Chloroform-*d*) δ 8.02 (d, *J* = 7.9 Hz, 1H), 7.89 – 7.84 (m, 1H), 7.73 (d, *J* = 8.0 Hz, 1H), 7.50 (p, *J* = 6.8 Hz, 2H), 7.43 – 7.37 (m, 1H), 7.33 (d, *J* = 6.9 Hz, 1H), 3.18 – 3.09 (t, *J* = 7.5 Hz, 2H), 2.40 (t, *J* = 7.5 Hz, 2H), 1.97 (p, *J* = 7.6 Hz, 2H), 1.48 (s, 9H); ^{13}C NMR (75 MHz, $CDCl_3$) δ 167.03, 137.56, 133.95, 131.77, 128.85, 126.84, 126.08, 125.89, 125.56, 125.51, 123.65, 81.17, 32.17, 28.63, 28.42, 23.24. HRMS (ESI+): Calculated for $[C_{19}H_{22}N_2O_2Na]^+$ ($[M+Na]^+$): 333.1573, Found: 333.1576.

ethyl 2-diazo-5-(naphthalen-1-yl)pentanoate (1c)

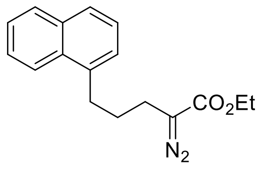

Yellow oil, 88% yield,^{1}H NMR (300 MHz, Chloroform-*d*) δ 8.01 (d, *J* = 7.9 Hz, 1H), 7.87 (d, *J* = 7.1 Hz, 1H), 7.73 (d, *J* = 8.0 Hz, 1H), 7.50 (p, *J* = 6.7 Hz, 2H), 7.44 – 7.37 (m, 1H), 7.33 (d, *J* = 6.7 Hz, 1H), 4.23 (q, *J* = 7.1 Hz, 2H), 3.20 – 3.10 (t, 2H), 2.45 (t, *J* = 7.5 Hz, 2H), 1.99 (p, *J* = 7.6 Hz, 2H), 1.27 (t, *J* = 6.1 Hz, 3H); ^{13}C NMR (75 MHz, $CDCl_3$) δ 167.76, 137.56, 134.06, 131.86, 128.97, 126.98, 126.18, 126.02, 125.65, 125.64, 123.72, 60.93, 32.20, 28.68, 23.28, 14.66; HRMS (ESI+): Calculated for $[C_{16}H_{16}N_2O_2Na]^+$ ($[M+Na]^+$): 305.1260, Found: 305.1257.

propyl 2-diazo-5-(naphthalen-1-yl)pentanoate (1d)

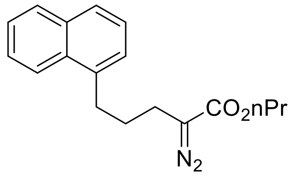

Yellow oil, 83% yield, ^{1}H NMR (300 MHz, Chloroform-*d*) δ 8.01 (d, *J* = 7.8 Hz, 1H), 7.88 – 7.83 (m, 1H), 7.72 (d, *J* = 8.0 Hz, 1H), 7.49 (p, *J* = 6.8 Hz, 2H), 7.40 (t, *J* = 7.6 Hz, 1H), 7.32 (d, *J* = 6.7 Hz, 1H), 4.13 (t, *J* = 6.6 Hz, 2H), 3.18 – 3.09 (t, *J* = 7.5 Hz, 2H), 2.44 (t, *J* = 7.5 Hz, 2H), 2.03 – 1.93 (m, 2H), 1.67 (dt, *J* = 14.2, 7.1 Hz, 2H), 0.93 (t, *J* = 7.4 Hz, 3H); ^{13}C NMR (75 MHz, $CDCl_3$) δ 167.84, 137.55, 134.05, 131.85, 128.97, 126.98, 126.18, 126.01, 125.65, 125.64, 123.72, 66.49, 32.21, 28.71, 23.30, 22.36, 10.47; HRMS (ESI+): Calculated for $[C_{18}H_{20}N_2O_2Na]^+$ ($[M+Na]^+$): 319.1417, Found: 319.1412.

neopentyl 2-diazo-5-(naphthalen-1-yl)pentanoate (1e)

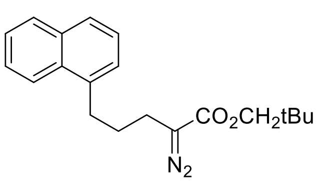

Yellow oil, 87% yield, ^{1}H NMR (300 MHz, Chloroform-*d*) δ 8.04 (d, *J* = 8.0 Hz, 1H), 7.89 (d, *J* = 7.2 Hz, 1H), 7.75 (d, *J* = 8.1 Hz, 1H), 7.52 (p, *J* = 6.7 Hz, 2H), 7.43 (t, *J* = 7.5 Hz, 1H), 7.35 (d, *J* = 6.8 Hz, 1H), 3.91 (s, 2H), 3.17 (t, *J* = 7.7 Hz, 2H), 2.47 (t, *J* = 7.5 Hz, 2H), 2.03 (p, *J* = 7.3 Hz, 2H), 0.97 (s, 9H); ^{13}C NMR (75 MHz, $CDCl_3$) δ 167.70, 137.44, 134.00, 131.79, 128.91, 126.93, 126.13, 125.95, 125.57, 123.65, 74.05, 32.16, 31.56, 30.29, 29.80, 28.71, 26.45, 23.24; HRMS (ESI+): Calculated for $[C_{20}H_{24}N_2O_2Na]^+$ ($[M+Na]^+$): 347.1730, Found: 347.1720.

benzyl 2-diazo-5-(naphthalen-1-yl)pentanoate (1f)

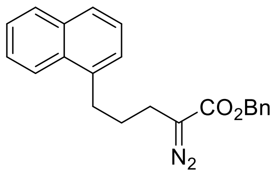

Yellow oil, 85% yield, ^{1}H NMR (300 MHz, Chloroform-*d*) δ 7.99 (d, *J* = 7.4 Hz, 1H), 7.89 – 7.83 (m, 1H), 7.72 (d, *J* = 8.1 Hz, 1H), 7.54 – 7.45 (m, 2H), 7.43 – 7.28 (m, 7H), 5.22 (s, 2H), 3.18 – 3.10 (t, *J* = 7.5 Hz, 2H), 2.46 (t, *J* = 7.5 Hz, 2H), 1.98 (q, *J* = 7.4 Hz, 2H); ^{13}C NMR (151 MHz, $CDCl_3$) δ 167.40, 137.39, 136.19, 133.97, 131.76, 128.89, 128.61, 128.23, 128.06, 126.91, 126.10, 125.95, 125.56, 123.62, 77.30, 77.09, 76.88, 66.41, 32.08, 28.56, 23.21. HRMS (ESI+): Calculated for $[C_{22}H_{20}N_2O_2Na]^+$ ($[M+Na]^+$): 367.1417, Found: 367.1401.

ethyl 2-diazo-5-(4-fluoronaphthalen-1-yl)pentanoate (1g)

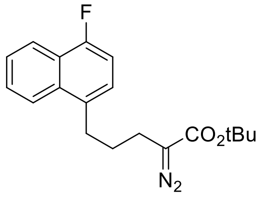

Yellow oil, 90% yield, ^{1}H NMR (300 MHz, Chloroform-*d*) δ 8.21 – 8.12 (m, 1H), 8.01 (dt, *J* = 7.3, 2.0 Hz, 1H), 7.64 – 7.53 (m, 2H), 7.25 (dd, *J* = 7.9, 5.5 Hz, 1H), 7.08 (dd, *J* = 10.4, 7.8 Hz, 1H), 3.15 – 3.07 (t, *J* = 7.5 Hz, 2H), 2.40 (t, *J* = 7.5 Hz, 2H), 2.02 – 1.90 (m, 2H), 1.50 (s, 9H). ^{13}C NMR (75 MHz, $CDCl_3$) δ 166.99, 159.33, 156.01, 133.35, 133.29, 132.83, 132.77, 126.79, 125.81, 125.78, 125.58, 125.47, 124.16, 123.95, 123.73, 123.69, 121.31, 121.24, 108.97, 108.71, 81.21, 77.46, 77.03, 76.61, 31.74, 28.65, 28.38, 23.14. HRMS (ESI+): Calculated for $[C_{19}H_{21}FN_2O_2Na]^+$ ($[M+Na]^+$): 351.1479, Found: 351.1474.

ethyl 5-(5-bromonaphthalen-1-yl)-2-diazopentanoate (1h)

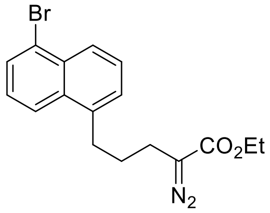

Yellow oil, 81% yield, ^{1}H NMR (300 MHz, Chloroform-*d*) δ 8.19 (d, *J* = 8.5 Hz, 1H), 8.01 (d, *J* = 8.5 Hz, 1H), 7.81 (dd, *J* = 7.4, 1.0 Hz, 1H), 7.53 (dd, *J* = 8.6, 7.0 Hz, 1H), 7.43 – 7.33 (m, 2H), 4.25 (q, *J* = 7.1 Hz, 2H), 3.20 – 3.11 (t, *J* = 7.5 Hz, 2H), 2.45 (t, *J* = 7.5 Hz, 2H), 1.98 (q, *J* = 7.5 Hz, 2H), 1.29 (t, *J* = 7.1 Hz, 3H). ^{13}C NMR (75 MHz, $CDCl_3$) δ 167.55, 137.95, 133.04, 132.44, 129.79, 127.02, 126.94, 126.11, 126.07, 123.84, 123.57, 60.86, 32.26, 28.65, 23.18, 14.55. Calculated for $[C_{17}H_{17}BrN_2O_2Na]^+$ ($[M+Na]^+$): 383.0366, Found: 383.0356.

tert-butyl 5-(6-(benzyloxy)naphthalen-1-yl)-2-diazopentanoate (1i)

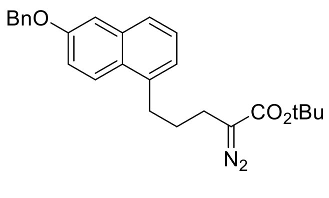

Yellow oil, 88% yield, ^{1}H NMR (300 MHz, Chloroform-*d*) δ 7.85 (dt, *J* = 8.6, 1.0 Hz, 1H), 7.52 (d, *J* = 8.2 Hz, 1H), 7.45 – 7.38 (m, 2H), 7.36 – 7.23 (m, 4H), 7.20 – 7.15 (m, 2H), 7.09 (dd, *J* = 7.1, 1.2 Hz, 1H), 5.10 (s, 2H), 3.05 – 2.97 (t, *J* = 7.5 Hz, 2H), 2.30 (t, *J* = 7.5 Hz, 2H), 1.91 – 1.81 (m, 2H), 1.39 (s, 9H). ^{13}C NMR (75 MHz, $CDCl_3$) δ 167.05, 156.47, 137.66, 136.96, 135.23, 128.68, 128.08, 127.64, 127.35, 126.28, 125.84, 125.39, 124.13, 118.88, 108.16, 81.19, 70.05, 32.25, 28.76, 28.46, 23.26. HRMS (ESI+): Calculated for $[C_{26}H_{28}N_2O_3Na]^+$ ($[M+Na]^+$): 439.1992, Found: 439.1982.

tert-butyl 5-(6-(allyloxy)naphthalen-1-yl)-2-diazopentanoate (1j)

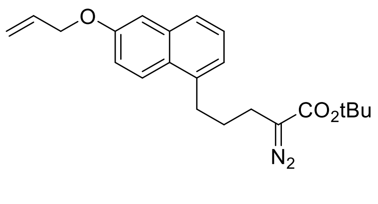

Yellow oil, 89% yield, ^{1}H NMR (300 MHz, Chloroform-*d*) δ 7.94 (d, *J* = 9.1 Hz, 1H), 7.62 (d, *J* = 8.2 Hz, 1H), 7.37 (dd, *J* = 8.2, 7.0 Hz, 1H), 7.25 – 7.16 (m, 3H), 6.16 (ddt, *J* = 17.2, 10.6, 5.3 Hz, 1H), 5.57 – 5.33 (m, 2H), 4.69 (dt, *J* = 5.3, 1.6

Hz, 2H), 3.19 – 3.03 (t, J = 7.5 Hz, 2H), 2.40 (t, J = 7.5 Hz, 2H), 2.06 – 1.88 (m, 2H), 1.50 (s, 9H). ^{13}C NMR (75 MHz, $CDCl_3$) δ 166.92, 158.73, 143.06, 133.43, 129.36, 120.97, 117.49, 114.99, 112.07, 81.06, 68.67, 35.00, 29.18, 28.39, 22.74. Calculated for $[C_{22}H_{26}N_2O_3Na]^+$ ($[M+Na]^+$):389.1836, Found: 389.1827

tert-butyl 2-diazo-5-(6-((3-phenylprop-2-yn-1-yl)oxy)naphthalen-1-yl)pentanoate (1k)

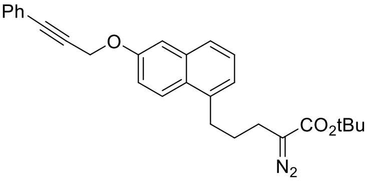

Yellow oil, 85% yield, ^{1}H NMR (300 MHz, Chloroform-*d*) δ 7.88 (d, J = 9.2 Hz, 1H), 7.57 (d, J = 8.3 Hz, 1H), 7.38 (dd, J = 7.3, 2.4 Hz, 2H), 7.33 – 7.29 (m, 1H), 7.30 – 7.14 (m, 5H), 7.13 (d, J = 7.0 Hz, 1H), 4.97 (s, 2H), 3.08 – 2.97 (t, J = 7.8 Hz, 2H), 2.31 (t, J = 7.5 Hz, 2H), 1.88 (p, J = 7.5 Hz, 2H), 1.40 (s, 9H). ^{13}C NMR (75 MHz, $CDCl_3$) δ 167.00, 155.40, 137.64, 135.04, 131.86, 128.71, 128.30, 127.51, 126.29, 125.93, 125.41, 124.29, 122.26, 118.62, 108.48, 87.36, 83.80, 81.18, 56.72, 32.22, 28.71, 28.40, 23.22. Calculated for $[C_{28}H_{28}N_2O_3Na]^+$ ($[M+Na]^+$):463.1992, Found:463.1984.

tert-butyl 2-diazo-5-(6-morpholinonaphthalen-1-yl)pentanoate (1l)

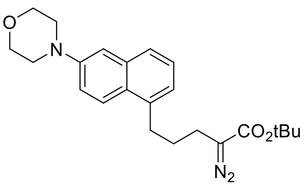

Yellow oil, 82% yield, ^{1}H NMR (300 MHz, Chloroform-*d*) δ 7.92 (d, J = 9.2 Hz, 1H), 7.58 (d, J = 8.2 Hz, 1H), 7.38 – 7.28 (m, 2H), 7.15 (dd, J = 6.7, 1.7 Hz, 2H), 3.99 – 3.87 (m, 4H), 3.33 – 3.22 (m, 4H), 3.14 – 3.03 (t, J = 4.8 Hz 2H), 2.38 (t, J = 7.5 Hz, 2H), 2.00 – 1.87 (m, 2H), 1.48 (s, 9H). ^{13}C NMR (75 MHz, $CDCl_3$) δ 167.03, 148.71, 137.40, 135.12, 126.84, 126.16, 125.75, 124.73, 123.87, 118.64, 110.96, 81.14, 66.96, 49.70, 32.08, 28.70, 28.40, 23.17.HRMS (ESI+): Calculated for $[C_{23}H_{29}N_3O_3Na]^+$ ($[M+Na]^+$):418.2101, Found: 418.2093

tert-butyl 2-diazo-5-(6-(((trifluoromethyl)sulfonyl)oxy)naphthalen-1-yl)pentanoate (1m)

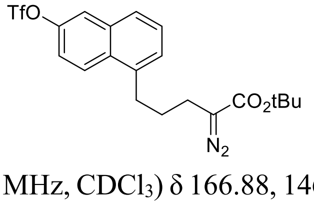

Yellow oil, 87% yield, ^{1}H NMR (300 MHz, Chloroform-*d*) δ 8.12 (d, J = 9.3 Hz, 1H), 7.83 – 7.72 (t, J = 7.5 Hz 2H), 7.60 – 7.49 (m, 1H), 7.49 – 7.37 (m, 2H), 3.20 – 3.10 (t, J = 7.5 Hz, 2H), 2.42 (t, J = 7.5 Hz, 2H), 1.98 (p, J = 7.6 Hz, 2H), 1.49 (s, 9H).^{13}C NMR (75 MHz, $CDCl_3$) δ 166.88, 146.92, 138.06, 134.12, 130.82, 127.49, 127.35, 126.94, 126.58, 119.95, 119.40, 81.28, 32.14, 28.63, 28.37, 23.24. HRMS (ESI+): Calculated for $[C_{20}H_{21}F_3N_2O_5SNa]^+$ ($[M+Na]^+$):481.1015, Found: 481.1025.

tert-butyl 2-diazo-5-(6-vinylnaphthalen-1-yl)pentanoate (1n)

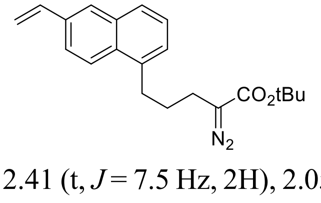

Yellow oil, 87% yield, ^{1}H NMR (300 MHz, Chloroform-*d*) δ 8.08 – 7.95 (m, 1H), 7.83 – 7.65 (m, 3H), 7.48 – 7.38 (m, 1H), 7.36 – 7.27 (m, 1H), 6.91 (dd, J = 17.6, 10.9 Hz, 1H), 5.91 (d, J = 17.6 Hz, 1H), 5.37 (d, J = 10.8 Hz, 1H), 3.18 – 3.08 (t, J = 7.5 Hz, 2H), 2.41 (t, J = 7.5 Hz, 2H), 2.05 – 1.93 (m, 2H), 1.50 (s, 9H). ^{13}C NMR (75 MHz, $CDCl_3$) δ 167.00, 137.53, 136.75, 134.63, 134.07, 131.40, 127.21, 126.98, 126.18, 125.97, 123.96, 123.19, 114.24, 81.18, 32.12, 28.66, 28.40, 23.19. HRMS (ESI+): Calculated for $[C_{21}H_{25}N_2O_2]^+$ ($[M+H]^+$):337.1911, Found: 337.1935

tert-butyl 5-(6-allylnaphthalen-1-yl)-2-diazopentanoate (1o)

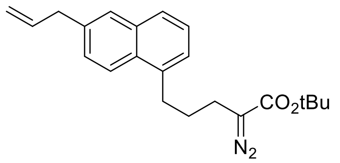

Yellow oil, 87% yield, ^{1}H NMR (300 MHz, Chloroform-*d*) δ 7.94 (d, *J* = 8.7 Hz, 1H), 7.69 – 7.61 (m, 2H), 7.36 (ddd, *J* = 8.1, 4.2, 2.3 Hz, 2H), 7.28 – 7.23 (m, 1H), 6.05 (ddt, *J* = 16.8, 10.1, 6.6 Hz, 1H), 5.21 – 5.06 (m, 2H), 3.55 (d, *J* = 6.9 Hz, 2H), 3.18 – 3.01 (t, *J* = 7.5 Hz, 2H), 2.38 (t, *J* = 7.5 Hz, 2H), 2.01 – 1.89 (m, 2H), 1.47 (s, 9H). ^{13}C NMR (75 MHz, $CDCl_3$) δ 167.06, 137.41, 137.28, 137.18, 134.17, 130.39, 127.61, 127.38, 126.43, 125.68, 125.54, 123.76, 116.09, 81.16, 40.18, 32.16, 28.64, 28.40, 23.19. HRMS (ESI+): Calculated for $[C_{22}H_{26}N_2O_2Na]^+$ ($[M+Na]^+$):373.1886, Found: 373.1870

methyl 4-(5-(5-(tert-butoxy)-4-diazo-5-oxopentyl)naphthalen-2-yl)benzoate (1p)

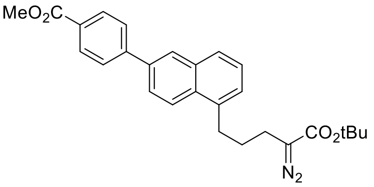

Yellow oil, 81% yield, ^{1}H NMR (300 MHz, Chloroform-*d*) δ 8.20 – 8.07 (m, 4H), 7.84 – 7.80 (m, 4H), 7.47 (dd, *J* = 8.1, 7.0 Hz, 1H), 7.38 (d, *J* = 6.4 Hz, 1H), 3.98 (s, 3H), 3.28 – 3.08 (t, *J* = 7.5 Hz, 2H), 2.43 (t, *J* = 7.5 Hz, 2H), 2.12 – 1.93 (m, 2H), 1.50 (s, 9H). ^{13}C NMR (75 MHz, $CDCl_3$) δ 166.99, 145.32, 137.57, 136.83, 134.11, 131.32, 130.20, 128.94, 127.29, 127.22, 127.19, 126.63, 126.28, 125.16, 124.53, 81.19, 52.16, 32.11, 28.66, 28.41, 23.22. HRMS (ESI+): Calculated for $[C_{27}H_{28}N_2O_4Na]^+$ ($[M+Na]^+$):467.1941, Found: 467.1926

tert-butyl 5-([2,2'-binaphthalen]-5-yl)-2-diazopentanoate (1q)

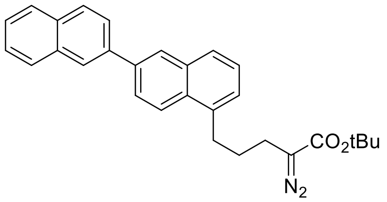

Yellow oil, 85% yield, ^{1}H NMR (300 MHz, Chloroform-*d*) δ 8.22 (t, *J* = 2.2 Hz, 2H), 8.02 – 7.90 (m, 6H), 7.58 – 7.44 (m, 4H), 7.40 – 7.35 (m, 1H), 3.21 (t, *J* = 7.7 Hz, 2H), 2.46 (t, *J* = 7.5 Hz, 2H), 2.04 (dd, *J* = 10.2, 5.1 Hz, 2H), 1.53 (s, 9H). ^{13}C NMR (75 MHz, $CDCl_3$) δ 167.09, 138.26, 138.03, 137.57, 134.33, 133.80, 132.73, 131.02, 128.59, 128.30, 127.73, 127.23, 127.01, 126.40, 126.27, 126.15, 126.09, 126.06, 125.71, 124.41, 81.23, 77.55, 77.12, 76.70, 32.19, 28.71, 28.46, 23.26. HRMS (ESI+): Calculated for $[C_{29}H_{28}N_2O_2Na]^+$ ($[M+Na]^+$):459.2043, Found: 459.2063

tert-butyl 2-diazo-5-(6-phenylnaphthalen-1-yl)pentanoate (1r)

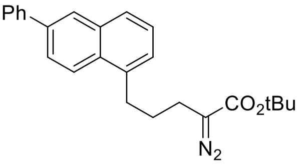

Yellow oil, 86% yield, ^{1}H NMR (300 MHz, Chloroform-*d*) δ 8.16 – 8.10 (m, 2H), 7.81 (ddd, *J* = 12.9, 8.2, 2.0 Hz, 4H), 7.57 – 7.43 (m, 4H), 7.38 (d, *J* = 7.0 Hz, 1H), 3.24 – 3.18 (t, *J* = 7.5 Hz, 2H), 2.47 (t, *J* = 7.5 Hz, 2H), 2.05 (p, *J* = 7.6 Hz, 2H), 1.54 (s, 9H). ^{13}C NMR (75 MHz, $CDCl_3$) δ 167.03, 140.97, 138.18, 137.52, 134.25, 130.95, 128.91, 127.41, 127.18, 126.70, 126.19, 126.07, 125.56, 124.30, 81.20, 77.53, 77.10, 76.68, 32.19, 28.70, 28.44, 23.26. HRMS (ESI+): Calculated for $[C_{25}H_{26}N_2O_2Na]^+$ ($[M+Na]^+$): 409.1886, Found: 409.1877.

tert-butyl 2-diazo-5-(6-(thiophen-2-yl)naphthalen-1-yl)pentanoate (1s)

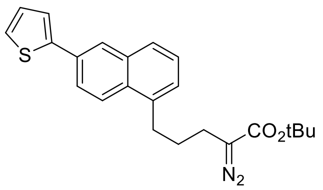

Yellow oil, 88% yield, ^{1}H NMR (300 MHz, Chloroform-*d*) δ 8.08 – 7.99 (m, 2H), 7.82 – 7.71 (m, 2H), 7.48 – 7.38 (m, 2H), 7.32 (ddd, *J* = 8.2, 6.1, 1.2 Hz, 2H), 7.14 (dd, *J* = 5.1, 3.6 Hz, 1H), 3.18 – 3.07 (t, *J* = 7.5 Hz, 2H), 2.41 (t, *J* = 7.5 Hz, 2H), 1.98 (h, *J* = 7.5, 6.9 Hz, 2H), 1.49 (s, 9H). ^{13}C NMR (75 MHz, $CDCl_3$) δ 167.03, 147.31, 144.32, 137.60, 134.19, 131.46, 131.03, 128.18, 126.97, 126.31, 126.20, 125.10, 125.07, 124.40, 123.49, 81.21, 32.10, 28.67, 28.43, 23.23. HRMS (ESI+): Calculated for $[C_{23}H_{24}N_2O_2SNa]^+$ ($[M+Na]^+$): 415.1451, Found: 415.1451.

tert-butyl 5-(6-cyanonaphthalen-1-yl)-2-diazopentanoate (1t)

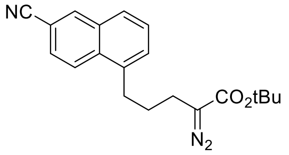

Yellow oil, 81% yield, ^{1}H NMR (300 MHz, Chloroform-*d*) δ 8.24 (d, *J* = 1.7 Hz, 1H), 8.10 (dt, *J* = 8.8, 0.8 Hz, 1H), 7.78 (dd, *J* = 7.6, 1.9 Hz, 1H), 7.65 (dd, *J* = 8.8, 1.8 Hz, 1H), 7.57 – 7.48 (m, 2H), 3.19 – 3.10 (t, *J* = 7.5 Hz, 2H), 2.41 (t, *J* = 7.5 Hz, 2H), 2.03 – 1.91 (m, 2H), 1.49 (s, 9H). ^{13}C NMR (75 MHz, $CDCl_3$) δ 166.86, 138.08, 134.93, 133.10, 132.84, 129.20, 127.40, 127.26, 126.37, 125.06, 119.21, 109.08, 81.29, 31.93, 28.61, 28.38, 23.24. HRMS (ESI+): Calculated for $[C_{20}H_{21}N_3O_2Na]^+$ ($[M+Na]^+$):358.1526, Found: 358.1505

tert-butyl 2-diazo-5-(8-(methoxymethyl)naphthalen-1-yl)pentanoate (1u)

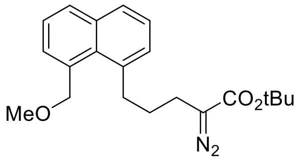

Yellow oil, 82% yield, ^{1}H NMR (300 MHz, Chloroform-*d*) δ 7.90 – 7.83 (m, 1H), 7.76 (dd, *J* = 6.3, 3.3 Hz, 1H), 7.54 (dd, *J* = 7.1, 1.4 Hz, 1H), 7.45 – 7.37 (m, 3H), 4.89 (s, 2H), 3.44 (s, 3H), 3.36 – 3.29 (t, *J* = 7.8 Hz, 2H), 2.44 (t, *J* = 7.6 Hz, 2H), 1.89 (p, *J* = 7.8 Hz, 2H), 1.50 (s, 9H). ^{13}C NMR (75 MHz, $CDCl_3$) δ 168.20, 138.49, 135.98, 133.30, 130.96, 130.88, 130.78, 129.58, 128.35, 125.27, 124.56, 81.15, 76.20, 57.67, 34.86, 31.09, 28.40, 23.23. HRMS (ESI+): Calculated for $[C_{21}H_{26}N_2O_3Na]^+$ ($[M+Na]^+$):377.1836, Found: 377.1839.

tert-butyl 2-diazo-5-(1,2-dihydroacenaphthylen-5-yl)pentanoate (1v)

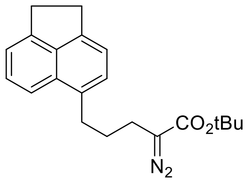

Yellow oil, 88% yield, ^{1}H NMR (300 MHz, Chloroform-*d*) δ 7.73 – 7.68 (m, 1H), 7.49 (dd, *J* = 8.4, 6.8 Hz, 1H), 7.36 – 7.18 (m, 3H), 3.51 – 3.32 (m, 4H), 3.20 – 3.03 (t, *J* = 7.5 Hz, 2H), 2.51 – 2.31 (t, *J* = 7.8 Hz, 2H), 2.06 – 1.89 (m, 2H), 1.51 (s, 9H). ^{13}C NMR (75 MHz, $CDCl_3$) δ 167.08, 146.56, 144.31, 139.60, 133.30, 130.28, 127.61, 127.46, 119.12, 119.08, 119.01, 81.12, 31.20, 30.57, 29.87, 28.70, 28.41, 23.09. HRMS (ESI+): Calculated for $[C_{21}H_{24}N_2O_2Na]^+$ ($[M+Na]^+$): 359.1730, Found: 359.1738.

2.2 Characterization data of products 2

methyl (3aR,3bR,9bR)-2,3-dihydro-1H-cyclopenta[1,3]cyclopropa[1,2-a]naphthalene-3a(3bH)-carboxylate (2a)

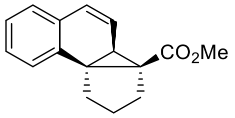

Light yellow oil, 84% yield, 85% ee; ^{1}H NMR (300 MHz, Chloroform-*d*) δ 7.61 (d, *J* = 7.2 Hz, 1H), 7.32 – 7.22 (m, 2H), 7.20 (t, *J* = 5.2 Hz, 1H), 6.61 (d, *J* = 9.6 Hz, 1H), 6.12 (dd, *J* = 9.5, 5.3 Hz, 1H), 3.44 (s, 3H),

2.84 (td, J = 12.0, 8.8 Hz, 1H), 2.46 (dd, J = 10.9, 4.3 Hz, 2H), 2.38 (d, J = 8.4 Hz, 1H), 2.04 (dd, J = 12.6, 7.7 Hz, 1H), 1.85 (dd, J = 13.5, 8.0 Hz, 1H), 1.39 (d, J = 11.5 Hz, 1H); ^{13}C NMR (75 MHz, $CDCl_3$) δ 169.11, 133.08, 132.27, 128.63, 127.53, 127.25, 126.39, 125.84, 122.69, 51.35, 41.55, 31.24, 31.07, 30.86, 26.56, 18.84; HRMS (ESI+): Calculated for $[C_{16}H_{17}O_2]^+$ ($[M+H]^+$): 241.1222, Found: 241.1230. HPLC conditions (Agilent 1260): Chiralpak IC (Particle Size: 5 μm, Dimensions: 4.6 mm Φ × 250 mmL); detected at 254 nm; temperature 25 °C; *i*-propanol/hexane=10/90; flow=0.5 mL/min; Retention time: 9.136 min(major), 9.977 min(minor).

tert-butyl (3aR,3bR,9bR)-2,3-dihydro-1H-cyclopenta[1,3]cyclopropa[1,2-a]naphthalene-3a(3bH)-carboxylate (2b)

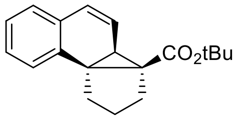

Light yellow oil, 80% yield, 99% ee; ^{1}H NMR (300 MHz, Chloroform-*d*) δ 7.57 (d, J = 7.7 Hz, 1H), 7.24 – 7.06 (m, 3H), 6.45 (d, J = 9.6 Hz, 1H), 6.15 (dd, J = 9.6, 5.2 Hz, 1H), 2.79 (td, J = 12.1, 8.6 Hz, 1H), 2.43 – 2.25 (m, 3H), 1.97 (dd, J = 12.6, 7.7 Hz, 1H), 1.79 (dt, J = 15.8, 8.1 Hz, 1H), 1.28 (m, 1H), 1.06 (s, 9H); ^{13}C NMR (75 MHz, $CDCl_3$) δ 169.62, 143.02, 141.86, 138.24, 131.42, 127.97, 122.62, 121.96, 112.02, 79.46, 39.95, 31.69, 30.32, 29.86, 27.90, 27.57, 19.32. HRMS (ESI+): Calculated for $[C_{19}H_{22}O_2Na]^+$ ($[M+Na]^+$): 305.1512, Found: 305.1517. HPLC conditions (Agilent 1260): Chiralpak OJ-H (Particle Size: 5 μm, Dimensions: 4.6 mm Φ × 250 mmL); detected at 254 nm; temperature 25 °C, *i*-propanol/hexane=10/90; flow=0.1 mL/min; Retention time: 44.811 min(minor), 47.513 min(major).

ethyl (3aR,3bR,9bR)-2,3-dihydro-1H-cyclopenta[1,3]cyclopropa[1,2-a]naphthalene-3a(3bH)-carboxylate (2c)

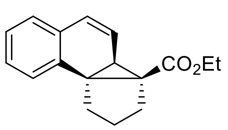

Light yellow oil, 86% yield, 91% ee; ^{1}H NMR (300 MHz, Chloroform-*d*) δ 7.59 (d, J = 7.4 Hz, 1H), 7.28 – 7.18 (m, 2H), 7.15 (t, J = 6.9 Hz, 1H), 6.54 (d, J = 9.6 Hz, 1H), 6.12 (dd, J = 9.6, 5.3 Hz, 1H), 3.84 (qq, J = 10.8, 7.1 Hz, 2H), 2.82 (td, J = 12.0, 8.6 Hz, 1H), 2.46 – 2.29 (m, 3H), 2.01 (dd, J = 12.6, 7.7 Hz, 1H), 1.81 (dt, J = 15.9, 8.1 Hz, 1H), 1.40 – 1.28 (m, 1H), 0.91 (t, J = 7.1 Hz, 3H); ^{13}C NMR (75 MHz, $CDCl_3$) δ 169.18, 133.01, 132.56, 128.37, 127.51, 127.20, 126.28, 125.87, 123.10, 60.00, 41.22, 31.27, 31.06, 30.64, 27.09, 18.95, 14.00; HRMS (ESI+): Calculated for $[C_{17}H_{19}O_2]^+$ ($[M+H]^+$): 255.1380, Found: 255.1390. HPLC conditions (Agilent 1260): Chiralpak IC (Particle Size: 5 μm, Dimensions: 4.6 mm Φ × 250 mmL); detected at 254 nm; temperature 25℃; *i*-propanol/hexane=8/92; flow=0.3 mL/min; Retention time: 15.220 min(major), 16.155 min(minor).

propyl (3aR,3bR,9bR)-2,3-dihydro-1H-cyclopenta[1,3]cyclopropa[1,2-a]naphthalene-3a(3bH)-carboxylate (2d)

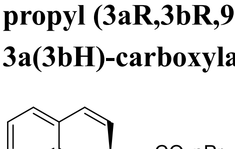

Colorless oil, 92% yield, 88% ee; ^{1}H NMR (300 MHz, Chloroform-*d*) δ 7.58 (d, J = 7.3 Hz, 1H), 7.25 – 7.17 (m, 2H), 7.17 – 7.11 (m, 1H), 6.53 (d, J = 9.6 Hz, 1H), 6.11 (dd, J = 9.5, 5.3 Hz, 1H), 3.83 – 3.65 (m, 2H), 2.81 (td, J = 11.8, 8.5 Hz, 1H), 2.38 (qd, J = 12.6, 12.1, 6.9 Hz, 3H), 2.01 (dt, J = 12.4, 5.5 Hz, 1H), 1.80 (dt, J = 15.1, 8.1 Hz, 1H), 1.32 – 1.28 (m, 2H), 0.89 (d, J = 7.3 Hz, 1H), 0.69 (t, J = 7.4 Hz, 3H); ^{13}C NMR (75 MHz, $CDCl_3$) δ 169.36, 133.00, 132.58, 128.40,

127.57, 127.24, 126.33, 125.90, 123.13, 65.82, 41.25, 31.35, 31.17, 30.67, 29.85, 21.87, 18.93, 10.41; HRMS (ESI+): Calculated for $[C_{18}H_{21}O_2]^+$ ($[M+H]^+$): 269.1536, Found: 269.1546. HPLC conditions (Agilent 1260): Chiralpak IC (Particle Size: 5 μm, Dimensions: 4.6 mm Φ × 250 mmL); detected at 254 nm; temperature 25°C; *i*-propanol/hexane=5/95; flow=0.3 mL/min; Retention time: 16.880 min(major), 17.975 min(minor).

neopentyl (3aR,3bR,9bR)-2,3-dihydro-1H-cyclopenta[1,3]cyclopropa[1,2-a]naphthalene-3a(3bH)-carboxylate (2e)

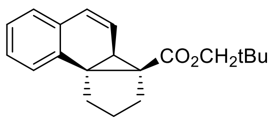

Light yellow oil, 61% yield, 92% ee; ^{1}H NMR (600 MHz, Chloroform-*d*) δ 7.58 (d, *J* = 7.7 Hz, 1H), 7.23 (td, *J* = 7.5, 1.6 Hz, 1H), 7.18 (td, *J* = 7.4, 1.3 Hz, 1H), 7.13 (dd, *J* = 7.5, 1.5 Hz, 1H), 6.53 (d, *J* = 9.6 Hz, 1H), 6.12 (dd, *J* = 9.7, 5.2 Hz, 1H), 3.54 – 3.46 (m, 2H), 2.82 (td, *J* = 12.0, 8.2 Hz, 1H), 2.46 – 2.40 (m, 2H), 2.36 (dd, *J* = 12.8, 8.2 Hz, 1H), 2.01 (dd, *J* = 12.5, 7.7 Hz, 1H), 1.81 (dt, *J* = 13.5, 8.2 Hz, 1H), 1.35 – 1.28 (m, 1H), 0.72 (s, 9H). ^{13}C NMR (151 MHz, $CDCl_3$) δ 169.01, 132.72, 132.36, 128.24, 127.61, 127.18, 126.23, 125.78, 123.04, 73.55, 41.08, 31.25, 31.21, 31.01, 30.39, 27.21, 26.28, 18.80. HRMS (ESI+): Calculated for $[C_{20}H_{24}O_2Na]^+$ ($[M+Na]^+$): 319.1669, Found: 319.1665, HPLC conditions (Agilent 1260): Chiralpak IC (Particle Size: 5 μm, Dimensions: 4.6 mm Φ × 250 mmL); detected at 254 nm; temperature 25°C; *i*-propanol/hexane=1/99; flow=0.5 mL/min; Retention time: 10.082 min(major), 10.956 min(minor).

benzyl (3aR,3bR,9bR)-2,3-dihydro-1H-cyclopenta[1,3]cyclopropa[1,2-a]naphthalene-3a(3bH)-carboxylate (2g)

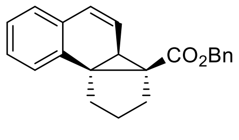

Colorless oil, 95% yield, 91% ee; ^{1}H NMR (300 MHz, Chloroform-*d*) δ 7.62 (d, *J* = 7.5 Hz, 1H), 7.31 – 7.24 (m, 4H), 7.22 (dd, *J* = 7.4, 1.4 Hz, 1H), 7.12 (dd, *J* = 7.3, 1.6 Hz, 1H), 7.03 (dd, *J* = 6.5, 3.0 Hz, 2H), 6.52 (d, *J* = 9.7 Hz, 1H), 6.14 (dd, *J* = 9.7, 5.3 Hz, 1H), 4.99 – 4.75 (m, 2H), 2.85 (td, *J* = 12.1, 8.3 Hz, 1H), 2.56 – 2.33 (m, 3H), 2.04 (dd, *J* = 12.7, 7.8 Hz, 1H), 1.84 (dt, *J* = 13.9, 8.2 Hz, 1H), 1.44 – 1.26 (m, 1H). ^{13}C NMR (151 MHz, $CDCl_3$) δ 168.82, 136.05, 132.23, 128.39, 128.25, 127.84, 127.74, 127.60, 127.15, 126.30, 125.85, 122.88, 77.23, 77.02, 76.81, 65.77, 41.42, 31.20, 31.01, 30.66, 26.89, 18.79. HRMS (ESI+): Calculated for $[C_{22}H_{21}O_2]^+$ ($[M+H]^+$): 317.1536, Found: 317.1550. HPLC conditions (Agilent 1260): Chiralpak IC (Particle Size: 5 μm, Dimensions: 4.6 mm Φ × 250 mmL); detected at 254 nm; temperature 25°C; *i*-propanol/hexane=10/90; flow=0.3 mL/min; Retention time: 16.912 min(major), 18.999 min(minor).

tert-butyl (3aR,3bR,9bR)-5-fluoro-2,3-dihydro-1H-cyclopenta[1,3]cyclopropa[1,2-a]naphthalene-3a(3bH)-carboxylate (2g)

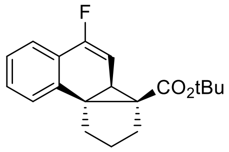

Colorless oil, 68% yield, 96% ee. ^{1}H NMR (300 MHz, Chloroform-*d*) δ 7.61 (dt, *J* = 7.5, 1.8 Hz, 1H), 7.50 (dd, *J* = 7.5, 1.7 Hz, 1H), 7.37 – 7.25 (m, 2H), 5.70 (dd, *J* = 13.6, 5.7 Hz, 1H), 2.78 (td, *J* = 12.0, 8.4 Hz, 1H), 2.47 – 2.27 (m, 3H), 1.99 (dd, *J* = 12.6, 7.7 Hz, 1H), 1.81 (dt, *J* = 13.3, 8.2 Hz, 1H), 1.48 – 1.38 (m, 1H), 1.11 (s, 9H). ^{13}C NMR (75 MHz, $CDCl_3$) δ 169.21, 154.96, 134.89, 128.13, 126.17, 126.12, 125.99, 120.87, 120.79, 100.55, 100.25, 80.33, 39.35, 31.43,

31.06, 27.59, 27.50, 27.36, 18.75. HRMS (ESI+): Calculated for $[C_{19}H_{21}FO_2Na]^+$ ($[M+Na]^+$): 323.1418, Found: 323.1406 HPLC conditions (Agilent 1260): Chiralpak IB (Particle Size: 5 μm, Dimensions: 4.6 mm Φ × 250 mmL); detected at 210 nm; temperature 25 ºC; *i*-propanol/hexane=5/95; flow=0.5 mL/min; Retention time: 9.471 min(major), 11.014 min(minor).

ethyl (3aR,3bR,9bR)-6-bromo-2,3-dihydro-1H-cyclopenta[1,3]cyclopropa[1,2-a]naphthalene-3a(3bH)-carboxylate (2h)

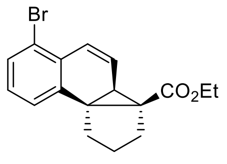

Colorless oil, 51% yield, 99% ee. ^{1}H NMR (300 MHz, Chloroform-*d*) δ 7.56 (d, *J* = 7.7 Hz, 1H), 7.51 – 7.35 (m, 1H), 7.13 – 6.98 (m, 2H), 6.26 (dd, *J* = 9.8, 5.4 Hz, 1H), 3.96 – 3.77 (m, 2H), 2.89 – 2.72 (m, 1H), 2.48 – 2.34 (m, 3H), 2.07 – 1.94 (m, 2H), 1.83 (dd, *J* = 14.3, 6.9 Hz, 1H), 1.48 – 1.35 (m, 1H), 0.92 (t, *J* = 7.1 Hz, 3H). ^{13}C NMR (75 MHz, $CDCl_3$) δ 169.12, 148.84, 130.55, 130.10, 127.68, 126.31, 125.55, 125.14, 122.87, 60.10, 41.14, 31.44, 30.94, 30.63, 30.20, 18.77, 13.78. HRMS (ESI+): Calculated for $[C_{17}H_{17}BrO_2Na]^+$ ($[M+Na]^+$): 355.0304, Found: 355.0298. HPLC conditions (Agilent 1260): Chiralpak OJ (Particle Size: 5 μm, Dimensions: 4.6 mm Φ × 250 mmL); detected at 254 nm; temperature 25 ºC; *i*-propanol/hexane=8/92; flow=0.5 mL/min; Retention time: 11.436 min(minor), 12.554 min(major).

tert-butyl (3aR,3bR,9bR)-7-(benzyloxy)-2,3-dihydro-1H-cyclopenta[1,3]cyclopropa[1,2-a]naphthalene-3a(3bH)-carboxylate (2i)

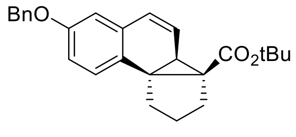

Colorless oil, 78% yield, 95% ee. ^{1}H NMR (300 MHz, Chloroform-*d*) δ 7.52 – 7.29 (m, 6H), 6.89 (dd, *J* = 8.6, 2.8 Hz, 1H), 6.77 (d, *J* = 2.8 Hz, 1H), 6.42 (d, *J* = 9.6 Hz, 1H), 6.19 (dd, *J* = 9.7, 5.2 Hz, 1H), 5.09 (s, 2H), 2.82 – 2.67 (m, 1H), 2.48 – 2.23 (m, 3H), 1.97 (dd, *J* = 12.5, 7.7 Hz, 1H), 1.85 – 1.73 (m, 1H), 1.33 (d, *J* = 12.7 Hz, 1H), 1.10 (s, 9H). ^{13}C NMR (75 MHz, $CDCl_3$) δ 169.19, 156.93, 137.22, 133.77, 128.54, 127.85, 127.52, 127.39, 126.81, 125.65, 124.90, 113.65, 113.47, 79.94, 69.98, 40.18, 31.40, 31.34, 29.22, 28.51, 27.71, 18.96. HRMS (ESI+): Calculated for $[C_{26}H_{28}O_3Na]^+$ ($[M+Na]^+$): 411.1931, Found: 411.1921. HPLC conditions (Agilent 1260): Chiralpak OJ (Particle Size: 5 μm, Dimensions: 4.6 mm Φ × 250 mmL); detected at 254 nm; temperature 25 ºC; *i*-propanol/hexane=5/95; flow=0.5 mL/min; Retention time: 15.955 min(minor), 20.905 min(major).

tert-butyl (3aR,3bR,9bR)-7-(allyloxy)-2,3-dihydro-1H-cyclopenta[1,3]cyclopropa[1,2-a]naphthalene-3a(3bH)-carboxylate (2j)

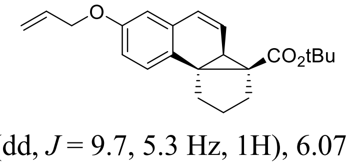

Colorless oil, 72% yield, 97% ee. ^{1}H NMR (300 MHz, Chloroform-*d*) δ 7.48 (d, *J* = 8.5 Hz, 1H), 6.83 (dd, *J* = 8.5, 2.8 Hz, 1H), 6.70 (d, *J* = 2.7 Hz, 1H), 6.42 (d, *J* = 9.7 Hz, 1H), 6.19 (dd, *J* = 9.7, 5.3 Hz, 1H), 6.07 (ddt, *J* = 15.8, 10.5, 5.3 Hz, 1H), 5.47 – 5.37 (m, 1H), 5.29 (dd, *J* = 10.4, 1.6 Hz, 1H), 4.55 (dt, *J* = 5.4, 1.5 Hz, 2H), 2.75 (td, *J* = 12.0, 8.4 Hz, 1H), 2.51 – 2.24 (m, 3H), 1.97 (dd, *J* = 12.6, 7.7 Hz, 1H), 1.79 (dt, *J* = 13.5, 8.1 Hz, 1H), 1.38 – 1.28 (m, 1H), 1.11 (s, 9H). ^{13}C NMR (75 MHz, $CDCl_3$) δ 169.20, 156.77, 133.74, 133.46, 127.52, 126.78,

125.56, 124.89, 117.49, 113.56, 113.33, 79.93, 68.87, 40.20, 31.41, 31.34, 29.21, 28.51, 27.71, 18.97. HRMS (ESI+): Calculated for $[C_{22}H_{26}O_3Na]^+$ ($[M+Na]^+$): 361.1774, Found: 361.1763. HPLC conditions (Agilent 1260): Chiralpak IB (Particle Size: 5 μm, Dimensions: 4.6 mm Φ × 250 mmL); detected at 254 nm; temperature 25°C; *i*-propanol/hexane=5/95; flow=0.5 mL/min; Retention time: 8.102 min(minor), 10.363 min(major).

tert-butyl (3aR,3bR,9bR)-7-((3-phenylprop-2-yn-1-yl)oxy)-2,3-dihydro-1H-cyclopenta[1,3]cyclopropa[1,2-a]naphthalene-3a(3bH)-carboxylate (2k)

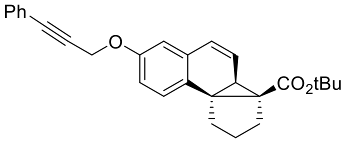

Colorless oil, 67% yield, 99% ee. ^{1}H NMR (300 MHz, Chloroform-*d*) δ 7.52 (d, *J* = 8.5 Hz, 1H), 7.47 – 7.42 (m, 2H), 7.36 – 7.30 (m, 3H), 6.94 (dd, *J* = 8.5, 2.8 Hz, 1H), 6.82 (d, *J* = 2.8 Hz, 1H), 6.45 (d, *J* = 9.6 Hz, 1H), 6.21 (dd, *J* = 9.7, 5.3 Hz, 1H), 4.93 (s, 2H), 2.76 (td, *J* = 12.0, 8.3 Hz, 1H), 2.47 – 2.24 (m, 3H), 1.98 (dd, *J* = 12.6, 7.7 Hz, 1H), 1.80 (dt, *J* = 13.3, 8.1 Hz, 1H), 1.30 (d, *J* = 12.5 Hz, 1H), 1.08 (s, 9H). ^{13}C NMR (75 MHz, $CDCl_3$) δ 169.21, 133.80, 131.81, 130.77, 128.62, 128.24, 127.44, 126.83, 126.22, 125.06, 122.26, 113.71, 113.55, 87.05, 84.07, 79.98, 56.74, 40.17, 31.41, 31.34, 29.22, 28.60, 27.66, 18.98. HRMS (ESI+): Calculated for $[C_{28}H_{28}O_3Na]^+$ ($[M+Na]^+$): 435.1931, Found: 435.1922. HPLC conditions (Agilent 1260): Chiralpak IB (Particle Size: 5 μm, Dimensions: 4.6 mm Φ × 250 mmL); detected at 254 nm; temperature 25 °C; *i*-propanol/hexane=5/95; flow=0.5 mL/min; Retention time: 11.026 min(minor), 13.471 min(major).

tert-butyl (3aR,3bR,9bR)-7-morpholino-2,3-dihydro-1H-cyclopenta[1,3]cyclopropa[1,2-a]naphthalene-3a(3bH)-carboxylate (2l)

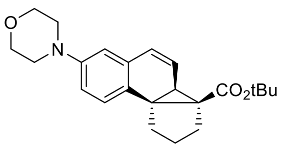

Yellow oil, 63% yield, 85% ee. ^{1}H NMR (300 MHz, Chloroform-*d*) δ 7.48 (d, *J* = 8.5 Hz, 1H), 6.85 (dd, *J* = 8.4, 2.7 Hz, 1H), 6.70 (d, *J* = 2.7 Hz, 1H), 6.42 (d, *J* = 9.6 Hz, 1H), 6.17 (dd, *J* = 9.7, 5.3 Hz, 1H), 3.97 – 3.82 (m, 4H), 3.15 (dd, *J* = 5.8, 3.8 Hz, 4H), 2.75 (td, *J* = 12.0, 8.3 Hz, 1H), 2.47 – 2.22 (m, 3H), 1.96 (dd, *J* = 12.5, 7.7 Hz, 1H), 1.80 (dd, *J* = 13.5, 8.0 Hz, 1H), 1.40 – 1.25 (m, 1H), 1.12 (s, 9H). ^{13}C NMR (75 MHz, $CDCl_3$) δ 169.22, 152.49, 149.56, 142.92, 133.36, 127.88, 126.52, 124.56, 115.01, 79.83, 66.93, 49.89, 40.24, 31.41, 31.24, 29.28, 28.66, 27.73, 18.98. HRMS (ESI+): Calculated for $[C_{23}H_{30}NO_3]^+$ ($[M+Na]^+$): 368.2220, Found: 368.2227.. HPLC conditions (Agilent 1260): Chiralpak OJ (Particle Size: 5 μm, Dimensions: 4.6 mm Φ × 250 mmL); detected at 254 nm; temperature 25 °C; *i*-propanol/hexane=10/90; flow=0.5 mL/min; Retention time: 12.448 min(major), 13.147 min(minor).

tert-butyl (3aR,3bR,9bR)-7-(((trifluoromethyl)sulfonyl)oxy)-2,3-dihydro-1H-cyclopenta[1,3]cyclopropa[1,2-a]naphthalene-3a(3bH)-carboxylate (2m)

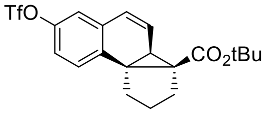

Yellow oil, 68% yield, 99% ee. ^{1}H NMR (300 MHz, Chloroform-d) δ 7.64 (d, J = 8.6 Hz, 1H), 7.14 (dd, J = 8.6, 2.7 Hz, 1H), 7.03 (d, J = 2.7 Hz, 1H), 6.44 (d, J = 9.7 Hz, 1H), 6.38 – 6.27 (m, 1H), 2.77 (td, J = 12.0, 8.3 Hz, 1H), 2.47 – 2.30 (m, 3H), 2.01 (dd, J = 12.5, 7.7 Hz, 1H), 1.93 – 1.77 (m, 1H), 1.43 – 1.31 (m, 1H), 1.09 (s, 9H). ^{13}C NMR (75 MHz, $CDCl_3$) δ 168.50, 147.65, 134.71, 133.58, 127.51, 127.01, 126.01, 119.35, 119.12, 80.55, 39.97, 31.33, 29.71,

29.50, 29.23, 27.58, 18.91. HRMS (ESI+): Calculated for $[C_{20}H_{21}F_3O_5SNa]^+$ ($[M+Na]^+$): 453.0954, Found: 453.0961. HPLC conditions (Agilent 1260): Chiralpak AS-H (Particle Size: 5 μm, Dimensions: 4.6 mm Φ × 250 mmL); detected at 210 nm; temperature 25 °C; *i*-propanol/hexane=1/99; flow=0.5 mL/min; Retention time: 7.773 min(major), 8.348 min(minor).

tert-butyl (3aR,3bR,9bR)-7-vinyl-2,3-dihydro-1H-cyclopenta[1,3]cyclopropa[1,2-a]naphthalene-3a(3bH)-carboxylate (2n)

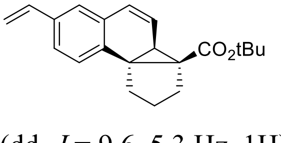

Colorless oil, 75% yield, 98% ee; ^{1}H NMR (300 MHz, Chloroform-*d*) δ 7.55 (d, *J* = 8.0 Hz, 1H), 7.35 – 7.29 (m, 1H), 7.17 (d, *J* = 1.9 Hz, 1H), 6.71 (dd, *J* = 17.6, 10.9 Hz, 1H), 6.48 (d, *J* = 9.6 Hz, 1H), 6.19 (dd, *J* = 9.6, 5.3 Hz, 1H), 5.73 (dd, *J* = 17.6, 1.0 Hz, 1H), 5.22 (dd, *J* = 10.9, 1.0 Hz, 1H), 2.80 (td, *J* = 12.0, 8.2 Hz, 1H), 2.47 – 2.28 (m, 3H), 1.98 (dd, *J* = 12.6, 7.7 Hz, 1H), 1.80 (dd, *J* = 14.5, 6.9 Hz, 1H), 1.36 – 1.28 (m, 1H), 1.10 (s, 9H). ^{13}C NMR (75 MHz, $CDCl_3$) δ 168.94, 136.66, 135.21, 132.88, 132.70, 127.48, 125.96, 125.20, 124.74, 124.31, 112.99, 80.06, 40.36, 31.41, 31.23, 29.56, 29.35, 27.66, 19.00. HRMS (ESI+): Calculated for $[C_{21}H_{24}O_2Na]^+$ ($[M+Na]^+$): 331.1669, Found: 331.1672 HPLC conditions (Agilent 1260): Chiralpak OJ (Particle Size: 5 μm, Dimensions: 4.6 mm Φ × 250 mmL); detected at 254 nm; temperature 25 °C; *i*-propanol/hexane=5/95; flow=0.5 mL/min; Retention time: 28.077 min(minor), 34.785 min(major).

tert-butyl (3aR,3bR,9bR)-7-allyl-2,3-dihydro-1H-cyclopenta[1,3]cyclopropa[1,2-a]naphthalene-3a(3bH)-carboxylate (2o)

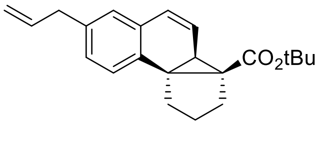

Colorless oil, 73% yield, 97% ee. ^{1}H NMR (300 MHz, Chloroform-d) δ 7.49 (d, J = 7.9 Hz, 1H), 7.06 (dd, J = 7.9, 1.9 Hz, 1H), 6.94 (d, J = 1.9 Hz, 1H), 6.42 (d, J = 9.6 Hz, 1H), 6.14 (dd, J = 9.6, 5.2 Hz, 1H), 5.94 (ddt, J = 16.8, 10.0, 6.7 Hz, 1H), 5.12 – 5.00 (m, 2H), 3.38 – 3.33 (m, 2H), 2.83 – 2.69 (m, 1H), 2.45 – 2.23 (m, 3H), 1.95 (dd, J = 12.6, 7.7 Hz, 1H), 1.78 (dt, J = 13.2, 8.1 Hz, 1H), 1.38 – 1.27 (m, 1H), 1.07 (s, 9H). ^{13}C NMR (75 MHz, $CDCl_3$) δ 169.11, 137.66, 137.49, 132.66, 130.83, 127.60, 127.56, 127.25, 125.85, 124.03, 115.51, 79.94, 40.28, 39.79, 31.43, 31.25, 29.43, 28.71, 27.65, 19.00. HRMS (ESI+): Calculated for $[C_{22}H_{26}O_2Na]^+$ ($[M+Na]^+$): 345.1825, Found: 345.1820. HPLC conditions (Agilent 1260): Chiralpak OJ (Particle Size: 5 μm, Dimensions: 4.6 mm Φ × 250 mmL); detected at 254 nm; temperature 25 °C; *i*-propanol/hexane=5/95; flow=0.2 mL/min; Retention time: 22.464 min(minor), 25.104 min(major).

tert-butyl (3aR,3bR,9bR)-7-(4-(methoxycarbonyl)phenyl)-2,3-dihydro-1H-cyclopenta[1,3]cyclopropa[1,2-a]naphthalene-3a(3bH)-carboxylate (2p)

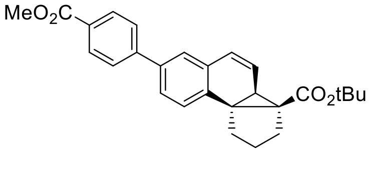

White solid, 61% yield, 99% ee. ^{1}H NMR (300 MHz, Chloroform-*d*) δ 8.11 (d, *J* = 8.4 Hz, 2H), 7.74 – 7.66 (m, 3H), 7.53 (dd, *J* = 8.1, 2.0 Hz, 1H), 7.40 (d, *J* = 2.0 Hz, 1H), 6.56 (d, *J* = 9.7 Hz, 1H), 6.25 (dd, *J* = 9.6, 5.3 Hz, 1H), 3.96 (s, 3H), 2.94 – 2.76 (m, 1H), 2.49 – 2.34 (m, 3H), 2.03 (dd, *J* = 13.2, 8.4 Hz, 1H), 1.93 – 1.79 (m, 1H), 1.39 – 1.28 (m, 1H), 1.11 (s, 9H). ^{13}C NMR (75 MHz,

CDCl$_3$) δ 168.80, 167.04, 145.47, 137.40, 133.43, 133.18, 130.07, 128.63, 127.34, 126.78, 126.45, 126.03, 125.67, 124.82, 80.13, 52.12, 40.23, 31.42, 31.25, 29.67, 29.50, 27.67, 19.02. HRMS (ESI+): Calculated for $[C_{27}H_{28}O_4Na]^+$ ($[M+Na]^+$): 439.1880, Found: 439.1864. HPLC conditions (Agilent 1260): Chiralpak IC (Particle Size: 5 μm, Dimensions: 4.6 mm Φ × 250 mmL); detected at 254 nm; temperature 25 °C; *i*-propanol/hexane=8/92; flow=0.5 mL/min. Retention time: 12.421 min(major), 14.568 min(minor).

tert-butyl (3aR,3bR,9bR)-7-(naphthalen-2-yl)-2,3-dihydro-1H-cyclopenta[1,3]cyclopropa[1,2-a]naphthalene-3a(3bH)-carboxylate (2q)

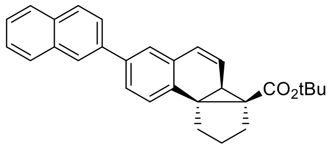

White solid, 63% yield, 99% ee. ^{1}H NMR (300 MHz, Chloroform-*d*) δ 8.06 (s, 1H), 7.95 – 7.86 (m, 3H), 7.81 – 7.69 (m, 2H), 7.63 (dd, *J* = 8.1, 2.0 Hz, 1H), 7.57 – 7.46 (m, 3H), 6.59 (d, *J* = 9.7 Hz, 1H), 6.25 (dd, *J* = 9.6, 5.3 Hz, 1H), 2.96 – 2.78 (m, 1H), 2.53 – 2.30 (m, 3H), 2.05 (dd, *J* = 12.6, 7.6 Hz, 1H), 1.85 (dt, *J* = 15.4, 7.9 Hz, 1H), 1.37 – 1.31 (m, 1H), 1.12 (s, 9H). ^{13}C NMR (75 MHz, CDCl$_3$) δ 169.03, 138.55, 138.35, 134.23, 133.69, 133.10, 132.50, 128.34, 128.13, 127.63, 127.57, 126.37, 126.24, 125.86, 125.80, 125.50, 125.39, 124.58, 116.58, 80.14, 40.30, 31.45, 31.27, 29.62, 29.39, 27.67, 19.06. HRMS (ESI+): Calculated for $[C_{29}H_{28}O_2Na]^+$ ($[M+Na]^+$): 431.1982, Found: 431.1979. HPLC conditions (Agilent 1260): Chiralpak AY-3 (Particle Size: 5 μm, Dimensions: 4.6 mm Φ × 250 mmL); detected at 254 nm; temperature 25 °C; *i*-propanol/hexane=10/90; flow=0.5 mL/min. Retention time: 10.468 min(major), 13.691 min(minor).

tert-butyl 7-phenyl-2,3-dihydro-1H-cyclopenta[1,3]cyclopropa[1,2-a]naphthalene-3a(3bH)-carboxylate (2r)

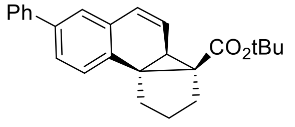

White solid, 71% yield, 92% ee; ^{1}H NMR (300 MHz, Chloroform-*d*) δ 7.69 – 7.60 (m, 3H), 7.52 – 7.42 (m, 3H), 7.39 – 7.34 (m, 2H), 6.55 (d, *J* = 9.6 Hz, 1H), 6.24 (dd, *J* = 9.7, 5.2 Hz, 1H), 2.92 – 2.76 (m, 1H), 2.50 – 2.33 (m, 3H), 2.03 (dd, *J* = 12.6, 7.7 Hz, 1H), 1.84 (dt, *J* = 15.2, 8.0 Hz, 1H), 1.42 – 1.31 (m, 1H), 1.10 (s, 9H). ^{13}C NMR (75 MHz, CDCl$_3$) δ 169.06, 141.08, 138.71, 132.98, 132.36, 128.71, 127.54, 127.05, 126.96, 126.26, 126.03, 125.63, 124.53, 80.11, 77.45, 77.03, 76.60, 40.29, 31.45, 31.25, 29.57, 29.35, 27.64, 19.07. HRMS (ESI+): Calculated for $[C_{25}H_{26}O_2Na]^+$ ($[M+Na]^+$): 381.1825, Found: 381.1830. HPLC conditions (Agilent 1260): Chiralpak OJ (Particle Size: 5 μm, Dimensions: 4.6 mm Φ × 250 mmL); detected at 254 nm; temperature 25 °C; *i*-propanol/hexane=10/90; flow=0.5 mL/min; Retention time: 8.333 min(major), 9.150 min(minor).

tert-butyl (3aR,3bR,9bR)-7-(thiophen-2-yl)-2,3-dihydro-1H-cyclopenta[1,3]cyclopropa[1,2-a]naphthalene-3a(3bH)-carboxylate (2s)

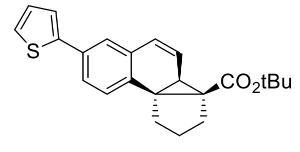

Colorless oil, 68% yield, 95% ee. ^{1}H NMR (300 MHz, Chloroform-*d*) δ 7.60 (d, *J* = 8.1 Hz, 1H), 7.51 (dd, *J* = 8.1, 2.0 Hz, 1H), 7.38 (d, *J* = 2.0 Hz, 1H), 7.33 – 7.24 (m, 2H), 7.10 (dd, *J* = 5.1, 3.6 Hz, 1H), 6.52 (d, *J* = 9.7 Hz, 1H), 6.23 (dd, *J* = 9.7, 5.2 Hz, 1H), 2.81 (td, *J* = 12.0, 8.4 Hz, 1H), 2.52 – 2.30 (m, 3H), 2.01 (dd, *J* = 12.5, 7.7 Hz, 1H), 1.83 (dt, *J* = 15.5, 7.8 Hz, 1H), 1.40 – 1.29 (m, 1H), 1.12 (s, 9H). ^{13}C NMR (75 MHz, CDCl$_3$) δ 168.92, 144.48,

133.08, 132.63, 131.99, 127.93, 127.31, 126.33, 124.77, 124.47, 124.36, 122.70, 80.16, 40.32, 31.41, 31.22, 29.60, 29.47, 27.69, 19.01. HRMS (ESI+): Calculated for $[C_{23}H_{24}O_2SNa]^+$ ($[M+Na]^+$): 387.1389, Found: 387.1384. HPLC conditions (Agilent 1260): Chiralpak MX (Particle Size: 5 µm, Dimensions: 4.6 mm Φ × 250 mmL); detected at 254 nm; temperature 25 °C; *i*-propanol/hexane=1/99; flow=1 mL/min; Retention time: 4.897 min(major), 5.413 min(minor).

tert-butyl (3aR,3bR,9bR)-7-cyano-2,3-dihydro-1H-cyclopenta[1,3]cyclopropa[1,2-a]naphthalene-3a(3bH)-carboxylate (2t)

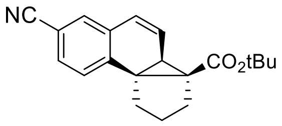

Colorless oil, 18% yield, 84% ee. ^{1}H NMR (300 MHz, Chloroform-*d*) δ 7.68 (d, *J* = 7.9 Hz, 1H), 7.52 (d, *J* = 8.3 Hz, 1H), 7.41 (s, 1H), 6.46 (d, *J* = 9.7 Hz, 1H), 6.32 (d, *J* = 5.2 Hz, 1H), 2.84 – 2.74 (m, 1H), 2.39 (dd, *J* = 8.9, 4.8 Hz, 3H), 2.10 – 1.75 (m, 2H), 1.44 – 1.32 (m, 2H), 1.11 (s, 8H). ^{13}C NMR (75 MHz, $CDCl_3$) δ 174.32, 143.06, 139.31, 135.87, 134.71, 130.30, 129.40, 127.05, 122.54, 81.05, 40.67, 38.25, 33.37, 31.12, 29.70, 27.66, 15.94. HRMS (ESI+): Calculated for $[C_{20}H_{21}NO_2Na]^+$ ($[M+Na]^+$): 330.1465, Found: 330.1461. HPLC conditions (Agilent 1260): Chiralpak OJ (Particle Size: 5 µm, Dimensions: 4.6 mm Φ × 250 mmL); detected at 254 nm; temperature 25 °C; *i*-propanol/hexane=10/90; flow=0.5 mL/min; Retention time: 8.791 min(minor), 10.939 min(major).

tert-butyl (3aR,3bR,9bR)-9-(methoxymethyl)-2,3-dihydro-1H-cyclopenta[1,3]cyclopropa[1,2-a]naphthalene-3a(3bH)-carboxylate (2u)

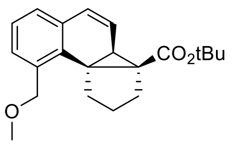

Colorless oil, 53% yield, 91% ee. ^{1}H NMR (400 MHz, Chloroform-*d*) δ 7.24 (dd, *J* = 7.7, 1.5 Hz, 1H), 7.15 (t, *J* = 7.5 Hz, 1H), 6.99 (dd, *J* = 7.4, 1.5 Hz, 1H), 6.41 (d, *J* = 9.5 Hz, 1H), 6.08 (dd, *J* = 9.5, 5.2 Hz, 1H), 4.59 (d, *J* = 11.5 Hz, 1H), 4.42 (d, *J* = 11.5 Hz, 1H), 3.37 (s, 3H), 2.64 (ddd, *J* = 13.0, 10.8, 9.0 Hz, 1H), 2.57 – 2.49 (m, 1H), 2.19 (ddd, *J* = 13.1, 8.5, 1.6 Hz, 1H), 2.06 (d, *J* = 5.2 Hz, 1H), 1.94 (ddd, *J* = 13.2, 9.0, 1.8 Hz, 1H), 1.89 – 1.80 (m, 1H), 1.40 – 1.32 (m, 1H), 1.01 (s, 9H). ^{13}C NMR (151 MHz, $CDCl_3$) δ 169.94, 137.54, 134.31, 130.65, 129.29, 128.98, 127.41, 126.42, 123.61, 80.00, 77.23, 77.02, 76.81, 73.56, 58.29, 39.28, 34.13, 31.72, 30.97, 27.59, 26.63, 20.22. HRMS (ESI+): Calculated for $[C_{21}H_{26}O_3Na]^+$ ($[M+Na]^+$): 349.1774, Found: 349.1764. HPLC conditions (Agilent 1260): Chiralpak OJ (Particle Size: 5 µm, Dimensions: 4.6 mm Φ × 250 mmL); detected at 254 nm; temperature 25 °C; *i*-propanol/hexane=5/95; flow=0.5 mL/min; Retention time: 7.304 min(major), 8.154 min(minor).

tert-butyl (3aR,3bR,9bR)-2,3,5,6-tetrahydro-1H-cyclopenta[2,3]cyclopropa[1,2-e]acenaphthylene-3a(3bH)-carboxylate (2v)

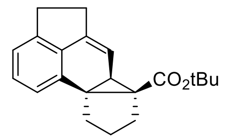

Colorless oil, 74% yield, 93% ee. ^{1}H NMR (300 MHz, Chloroform-*d*) δ 7.34 (d, *J* = 7.4 Hz, 1H), 7.22 (t, *J* = 7.5 Hz, 1H), 7.16 – 7.08 (m, 1H), 5.85 – 5.78 (m, 1H), 3.06 (t, *J* = 6.9 Hz, 2H), 2.93 – 2.69 (m, 3H), 2.44 – 2.29 (m, 3H), 1.97 (dd, *J* = 12.6, 7.7 Hz, 1H), 1.87 – 1.77 (m, 1H), 1.35 (s, 1H), 1.06 (s, 9H). ^{13}C NMR (75 MHz, $CDCl_3$) δ 171.39, 143.03, 141.86, 138.24, 131.41, 127.98, 122.62, 121.96, 112.01, 79.49, 39.95, 31.67, 31.25, 30.70, 30.31, 29.85, 27.90, 27.56, 19.31. HRMS (ESI+): Calculated for $[C_{21}H_{24}O_2Na]^+$ ($[M+Na]^+$): 331.1669, Found: 331.1661.

HPLC conditions (Agilent 1260): Chiralpak OJ (Particle Size: 5 μm, Dimensions: 4.6 mm Φ × 250 mmL); detected at 254 nm; temperature 25 °C; *i*-propanol/hexane=5/95; flow=0.5 mL/min; Retention time: 6.915 min(minor), 7.718 min(major).

tert-butyl (3aR,3bR,9bR)-2,3,4,5-tetrahydro-1H-cyclopenta[1,3]cyclopropa[1,2-a]naphthalene-3a(3bH)-carboxylate (4)

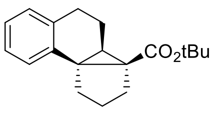

Colorless oil, 88% yield, 94% ee. ^{1}H NMR (300 MHz, Chloroform-*d*) δ 7.33 (d, *J* = 7.6 Hz, 1H), 7.15 (dt, *J* = 7.9, 4.3 Hz, 1H), 7.06 (d, *J* = 4.4 Hz, 2H), 2.78 (ddd, *J* = 16.3, 11.3, 5.9 Hz, 1H), 2.53 – 2.28 (m, 3H), 2.28 – 2.06 (m, 2H), 1.98 (ddd, *J* = 15.1, 12.9, 8.1 Hz, 2H), 1.83 – 1.70 (m, 2H), 1.47 – 1.33 (m, 1H), 1.09 (s, 9H). ^{13}C NMR (75 MHz, $CDCl_3$) δ 171.90, 138.82, 135.81, 127.78, 127.72, 125.99, 125.63, 79.84, 43.54, 37.42, 33.59, 31.64, 28.84, 27.79, 25.04, 20.11, 19.63. HRMS (ESI+): Calculated for $[C_{19}H_{24}O_2Na]^+$ ($[M+Na]^+$): 307.1669, Found: 307.1664. HPLC conditions (Agilent 1260): Chiralpak OJ (Particle Size: 5 μm, Dimensions: 4.6 mm Φ × 250 mmL); detected at 254 nm; temperature 25 °C; *i*-propanol/hexane=10/90; flow=0.1 mL/min; Retention time: 34.789 min(minor), 36.256 min(major).

tert-butyl (1S,2S)-3',4'-dihydro-2'H-spiro[cyclopentane-1,1'-naphthalene]-2-carboxylate (5)

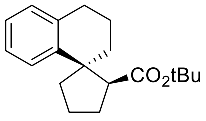

Colorless oil, 85% yield, 95% ee. ^{1}H NMR (300 MHz, Chloroform-*d*) δ 7.33 (d, *J* = 7.4 Hz, 1H), 7.20 – 7.07 (m, 3H), 3.26 (dd, *J* = 12.6, 6.2 Hz, 1H), 2.97 – 2.47 (m, 4H), 2.30 – 2.18 (m, 1H), 2.03 – 1.67 (m, 4H), 1.63 – 1.43 (m, 3H), 1.16 (s, 9H). ^{13}C NMR (75 MHz, $CDCl_3$) δ 175.23, 142.77, 140.09, 128.81, 126.92, 125.84, 125.32, 79.79, 53.96, 52.22, 41.97, 41.06, 36.28, 30.43, 27.71, 25.01, 23.43. HRMS (ESI+): Calculated for $[C_{19}H_{26}O_2Na]^+$ ($[M+Na]^+$): 309.1825, Found: 309.1819. HPLC conditions (Agilent 1260): Chiralpak OJ (Particle Size: 5 μm, Dimensions: 4.6 mm Φ × 250 mmL); detected at 254 nm; temperature 25 °C; *i*-propanol/hexane=10/90; flow=0.1 mL/min; Retention time: 35.097 min(minor), 36.643 min(major).

(3aR,4R,5R,9bR)-4-bromo-2,3,3b,4-tetrahydro-1H,5H-5,3a-(epoxymethano)cyclopenta[1,3]cyclopropa[1,2-a]naphthalen-11-one (6)

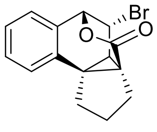

White solid, 93% yield, 95% ee. ^{1}H NMR (300 MHz, Chloroform-*d*) δ 7.55 – 7.44 (m, 2H), 7.40 – 7.33 (m, 2H), 5.30 (dd, *J* = 4.2, 2.0 Hz, 1H), 4.83 (ddd, *J* = 3.9, 2.6, 1.0 Hz, 1H), 2.69 – 2.41 (m, 3H), 2.25 (dd, *J* = 12.8, 7.6 Hz, 1H), 2.11 (dd, *J* = 13.5, 7.7 Hz, 1H), 1.94 (dt, *J* = 13.6, 8.1 Hz, 1H), 1.49 (dtt, *J* = 13.7, 12.1, 7.6 Hz, 1H). ^{13}C NMR (75 MHz, $CDCl_3$) δ 168.21, 134.13, 130.21, 129.07, 128.85, 126.88, 125.38, 76.92, 45.76, 41.89, 39.33, 28.67, 28.57, 28.49, 19.03. HRMS (ESI+): Calculated for $[C_{15}H_{13}BrO_2Na]^+$ ($[M+Na]^+$): 326.9991, Found: 326.9998. HPLC conditions (Agilent 1260): Chiralpak AD (Particle Size: 5 μm, Dimensions: 4.6 mm Φ × 250 mmL); detected at 254 nm; temperature 25°C; *i*-propanol/hexane=10/90; flow=0.5 mL/min; Retention time: 10.841 min(minor), 12.022 min(major).

(3aR,4R,5R,9bR)-4-hydroxy-2,3,3b,4-tetrahydro-1H,5H-5,3a-(epoxymethano)cyclopenta[1,3]cyclopropa[1,2-a]naphthalen-11-one (7)

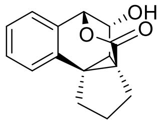

White solid, 94% yield, 96% ee. ^{1}H NMR (300 MHz, Chloroform-*d*) δ 7.57 – 7.43 (m, 2H), 7.40 – 7.30 (m, 2H), 5.14 (dd, *J* = 4.6, 2.0 Hz, 1H), 4.57 (ddd, *J* = 4.3, 2.9, 0.9 Hz, 1H), 2.64 – 2.34 (m, 3H), 2.22 (dd, *J* = 12.7, 7.5 Hz, 1H), 2.07 (dd, *J* = 13.5, 7.5 Hz, 1H), 1.92 (dt, *J* = 13.5, 8.0 Hz, 1H), 1.48 (dtt, *J* = 13.4, 12.1, 7.6 Hz, 1H). ^{13}C NMR (75 MHz, $CDCl_3$) δ 169.47, 136.60, 130.08, 128.39, 126.95, 125.32, 76.57, 61.38, 44.08, 40.43, 28.49, 28.48, 27.90, 19.06. HRMS (ESI+): Calculated for $[C_{15}H_{15}O_3]^+$ ($[M+H]^+$): 243.1016, Found: 243.1022. HPLC conditions (Agilent 1260): Chiralpak AD (Particle Size: 5 μm, Dimensions: 4.6 mm Φ × 250 mmL); detected at 254 nm; temperature 25°C; *i*-propanol/hexane=10/90; flow=0.5 mL/min; Retention time: 14.000 min(major), 15.419 min(minor).

((3aR,3bR,9bR)-2,3-dihydro-1H-cyclopenta[1,3]cyclopropa[1,2-a]naphthalen-3a(3bH)-yl)methanol (8)

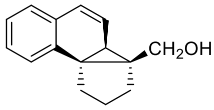

White solid, 90% yield, 91% ee. ^{1}H NMR (300 MHz, Chloroform-*d*) δ 7.58 – 7.50 (m, 1H), 7.26 – 7.10 (m, 3H), 6.48 (d, *J* = 9.6 Hz, 1H), 6.16 (dd, *J* = 9.6, 5.3 Hz, 1H), 3.35 – 3.04 (m, 2H), 2.71 (td, *J* = 11.9, 8.3 Hz, 1H), 2.29 – 2.19 (m, 2H), 2.11 (d, *J* = 5.3 Hz, 1H), 1.95 (dd, *J* = 12.5, 7.7 Hz, 1H), 1.80 (ddt, *J* = 17.2, 8.7, 4.0 Hz, 1H), 1.37 – 1.20 (m, 1H), 0.97 (s, 1H). ^{13}C NMR (75 MHz, $CDCl_3$) δ 133.23, 132.07, 127.78, 127.30, 127.04, 125.75, 125.54, 124.37, 60.40, 39.40, 31.19, 31.15, 29.06, 24.80, 19.21. HRMS (ESI+): Calculated for $[C_{15}H_{16}ONa]^+$ ($[M+Na]^+$): 235.1075, Found: 235.1093. HPLC conditions (Agilent 1260): Chiralpak AD (Particle Size: 5 μm, Dimensions: 4.6 mm Φ × 250 mmL); detected at 254 nm; temperature 25°C; *i*-propanol/hexane=10/90; flow=0.5 mL/min; Retention time: 18.648 min(minor), 20.193 min(major).

3. X-ray crystal structure of 6 and 7

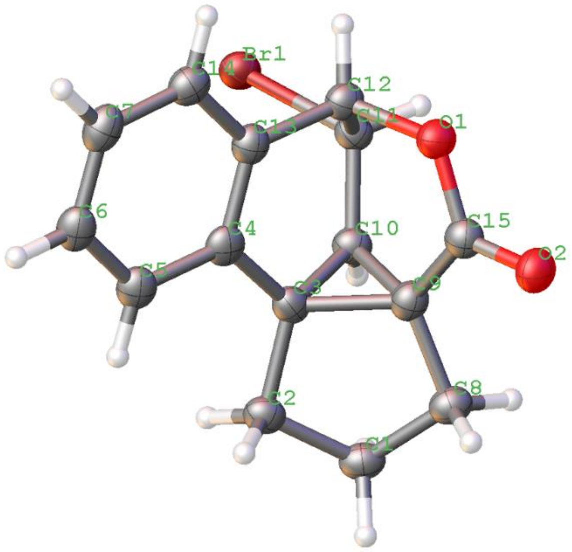

Fig. S1 X-ray crystal structure of **6** (CCDC 2191640 for **6** contain the supplementary crystallographic data for this paper. These data can be obtained free of charge from The Cambridge Crystallographic Data Centre via www.ccdc.cam.ac.uk/structures)

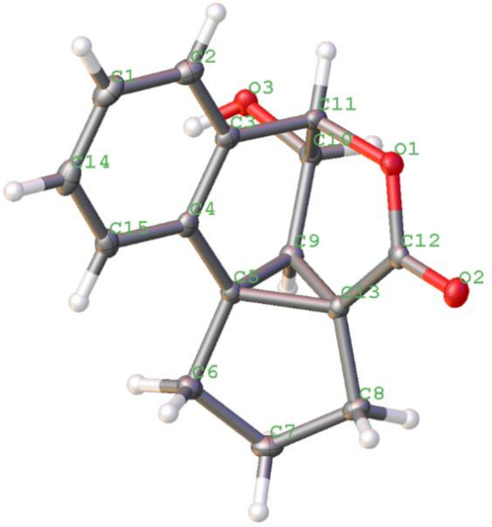

Fig. S2 X-ray crystal structure of **7** (CCDC 2191583 for **7** contains the supplementary crystallographic data for this paper. These data can be obtained free of charge from The Cambridge Crystallographic Data Centre via www.ccdc.cam.ac.uk/structutres).

Reference:

[1] J. Li, S. Qu, W. Zhao, *Angew. Chem. Int. Ed.* **2020**, *59*, 2360–2364.

[2] D. F. Taber, K. You, Y. Song, *J. Org. Chem.* **1995**, *60*, 1093-1094.

4. NMR Spectra of Compounds

NMR Spectrum for substrates 1

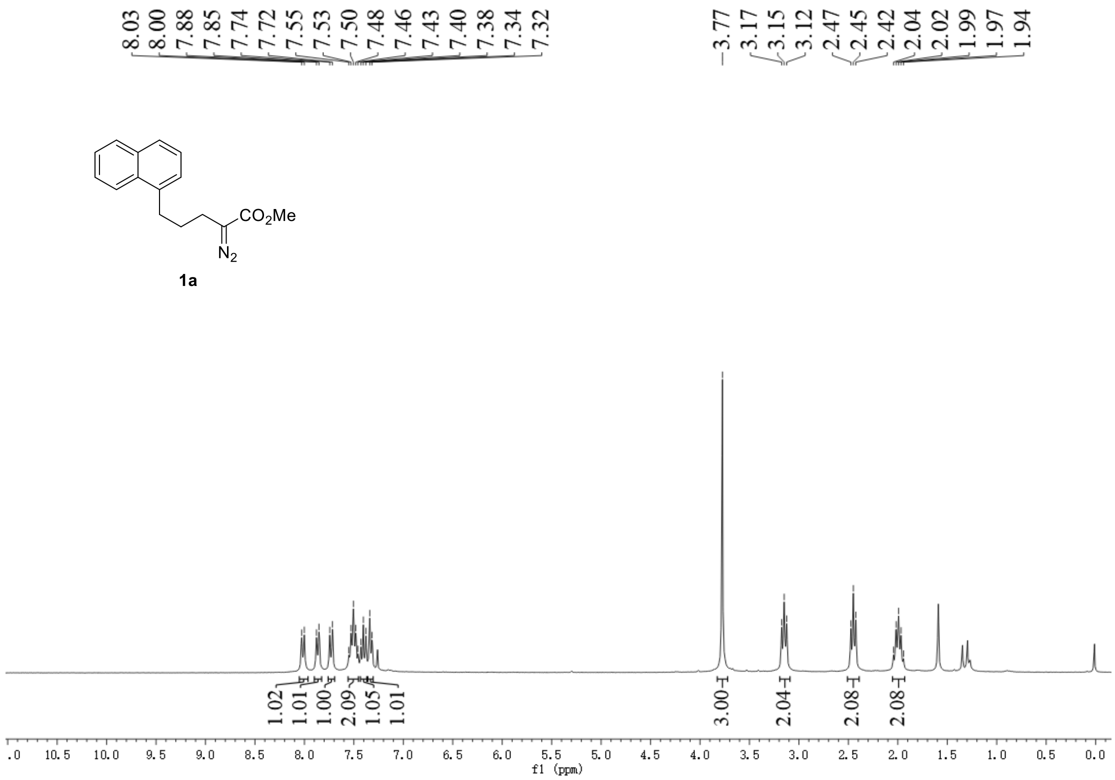

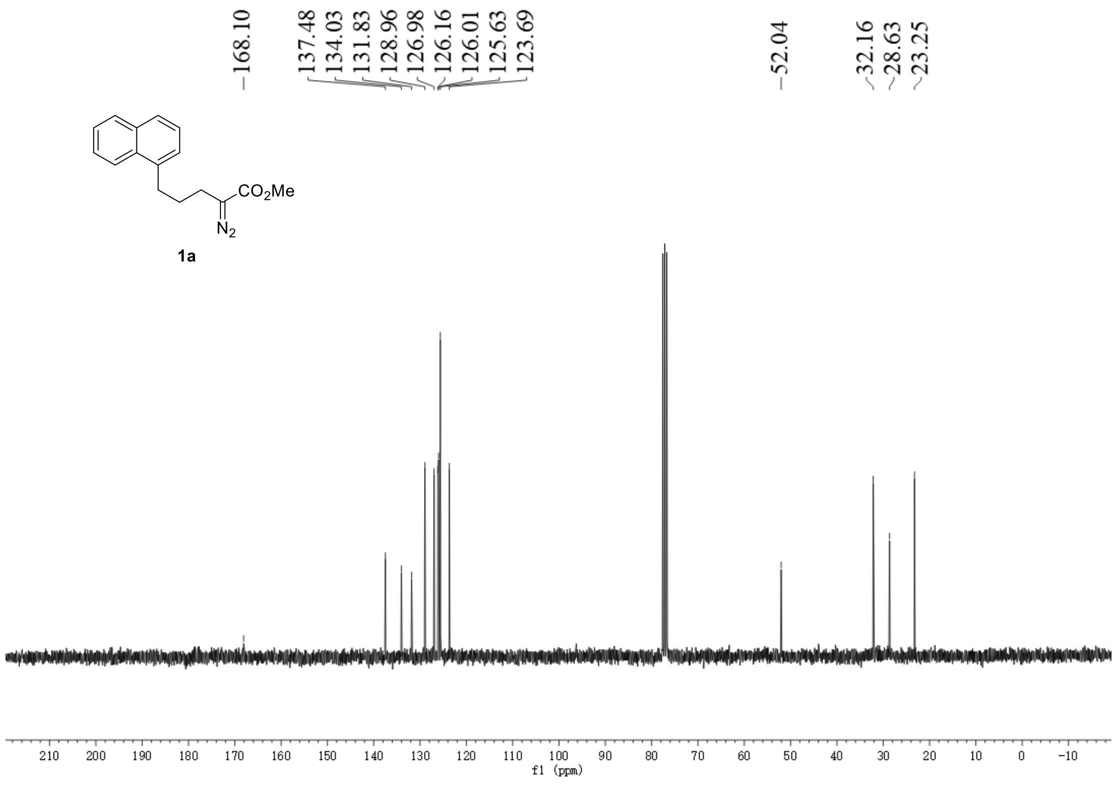

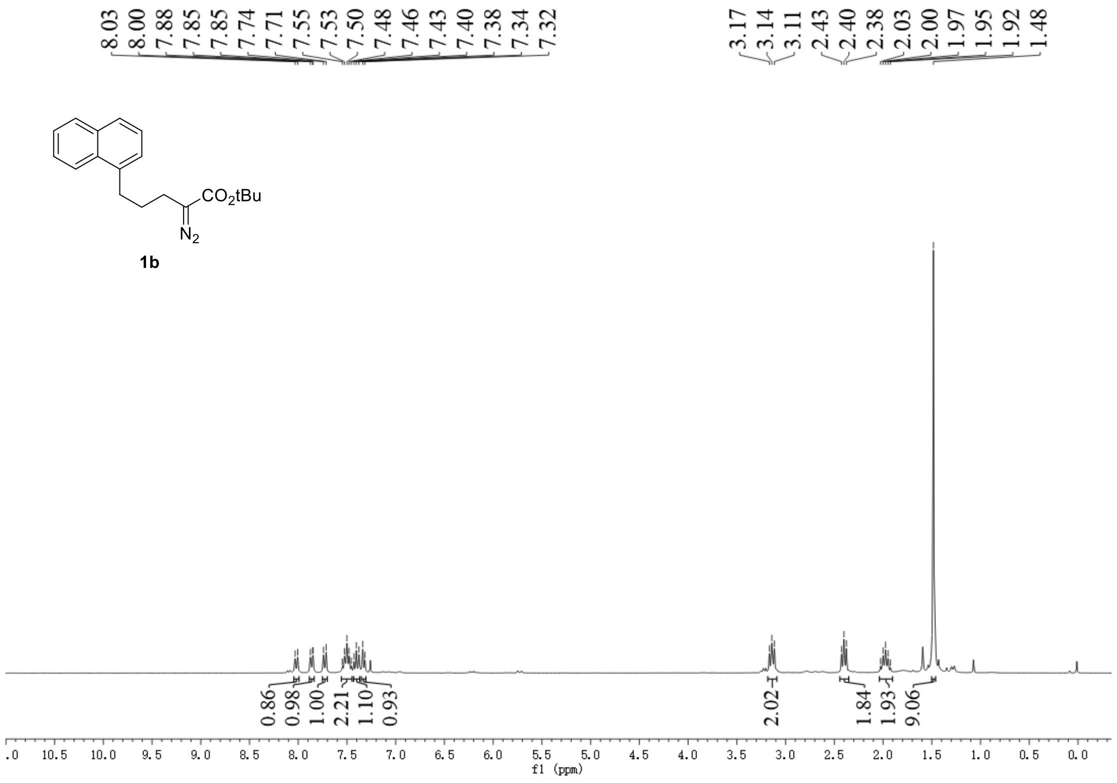
8.03
8.00
7.88
7.85
7.85
7.74
7.71
7.55
7.53
7.50
7.48
7.46
7.43
7.40
7.38
7.34
7.32
3.17
3.14
3.11
2.43
2.40
2.38
2.03
2.00
1.97
1.95
1.92
1.48
CO2tBu
N2
1b
0.86
0.98
1.00
2.21
1.10
0.93
2.02
1.84
1.93
9.06
f1 (ppm)

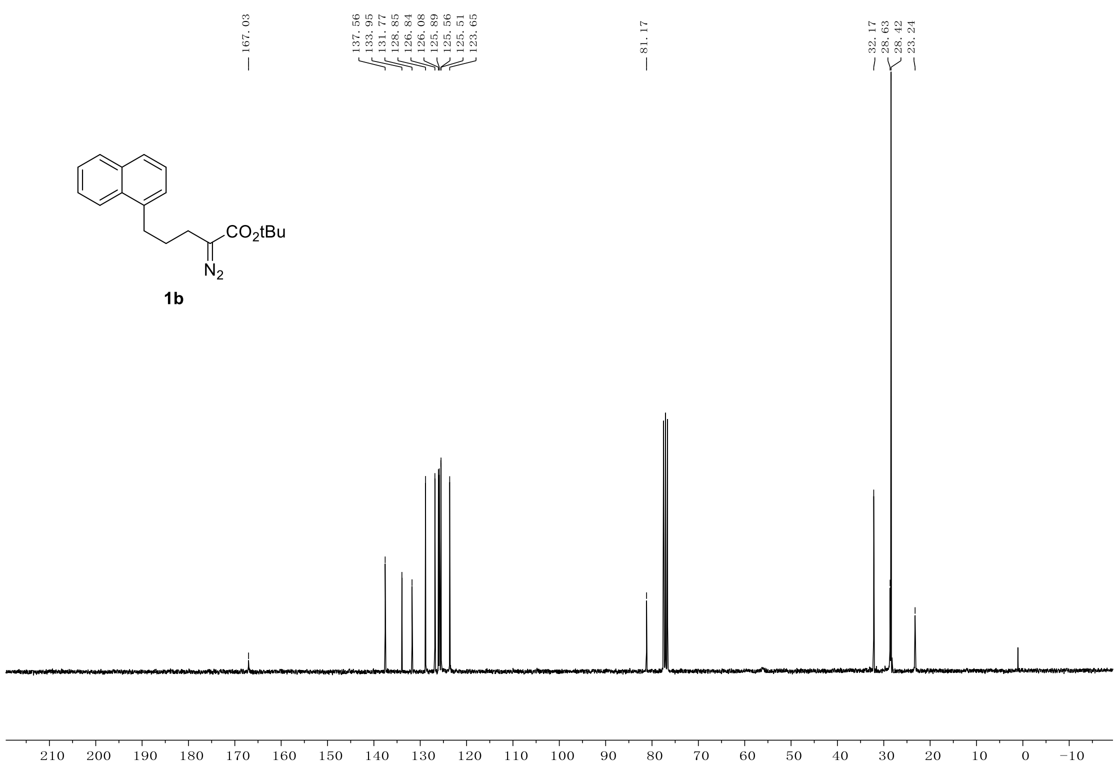
167.03
137.56
133.95
131.77
128.85
126.84
126.08
125.89
125.56
125.51
123.65
81.17
32.17
28.63
28.42
23.24
CO2tBu
N2
1b
f1 (ppm)

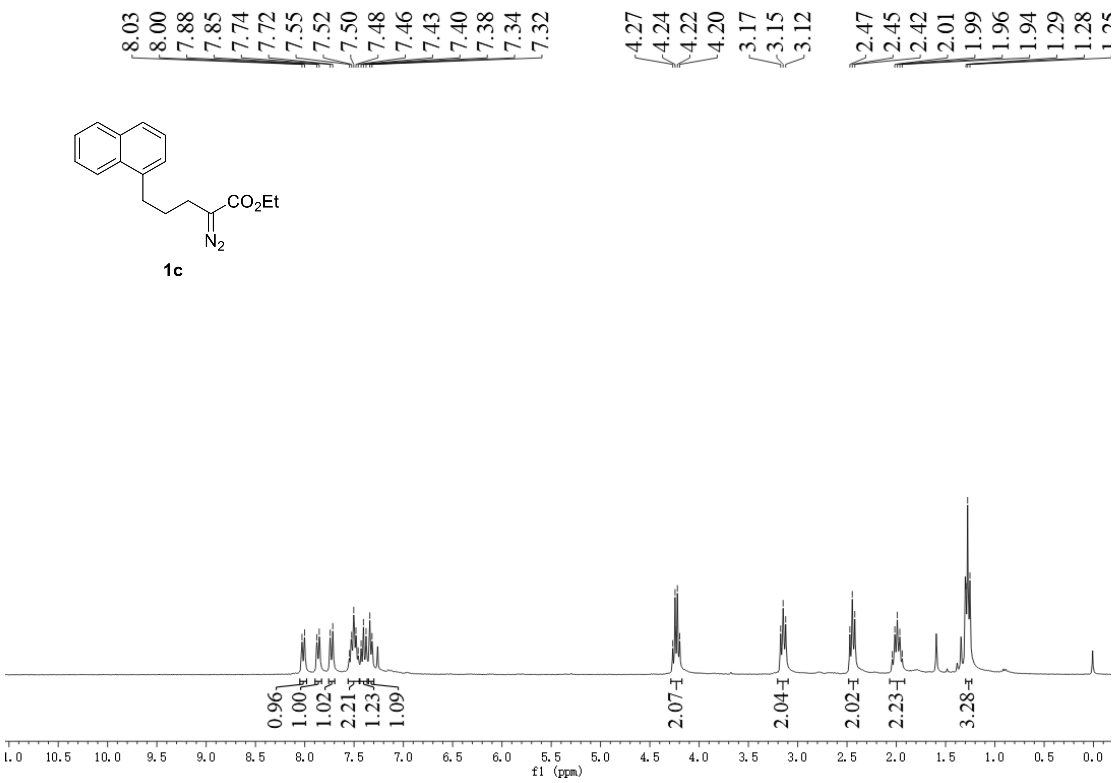
8.03 8.00 7.88 7.85 7.74 7.72 7.55 7.52 7.50 7.48 7.46 7.43 7.40 7.38 7.34 7.32
4.27 4.24 4.22 4.20 3.17 3.15 3.12
2.47 2.45 2.42 2.01 1.99 1.96 1.94 1.29 1.28 [illegible]
CO_2Et
N_2
1c
0.96 1.00 1.02 2.21 1.23 1.09
2.07 2.04 2.02 2.23 3.28
f1 (ppm)

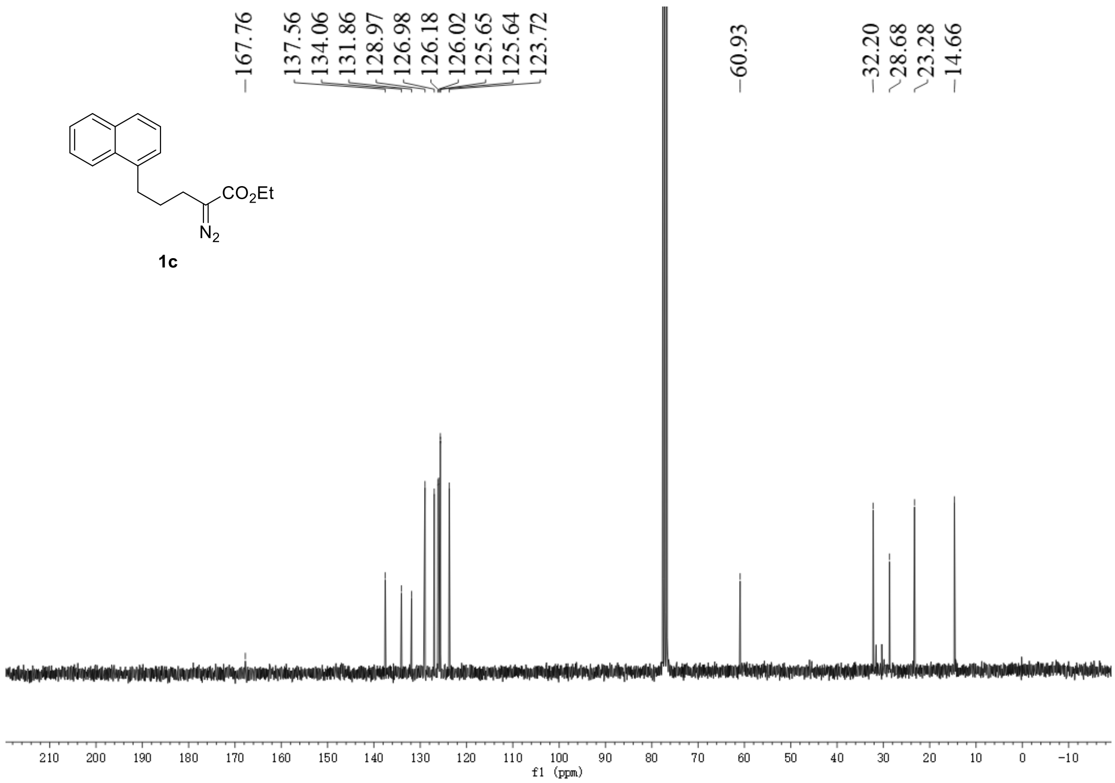
167.76
137.56 134.06 131.86 128.97 126.98 126.18 126.02 125.65 125.64 123.72
60.93
32.20 28.68 23.28 14.66
CO_2Et
N_2
1c
f1 (ppm)

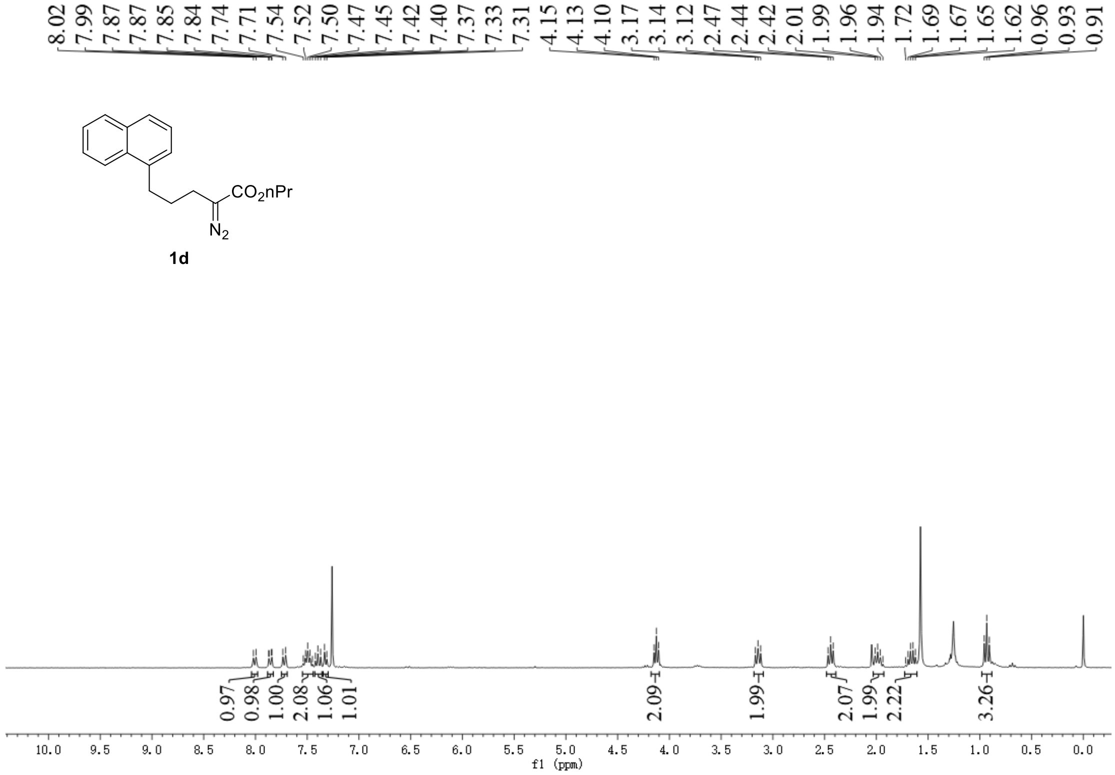
8.02 7.99 7.87 7.87 7.85 7.84 7.74 7.71 7.54 7.52 7.50 7.47 7.45 7.42 7.40 7.37 7.33 7.31 4.15 4.13 4.10 3.17 3.14 3.12 2.47 2.44 2.42 2.01 1.99 1.96 1.94 1.72 1.69 1.67 1.65 1.62 0.96 0.93 0.91
CO2nPr
N2
1d
0.97 0.98 1.00 2.08 1.06 1.01 2.09 1.99 2.07 1.99 2.22 3.26
10.0 9.5 9.0 8.5 8.0 7.5 7.0 6.5 6.0 5.5 5.0 4.5 4.0 3.5 3.0 2.5 2.0 1.5 1.0 0.5 0.0
f1 (ppm)

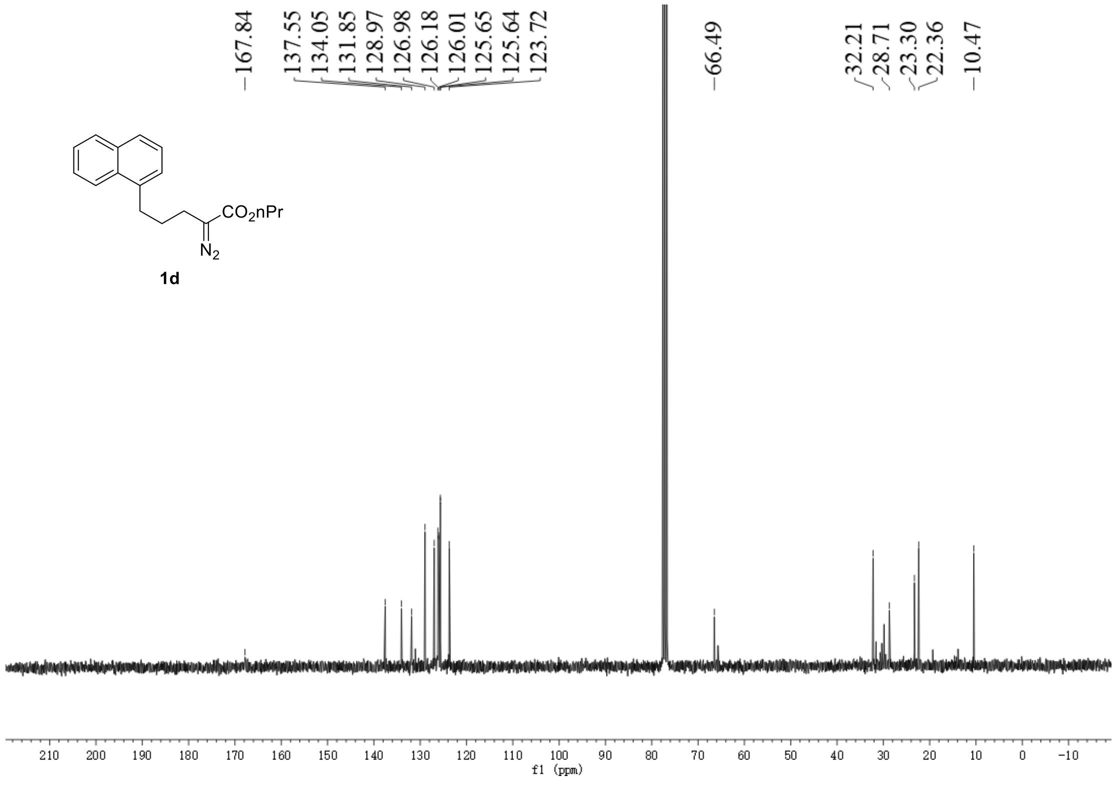
167.84
137.55 134.05 131.85 128.97 126.98 126.18 126.01 125.65 125.64 123.72
66.49
32.21 28.71 23.30 22.36
10.47
CO2nPr
N2
1d
210 200 190 180 170 160 150 140 130 120 110 100 90 80 70 60 50 40 30 20 10 0 -10
f1 (ppm)

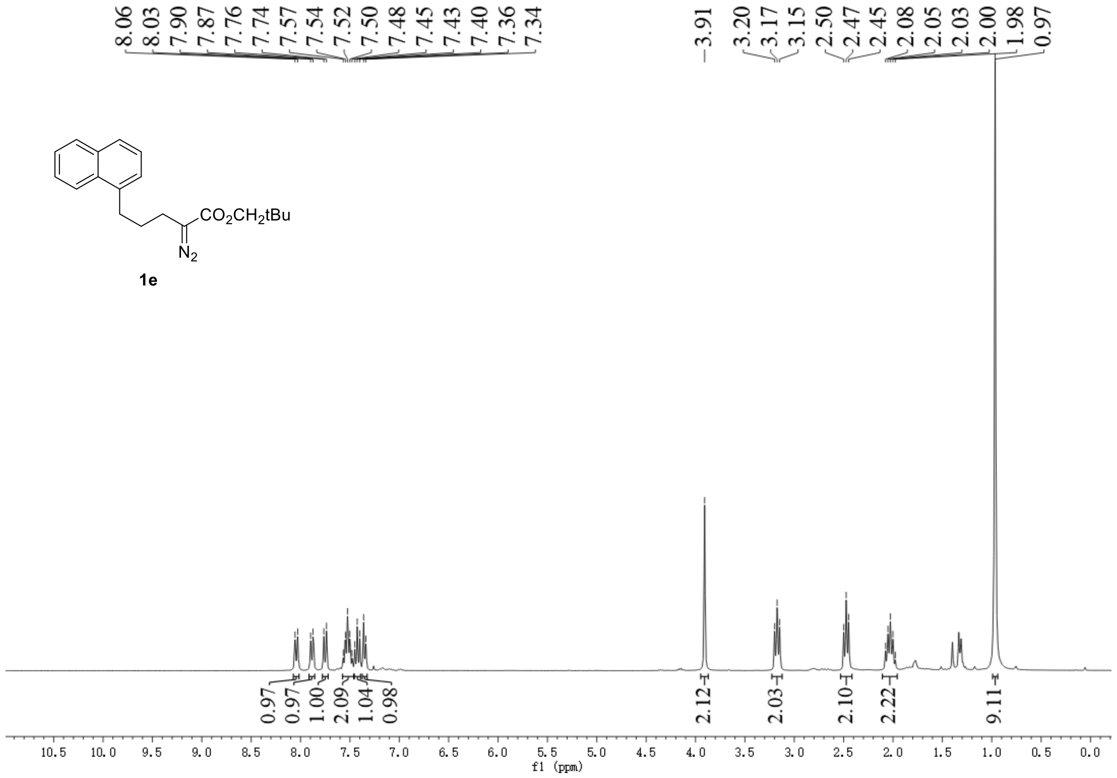
8.06 8.03 7.90 7.87 7.76 7.74 7.57 7.54 7.52 7.50 7.48 7.45 7.43 7.40 7.36 7.34
3.91
3.20 3.17 3.15 2.50 2.47 2.45 2.08 2.05 2.03 2.00 1.98 0.97
CO2CH2tBu
N2
1e
0.97 0.97 1.00 2.09 1.04 0.98
2.12 2.03 2.10 2.22 9.11
10.5 10.0 9.5 9.0 8.5 8.0 7.5 7.0 6.5 6.0 5.5 5.0 4.5 4.0 3.5 3.0 2.5 2.0 1.5 1.0 0.5 0.0
f1 (ppm)

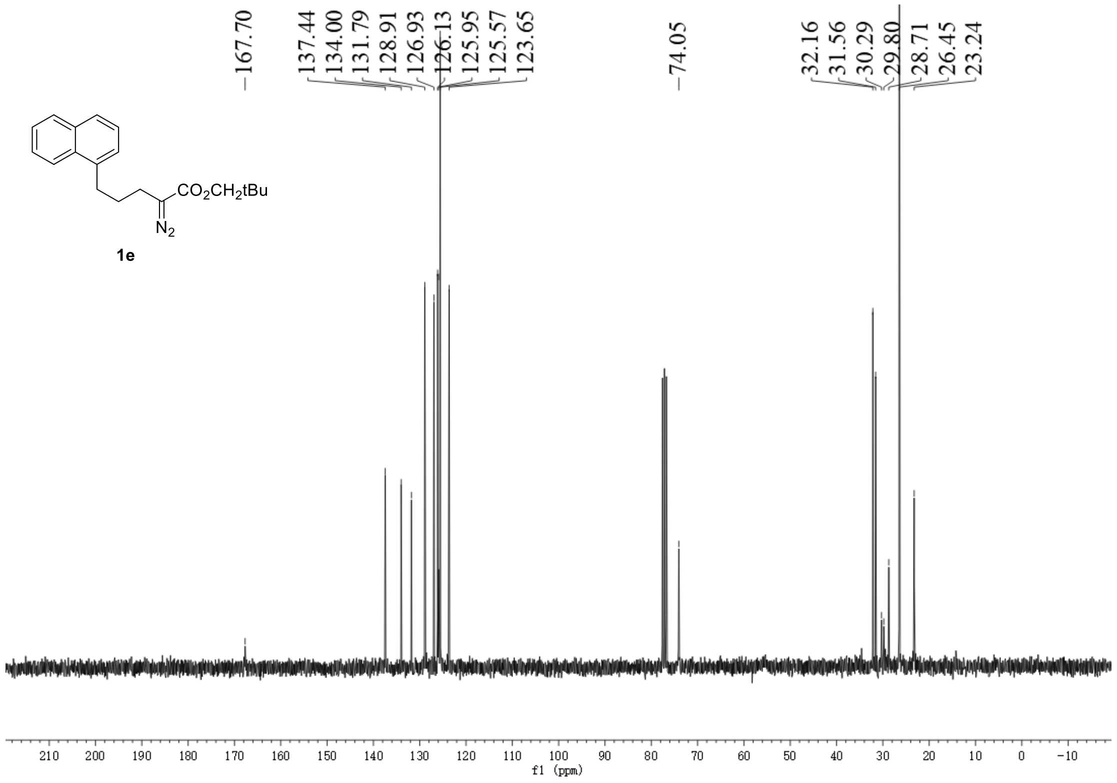
167.70
137.44 134.00 131.79 128.91 126.93 126.13 125.95 125.57 123.65
74.05
32.16 31.56 30.29 29.80 28.71 26.45 23.24
CO2CH2tBu
N2
1e
210 200 190 180 170 160 150 140 130 120 110 100 90 80 70 60 50 40 30 20 10 0 -10
f1 (ppm)

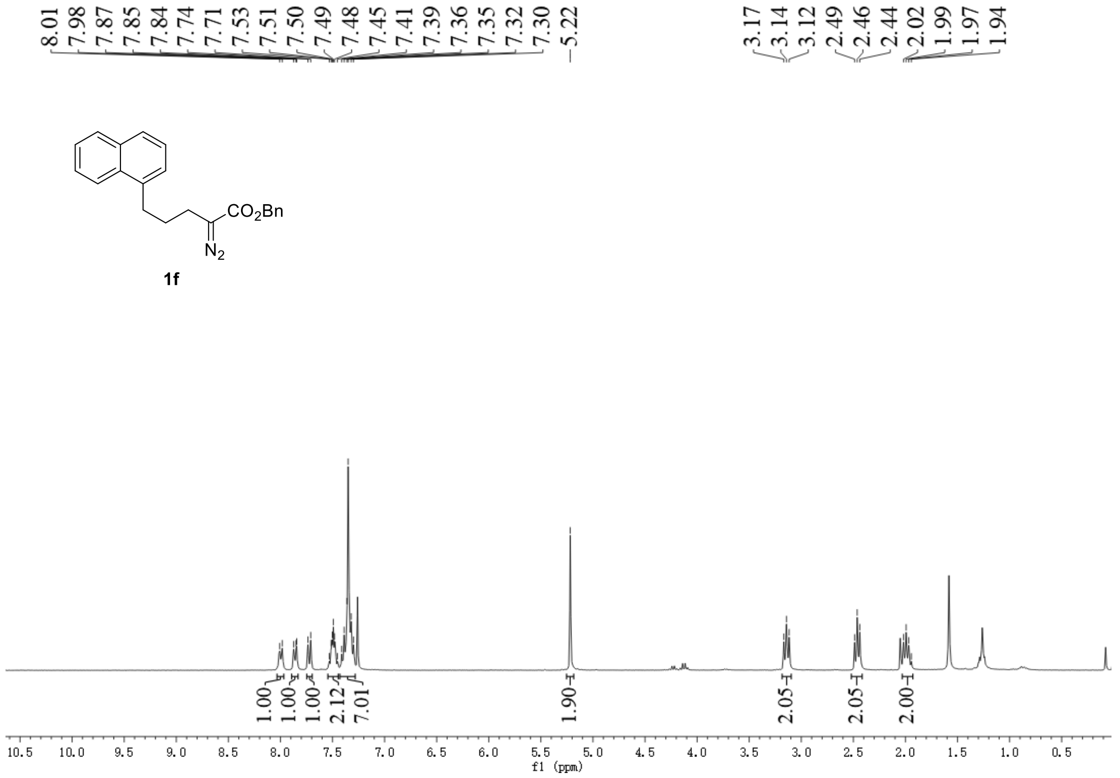
8.01 7.98 7.87 7.85 7.84 7.74 7.71 7.53 7.51 7.50 7.49 7.48 7.45 7.41 7.39 7.36 7.35 7.32 7.30 5.22
3.17 3.14 3.12 2.49 2.46 2.44 2.02 1.99 1.97 1.94
CO2Bn
N2
1f
1.00 1.00 1.00 2.12 7.01 1.90 2.05 2.05 2.00
10.5 10.0 9.5 9.0 8.5 8.0 7.5 7.0 6.5 6.0 5.5 5.0 4.5 4.0 3.5 3.0 2.5 2.0 1.5 1.0 0.5
f1 (ppm)

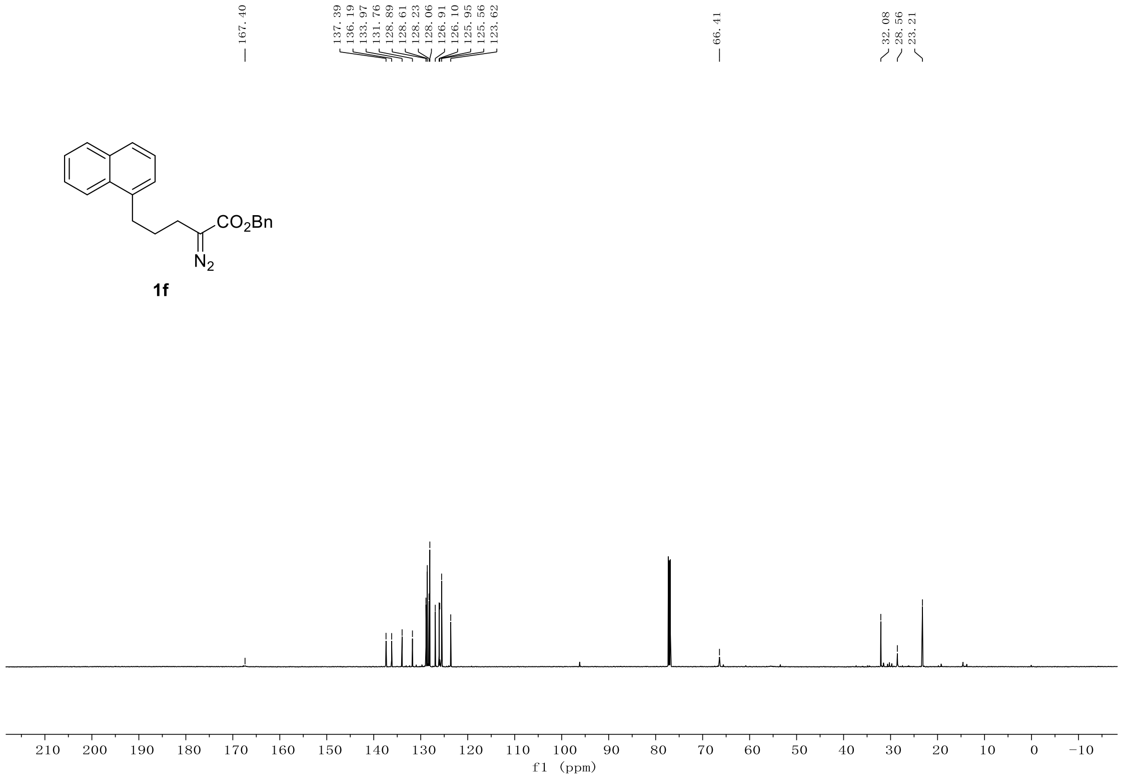
167.40
137.39 136.19 133.97 131.76 128.89 128.61 128.23 128.06 126.91 126.10 125.95 125.56 123.62
66.41
32.08 28.56 23.21
CO2Bn
N2
1f
210 200 190 180 170 160 150 140 130 120 110 100 90 80 70 60 50 40 30 20 10 0 -10
f1 (ppm)

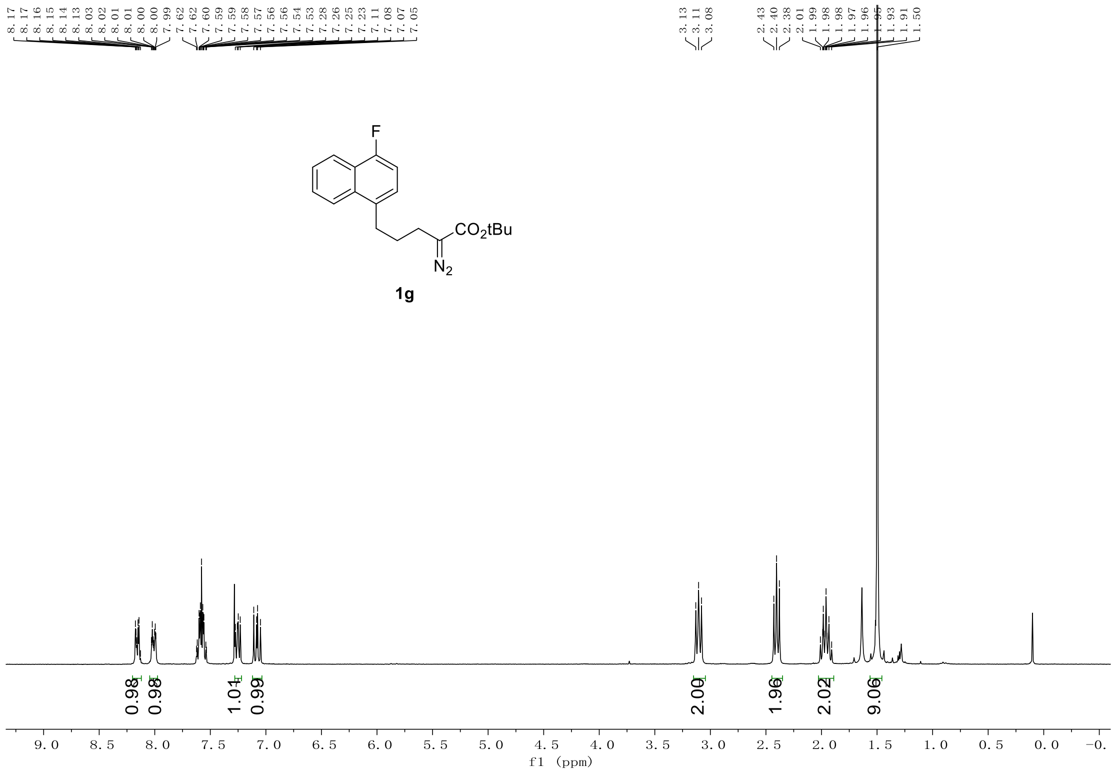
F
CO2tBu
N2
1g
f1 (ppm)

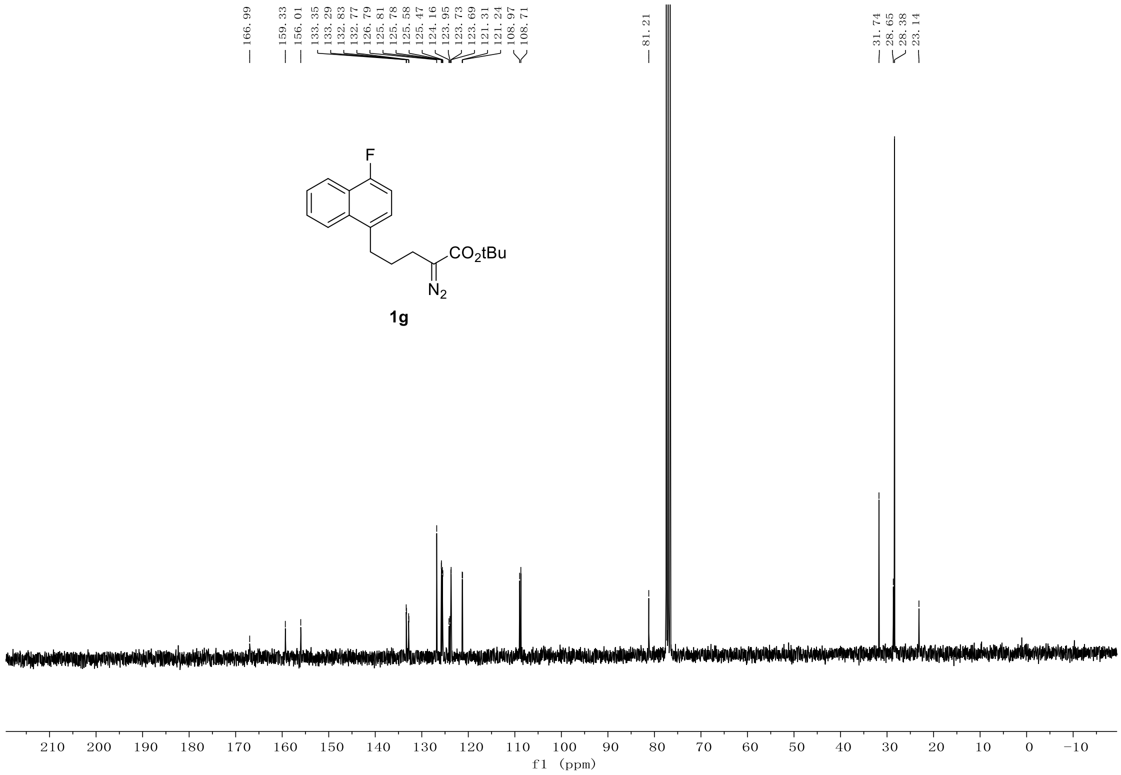
F
CO2tBu
N2
1g
f1 (ppm)

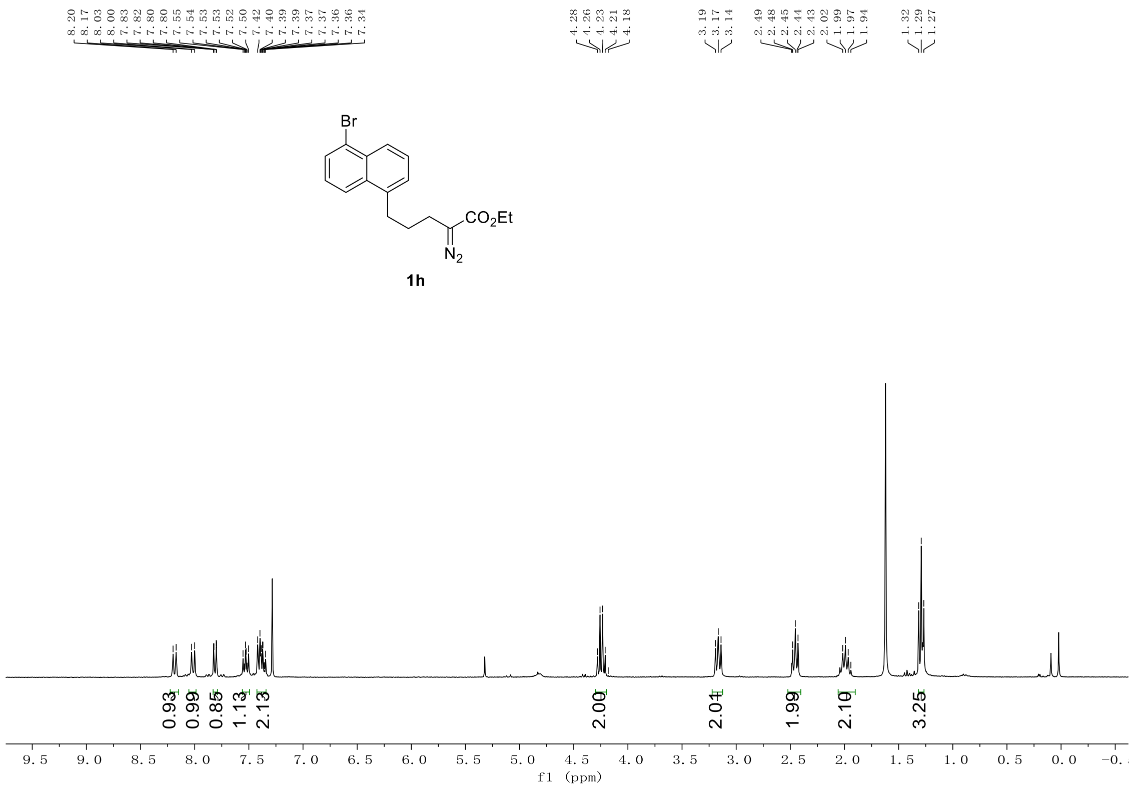
8.20 8.17 8.03 8.00 7.83 7.82 7.80 7.80 7.55 7.54 7.53 7.53 7.52 7.50 7.42 7.40 7.39 7.39 7.37 7.37 7.36 7.36 7.34
4.28 4.26 4.23 4.21 4.18
3.19 3.17 3.14
2.49 2.48 2.45 2.44 2.43 2.02 1.99 1.97 1.94
1.32 1.29 1.27
Br
CO_2Et
N_2
1h
0.93 0.99 0.85 1.13 2.13 2.00 2.04 1.99 2.10 3.25
9.5 9.0 8.5 8.0 7.5 7.0 6.5 6.0 5.5 5.0 4.5 4.0 3.5 3.0 2.5 2.0 1.5 1.0 0.5 0.0
f1 (ppm)

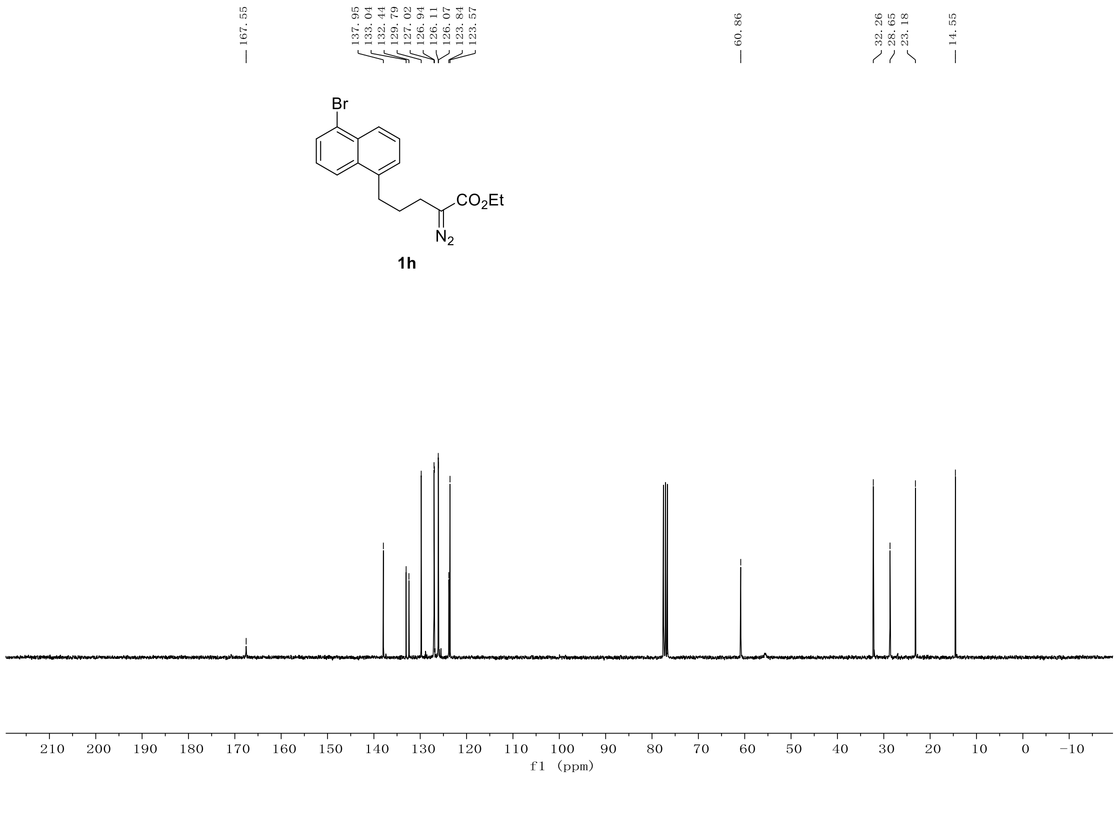
167.55
137.95 133.04 132.44 129.79 127.02 126.94 126.11 126.07 123.84 123.57
60.86
32.26 28.65 23.18
14.55
Br
CO_2Et
N_2
1h
210 200 190 180 170 160 150 140 130 120 110 100 90 80 70 60 50 40 30 20 10 0 −10
f1 (ppm)

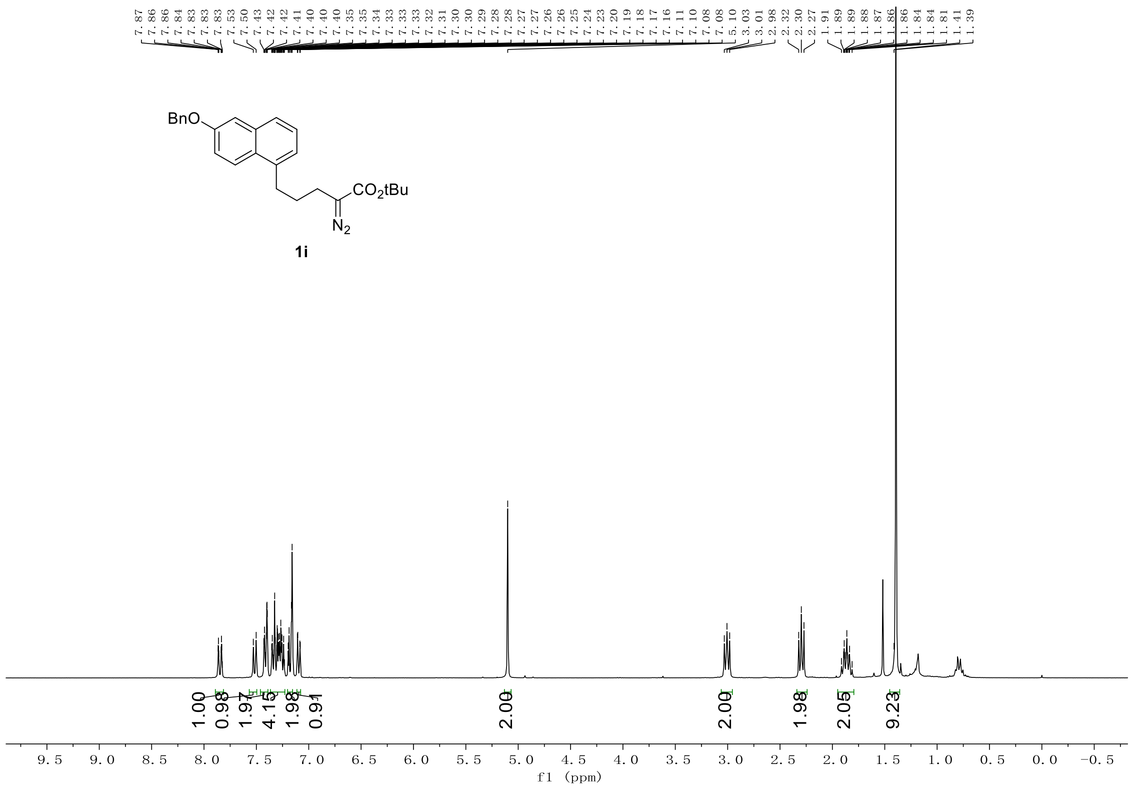
7.87 7.86 7.86 7.84 7.83 7.83 7.83 7.53 7.50 7.43 7.42 7.42 7.41 7.40 7.40 7.40 7.35 7.35 7.34 7.33 7.33 7.33 7.32 7.31 7.30 7.30 7.29 7.28 7.28 7.27 7.27 7.26 7.26 7.25 7.24 7.23 7.20 7.19 7.18 7.17 7.16 7.11 7.10 7.08 5.10 3.03 3.01 2.98 2.32 2.30 2.27 1.91 1.89 1.89 1.88 1.87 1.86 1.86 1.84 1.84 1.81 1.41 1.39
BnO
CO2tBu
N2
1i
1.00 0.98 1.97 4.15 1.98 0.91 2.00 2.00 1.98 2.05 9.23
f1 (ppm)

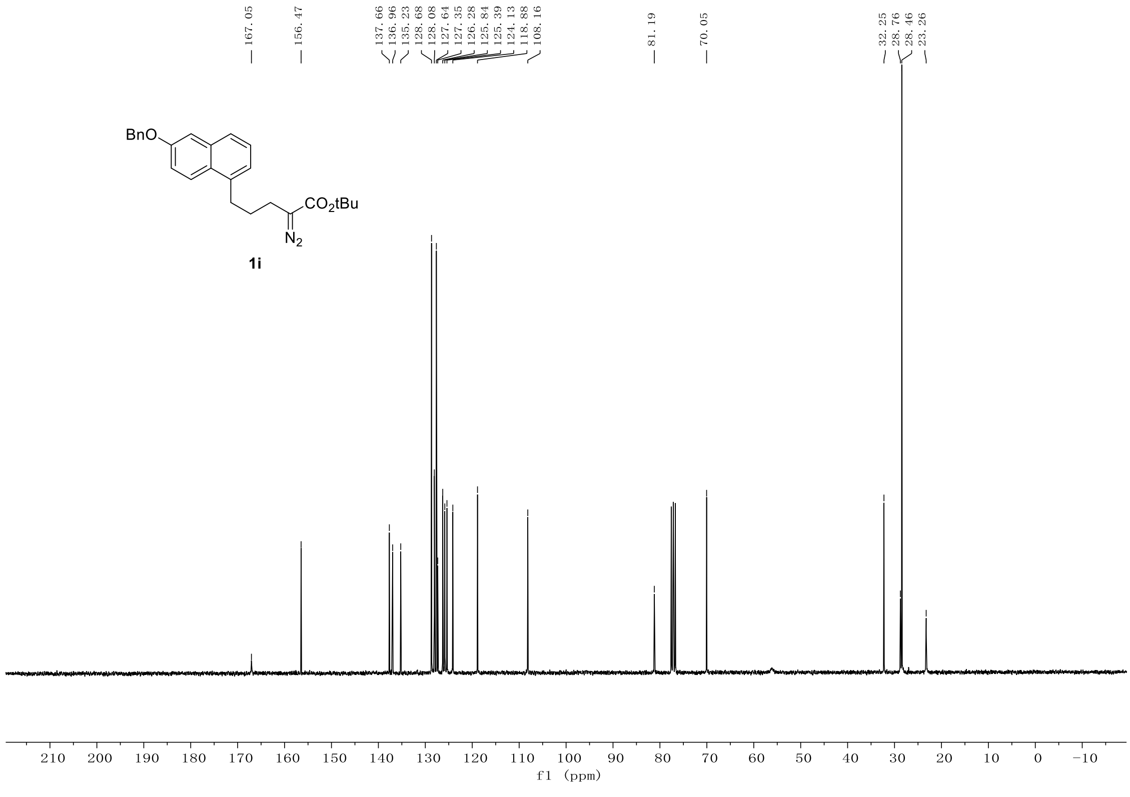
167.05
156.47
137.66 136.96 135.23 128.68 128.08 127.64 127.35 126.28 125.84 125.39 124.13 118.88 108.16
81.19
70.05
32.25 28.76 28.46 23.26
BnO
CO2tBu
N2
1i
f1 (ppm)

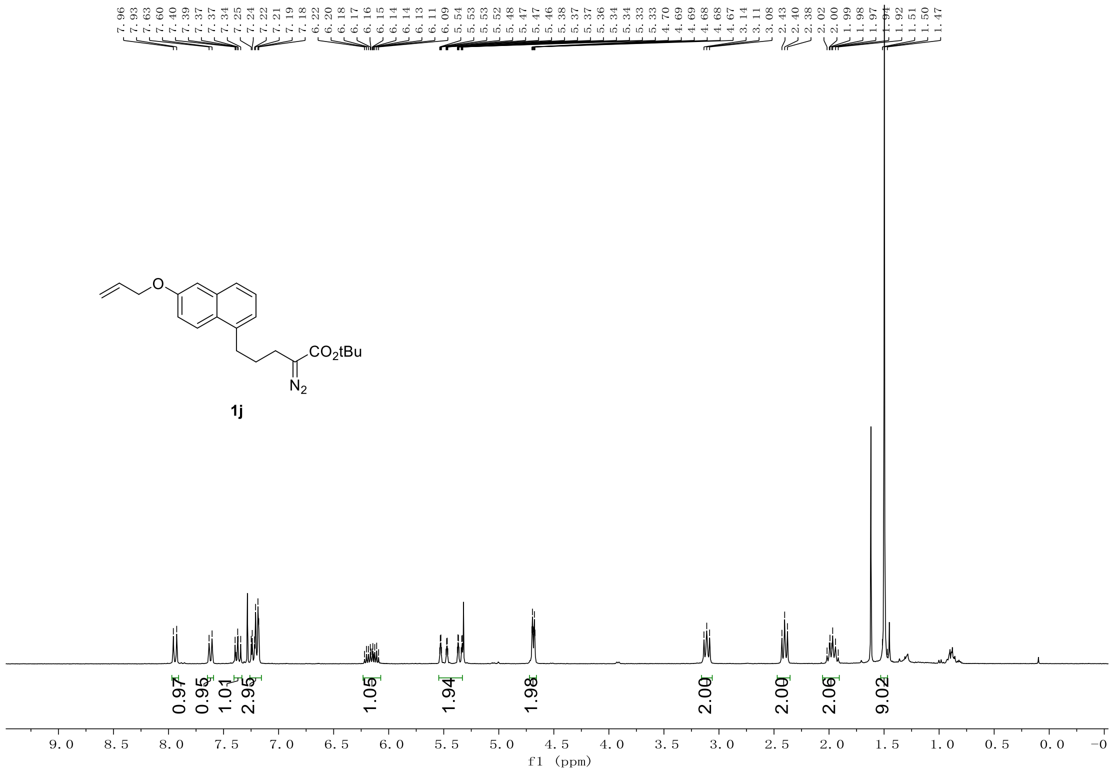

1j

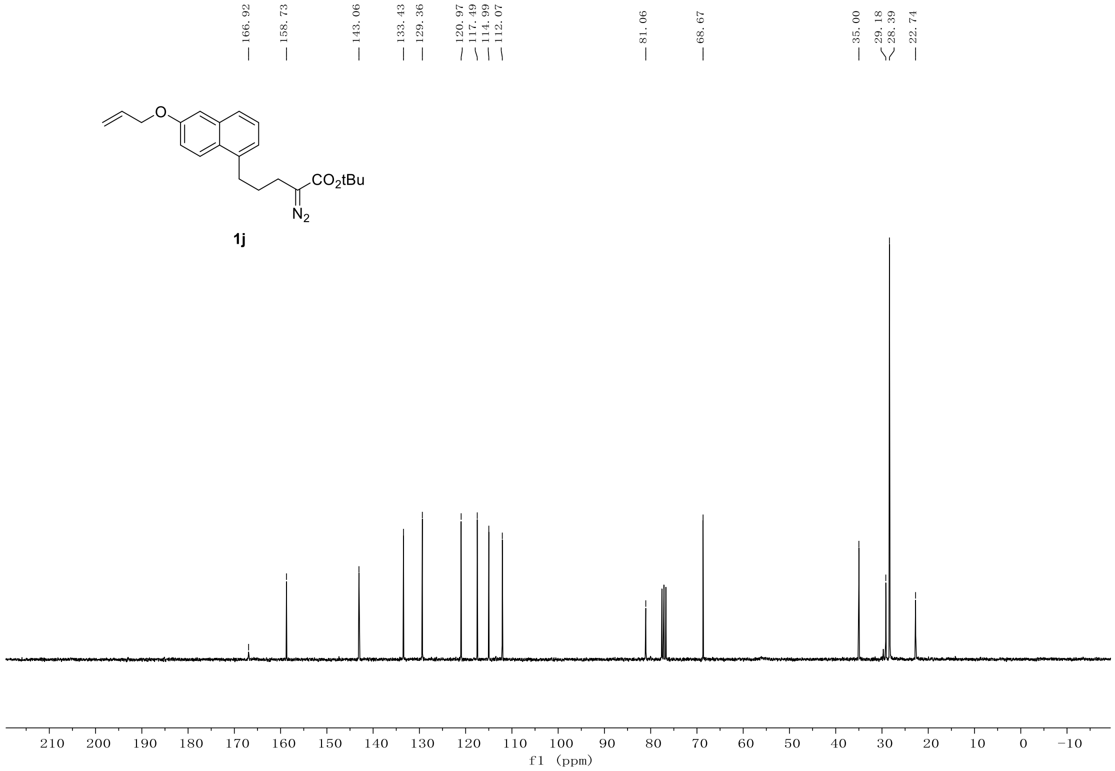

1j

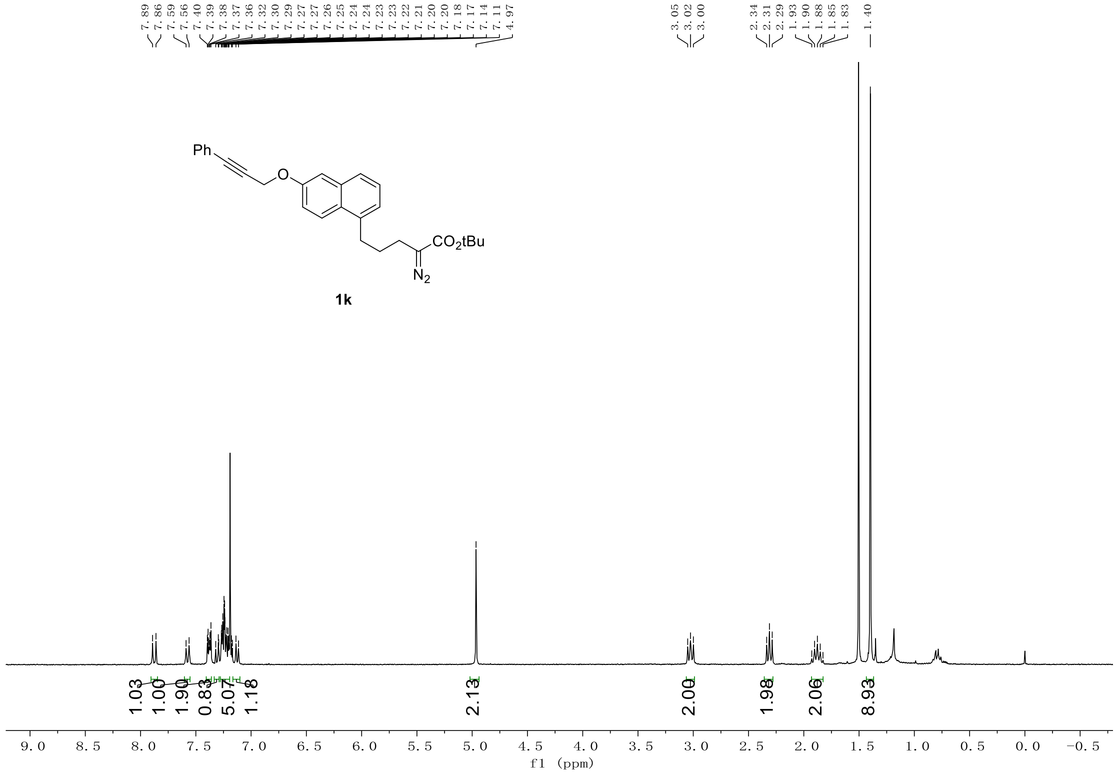
7.89 7.86 7.59 7.56 7.40 7.39 7.38 7.37 7.36 7.32 7.30 7.29 7.27 7.27 7.26 7.25 7.24 7.24 7.23 7.23 7.22 7.21 7.20 7.20 7.18 7.17 7.14 7.11 4.97
3.05 3.02 3.00
2.34 2.31 2.29 1.93 1.90 1.88 1.85 1.83
1.40
Ph
O
CO2tBu
N2
1k
1.03 1.00 1.90 0.83 5.07 1.18
2.13
2.00
1.98
2.06
8.93
f1 (ppm)

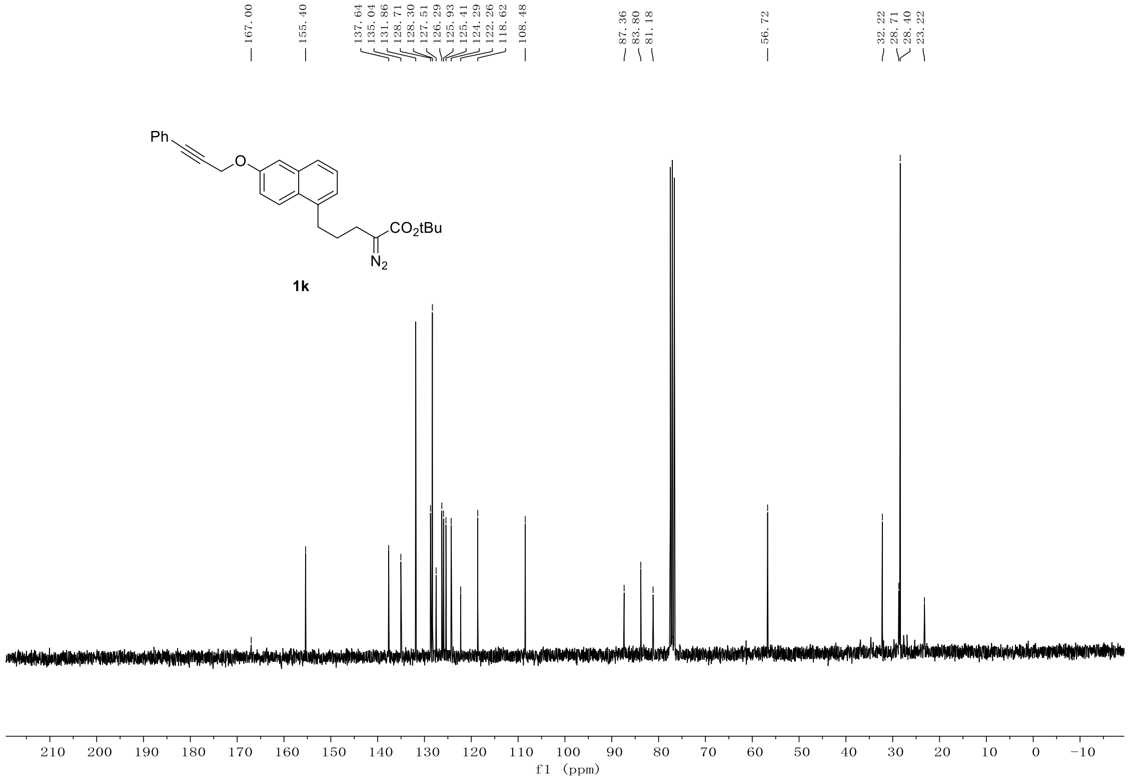
167.00
155.40
137.64 135.04 131.86 128.71 128.30 127.51 126.29 125.93 125.41 124.29 122.26 118.62
108.48
87.36 83.80 81.18
56.72
32.22 28.71 28.40 23.22
Ph
O
CO2tBu
N2
1k
f1 (ppm)

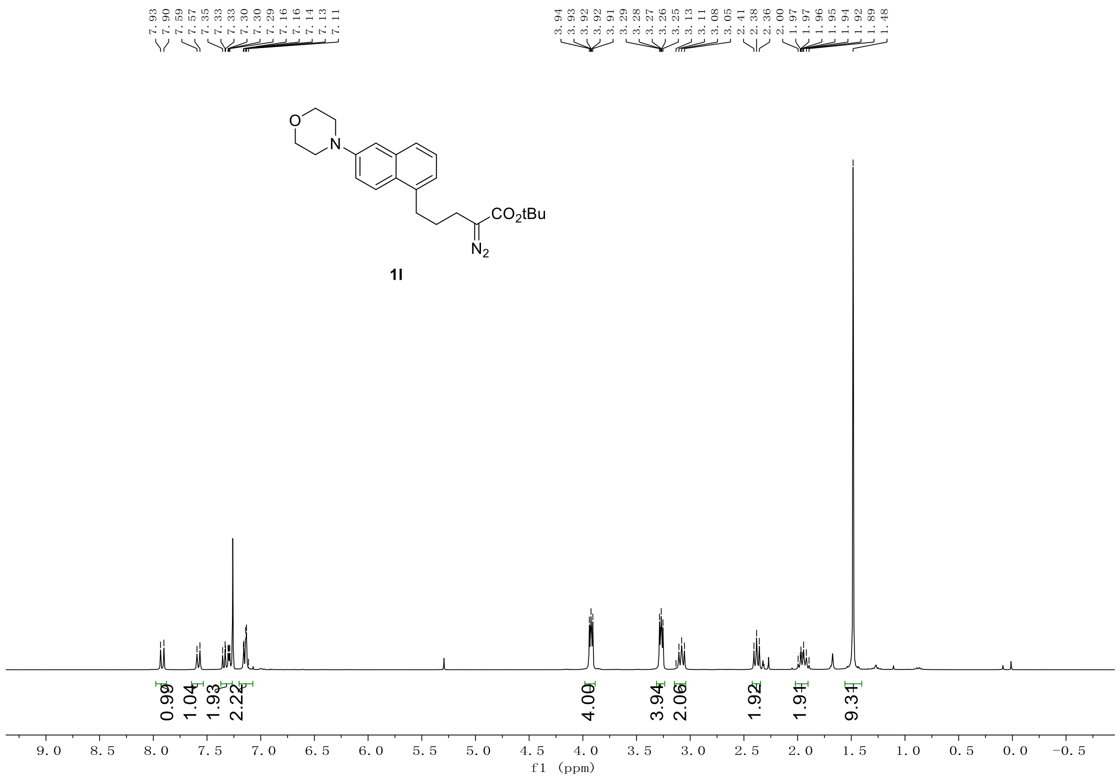
7.93 7.90 7.59 7.57 7.35 7.33 7.33 7.30 7.29 7.16 7.14 7.13 7.11
3.94 3.93 3.92 3.92 3.91 3.29 3.28 3.27 3.26 3.25 3.13 3.11 3.08 3.05 2.41 2.38 2.36 2.00 1.97 1.96 1.95 1.94 1.92 1.89 1.48
O
N
CO2tBu
N2
1l
0.99 1.04 1.93 2.22 4.00 3.94 2.06 1.92 1.91 9.31
9.0 8.5 8.0 7.5 7.0 6.5 6.0 5.5 5.0 4.5 4.0 3.5 3.0 2.5 2.0 1.5 1.0 0.5 0.0 -0.5
f1 (ppm)

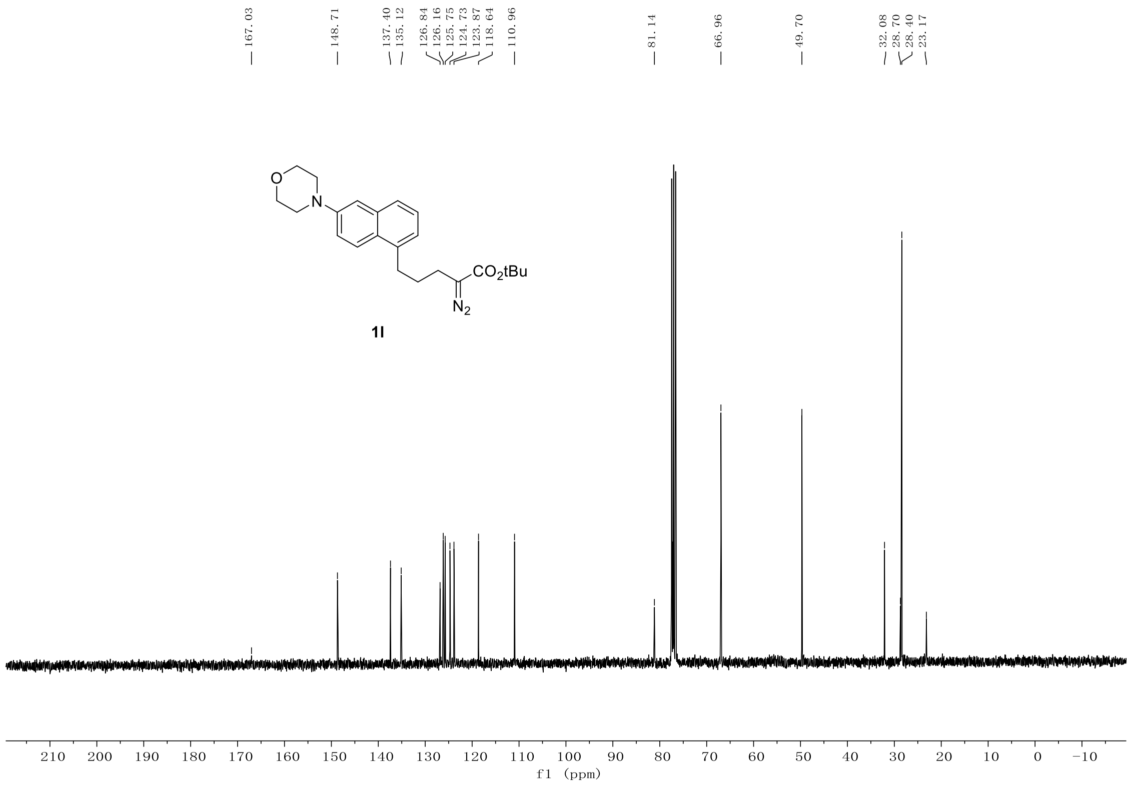
167.03
148.71
137.40 135.12
126.84 126.16 125.75 124.73 123.87 118.64
110.96
81.14
66.96
49.70
32.08 28.70 28.40 23.17
O
N
CO2tBu
N2
1l
210 200 190 180 170 160 150 140 130 120 110 100 90 80 70 60 50 40 30 20 10 0 -10
f1 (ppm)

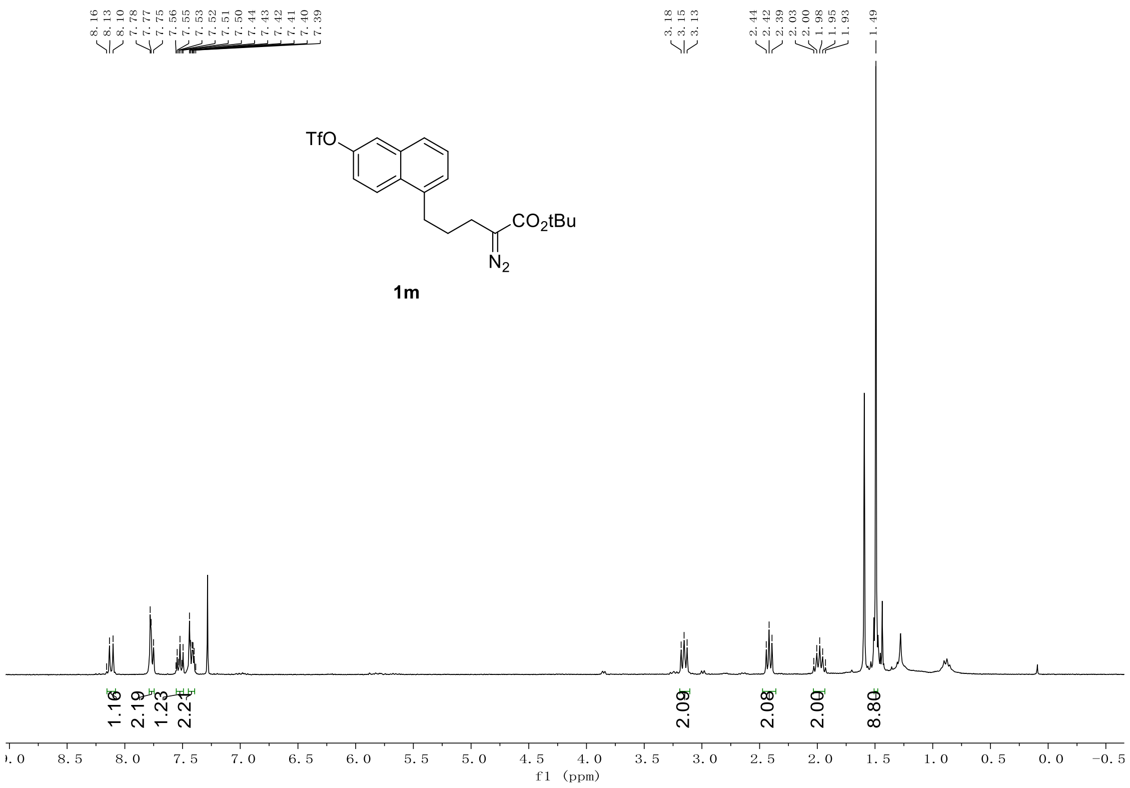
TfO
CO2tBu
N2
1m
8.16
8.13
8.10
7.78
7.77
7.75
7.56
7.55
7.53
7.52
7.51
7.50
7.44
7.43
7.42
7.41
7.40
7.39
3.18
3.15
3.13
2.44
2.42
2.39
2.03
2.00
1.98
1.95
1.93
1.49
1.16
2.19
1.23
2.24
2.09
2.08
2.00
8.80
f1 (ppm)

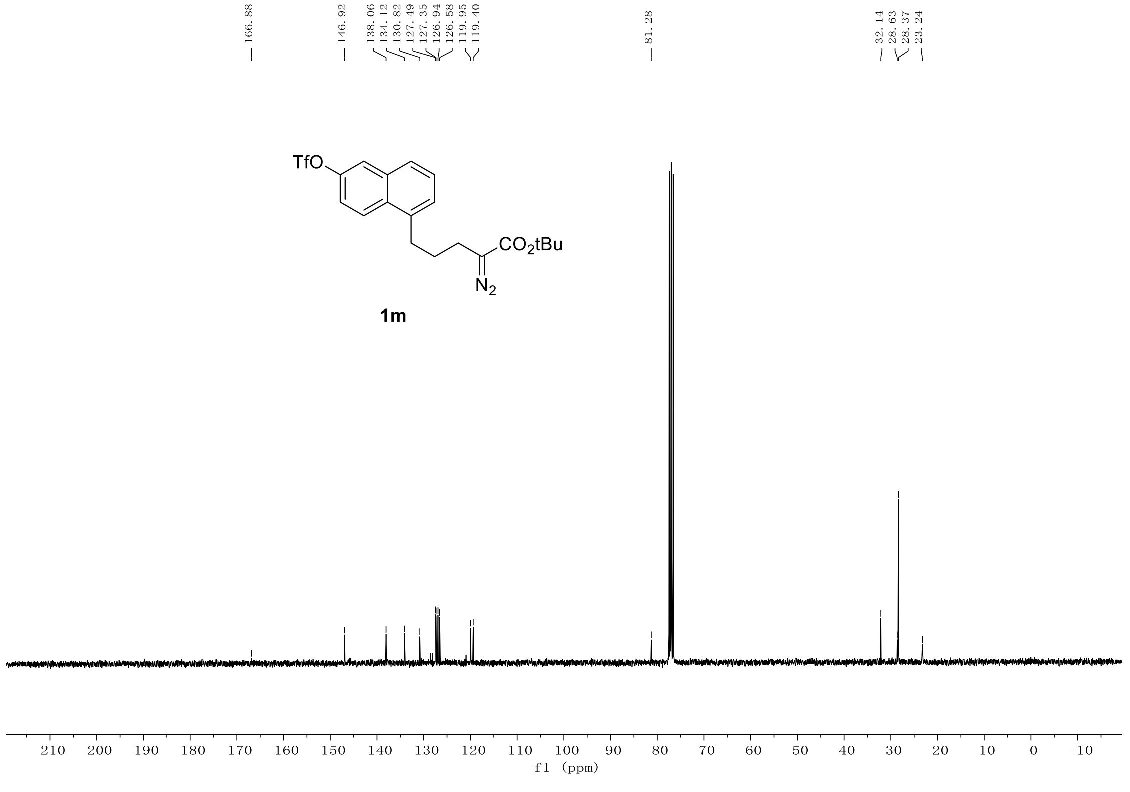
TfO
CO2tBu
N2
1m
166.88
146.92
138.06
134.12
130.82
127.49
127.35
126.94
126.58
119.95
119.40
81.28
32.14
28.63
28.37
23.24
f1 (ppm)

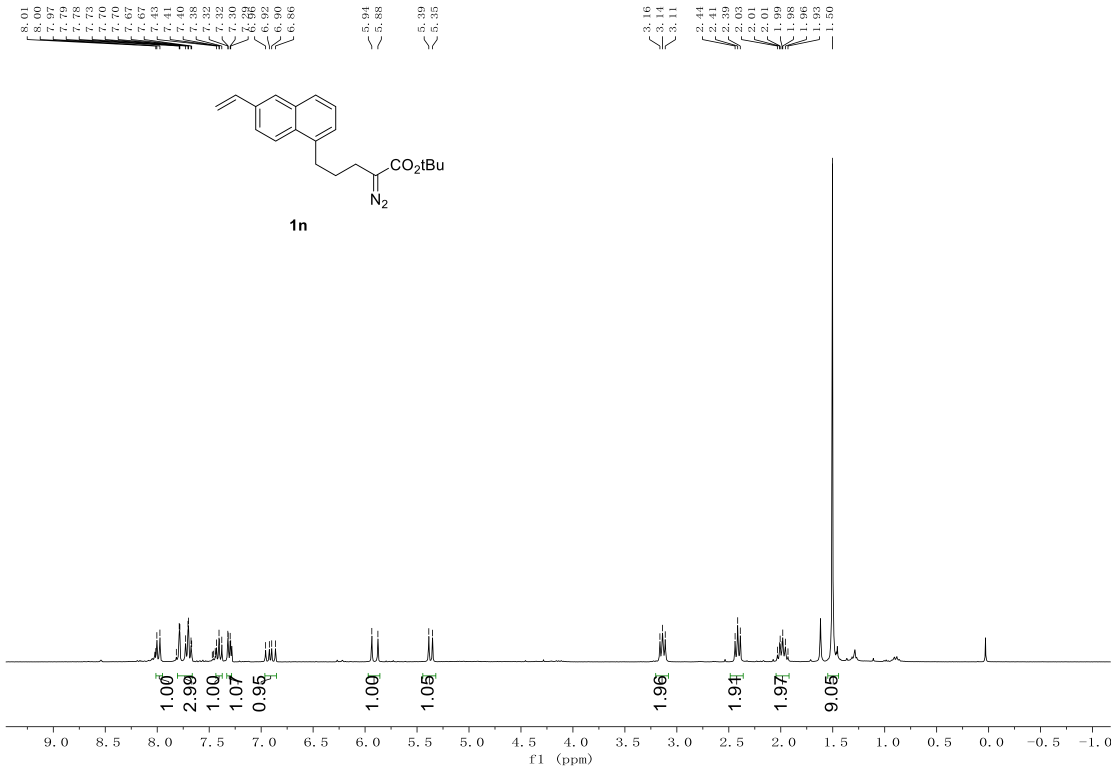
8.01 8.00 7.97 7.79 7.78 7.73 7.70 7.70 7.67 7.43 7.41 7.40 7.38 7.32 7.32 7.30 7.29 6.96 6.92 6.90 6.86
5.94 5.88
5.39 5.35
3.16 3.14 3.11
2.44 2.41 2.39 2.03 2.01 1.99 1.98 1.96 1.93
1.50
CO2tBu
N2
1n
1.00 2.99 1.00 1.07 0.95 1.00 1.05 1.96 1.91 1.97 9.05
f1 (ppm)

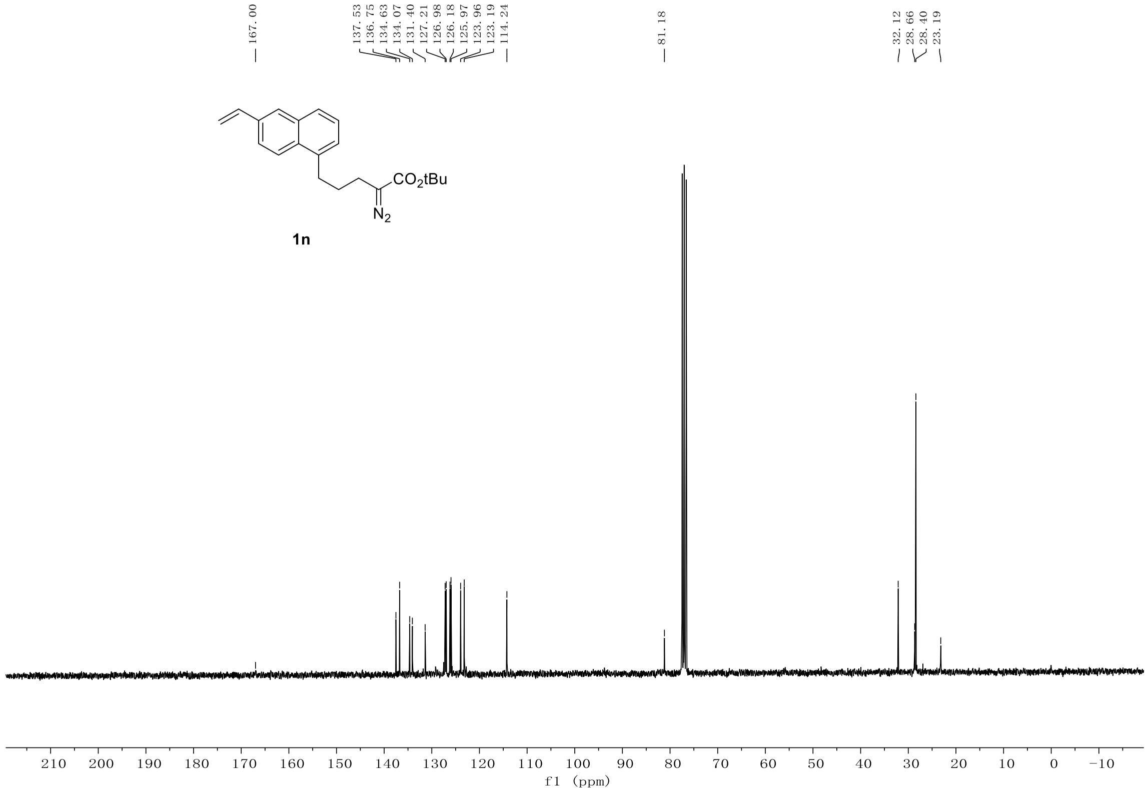
167.00
137.53 136.75 134.63 134.07 131.40 127.21 126.98 126.18 125.97 123.96 123.19 114.24
81.18
32.12 28.66 28.40 23.19
CO2tBu
N2
1n
f1 (ppm)

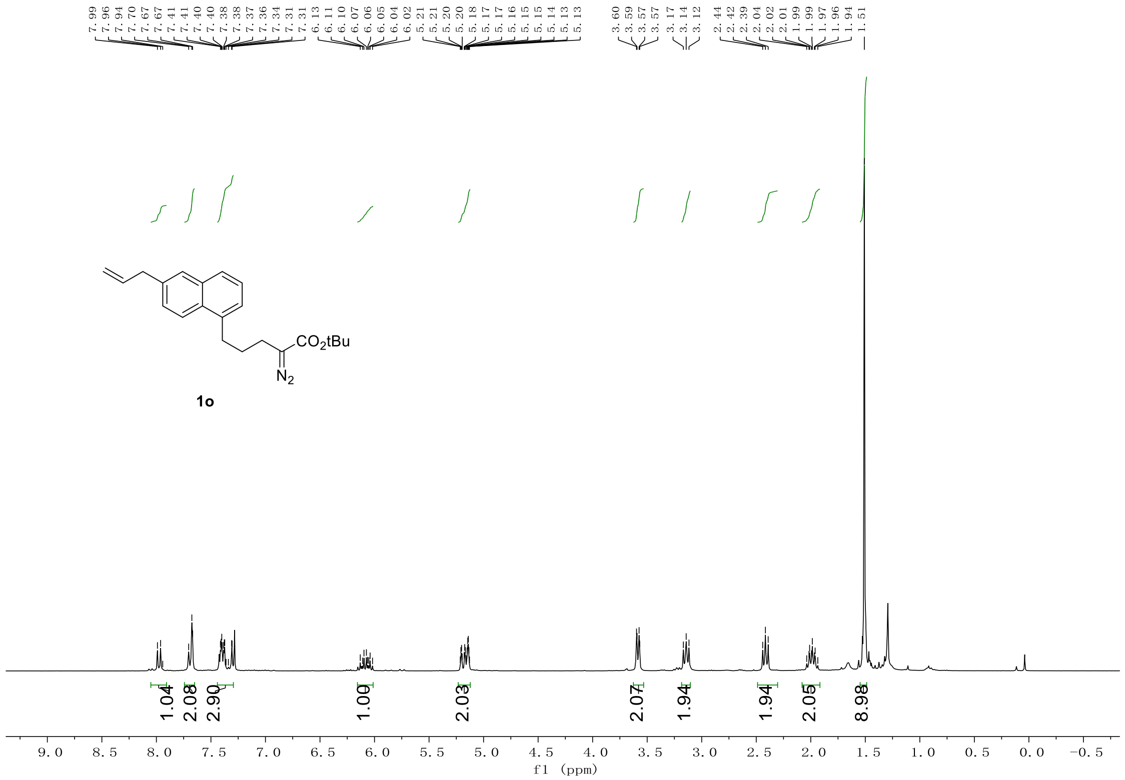
7.99 7.96 7.94 7.70 7.67 7.67 7.41 7.41 7.40 7.38 7.38 7.37 7.36 7.34 7.31 7.31 6.13 6.11 6.10 6.07 6.06 6.05 6.04 6.02 5.21 5.21 5.20 5.20 5.18 5.17 5.17 5.16 5.15 5.15 5.14 5.13 5.13 3.60 3.59 3.57 3.57 3.17 3.14 3.12 2.44 2.42 2.39 2.04 2.02 2.01 1.99 1.99 1.97 1.96 1.94 1.51
CO_2tBu
N_2
1o
1.04 2.08 2.90 1.00 2.03 2.07 1.94 1.94 2.05 8.98
9.0 8.5 8.0 7.5 7.0 6.5 6.0 5.5 5.0 4.5 4.0 3.5 3.0 2.5 2.0 1.5 1.0 0.5 0.0 -0.5
f1 (ppm)

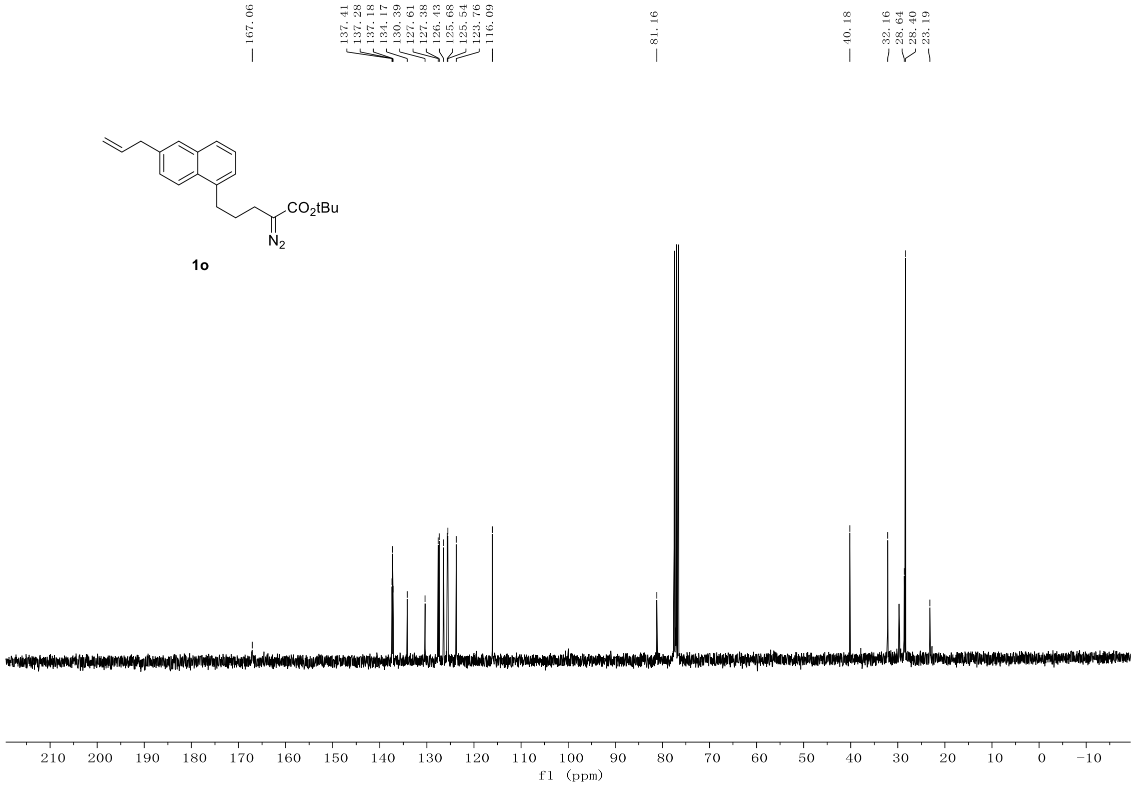
167.06 137.41 137.28 137.18 134.17 130.39 127.61 127.38 126.43 125.68 125.54 123.76 116.09 81.16 40.18 32.16 28.64 28.40 23.19
CO_2tBu
N_2
1o
210 200 190 180 170 160 150 140 130 120 110 100 90 80 70 60 50 40 30 20 10 0 -10
f1 (ppm)

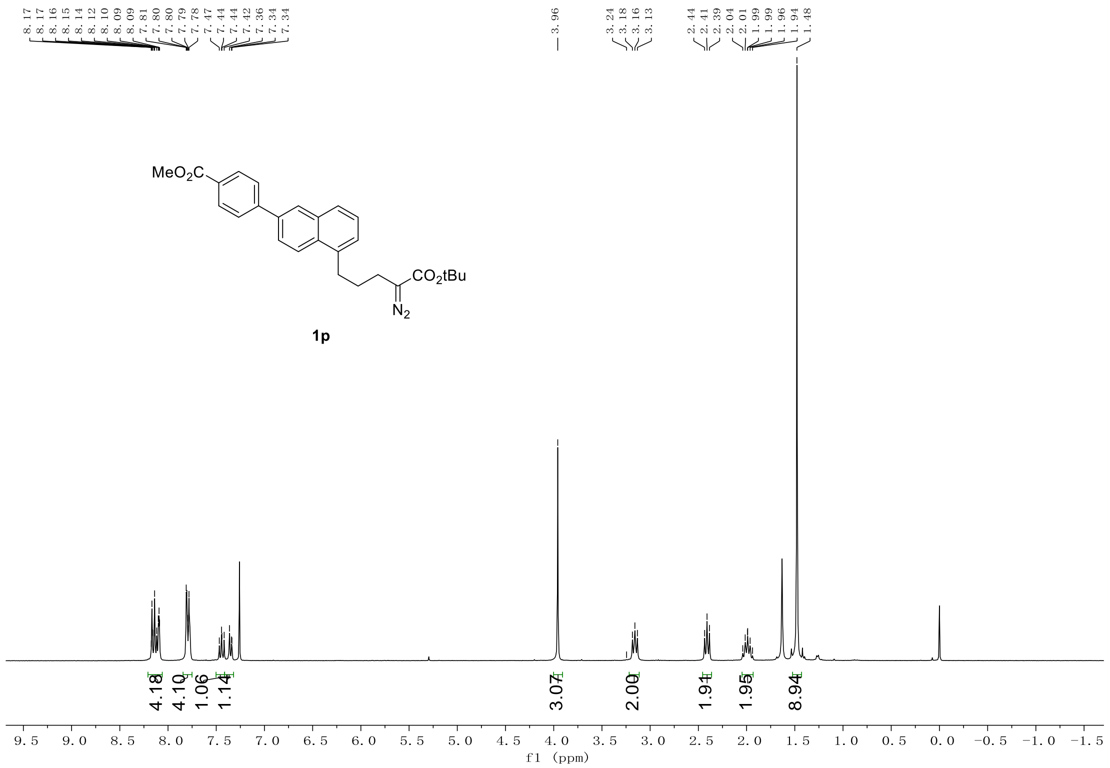
8.17
8.17
8.16
8.15
8.14
8.12
8.10
8.09
8.09
7.81
7.80
7.80
7.79
7.78
7.47
7.44
7.44
7.42
7.36
7.34
7.34
3.96
3.24
3.18
3.16
3.13
2.44
2.41
2.39
2.04
2.01
1.99
1.99
1.96
1.94
1.48
MeO2C
CO2tBu
N2
1p
4.18
4.10
1.06
1.14
3.07
2.00
1.91
1.95
8.94
f1 (ppm)

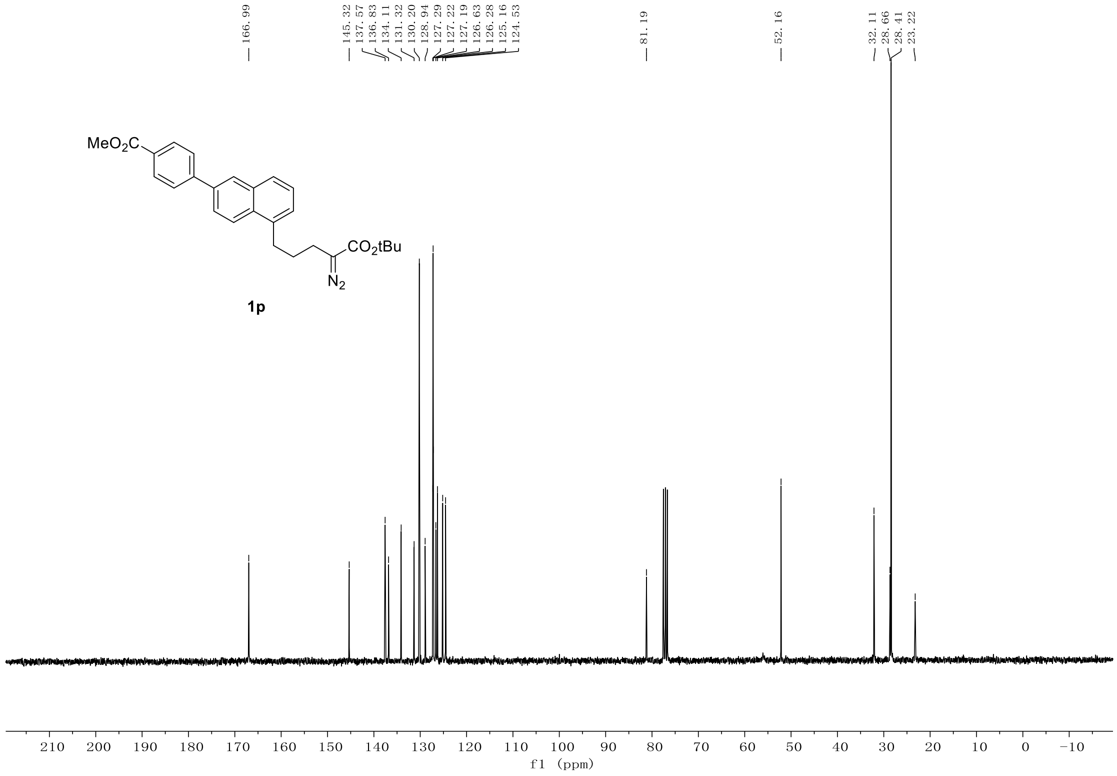
166.99
145.32
137.57
136.83
134.11
131.32
130.20
128.94
127.29
127.22
127.19
126.63
126.28
125.16
124.53
81.19
52.16
32.11
28.66
28.41
23.22
MeO2C
CO2tBu
N2
1p
f1 (ppm)

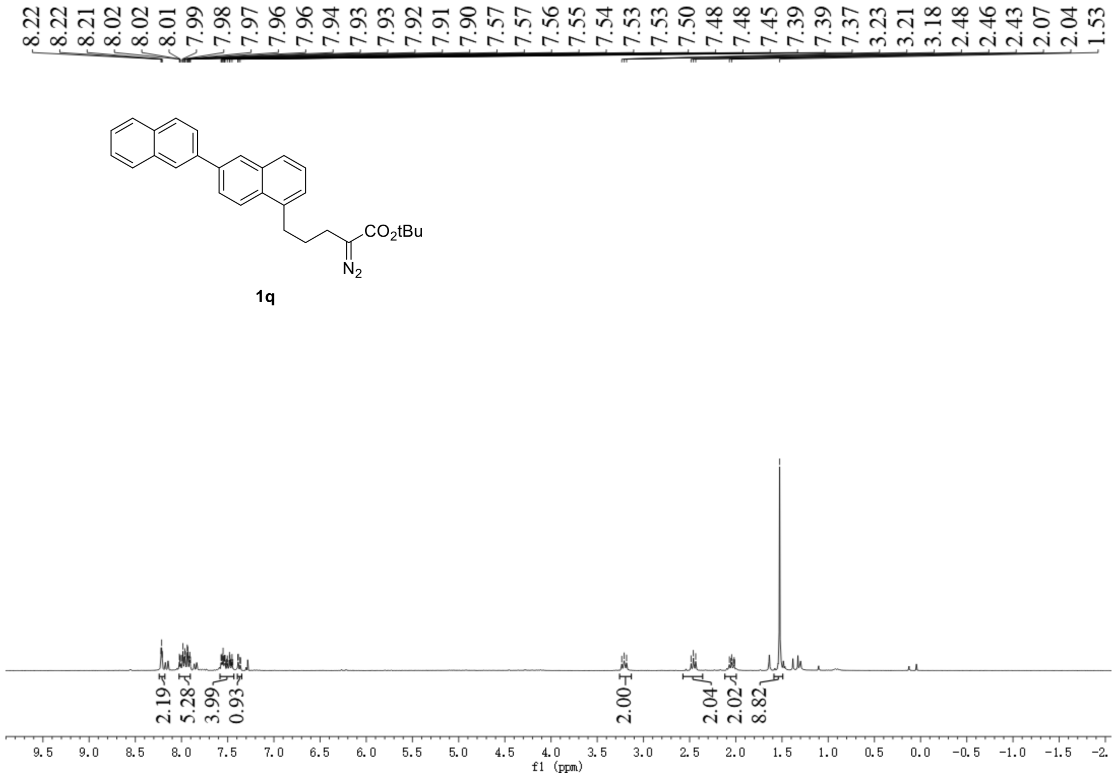
8.22 8.22 8.21 8.02 8.02 8.01 7.99 7.98 7.97 7.96 7.96 7.94 7.93 7.93 7.92 7.91 7.90 7.57 7.57 7.56 7.55 7.54 7.53 7.53 7.50 7.48 7.48 7.45 7.39 7.39 7.37 3.23 3.21 3.18 2.48 2.46 2.43 2.07 2.04 1.53
CO2tBu
N2
1q
2.19 5.28 3.99 0.93 2.00 2.04 2.02 8.82
9.5 9.0 8.5 8.0 7.5 7.0 6.5 6.0 5.5 5.0 4.5 4.0 3.5 3.0 2.5 2.0 1.5 1.0 0.5 0.0 -0.5 -1.0 -1.5 -2.
f1 (ppm)

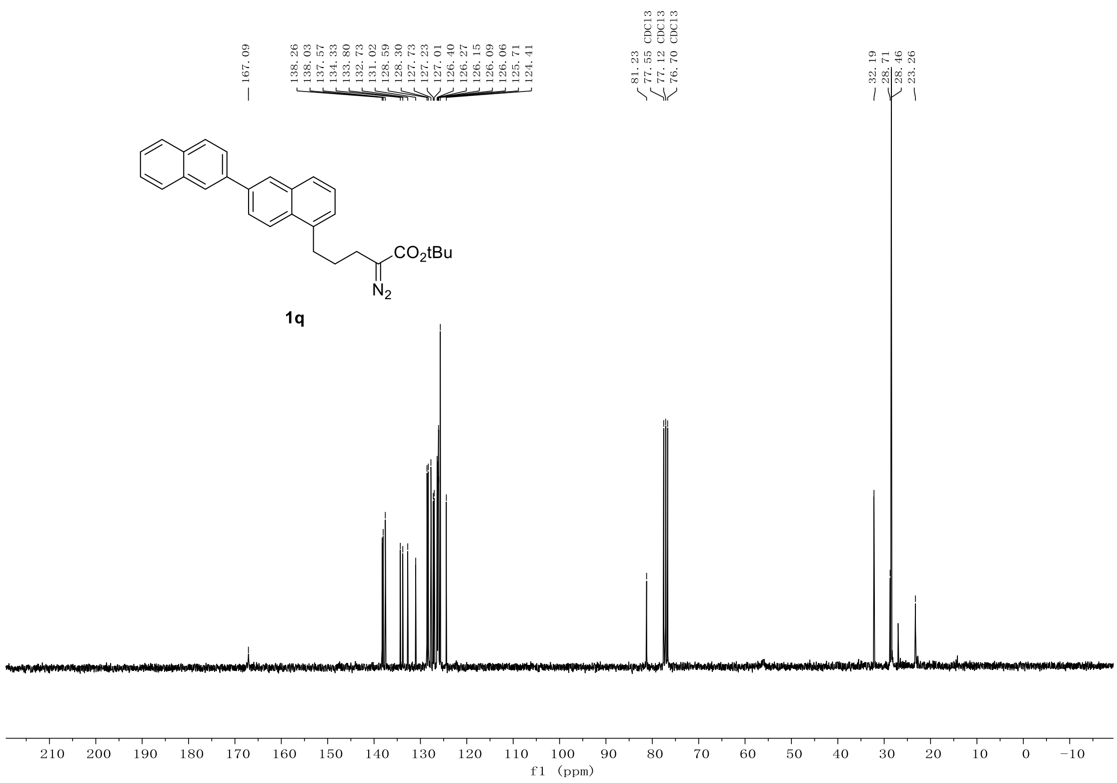
167.09
138.26 138.03 137.57 134.33 133.80 132.73 131.02 128.59 128.30 127.73 127.23 127.01 126.40 126.27 126.15 126.09 126.06 125.71 124.41
81.23 77.55 CDCl3 77.12 CDCl3 76.70 CDCl3
32.19 28.71 28.46 23.26
CO2tBu
N2
1q
210 200 190 180 170 160 150 140 130 120 110 100 90 80 70 60 50 40 30 20 10 0 -10
f1 (ppm)

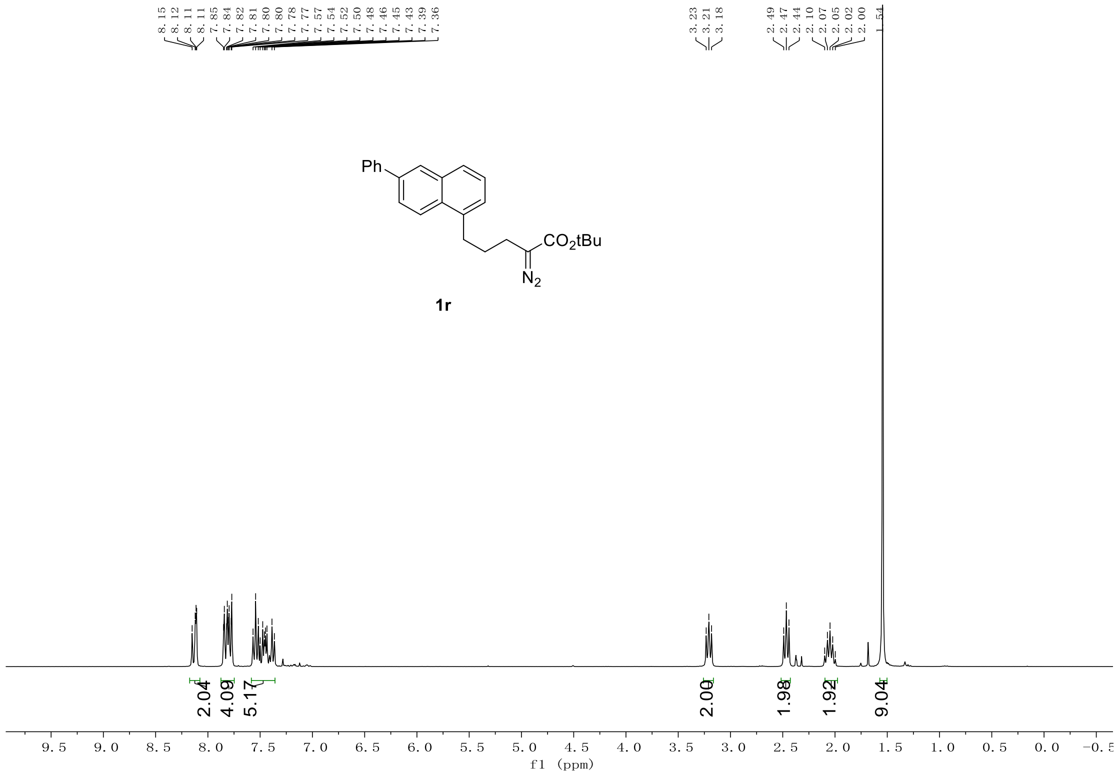
8.15
8.12
8.11
8.11
7.85
7.84
7.82
7.81
7.80
7.80
7.78
7.77
7.57
7.54
7.52
7.50
7.48
7.46
7.45
7.43
7.39
7.36
3.23
3.21
3.18
2.49
2.47
2.44
2.10
2.07
2.05
2.02
2.00
1.54
Ph
CO2tBu
N2
1r
2.04
4.09
5.17
2.00
1.98
1.92
9.04
f1 (ppm)

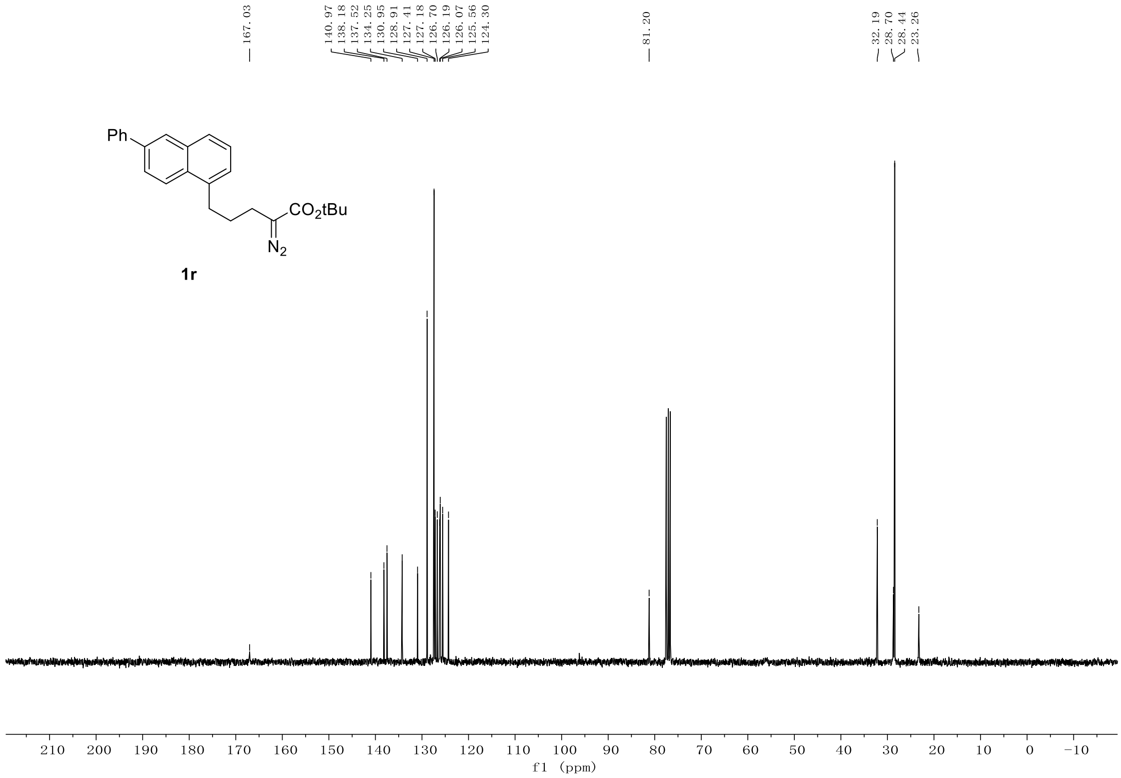
167.03
140.97
138.18
137.52
134.25
130.95
128.91
127.41
127.18
126.70
126.19
126.07
125.56
124.30
81.20
32.19
28.70
28.44
23.26
Ph
CO2tBu
N2
1r
f1 (ppm)

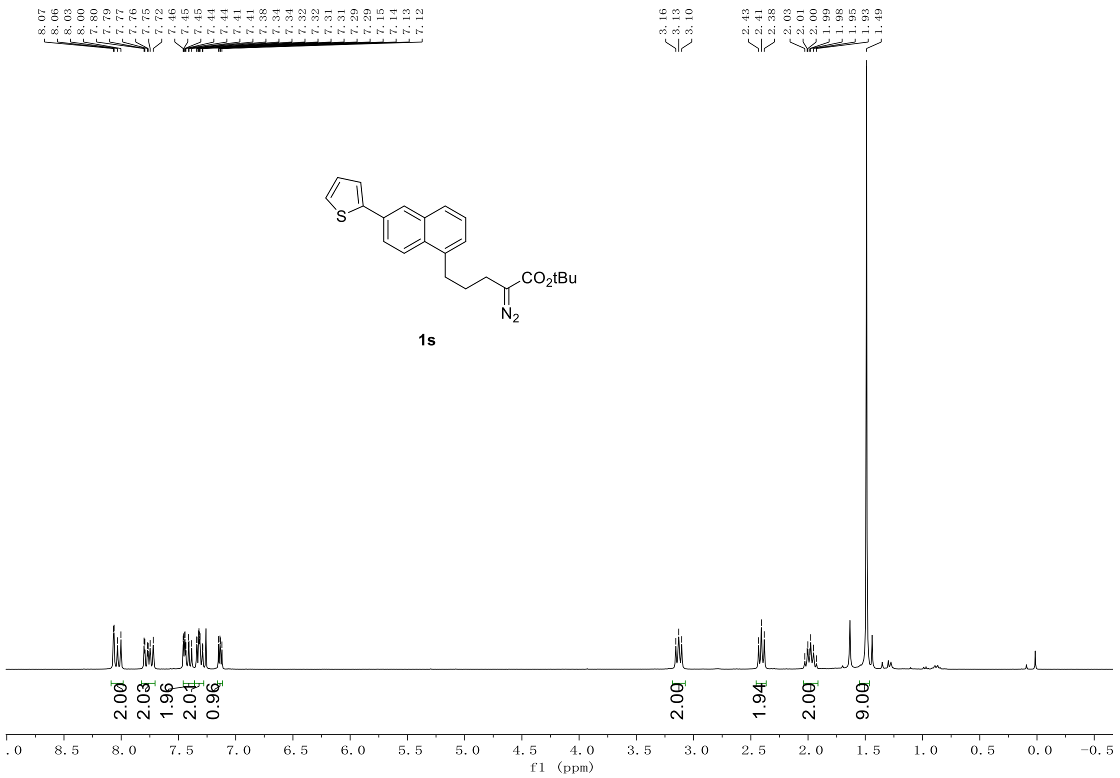
8.07 8.06 8.03 8.00 7.80 7.79 7.77 7.76 7.75 7.72 7.46 7.45 7.45 7.44 7.44 7.41 7.41 7.38 7.34 7.34 7.32 7.32 7.31 7.31 7.29 7.29 7.15 7.14 7.13 7.12
3.16 3.13 3.10
2.43 2.41 2.38 2.03 2.01 2.00 1.99 1.98 1.95 1.93 1.49
S
CO2tBu
N2
1s
2.00 2.03 1.96 2.04 0.96 2.00 1.94 2.00 9.00
f1 (ppm)

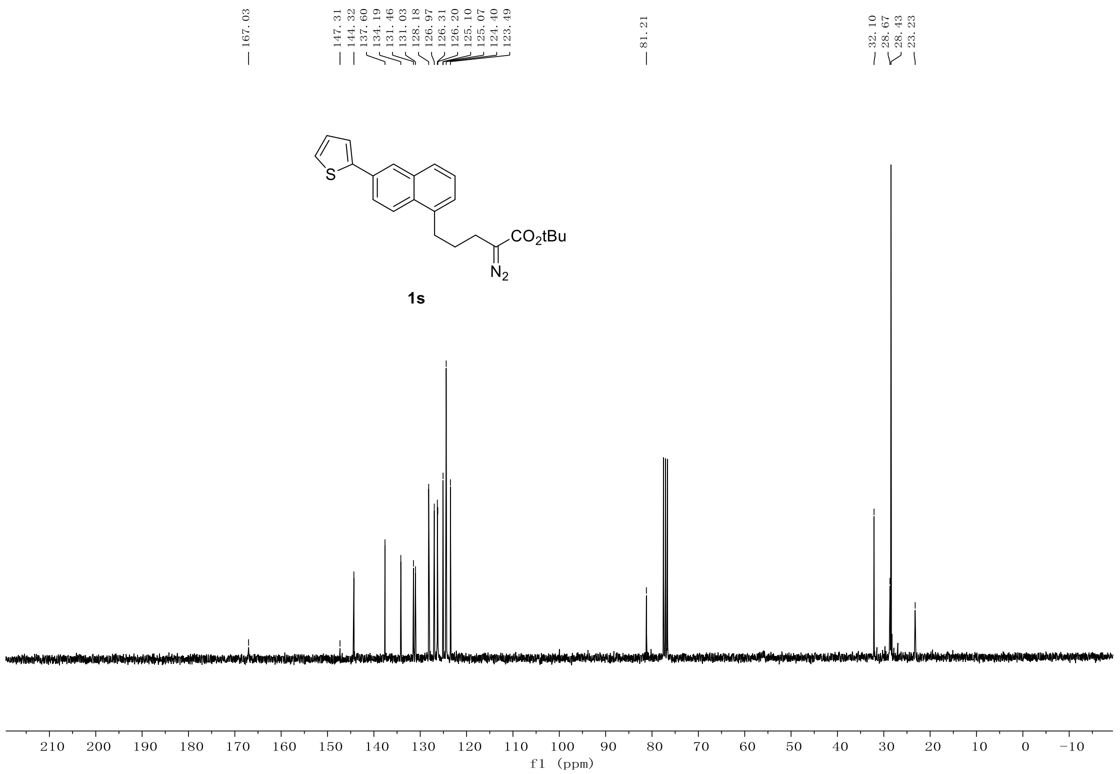
167.03
147.31 144.32 137.60 134.19 131.46 131.03 128.18 126.97 126.31 126.20 125.10 125.07 124.40 123.49
81.21
32.10 28.67 28.43 23.23
S
CO2tBu
N2
1s
f1 (ppm)

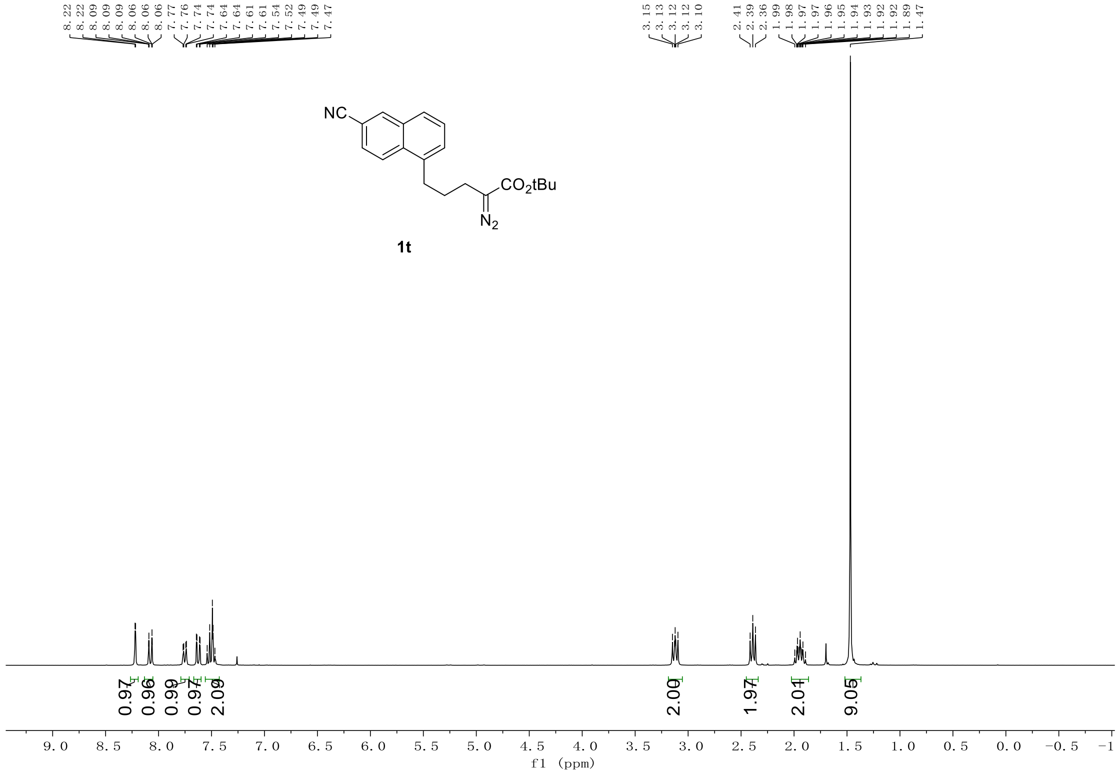
8.22
8.22
8.09
8.09
8.06
8.06
7.77
7.76
7.74
7.74
7.64
7.64
7.61
7.61
7.54
7.52
7.49
7.47
3.15
3.13
3.12
3.12
3.10
2.41
2.39
2.36
1.99
1.98
1.97
1.97
1.96
1.95
1.94
1.93
1.92
1.92
1.89
1.47
NC
CO2tBu
N2
1t
0.97
0.96
0.99
0.97
2.09
2.00
1.97
2.01
9.05
f1 (ppm)

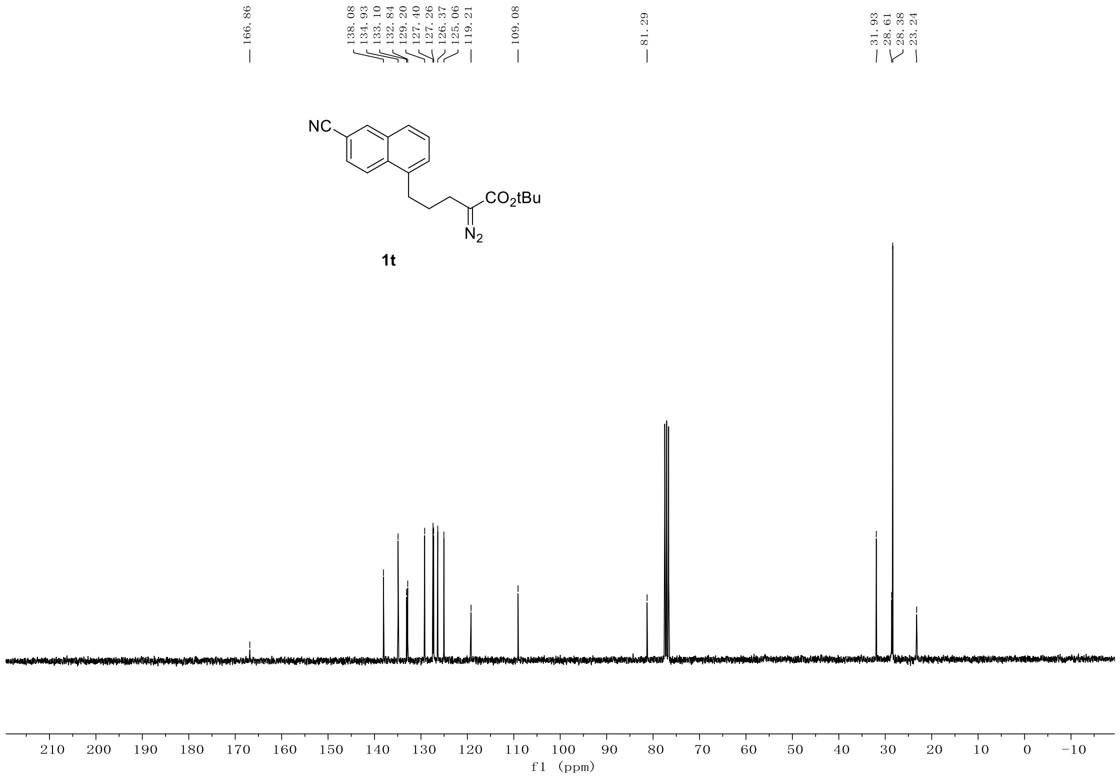
166.86
138.08
134.93
133.10
132.84
129.20
127.40
127.26
126.37
125.06
119.21
109.08
81.29
31.93
28.61
28.38
23.24
NC
CO2tBu
N2
1t
f1 (ppm)

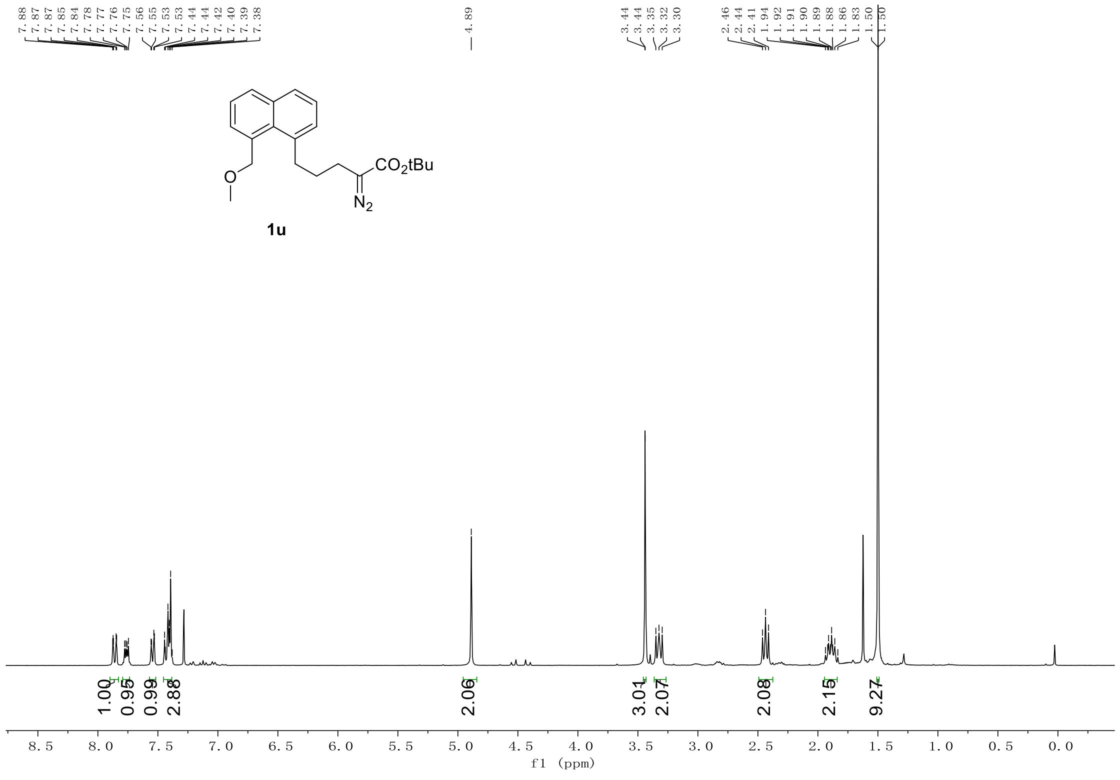

7.88
7.87
7.87
7.85
7.84
7.78
7.77
7.76
7.75
7.56
7.55
7.53
7.53
7.44
7.44
7.42
7.40
7.39
7.38
4.89
3.44
3.44
3.35
3.32
3.30
2.46
2.44
2.41
1.94
1.92
1.91
1.90
1.89
1.88
1.86
1.83
1.50
1.50
CO2tBu
N2
1u
1.00
0.95
0.99
2.88
2.06
3.01
2.07
2.08
2.15
9.27
f1 (ppm)

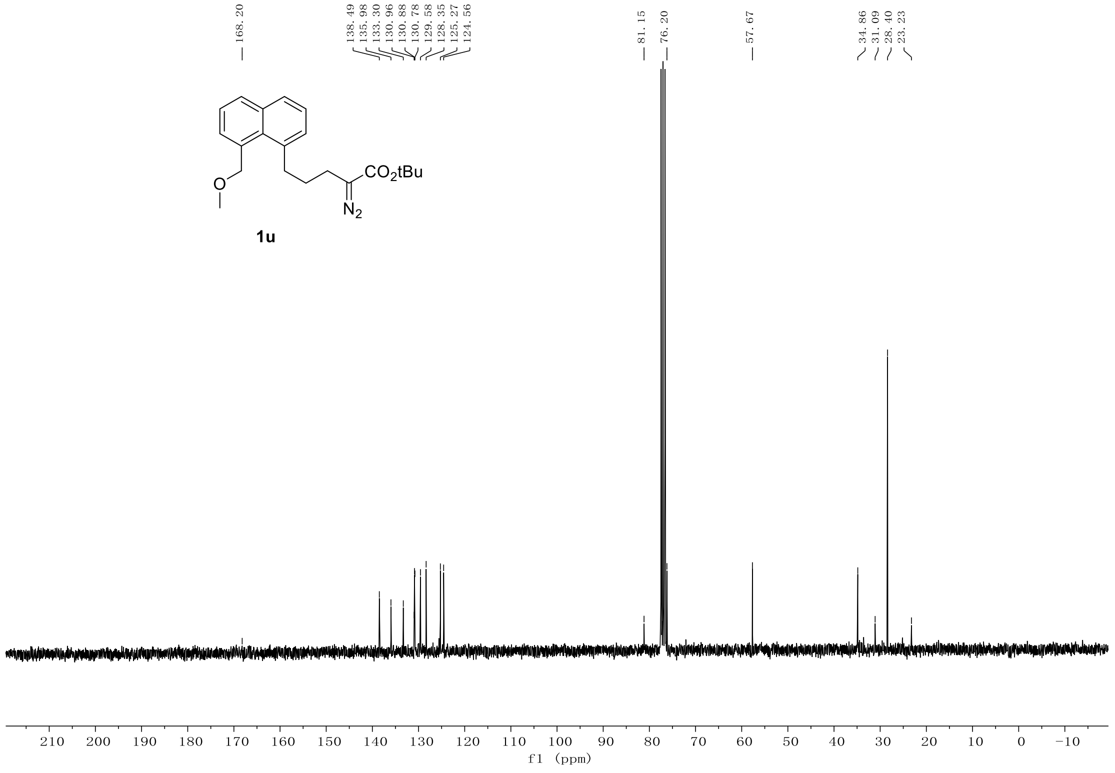

168.20
138.49
135.98
133.30
130.96
130.88
130.78
129.58
128.35
125.27
124.56
81.15
76.20
57.67
34.86
31.09
28.40
23.23
CO2tBu
N2
1u
f1 (ppm)

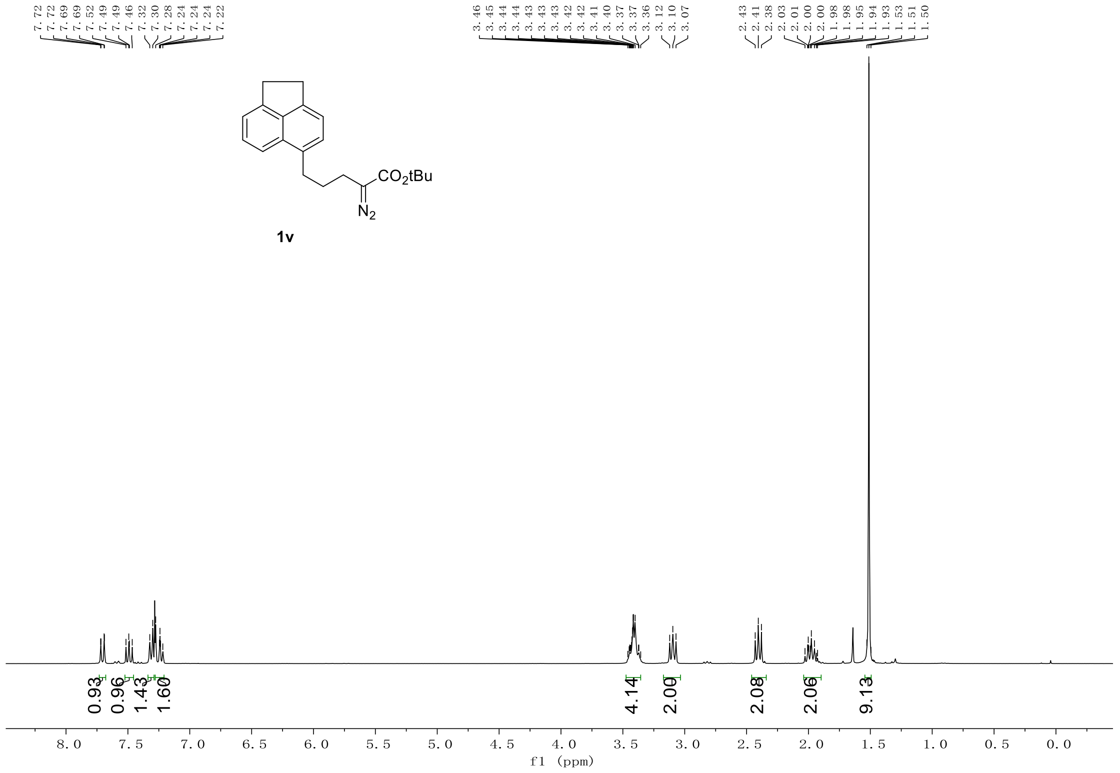
7.72 7.72 7.69 7.69 7.52 7.49 7.49 7.46 7.32 7.30 7.28 7.24 7.24 7.24 7.24 7.22
3.46 3.45 3.44 3.43 3.43 3.43 3.42 3.41 3.40 3.37 3.37 3.36 3.12 3.10 3.07
2.43 2.41 2.38 2.03 2.01 2.00 2.00 1.98 1.98 1.95 1.94 1.93 1.53 1.51 1.50
CO2tBu
N2
1v
0.93 0.96 1.43 1.60 4.14 2.00 2.08 2.06 9.13
8.0 7.5 7.0 6.5 6.0 5.5 5.0 4.5 4.0 3.5 3.0 2.5 2.0 1.5 1.0 0.5 0.0
f1 (ppm)

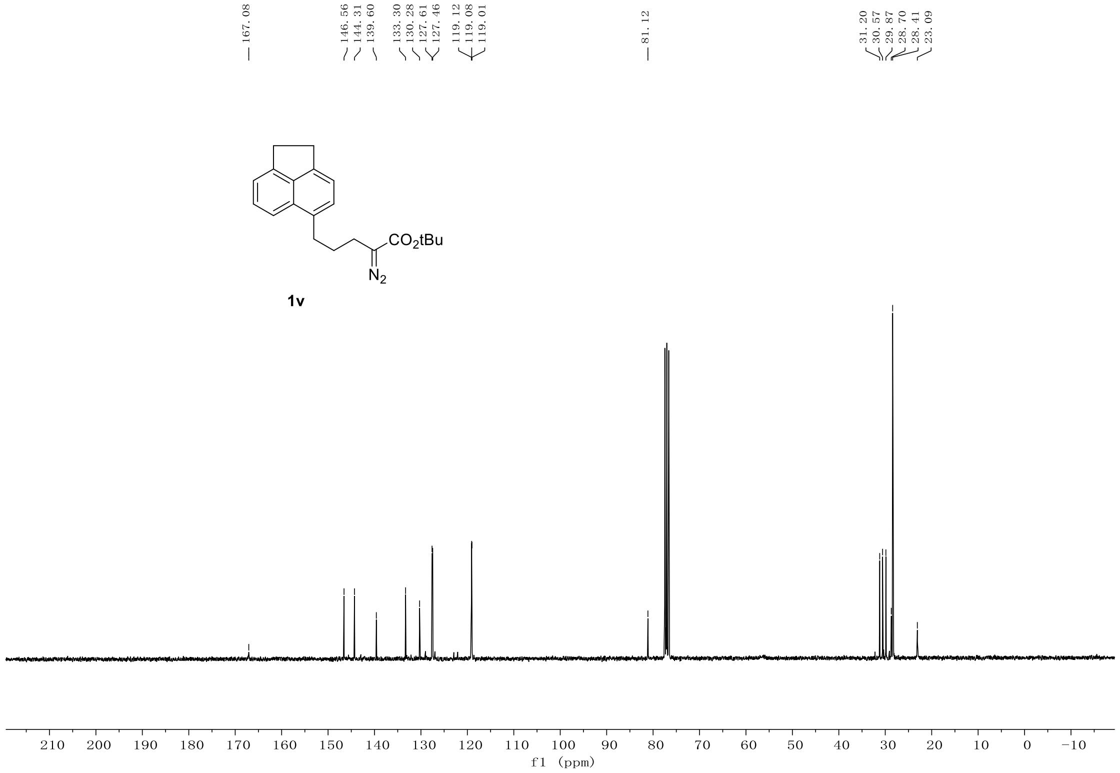
167.08
146.56 144.31 139.60 133.30 130.28 127.61 127.46 119.12 119.08 119.01
81.12
31.20 30.57 29.87 28.70 28.41 23.09
CO2tBu
N2
1v
210 200 190 180 170 160 150 140 130 120 110 100 90 80 70 60 50 40 30 20 10 0 -10
f1 (ppm)

NMR Spectrum for product

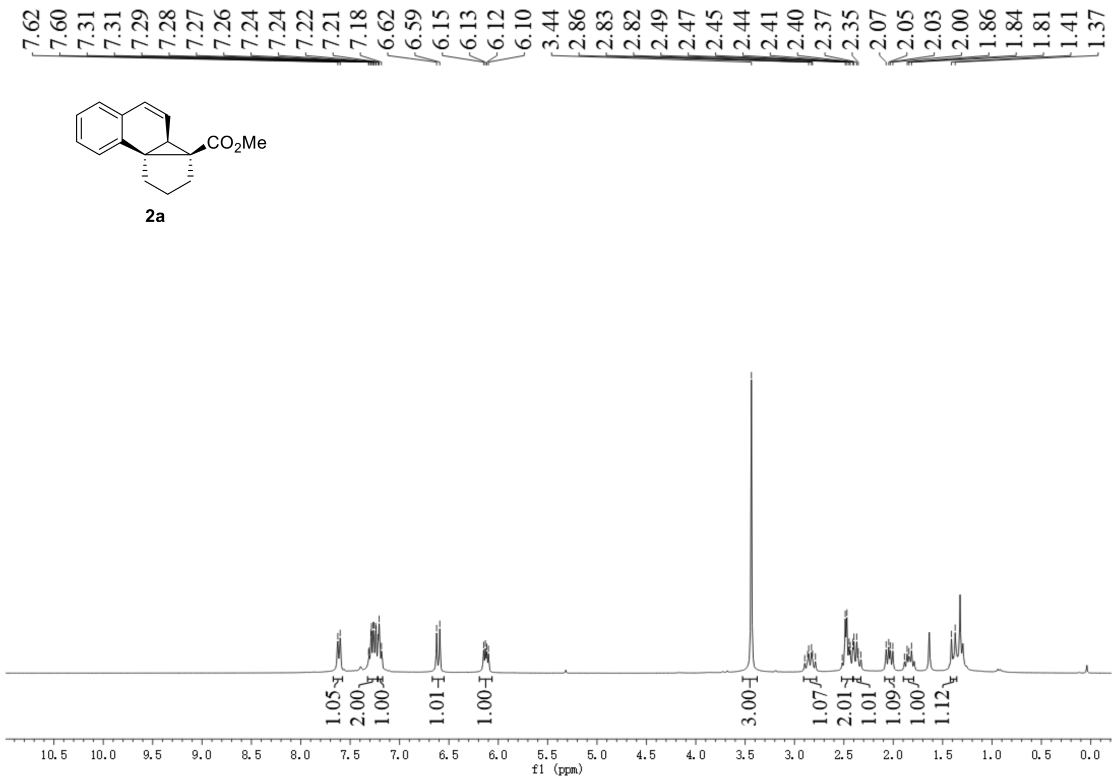

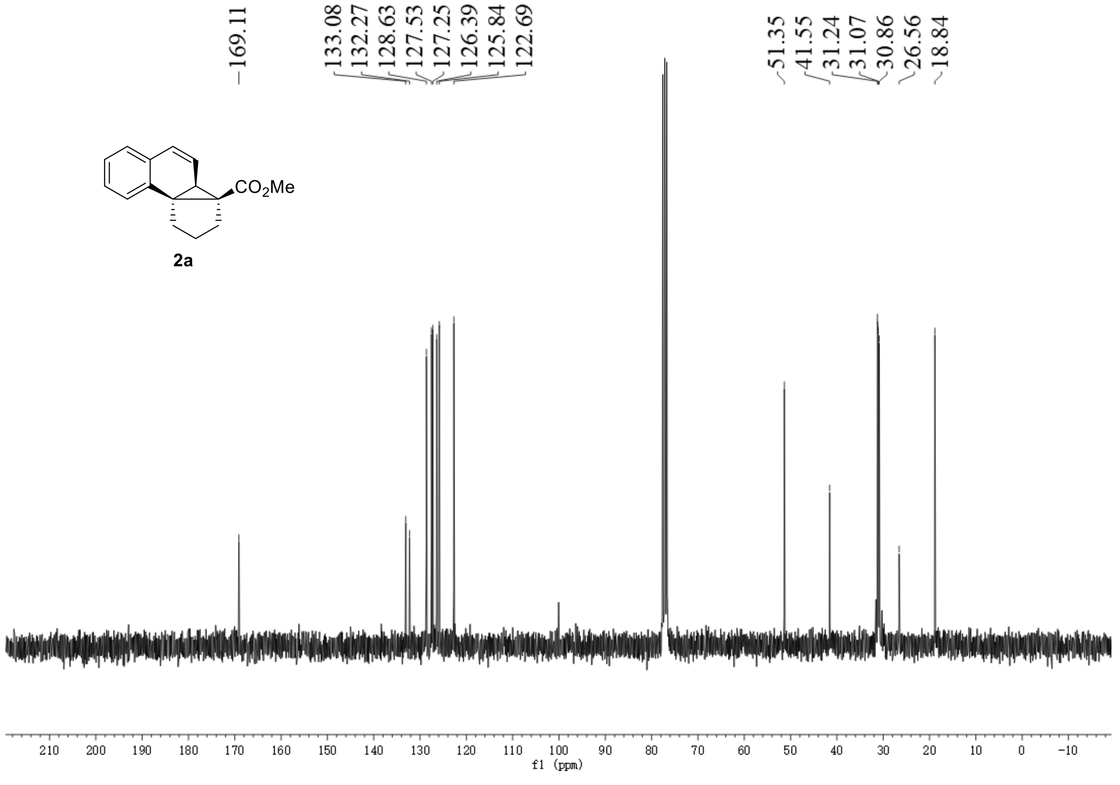

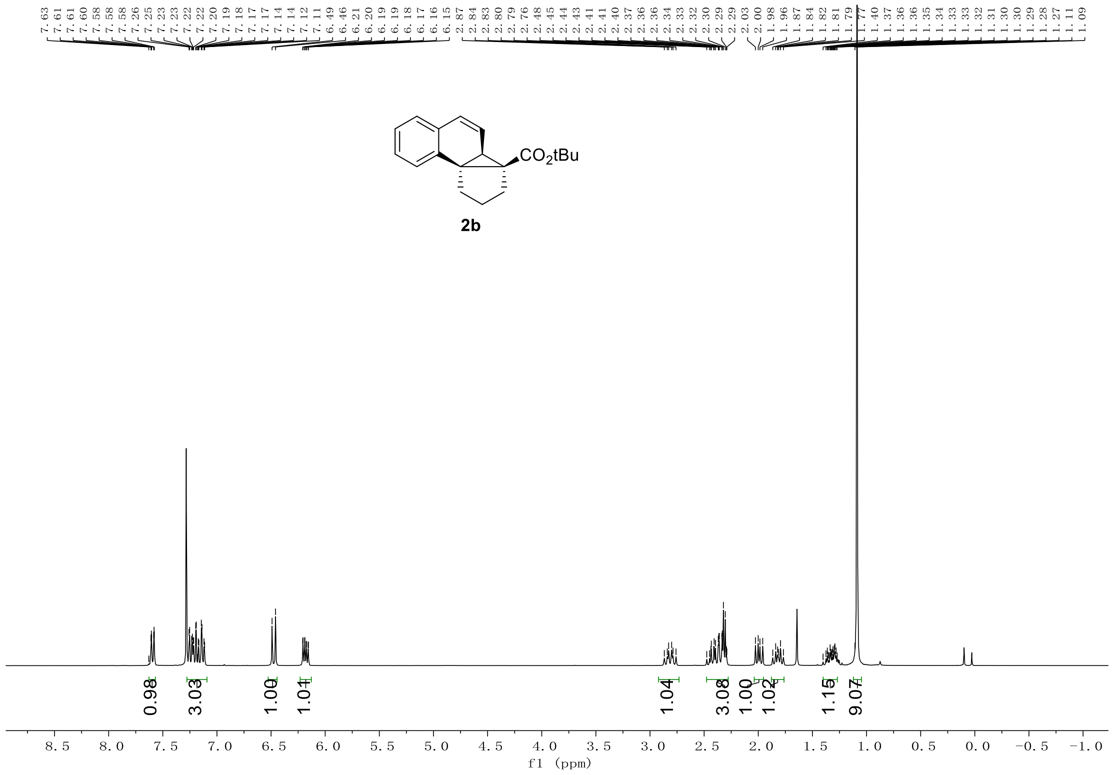
7.63 7.61 7.61 7.60 7.58 7.58 7.58 7.26 7.25 7.23 7.23 7.22 7.20 7.19 7.18 7.17 7.17 7.14 7.14 7.12 7.11 6.49 6.46 6.21 6.20 6.19 6.19 6.18 6.17 6.16 6.15 2.87 2.84 2.83 2.80 2.79 2.76 2.48 2.45 2.44 2.43 2.41 2.41 2.40 2.37 2.36 2.34 2.33 2.32 2.30 2.29 2.29 2.03 2.00 1.98 1.96 1.87 1.84 1.82 1.81 1.79 1.77 1.40 1.37 1.36 1.36 1.35 1.34 1.34 1.33 1.33 1.32 1.31 1.30 1.30 1.29 1.28 1.27 1.11 1.09
CO2tBu
2b
0.98 3.03 1.00 1.01 1.04 3.08 1.00 1.02 1.15 9.07
8.5 8.0 7.5 7.0 6.5 6.0 5.5 5.0 4.5 4.0 3.5 3.0 2.5 2.0 1.5 1.0 0.5 0.0 −0.5 −1.0
f1 (ppm)

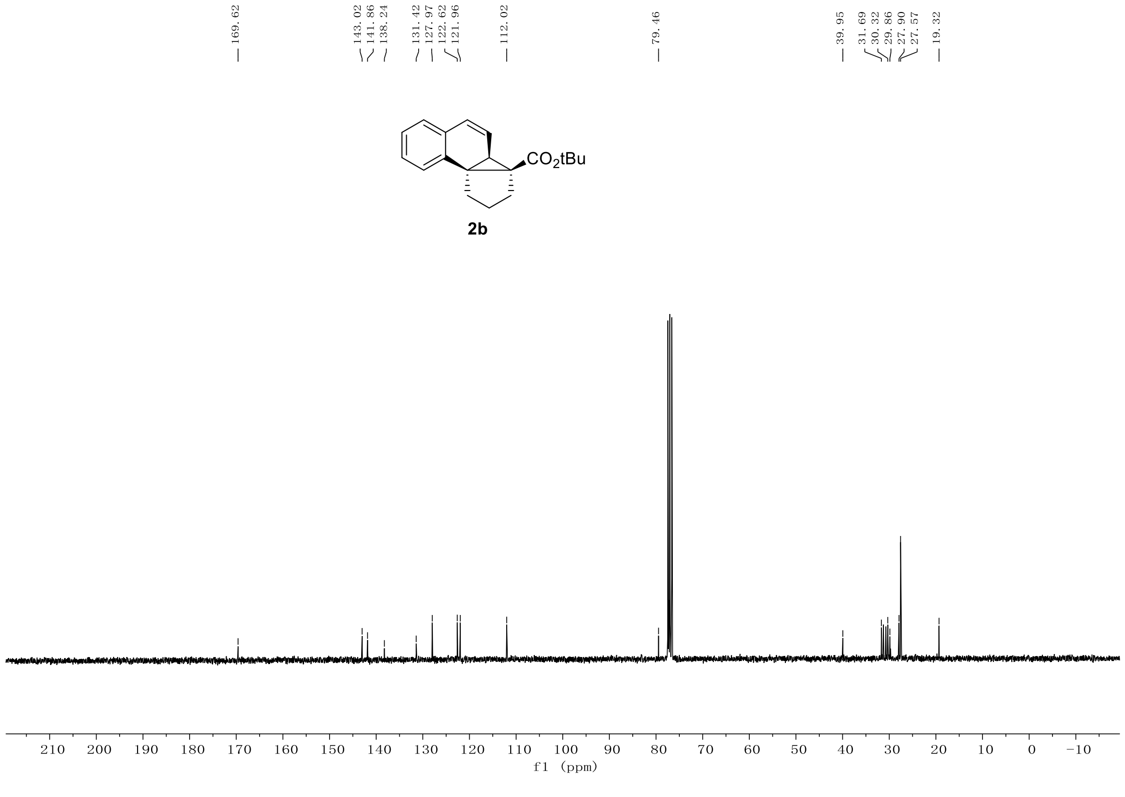
169.62
143.02 141.86 138.24
131.42 127.97 122.62 121.96
112.02
79.46
39.95
31.69 30.32 29.86 27.90 27.57
19.32
CO2tBu
2b
210 200 190 180 170 160 150 140 130 120 110 100 90 80 70 60 50 40 30 20 10 0 −10
f1 (ppm)

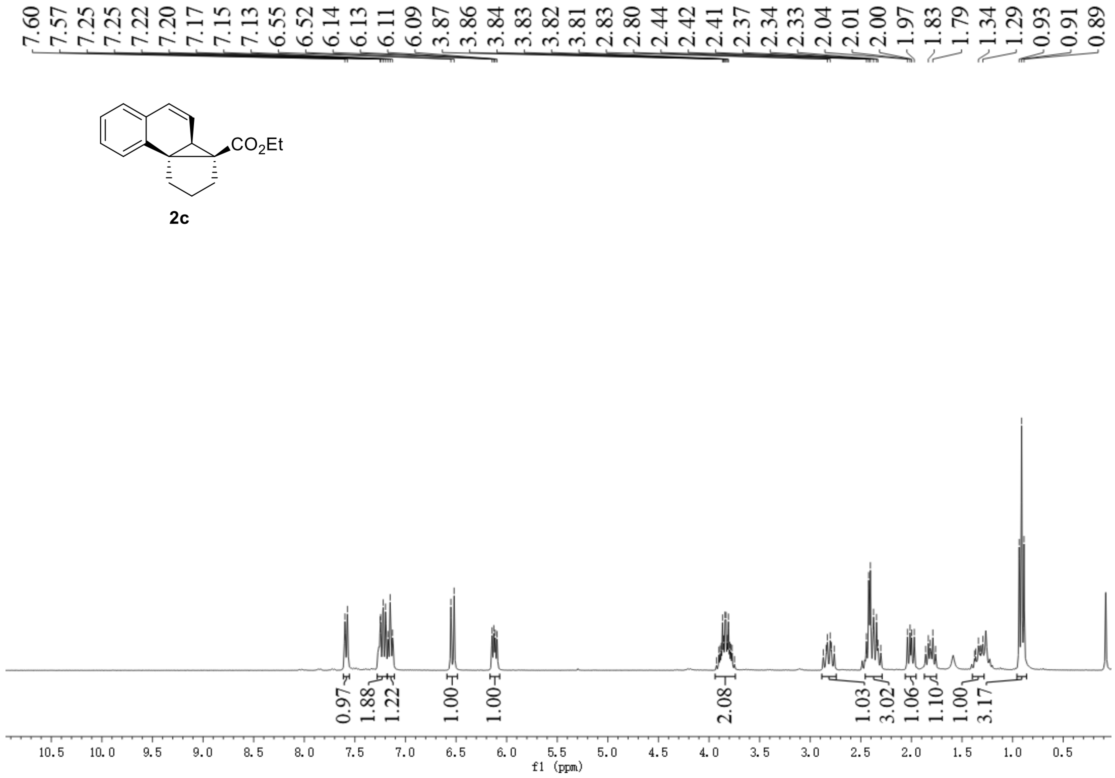
7.60
7.57
7.25
7.25
7.22
7.20
7.17
7.15
7.13
6.55
6.52
6.14
6.13
6.11
6.09
3.87
3.86
3.84
3.83
3.82
3.81
2.83
2.80
2.44
2.42
2.41
2.37
2.34
2.33
2.04
2.01
2.00
1.97
1.83
1.79
1.34
1.29
0.93
0.91
0.89
CO2Et
2c
0.97
1.88
1.22
1.00
1.00
2.08
1.03
3.02
1.06
1.10
1.00
3.17
f1 (ppm)

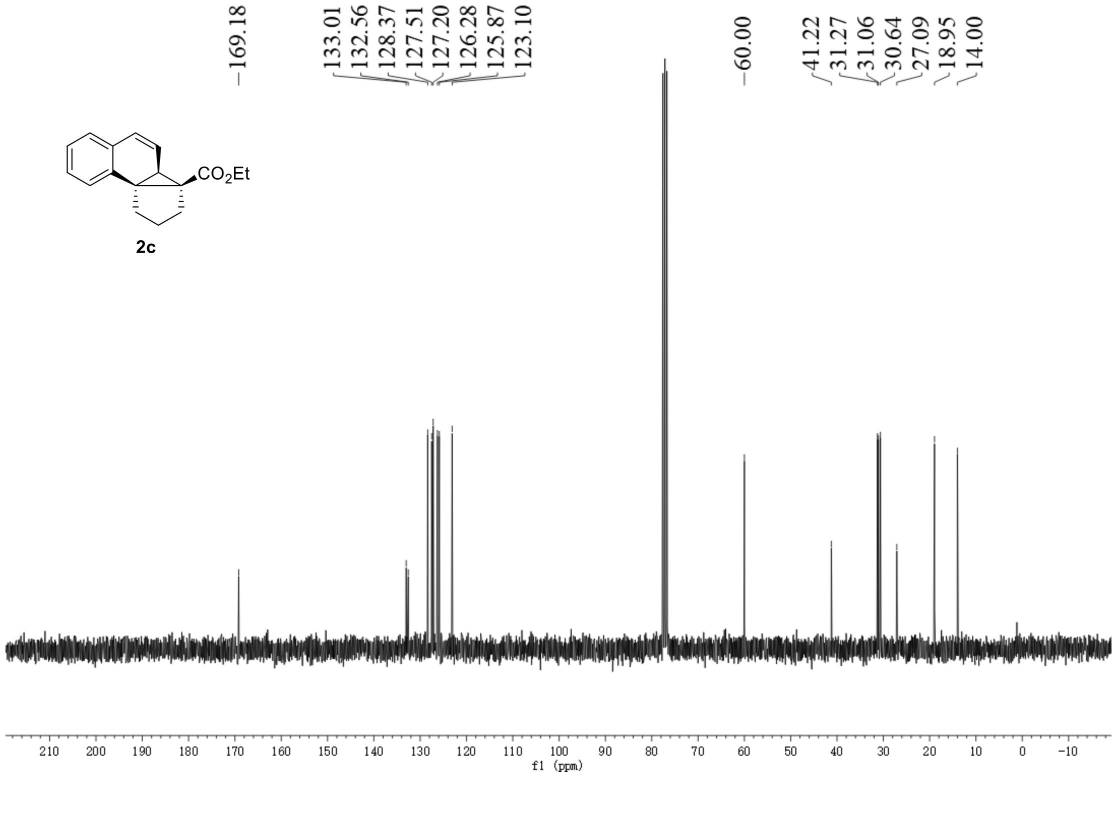
169.18
133.01
132.56
128.37
127.51
127.20
126.28
125.87
123.10
60.00
41.22
31.27
31.06
30.64
27.09
18.95
14.00
CO2Et
2c
f1 (ppm)

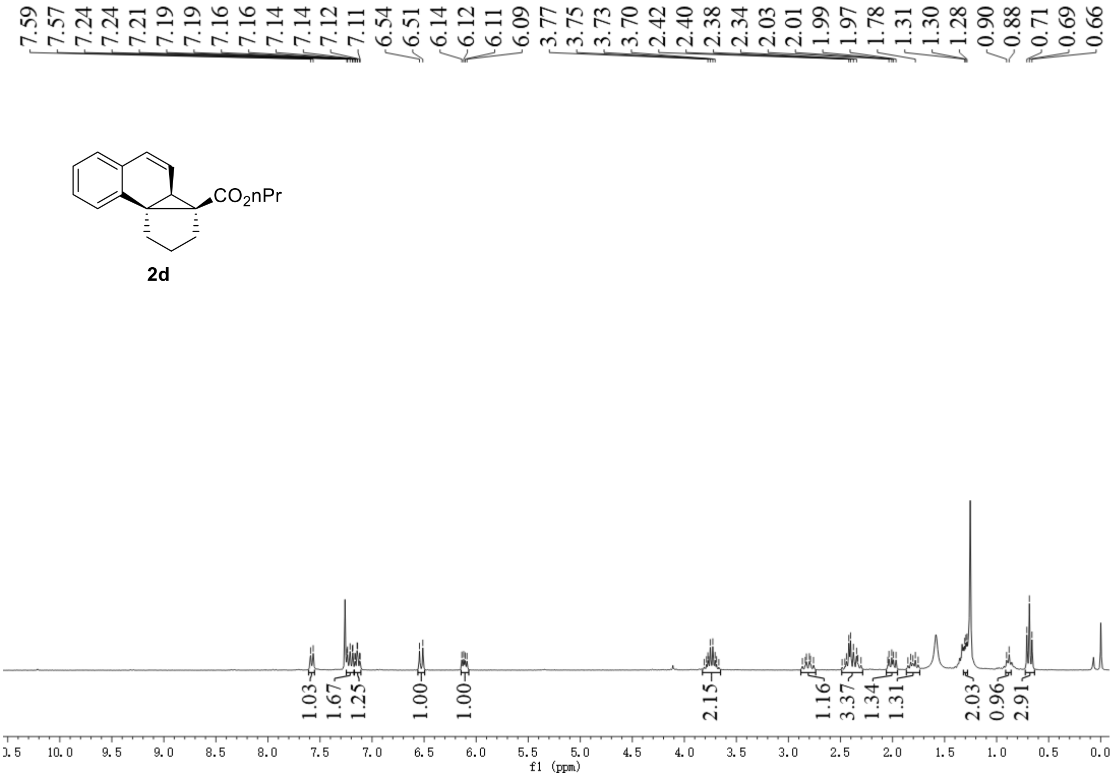
7.59 7.57 7.24 7.24 7.21 7.19 7.19 7.16 7.16 7.14 7.14 7.12 7.11 6.54 6.51 6.14 6.12 6.11 6.09 3.77 3.75 3.73 3.70 2.42 2.40 2.38 2.34 2.03 2.01 1.99 1.97 1.78 1.31 1.30 1.28 0.90 0.88 0.71 0.69 0.66
CO2nPr
2d
1.03 1.67 1.25 1.00 1.00 2.15 1.16 3.37 1.34 1.31 2.03 0.96 2.91
f1 (ppm)

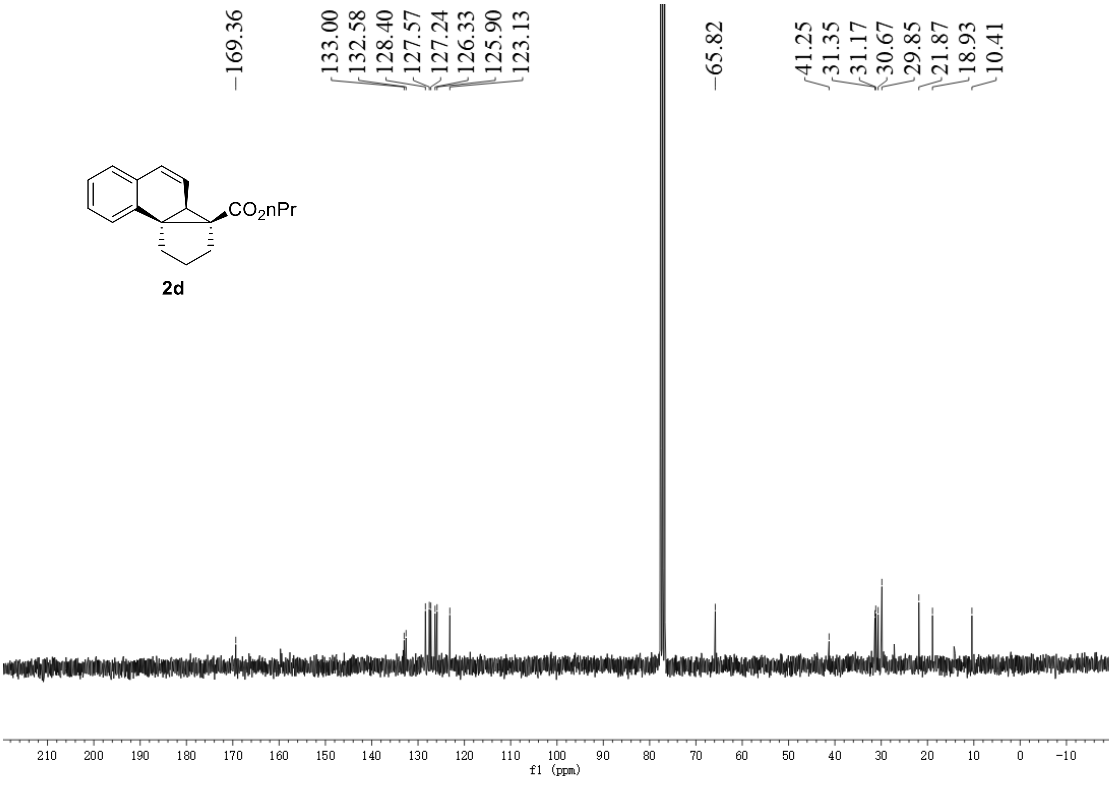
169.36
133.00 132.58 128.40 127.57 127.24 126.33 125.90 123.13
65.82
41.25 31.35 31.17 30.67 29.85 21.87 18.93 10.41
CO2nPr
2d
f1 (ppm)

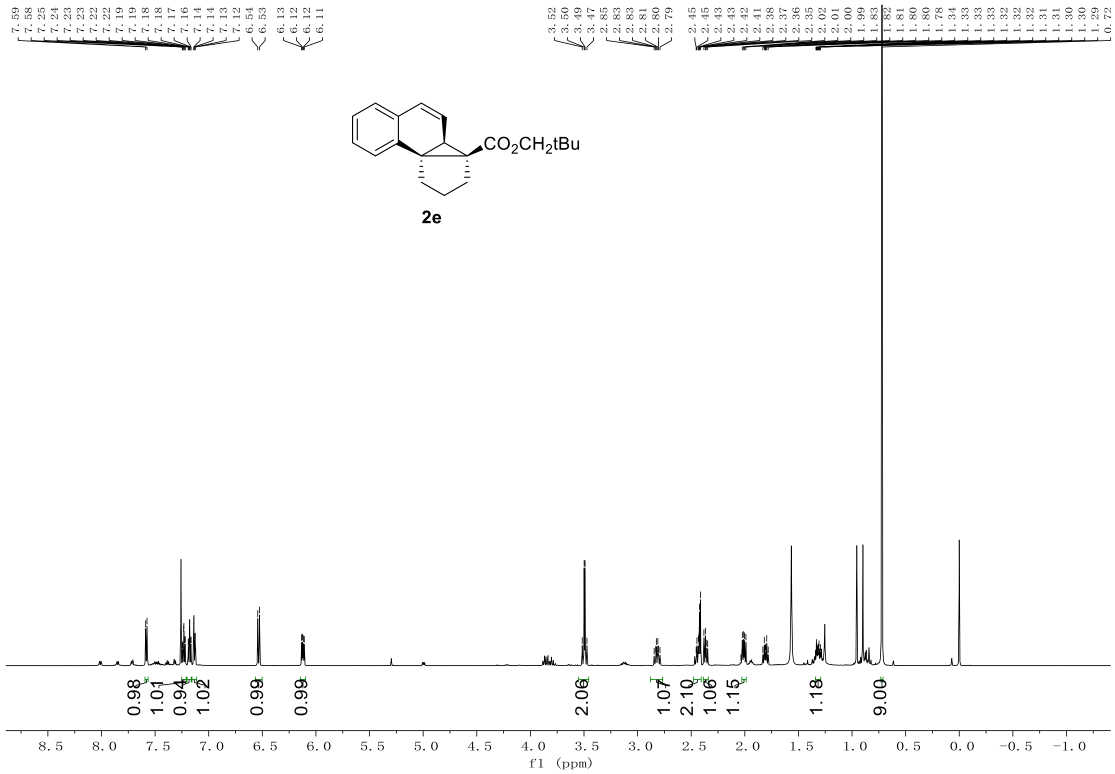
7.59 7.58 7.25 7.24 7.23 7.23 7.22 7.22 7.19 7.18 7.18 7.17 7.16 7.14 7.14 7.13 7.12 6.54 6.53 6.13 6.12 6.12 6.11
3.52 3.50 3.49 3.47 2.85 2.83 2.83 2.81 2.80 2.79 2.45 2.45 2.43 2.43 2.42 2.41 2.38 2.37 2.36 2.35 2.02 2.01 2.00 1.99 1.83 1.82 1.81 1.80 1.80 1.78 1.34 1.33 1.33 1.33 1.32 1.32 1.31 1.30 1.29 0.72
CO2CH2tBu
2e
0.98 1.01 0.94 1.02 0.99 0.99 2.06 1.07 2.10 1.06 1.15 1.18 9.00
8.5 8.0 7.5 7.0 6.5 6.0 5.5 5.0 4.5 4.0 3.5 3.0 2.5 2.0 1.5 1.0 0.5 0.0 −0.5 −1.0
f1 (ppm)

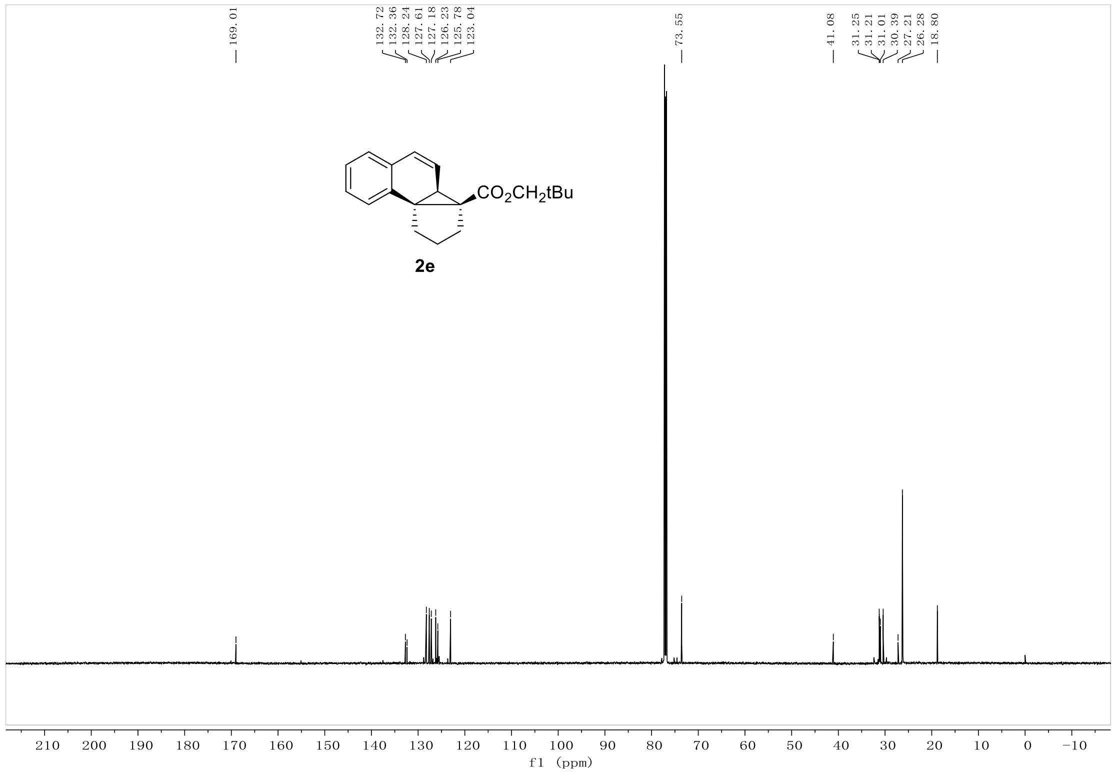
169.01
132.72 132.36 128.24 127.61 127.18 126.23 125.78 123.04
73.55
41.08
31.25 31.21 31.01 30.39 27.21 26.28 18.80
CO2CH2tBu
2e
210 200 190 180 170 160 150 140 130 120 110 100 90 80 70 60 50 40 30 20 10 0 −10
f1 (ppm)

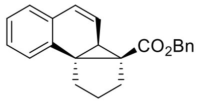

2f

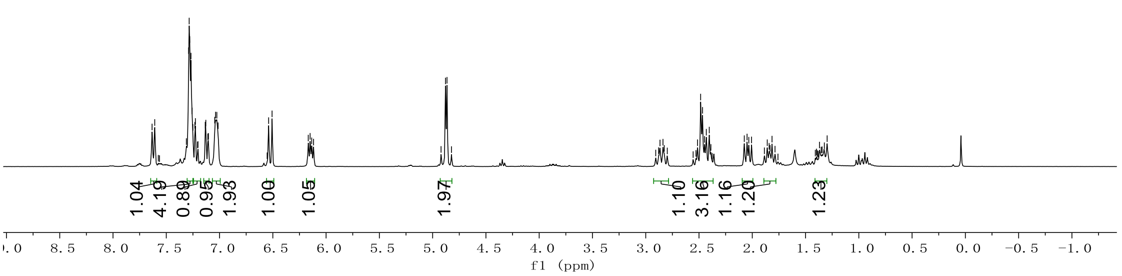
7.63 7.61 7.57 7.56 7.31 7.29 7.29 7.28 7.27 7.26 7.25 7.23 7.23 7.21 7.20 7.13 7.13 7.11 7.10 7.05 7.04 7.03 7.01 6.55 6.54 6.51 6.17 6.15 6.14 6.12 4.92 4.88 4.87 4.82
2.91 2.88 2.87 2.84 2.83 2.80 2.56 2.53 2.51 2.48 2.48 2.47 2.45 2.43 2.40 2.39 2.38 2.07 2.05 2.03 2.01 1.89 1.86 1.84 1.83 1.81 1.79 1.76 1.41 1.40 1.39 1.38 1.37 1.35 1.34 1.32 1.30
1.04 4.19 0.89 0.95 1.93 1.00 1.05 1.97 1.10 3.16 1.16 1.20 1.23
f1 (ppm)

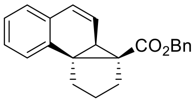

2f

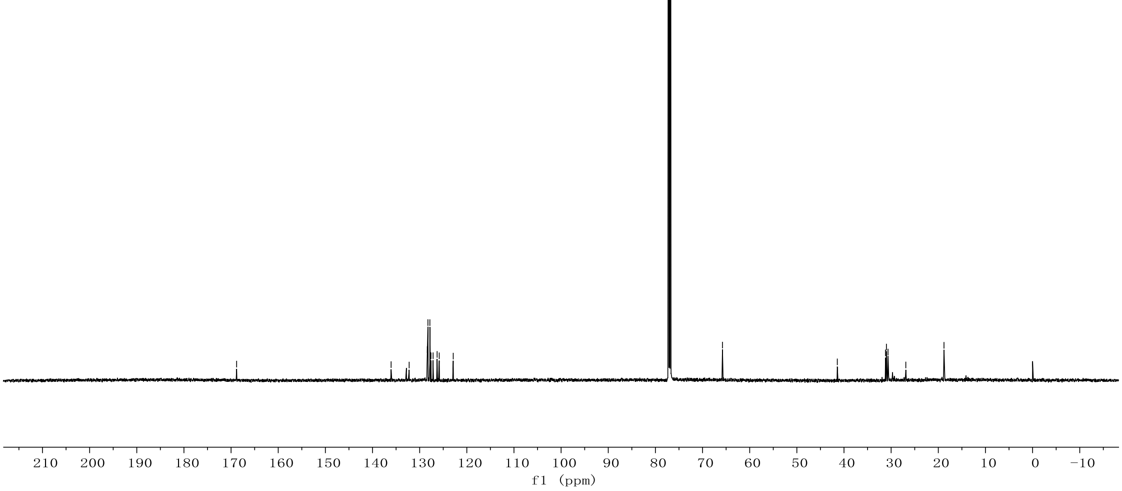
168.82
136.05 132.23 128.39 128.25 127.84 127.74 127.60 127.15 126.30 125.85 122.88
65.77
41.42
31.20 31.01 30.66 26.89
18.79
f1 (ppm)

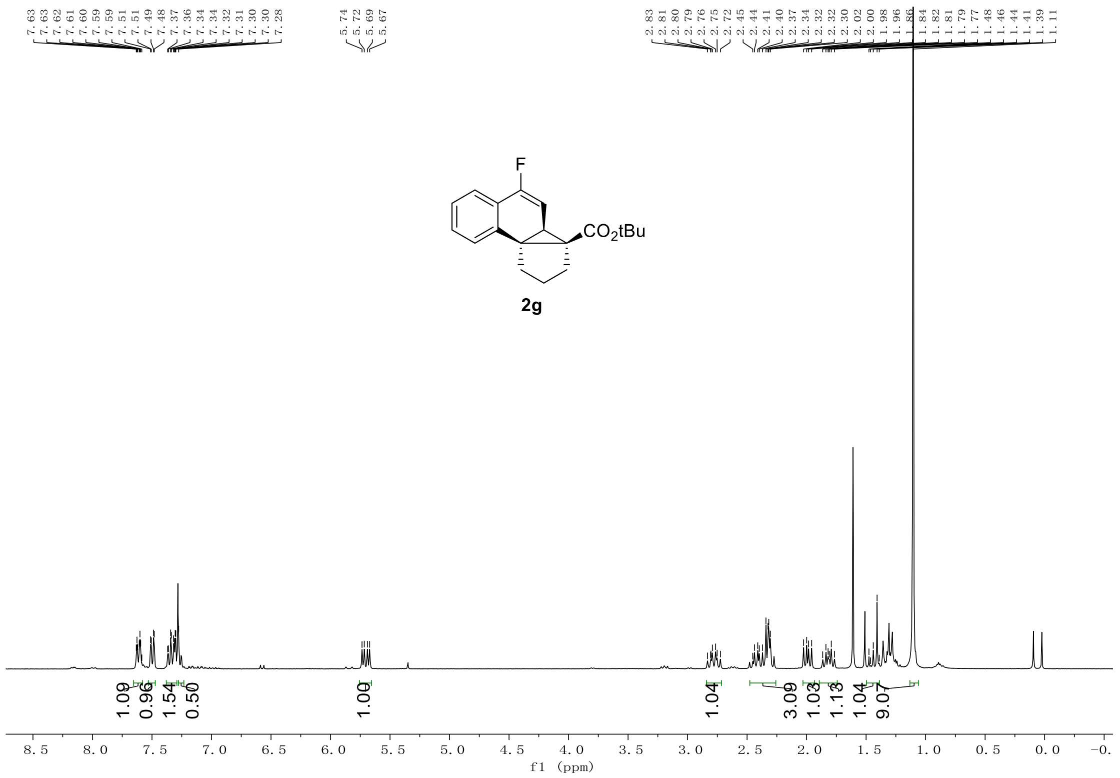
7.63 7.63 7.62 7.61 7.60 7.59 7.59 7.51 7.51 7.49 7.48 7.37 7.36 7.34 7.32 7.31 7.30 7.30 7.28
5.74 5.72 5.69 5.67
2.83 2.81 2.80 2.79 2.76 2.75 2.72 2.45 2.44 2.41 2.40 2.37 2.34 2.32 2.32 2.30 2.02 2.00 1.98 1.96 1.86 1.84 1.82 1.81 1.79 1.77 1.48 1.46 1.44 1.41 1.39 1.11
F
CO2tBu
2g
1.09
0.96
1.54
0.50
1.00
1.04
3.09
1.03
1.13
1.04
9.07
f1 (ppm)

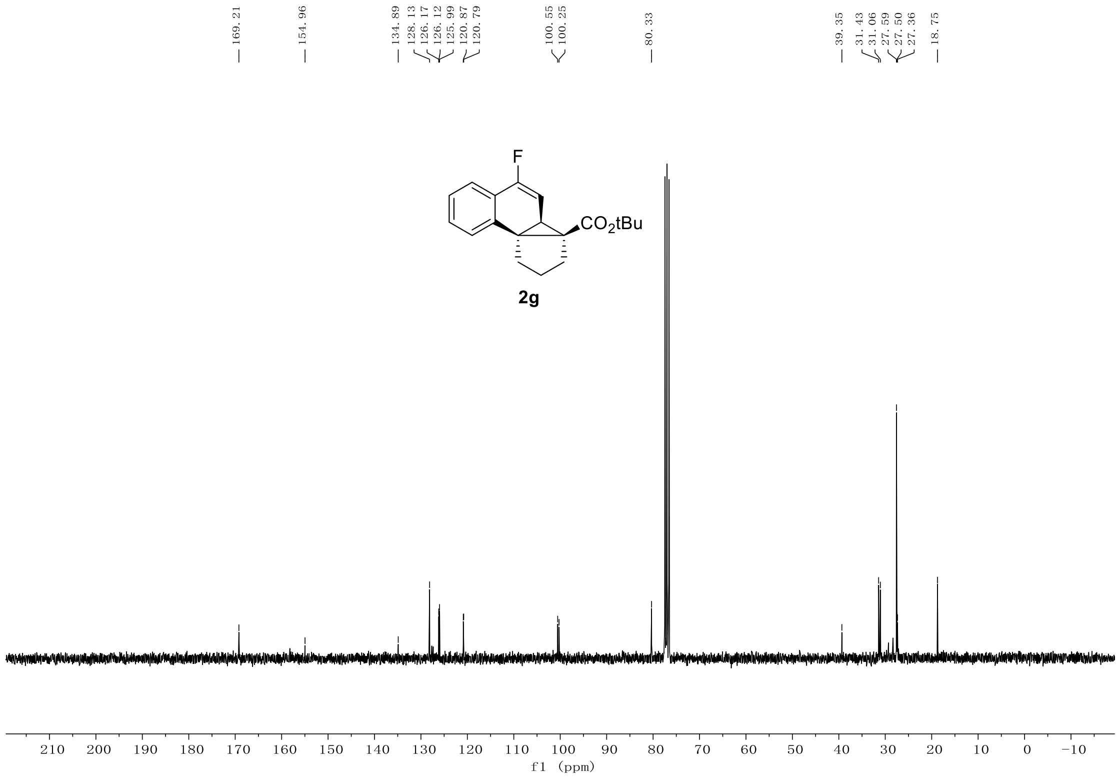
169.21
154.96
134.89 128.13 126.17 126.12 125.99 120.87 120.79
100.55 100.25
80.33
39.35
31.43 31.06 27.59 27.50 27.36
18.75
F
CO2tBu
2g
f1 (ppm)

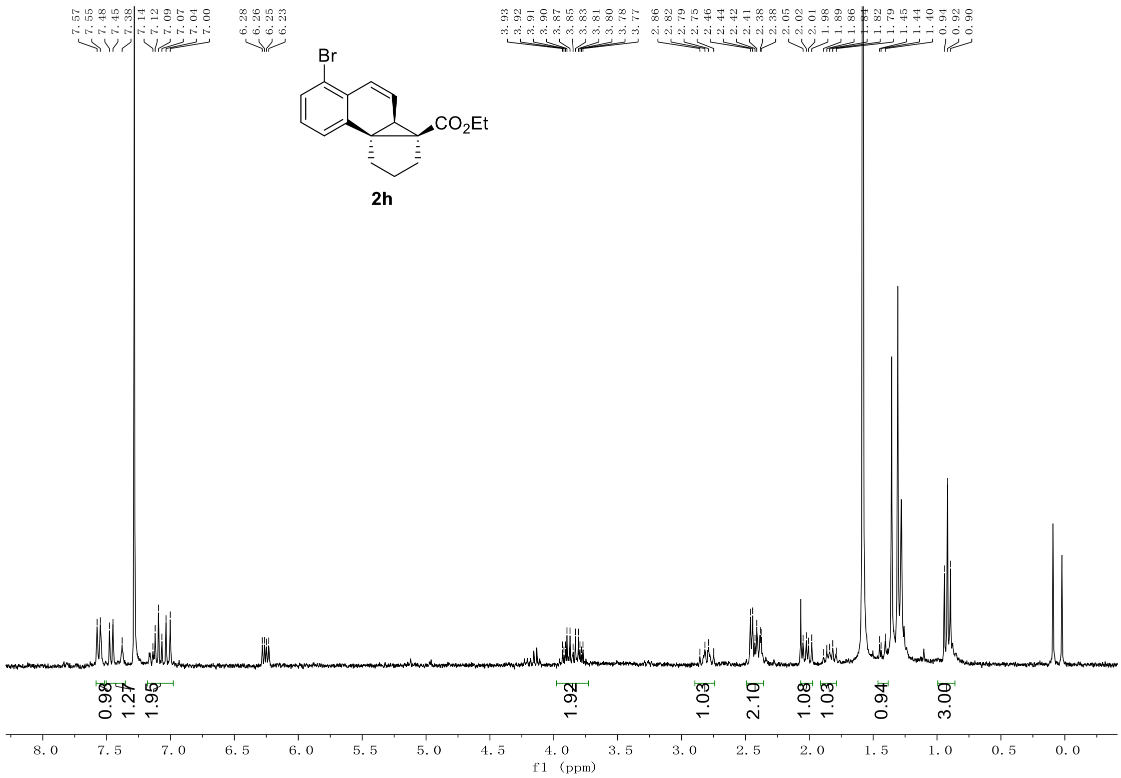
Br
CO2Et
2h
f1 (ppm)

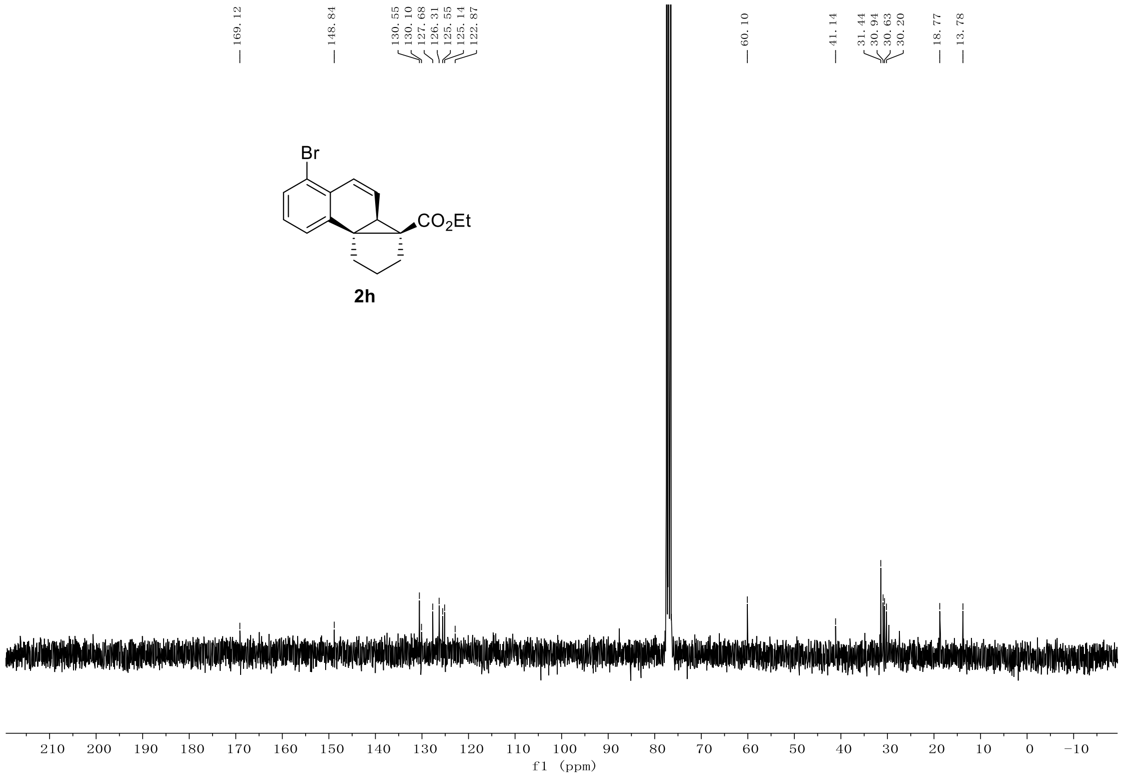
Br
CO2Et
2h
f1 (ppm)

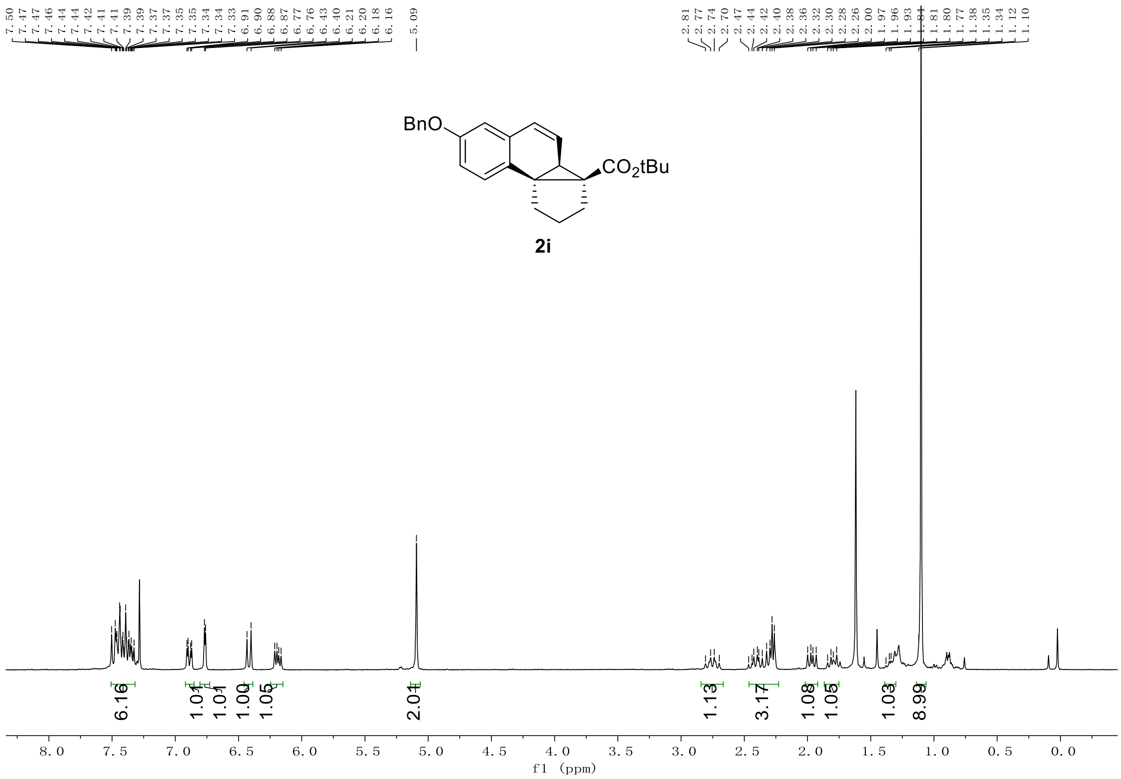
7.50 7.47 7.46 7.44 7.44 7.42 7.41 7.39 7.39 7.37 7.37 7.35 7.34 7.34 7.33 6.91 6.90 6.88 6.87 6.77 6.76 6.43 6.40 6.21 6.20 6.18 6.16
5.09
2.81 2.77 2.74 2.70 2.47 2.44 2.42 2.40 2.38 2.36 2.32 2.30 2.28 2.26 2.00 1.97 1.96 1.93 1.84 1.81 1.80 1.77 1.38 1.35 1.34 1.12 1.10
BnO
CO2tBu
2i
6.16 1.04 1.01 1.00 1.05 2.04 1.13 3.17 1.08 1.05 1.03 8.99
8.0 7.5 7.0 6.5 6.0 5.5 5.0 4.5 4.0 3.5 3.0 2.5 2.0 1.5 1.0 0.5 0.0
f1 (ppm)

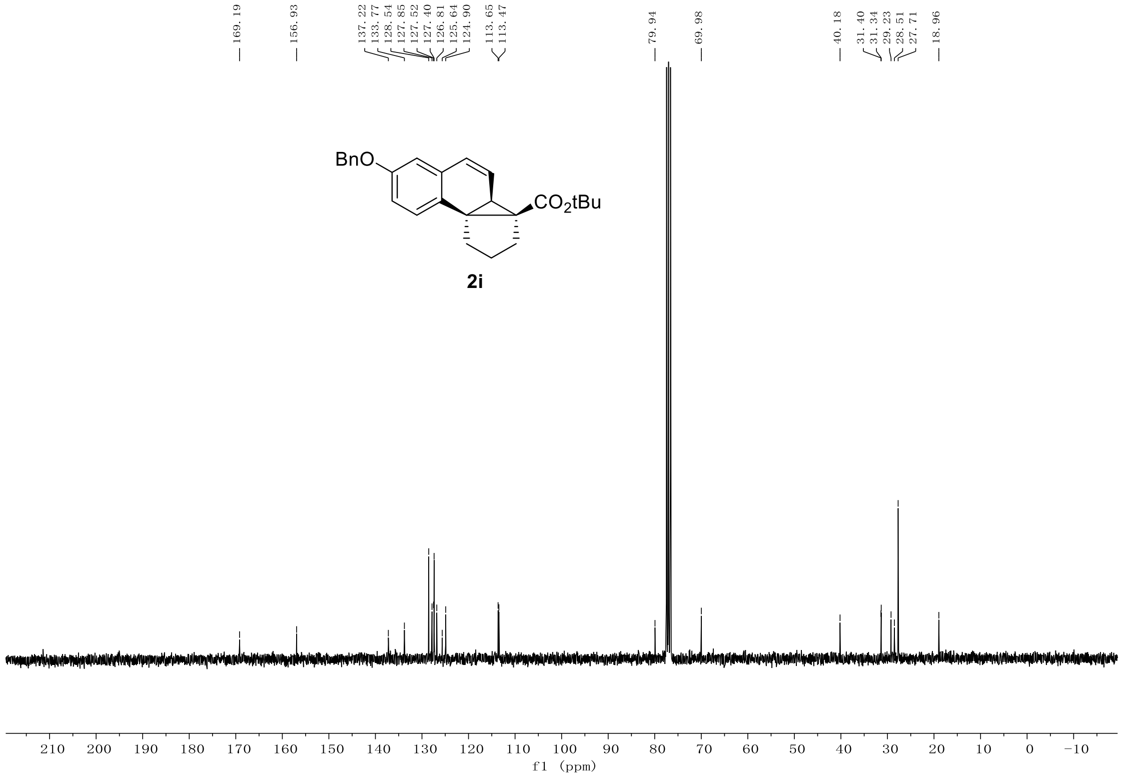
169.19
156.93
137.22 133.77 128.54 127.85 127.52 127.40 126.81 125.64 124.90
113.65 113.47
79.94
69.98
40.18 31.40 31.34 29.23 28.51 27.71
18.96
BnO
CO2tBu
2i
210 200 190 180 170 160 150 140 130 120 110 100 90 80 70 60 50 40 30 20 10 0 −10
f1 (ppm)

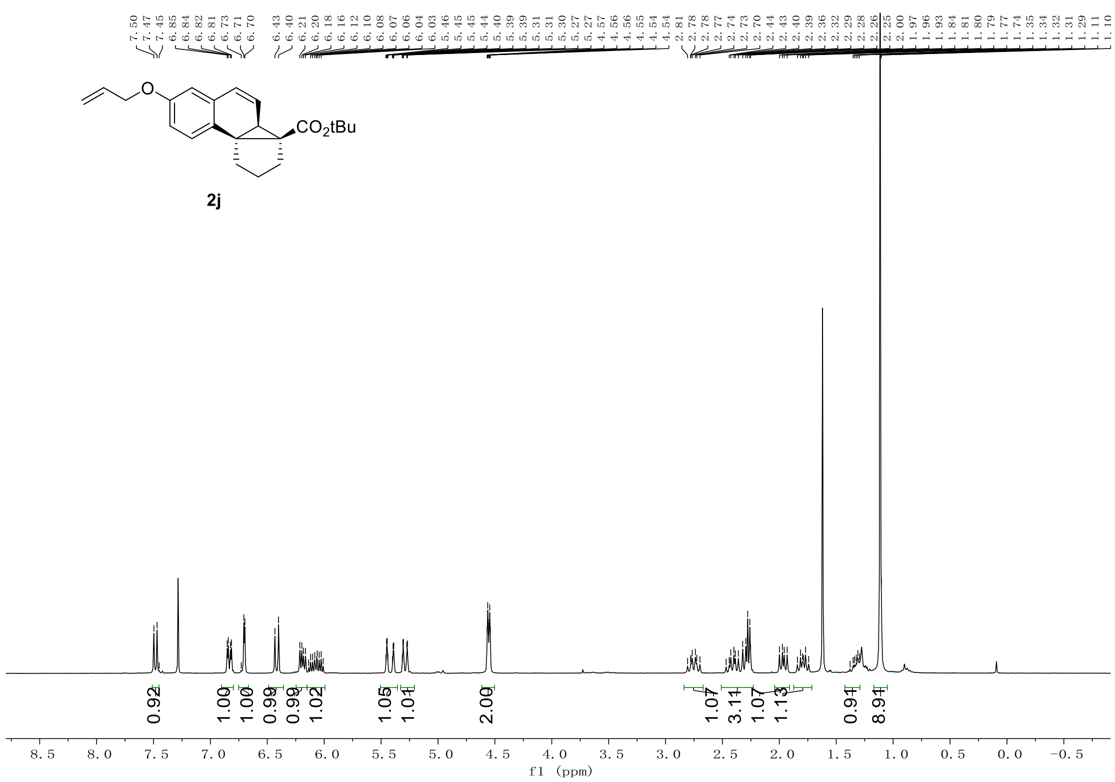
CO2tBu
2j
f1 (ppm)

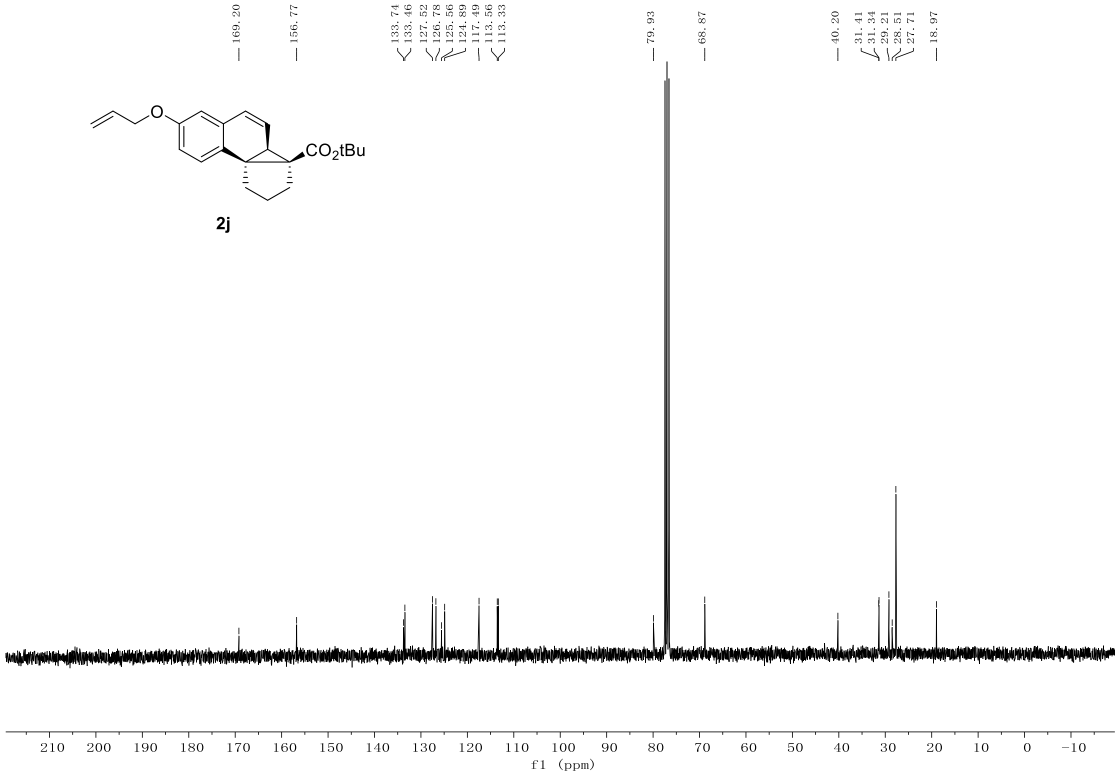
CO2tBu
2j
f1 (ppm)

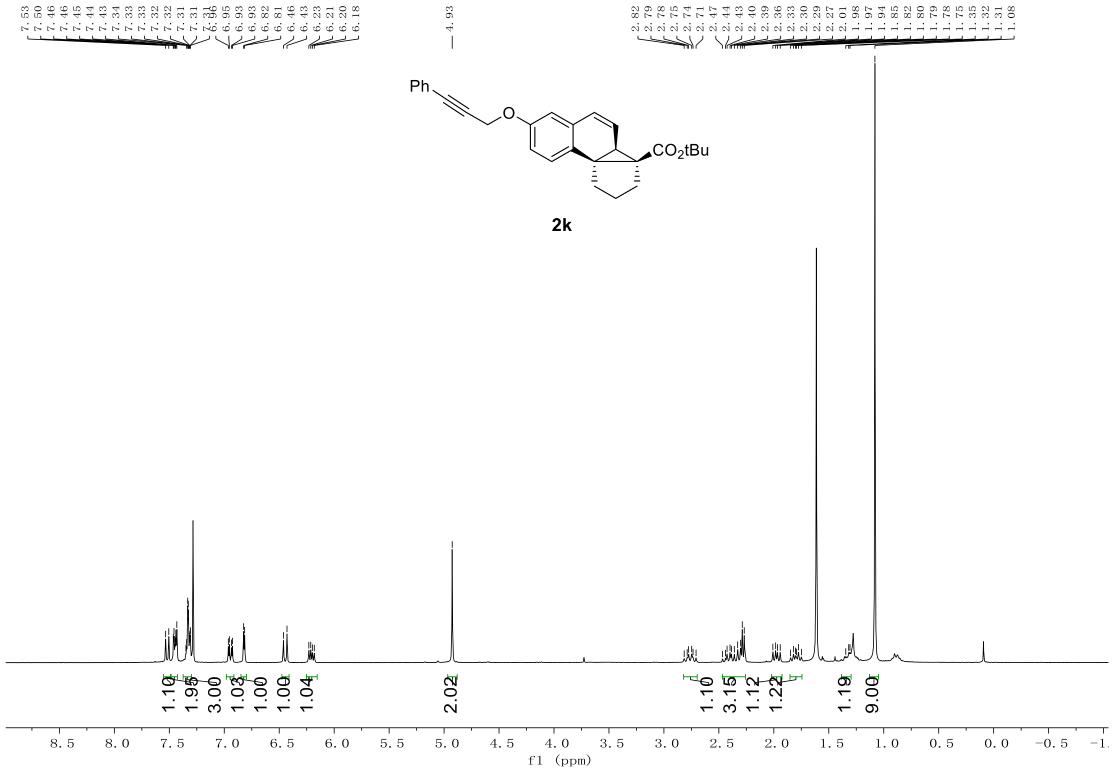

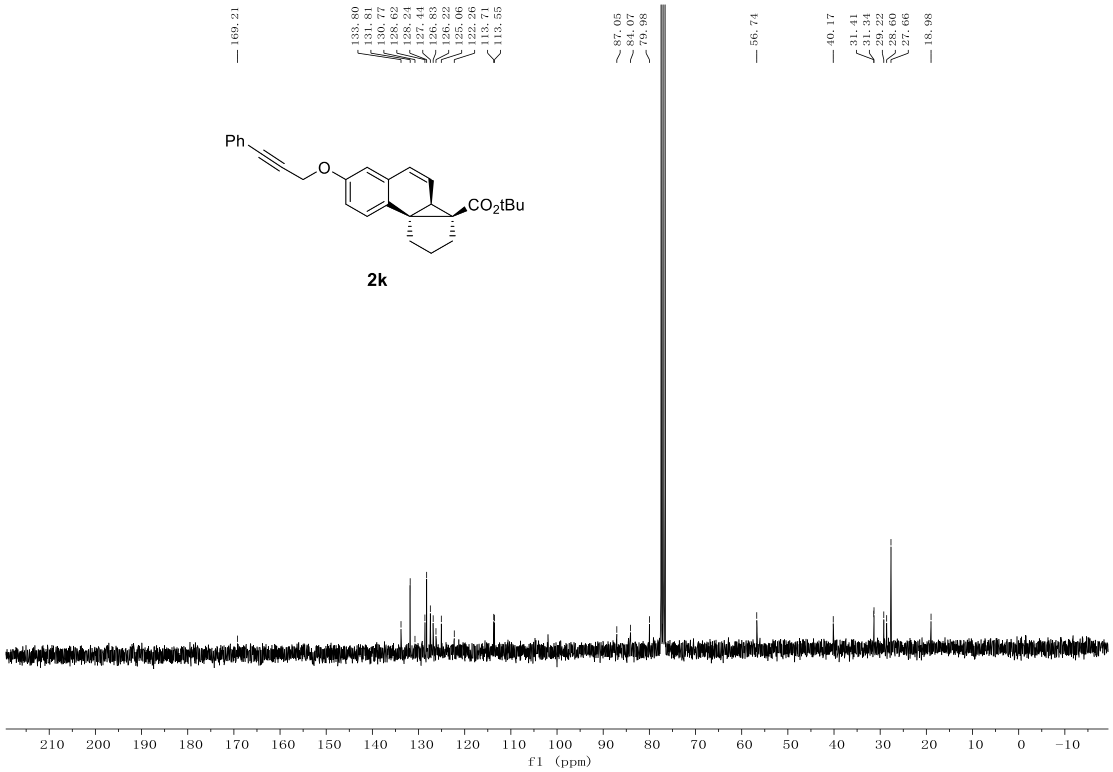

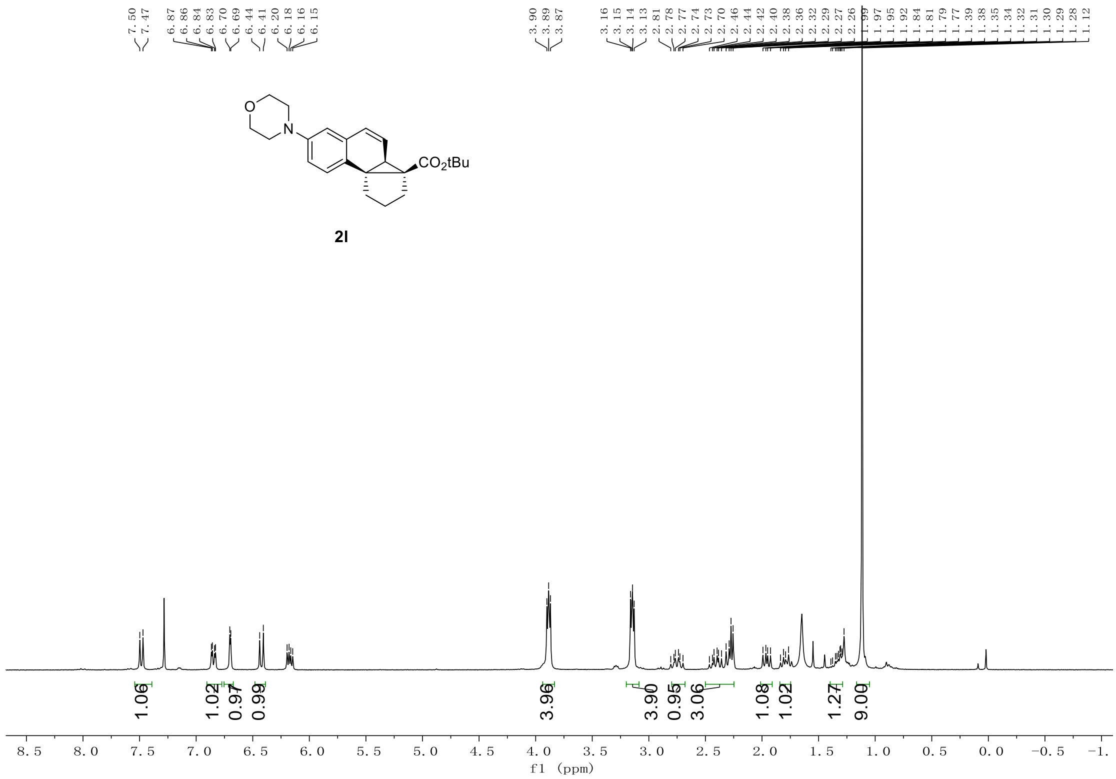
7.50
7.47
6.87
6.86
6.84
6.83
6.70
6.69
6.44
6.41
6.20
6.18
6.16
6.15
3.90
3.89
3.87
3.16
3.15
3.14
3.13
2.81
2.78
2.77
2.74
2.73
2.70
2.46
2.44
2.42
2.40
2.38
2.36
2.32
2.29
2.27
2.26
1.99
1.97
1.95
1.92
1.84
1.81
1.79
1.77
1.39
1.38
1.35
1.34
1.32
1.31
1.30
1.29
1.28
1.12
O
N
CO_2tBu
2l
1.06
1.02
0.97
0.99
3.96
3.90
0.95
3.06
1.08
1.02
1.27
9.00
8.5 8.0 7.5 7.0 6.5 6.0 5.5 5.0 4.5 4.0 3.5 3.0 2.5 2.0 1.5 1.0 0.5 0.0 −0.5 −1.
f1 (ppm)

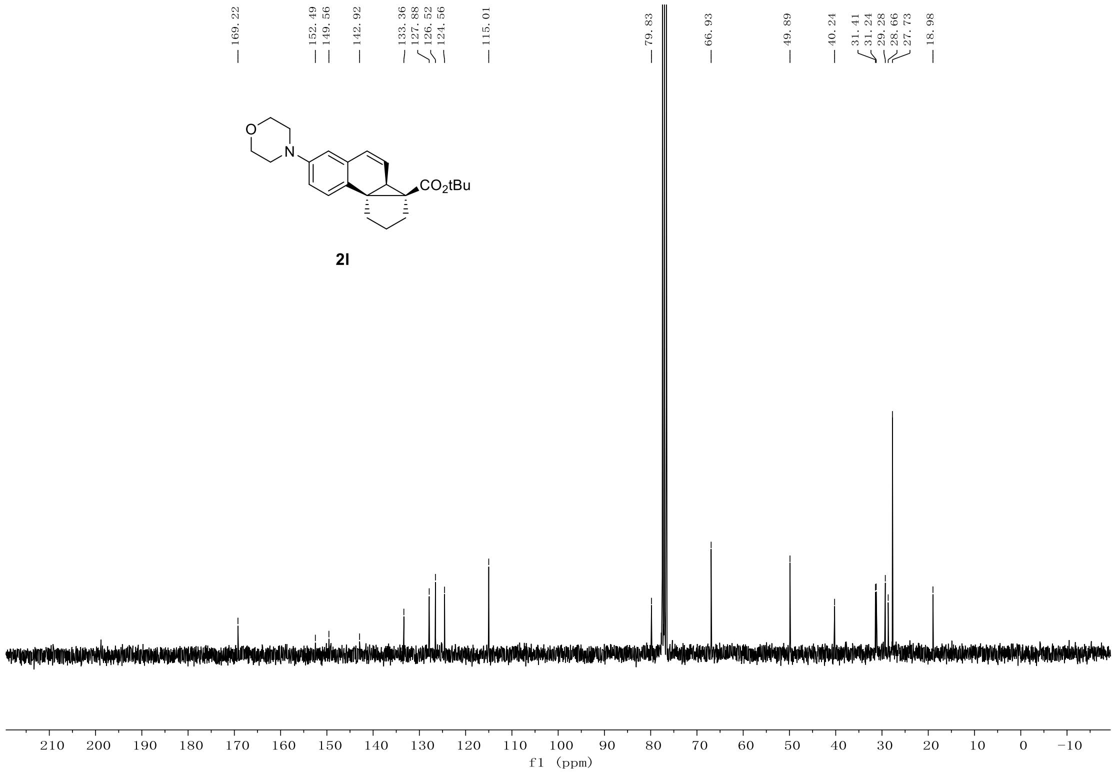
169.22
152.49
149.56
142.92
133.36
127.88
126.52
124.56
115.01
79.83
66.93
49.89
40.24
31.41
31.24
29.28
28.66
27.73
18.98
O
N
CO_2tBu
2l
210 200 190 180 170 160 150 140 130 120 110 100 90 80 70 60 50 40 30 20 10 0 −10
f1 (ppm)

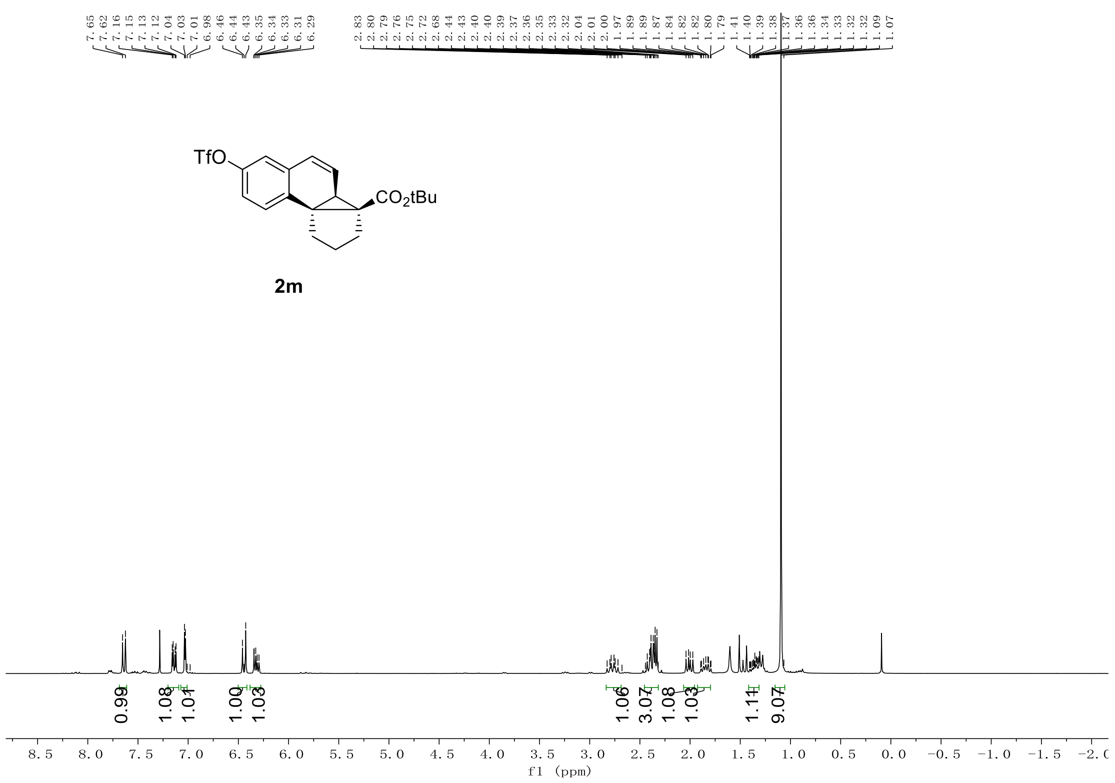

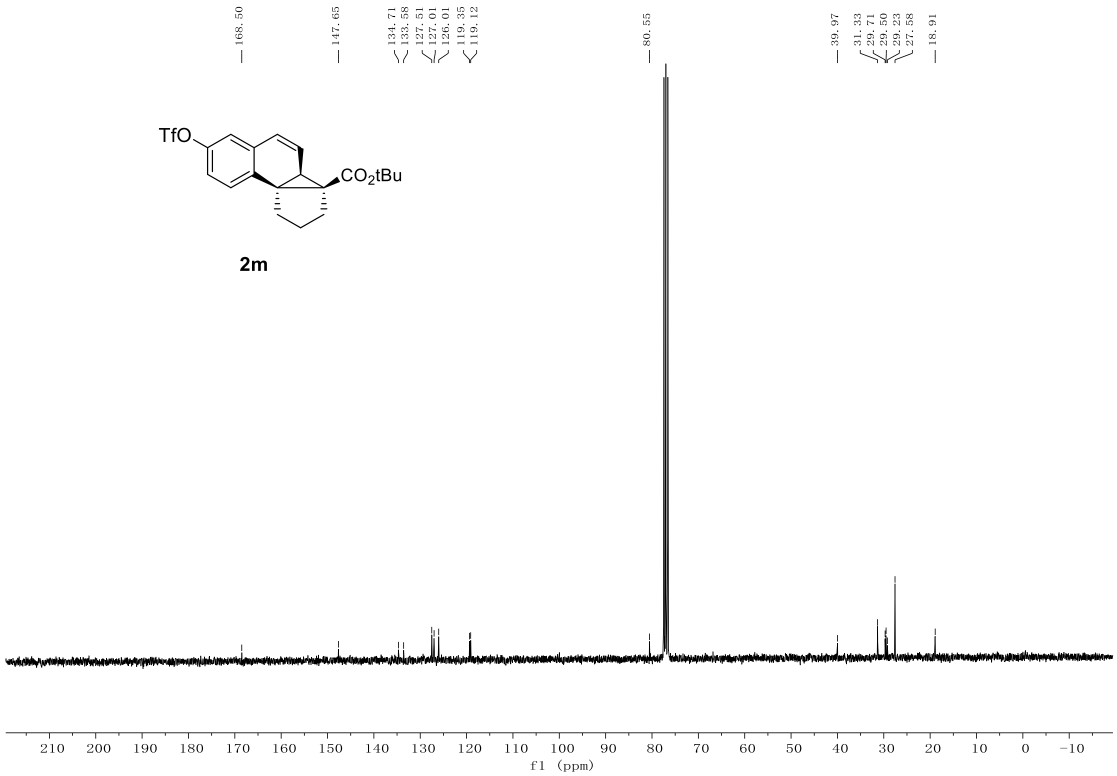

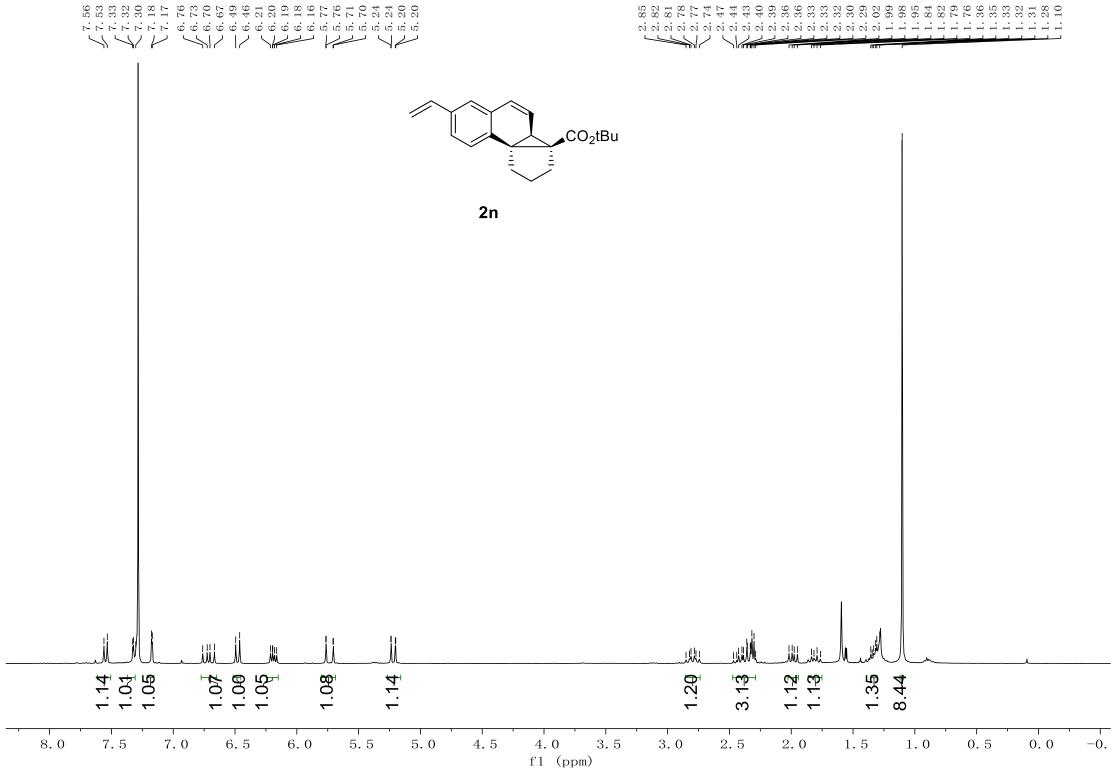

2n

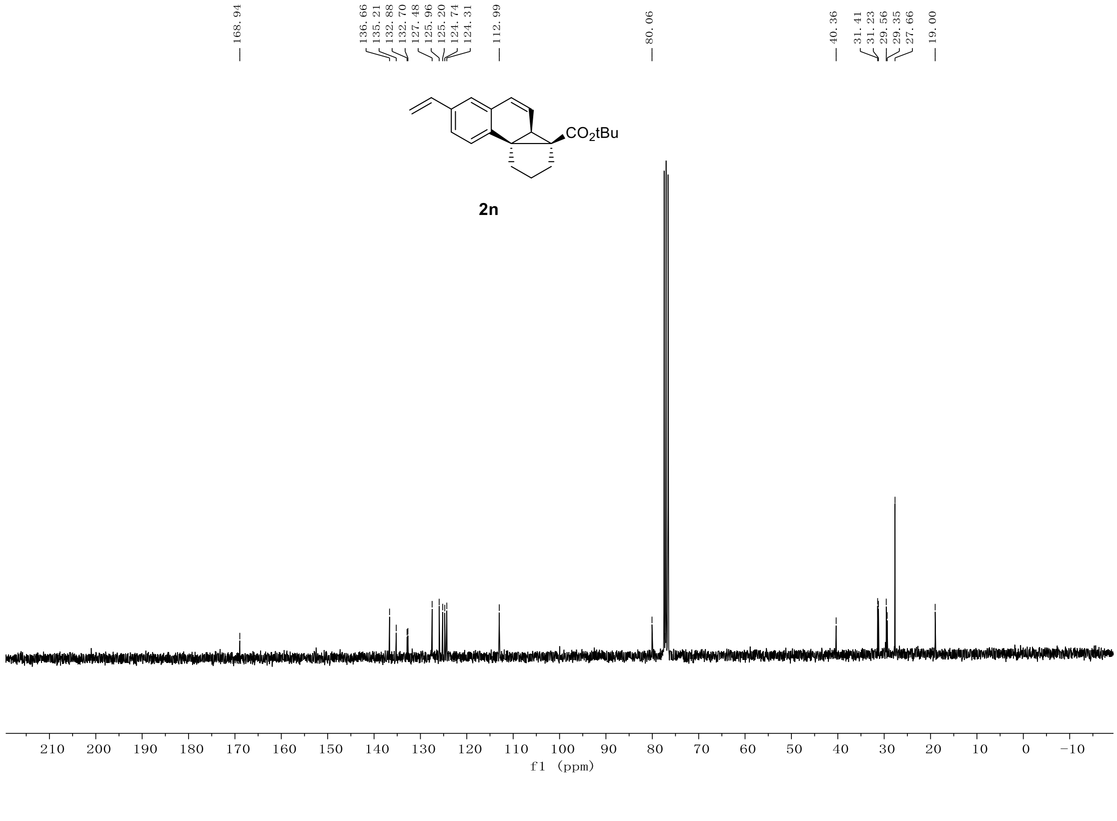

2n

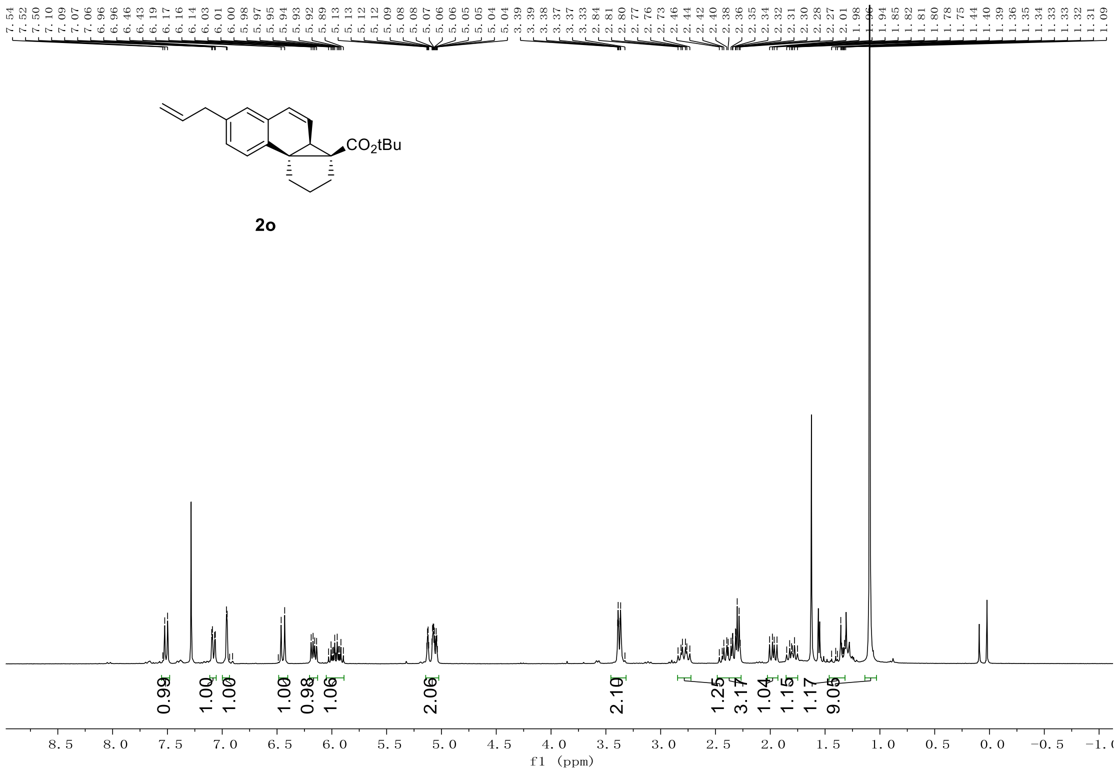
7.54 7.52 7.50 7.10 7.09 7.07 7.06 6.96 6.96 6.46 6.43 6.19 6.17 6.16 6.14 6.03 6.01 6.00 5.98 5.97 5.95 5.94 5.93 5.92 5.89 5.13 5.13 5.12 5.09 5.08 5.08 5.07 5.06 5.06 5.05 5.05 5.04 5.04 3.39 3.39 3.38 3.37 3.37 3.33 2.84 2.81 2.80 2.77 2.76 2.73 2.46 2.44 2.42 2.40 2.38 2.36 2.35 2.34 2.32 2.31 2.30 2.28 2.27 2.01 1.98 1.94 1.85 1.82 1.81 1.80 1.78 1.75 1.44 1.40 1.39 1.36 1.35 1.34 1.33 1.32 1.31 1.09
CO2tBu
2o
0.99 1.00 1.00 1.00 0.98 1.06 2.06 2.10 1.25 3.17 1.04 1.15 1.17 9.05
8.5 8.0 7.5 7.0 6.5 6.0 5.5 5.0 4.5 4.0 3.5 3.0 2.5 2.0 1.5 1.0 0.5 0.0 −0.5
f1 (ppm)

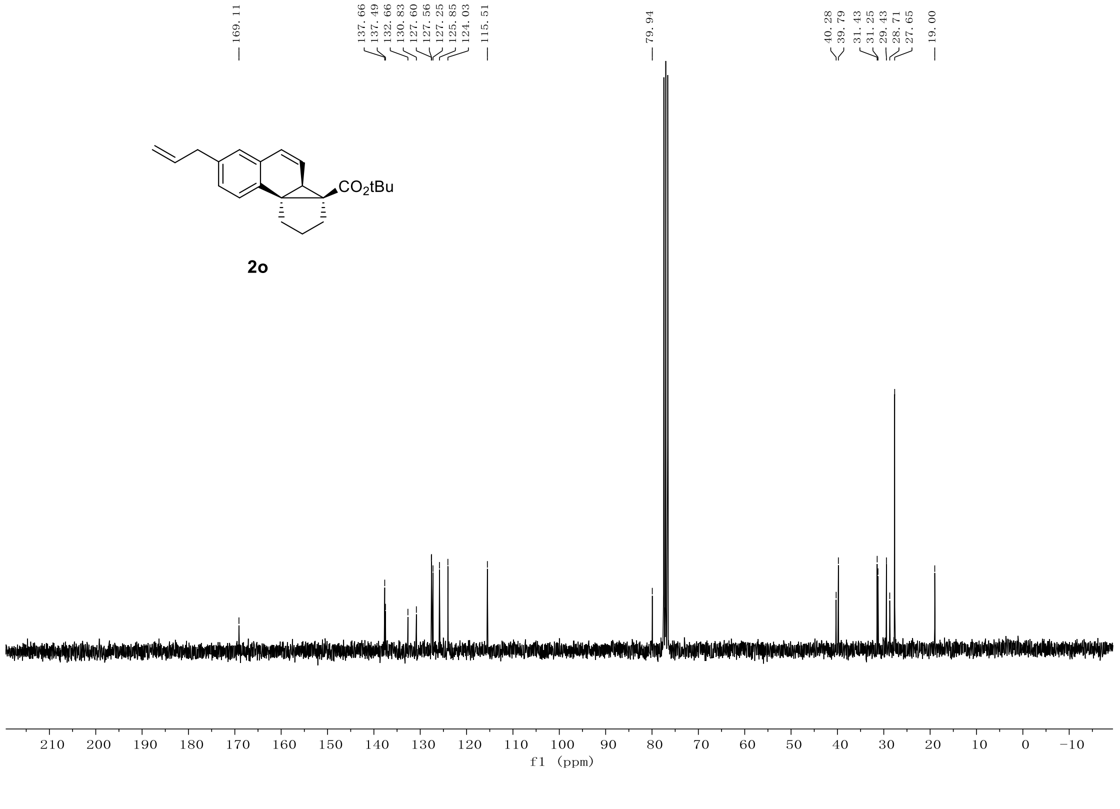
169.11
137.66 137.49 132.66 130.83 127.60 127.56 127.25 125.85 124.03 115.51
79.94
40.28 39.79 31.43 31.25 29.43 28.71 27.65 19.00
CO2tBu
2o
210 200 190 180 170 160 150 140 130 120 110 100 90 80 70 60 50 40 30 20 10 0 −10
f1 (ppm)

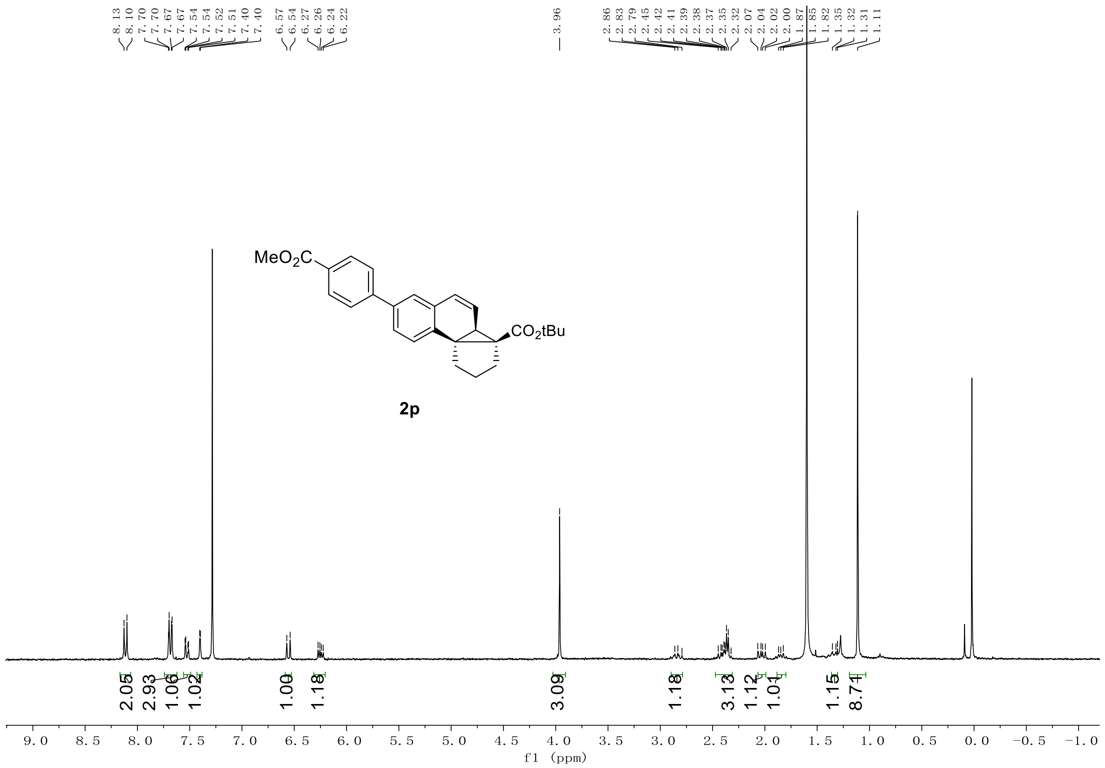
MeO2C
CO2tBu
2p
f1 (ppm)

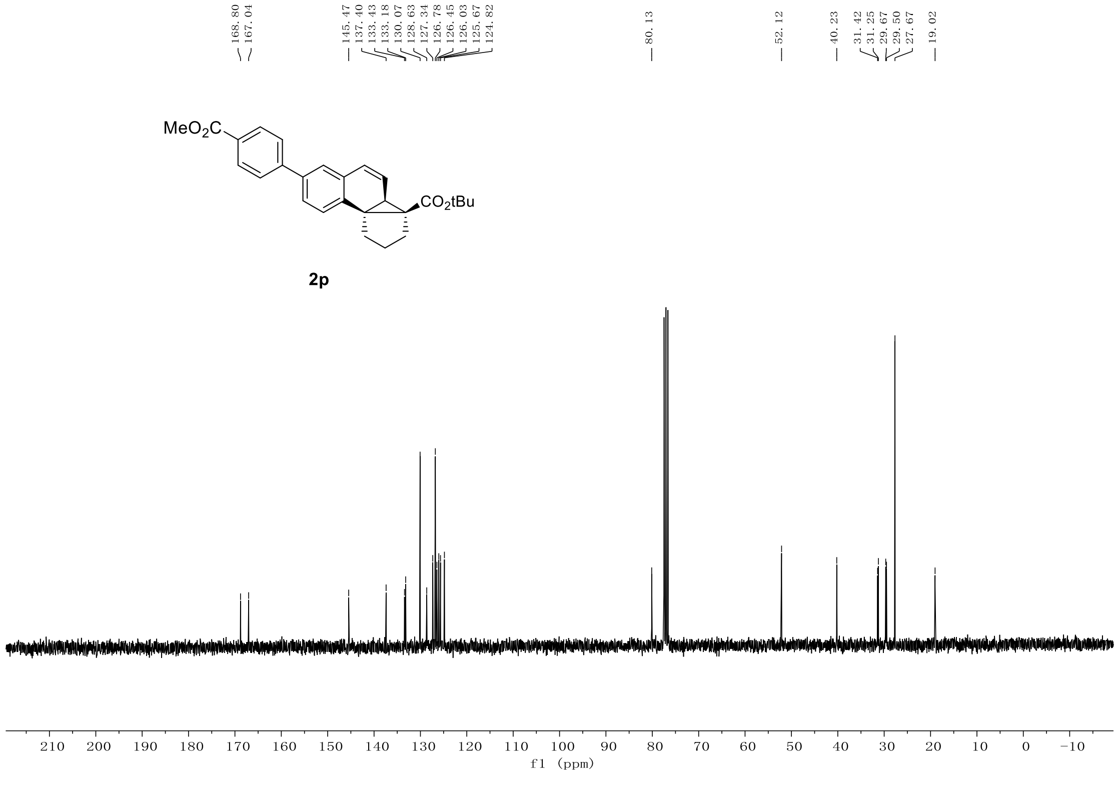
MeO2C
CO2tBu
2p
f1 (ppm)

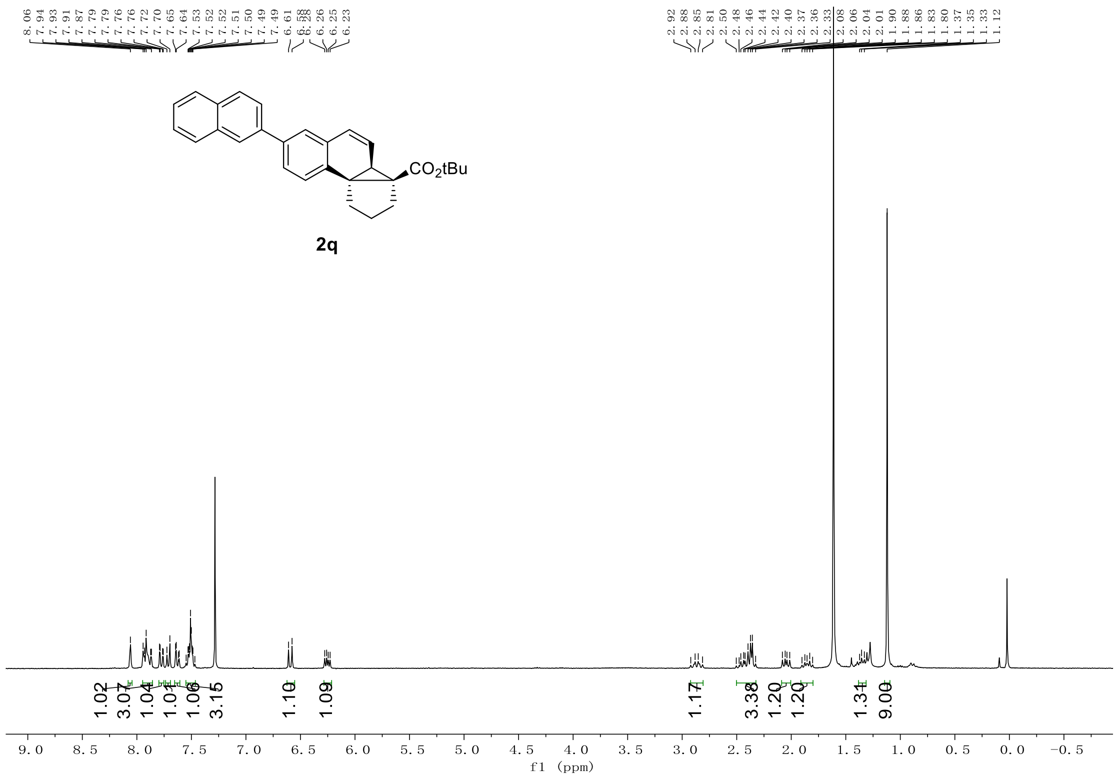

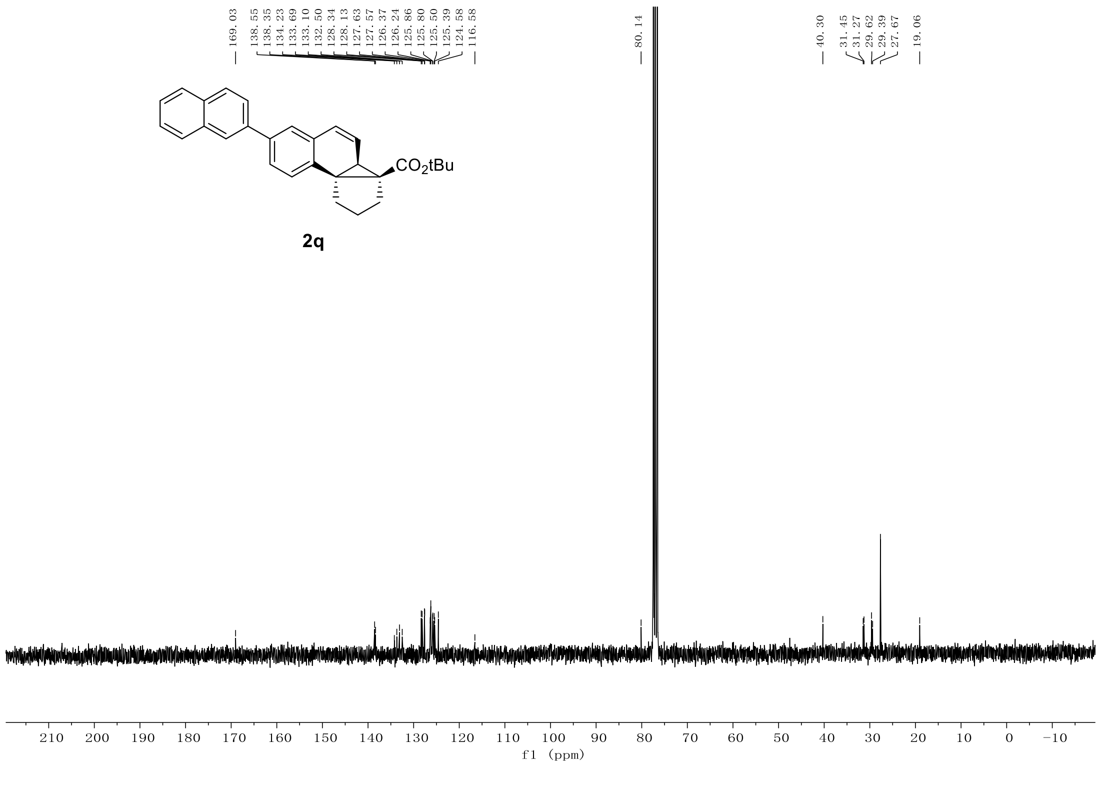

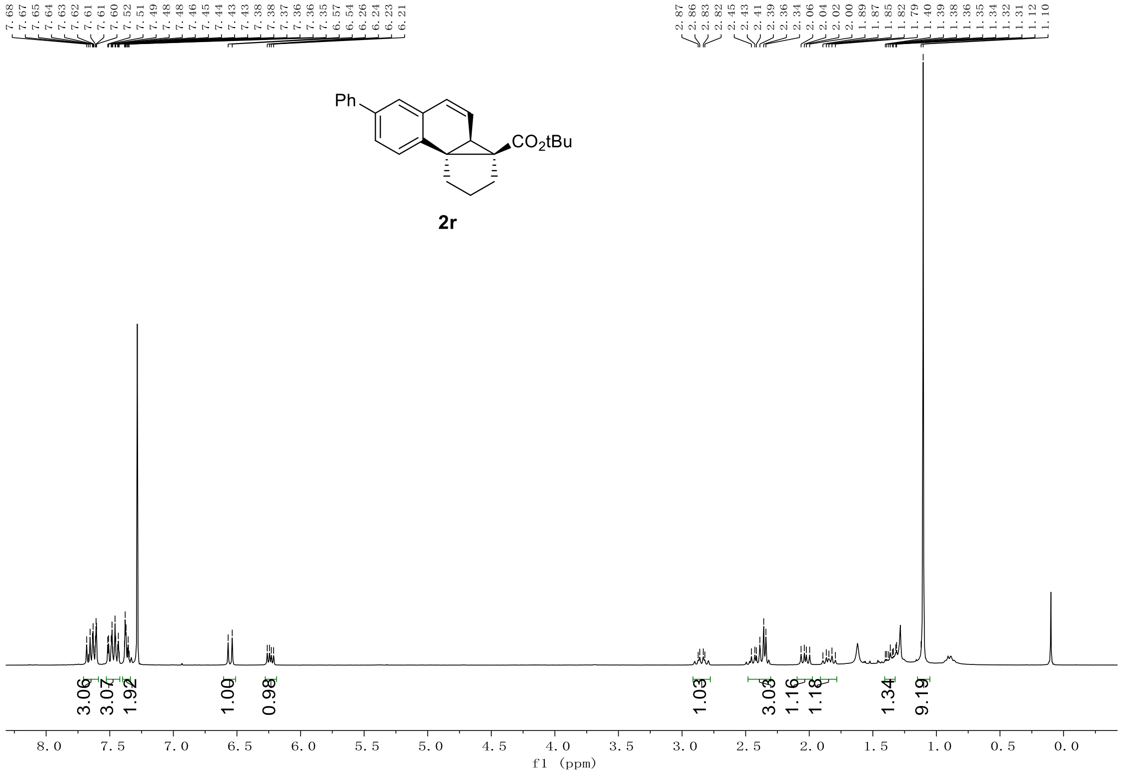
Ph
CO_2tBu
2r
f1 (ppm)

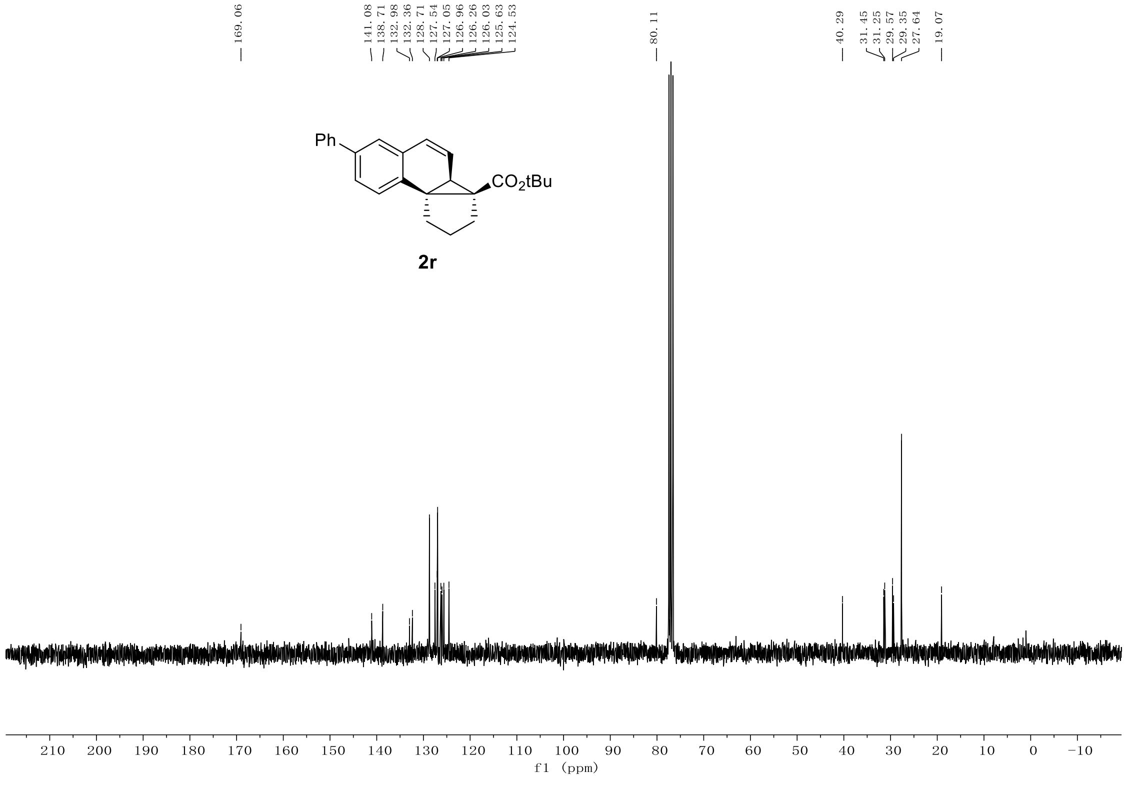
Ph
CO_2tBu
2r
f1 (ppm)

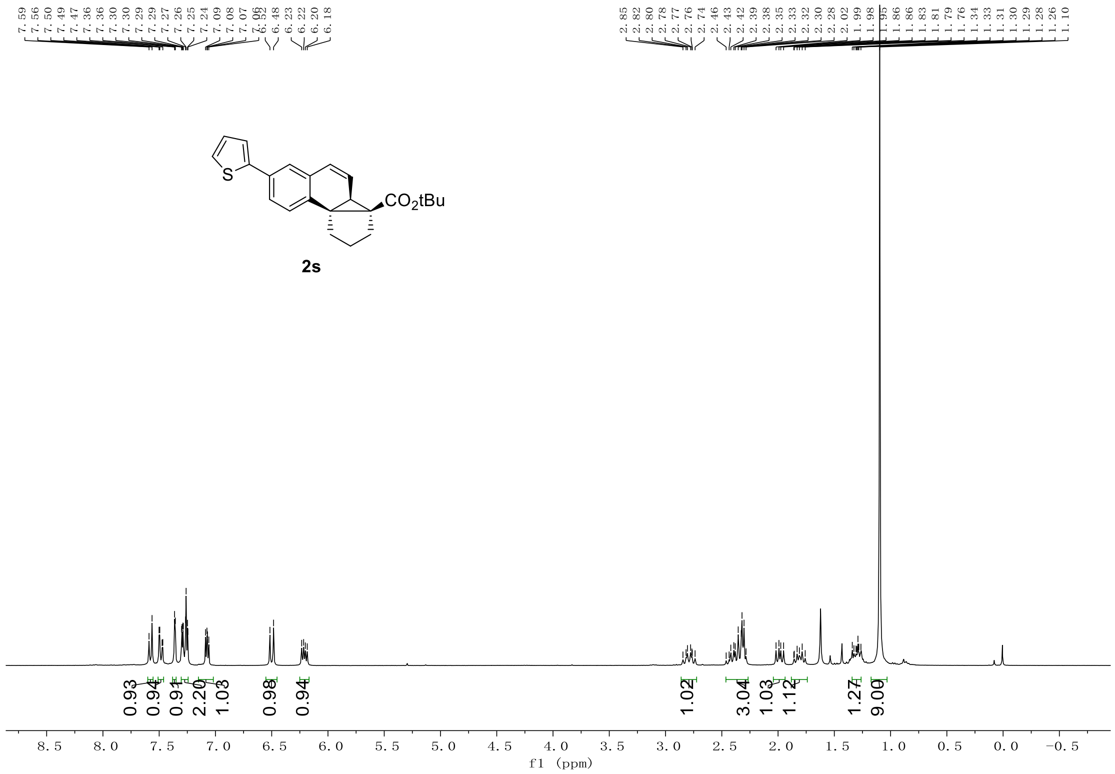
7.59 7.56 7.50 7.49 7.47 7.36 7.36 7.30 7.30 7.29 7.29 7.27 7.26 7.25 7.24 7.09 7.08 7.07 7.06 6.52 6.48 6.23 6.22 6.20 6.18
2.85 2.82 2.80 2.78 2.77 2.76 2.74 2.46 2.43 2.42 2.39 2.38 2.35 2.33 2.32 2.30 2.28 2.02 1.99 1.98 1.95 1.86 1.86 1.83 1.81 1.79 1.76 1.34 1.33 1.31 1.30 1.29 1.28 1.26 1.10
S
CO2tBu
2s
0.93 0.94 0.91 2.20 1.03 0.98 0.94 1.02 3.04 1.03 1.12 1.27 9.00
8.5 8.0 7.5 7.0 6.5 6.0 5.5 5.0 4.5 4.0 3.5 3.0 2.5 2.0 1.5 1.0 0.5 0.0 -0.5
f1 (ppm)

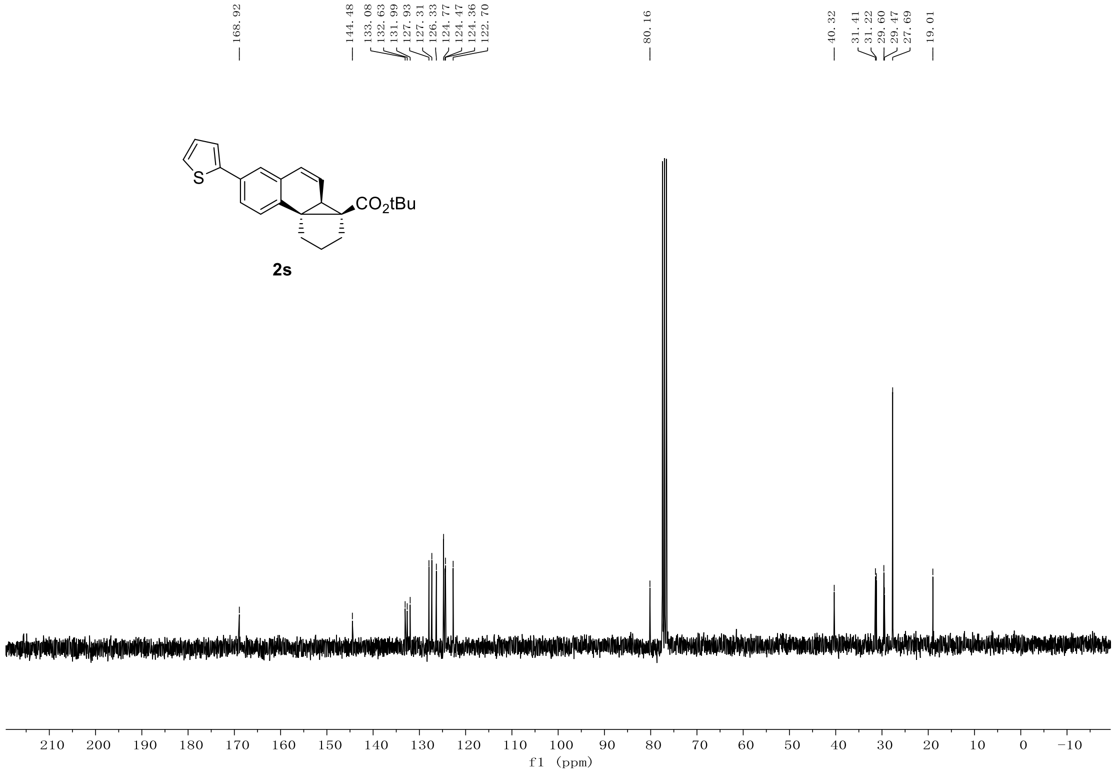
168.92
144.48 133.08 132.63 131.99 127.93 127.31 126.33 124.77 124.47 124.36 122.70
80.16
40.32 31.41 31.22 29.60 29.47 27.69 19.01
S
CO2tBu
2s
210 200 190 180 170 160 150 140 130 120 110 100 90 80 70 60 50 40 30 20 10 0 -10
f1 (ppm)

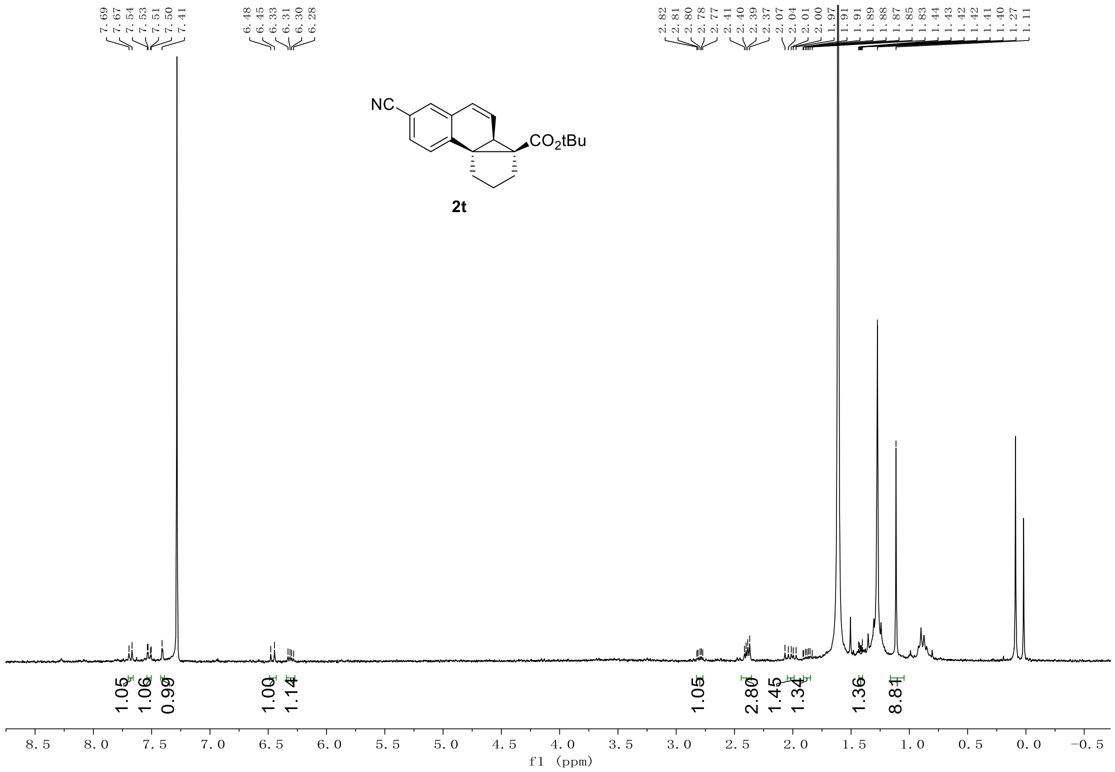
7.69
7.67
7.54
7.53
7.51
7.50
7.41
6.48
6.45
6.33
6.31
6.30
6.28
2.82
2.81
2.80
2.78
2.77
2.41
2.40
2.39
2.37
2.07
2.04
2.01
2.00
1.97
1.91
1.91
1.89
1.88
1.87
1.85
1.83
1.44
1.43
1.43
1.42
1.42
1.41
1.40
1.27
1.11
NC
CO2tBu
2t
1.05
1.06
0.99
1.00
1.14
1.05
2.80
1.45
1.34
1.36
8.81
f1 (ppm)

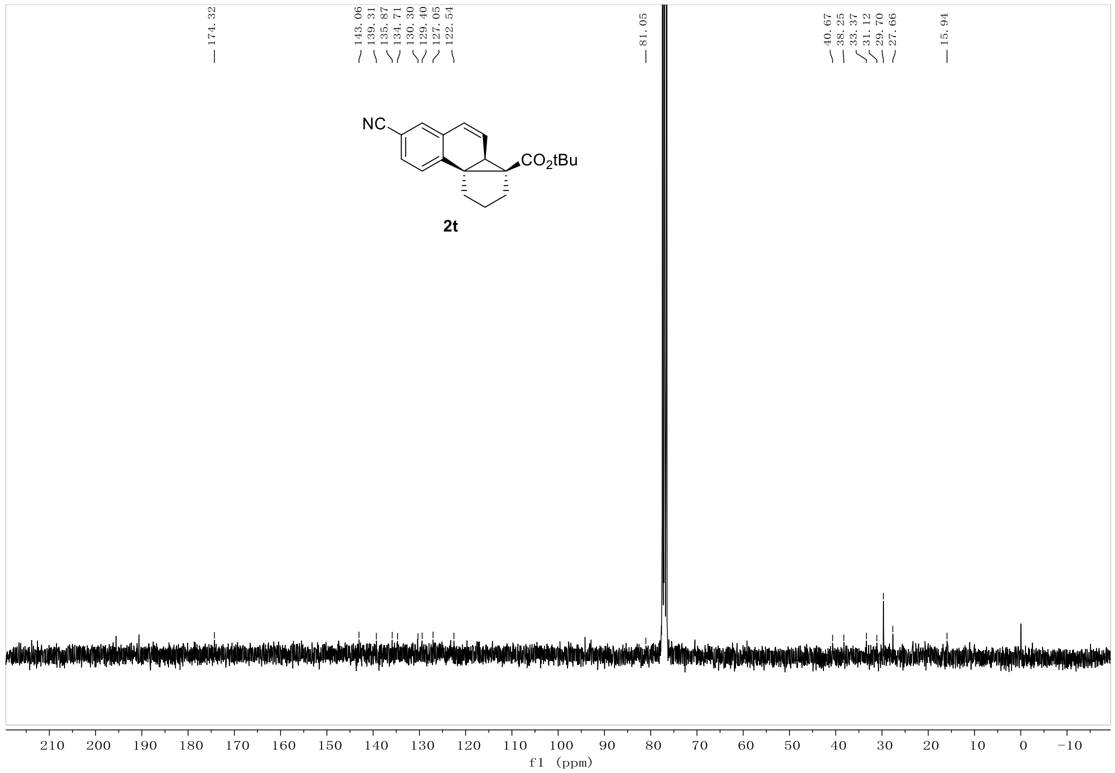
174.32
143.06
139.31
135.87
134.71
130.30
129.40
127.05
122.54
81.05
40.67
38.25
33.37
31.12
29.70
27.66
15.94
NC
CO2tBu
2t
f1 (ppm)

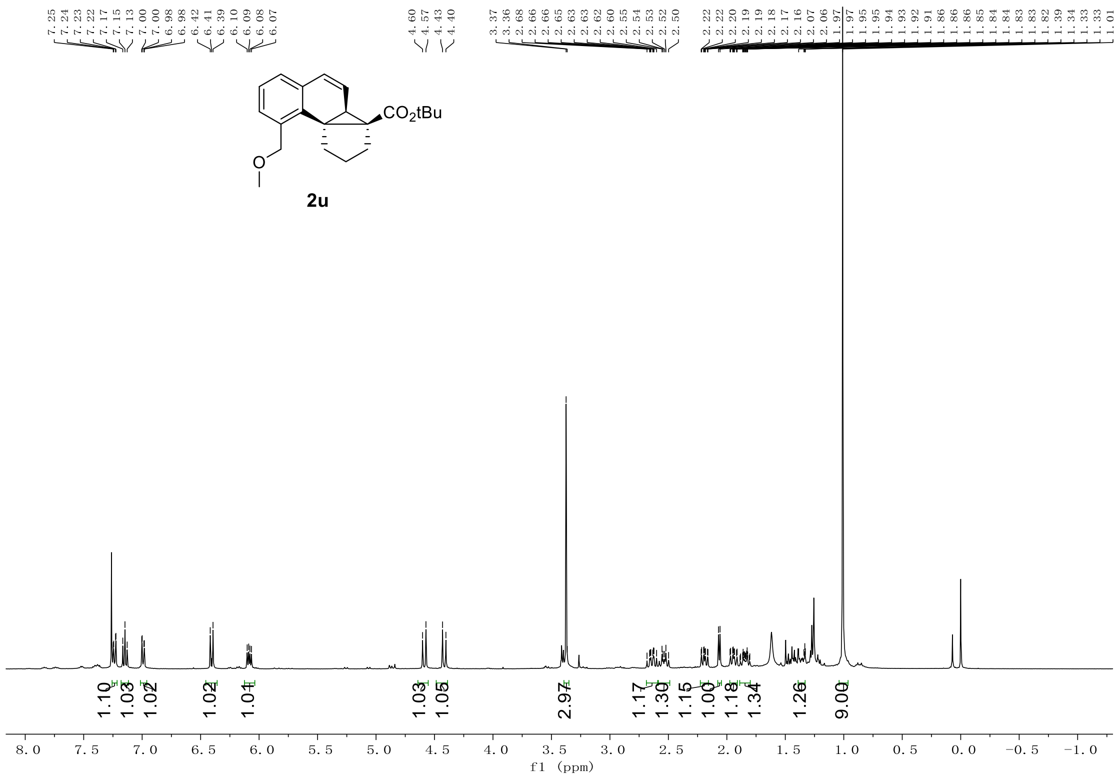
7.25 7.24 7.23 7.22 7.17 7.15 7.13 7.00 7.00 6.98 6.98 6.42 6.41 6.39 6.10 6.09 6.08 6.07
4.60 4.57 4.43 4.40
3.37 3.36 2.68 2.66 2.66 2.65 2.63 2.63 2.62 2.60 2.55 2.54 2.53 2.52 2.50
2.22 2.22 2.20 2.19 2.19 2.18 2.17 2.16 2.07 2.06 1.97 1.97 1.95 1.95 1.94 1.93 1.92 1.91 1.86 1.86 1.85 1.84 1.84 1.83 1.83 1.82 1.39 1.34 1.33 1.33 1.01
CO2tBu
O
2u
1.10 1.03 1.02 1.02 1.01 1.03 1.05 2.97 1.17 1.30 1.15 1.00 1.18 1.34 1.26 9.00
8.0 7.5 7.0 6.5 6.0 5.5 5.0 4.5 4.0 3.5 3.0 2.5 2.0 1.5 1.0 0.5 0.0 −0.5 −1.0
f1 (ppm)

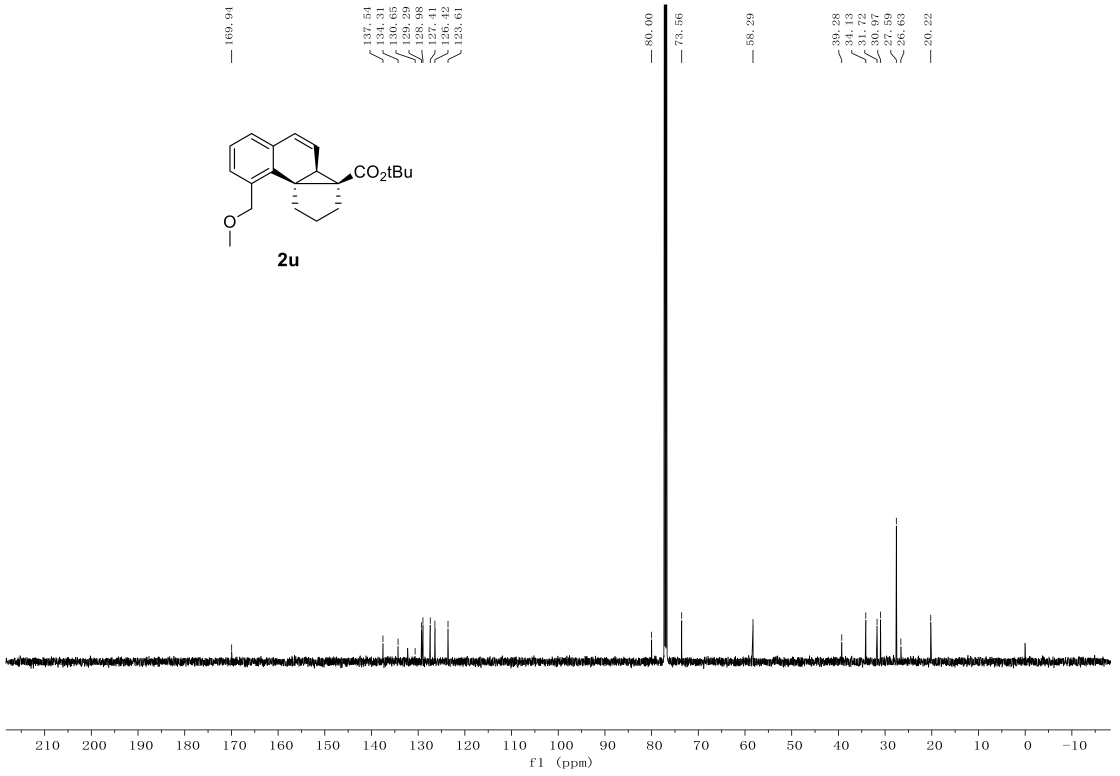
169.94
137.54 134.31 130.65 129.29 128.98 127.41 126.42 123.61
80.00
73.56
58.29
39.28 34.13 31.72 30.97 27.59 26.63
20.22
CO2tBu
O
2u
210 200 190 180 170 160 150 140 130 120 110 100 90 80 70 60 50 40 30 20 10 0 −10
f1 (ppm)

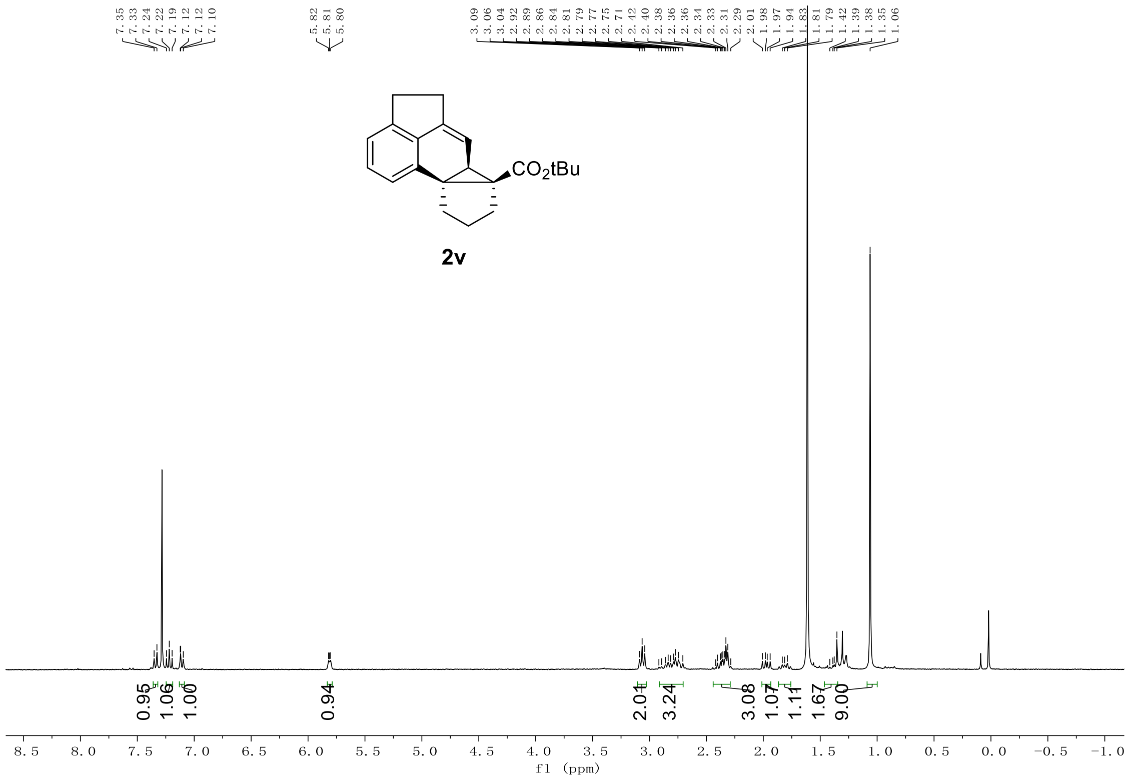

7.35
7.33
7.24
7.22
7.19
7.12
7.12
7.10
5.82
5.81
5.80
3.09
3.06
3.04
2.92
2.89
2.86
2.84
2.81
2.79
2.77
2.75
2.71
2.42
2.40
2.38
2.36
2.36
2.34
2.33
2.31
2.29
2.01
1.98
1.97
1.94
1.83
1.81
1.79
1.42
1.39
1.38
1.35
1.06
CO2tBu
2v
0.95
1.06
1.00
0.94
2.04
3.24
3.08
1.07
1.11
1.67
9.00
f1 (ppm)

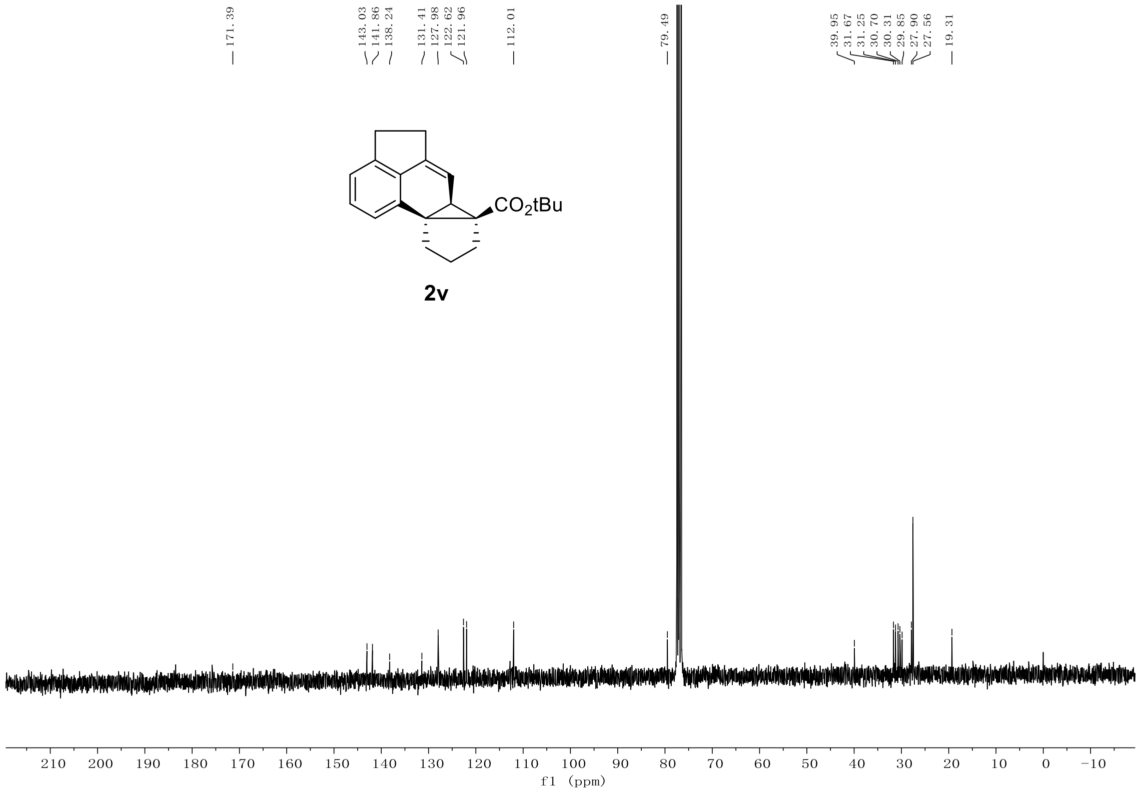

171.39
143.03
141.86
138.24
131.41
127.98
122.62
121.96
112.01
79.49
39.95
31.67
31.25
30.70
30.31
29.85
27.90
27.56
19.31
CO2tBu
2v
f1 (ppm)

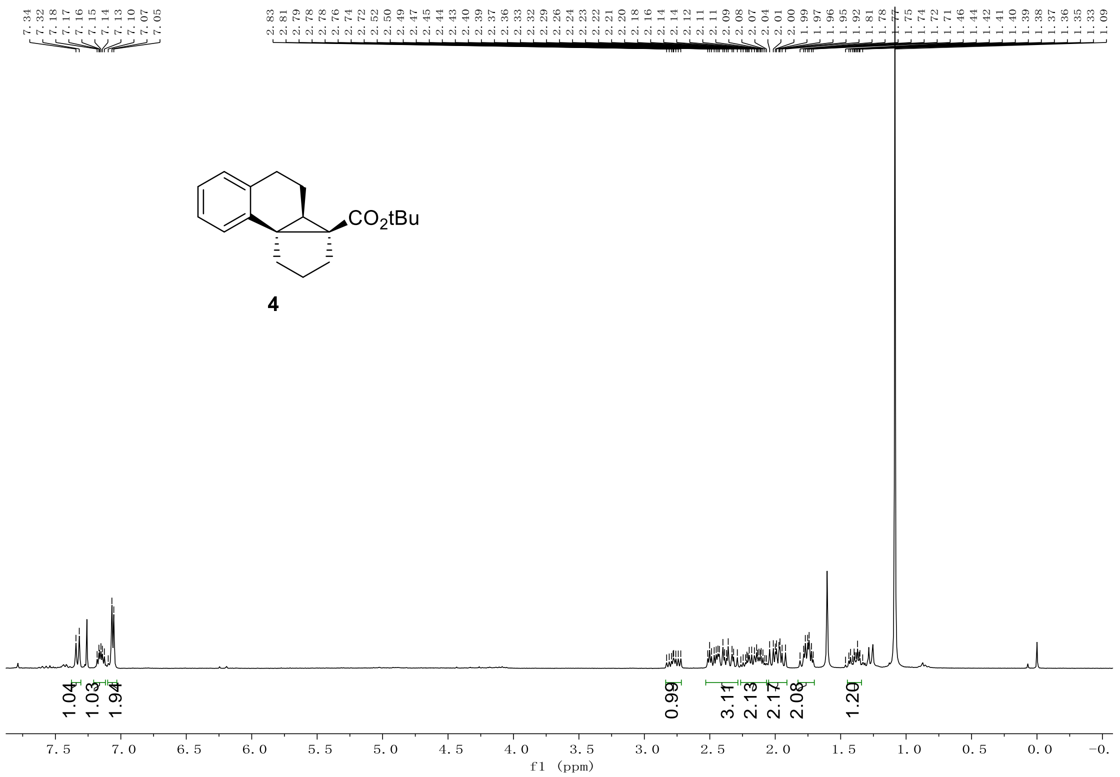
CO2tBu
4
f1 (ppm)

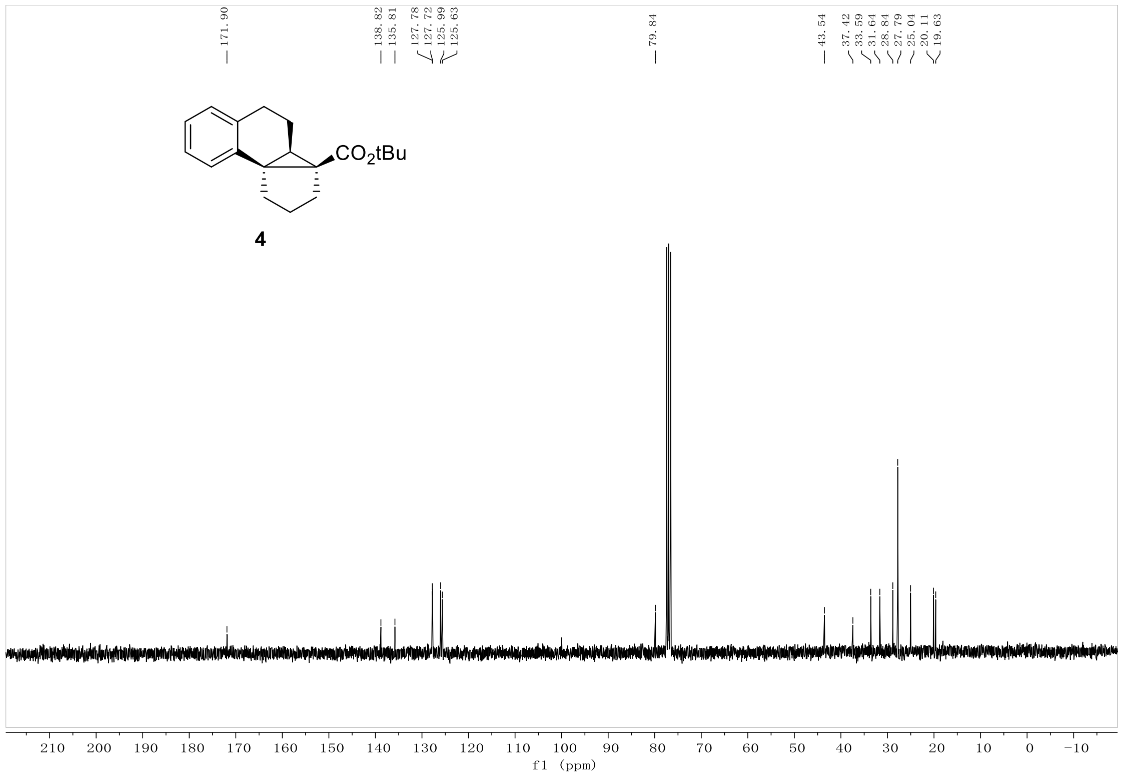
171.90
138.82
135.81
127.78
127.72
125.99
125.63
79.84
43.54
37.42
33.59
31.64
28.84
27.79
25.04
20.11
19.63
CO2tBu
4
f1 (ppm)

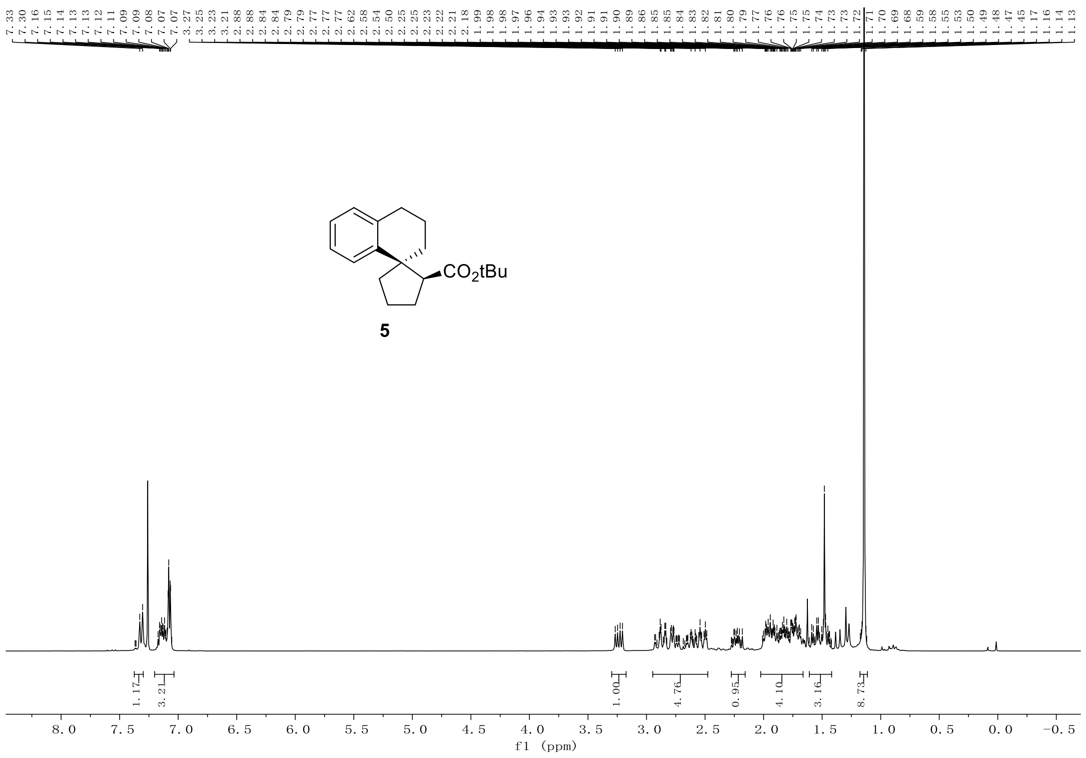
7.33 7.30 7.16 7.15 7.14 7.13 7.13 7.12 7.11 7.09 7.08 7.07 7.07 3.27 3.25 3.23 3.21 2.88 2.88 2.84 2.84 2.79 2.79 2.77 2.77 2.62 2.58 2.54 2.50 2.25 2.25 2.23 2.22 2.21 2.18 1.99 1.98 1.98 1.97 1.96 1.94 1.93 1.93 1.92 1.91 1.91 1.91 1.90 1.89 1.86 1.85 1.85 1.84 1.83 1.82 1.81 1.80 1.79 1.77 1.76 1.75 1.75 1.74 1.73 1.72 1.71 1.70 1.69 1.68 1.59 1.58 1.55 1.53 1.50 1.49 1.48 1.47 1.45 1.17 1.16 1.14 1.13
CO2tBu
5
1.17
3.21
1.00
4.76
0.95
4.10
3.16
8.73
8.0 7.5 7.0 6.5 6.0 5.5 5.0 4.5 4.0 3.5 3.0 2.5 2.0 1.5 1.0 0.5 0.0 -0.5
f1 (ppm)

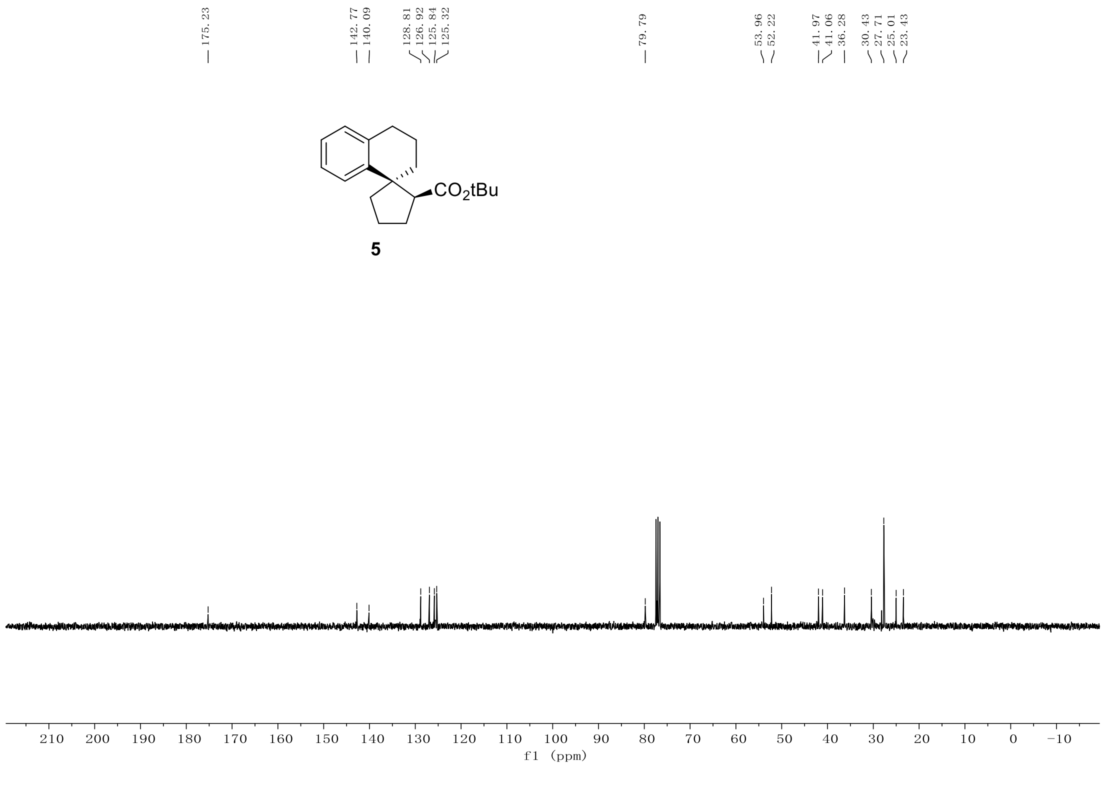
175.23
142.77 140.09
128.81 126.92 125.84 125.32
79.79
53.96 52.22
41.97 41.06 36.28
30.43 27.71 25.01 23.43
CO2tBu
5
210 200 190 180 170 160 150 140 130 120 110 100 90 80 70 60 50 40 30 20 10 0 -10
f1 (ppm)

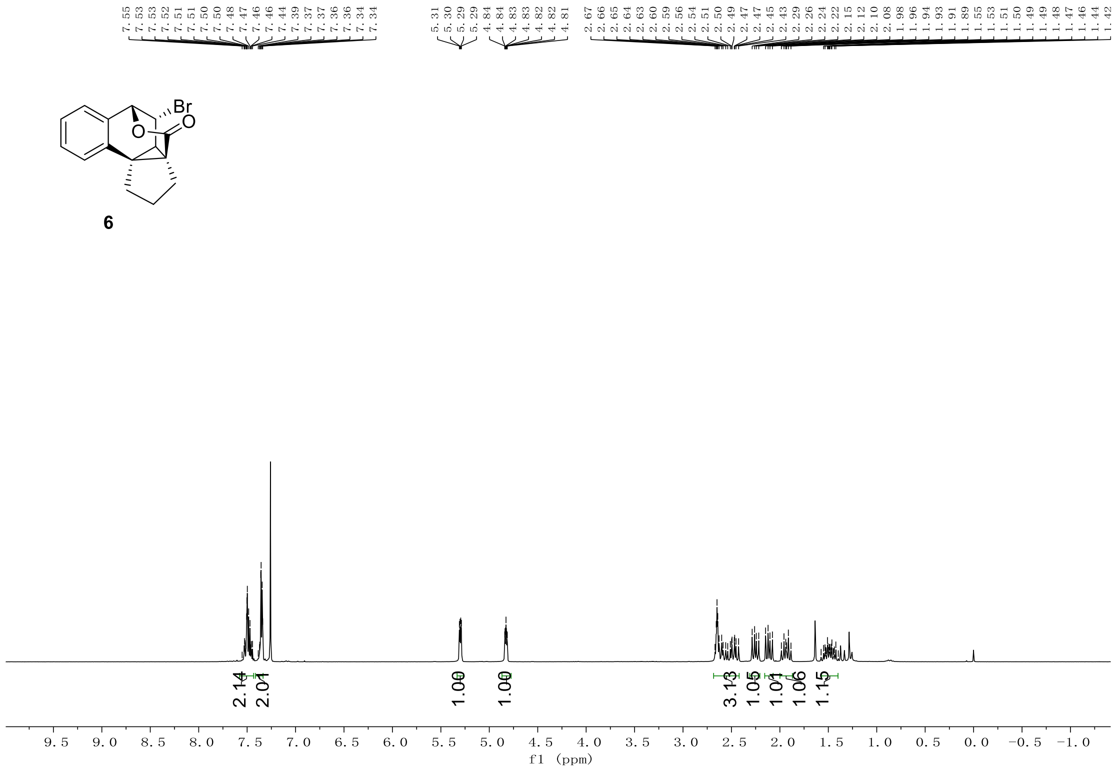

7.55 7.53 7.53 7.52 7.51 7.51 7.50 7.50 7.48 7.47 7.46 7.46 7.44 7.39 7.37 7.37 7.36 7.36 7.34 7.34
5.31 5.30 5.29 5.29 4.84 4.84 4.83 4.83 4.82 4.81
2.67 2.66 2.65 2.64 2.63 2.60 2.59 2.56 2.54 2.51 2.50 2.49 2.47 2.45 2.43 2.29 2.26 2.24 2.22 2.15 2.12 2.10 2.08 1.98 1.96 1.94 1.93 1.91 1.89 1.55 1.53 1.51 1.50 1.49 1.49 1.48 1.47 1.46 1.44 1.42
Br
O
O
6
2.14 2.01 1.00 1.00 3.13 1.05 1.01 1.06 1.15
9.5 9.0 8.5 8.0 7.5 7.0 6.5 6.0 5.5 5.0 4.5 4.0 3.5 3.0 2.5 2.0 1.5 1.0 0.5 0.0 -0.5 -1.0
f1 (ppm)

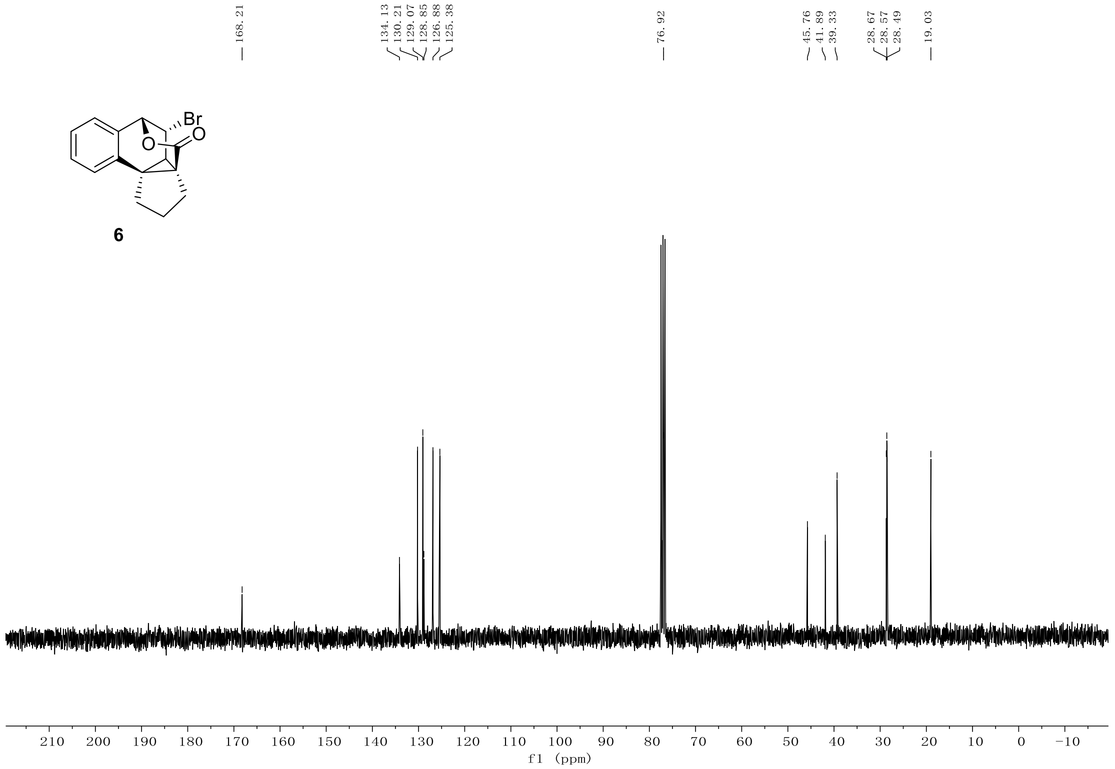

168.21
134.13 130.21 129.07 128.85 126.88 125.38
76.92
45.76 41.89 39.33
28.67 28.57 28.49
19.03
Br
O
O
6
210 200 190 180 170 160 150 140 130 120 110 100 90 80 70 60 50 40 30 20 10 0 -10
f1 (ppm)

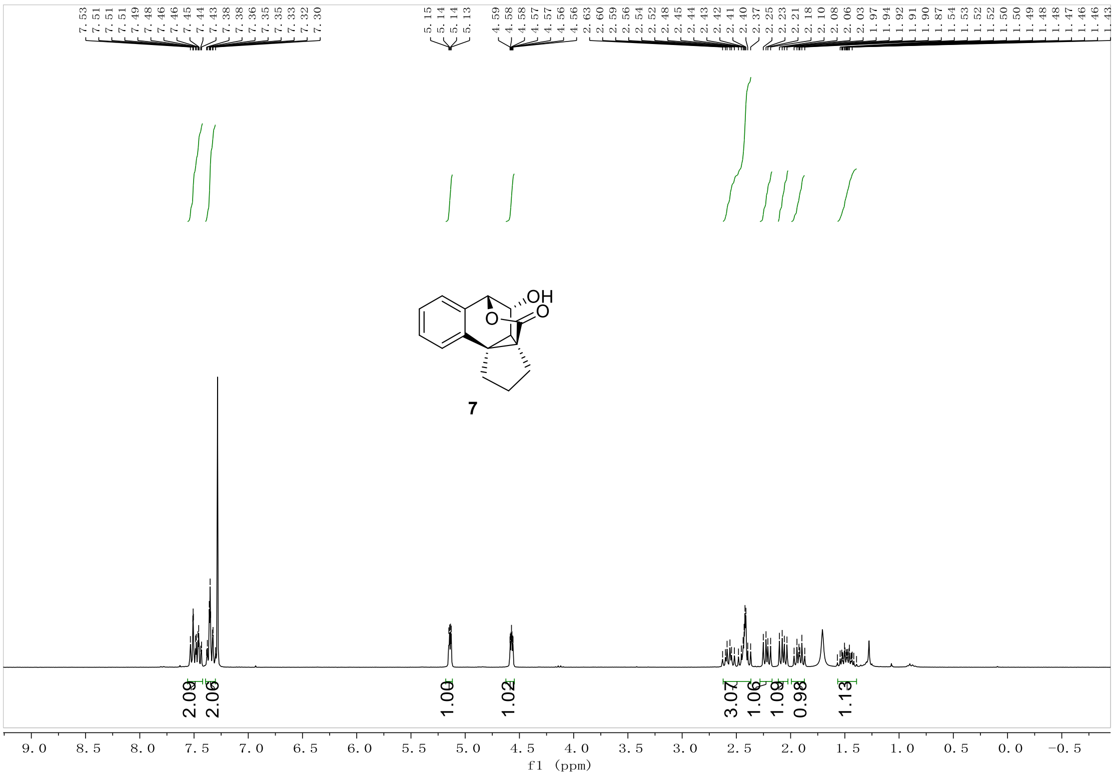

OH
O
O
7
2.09
2.06
1.00
1.02
3.07
1.06
1.09
0.98
1.13
f1 (ppm)

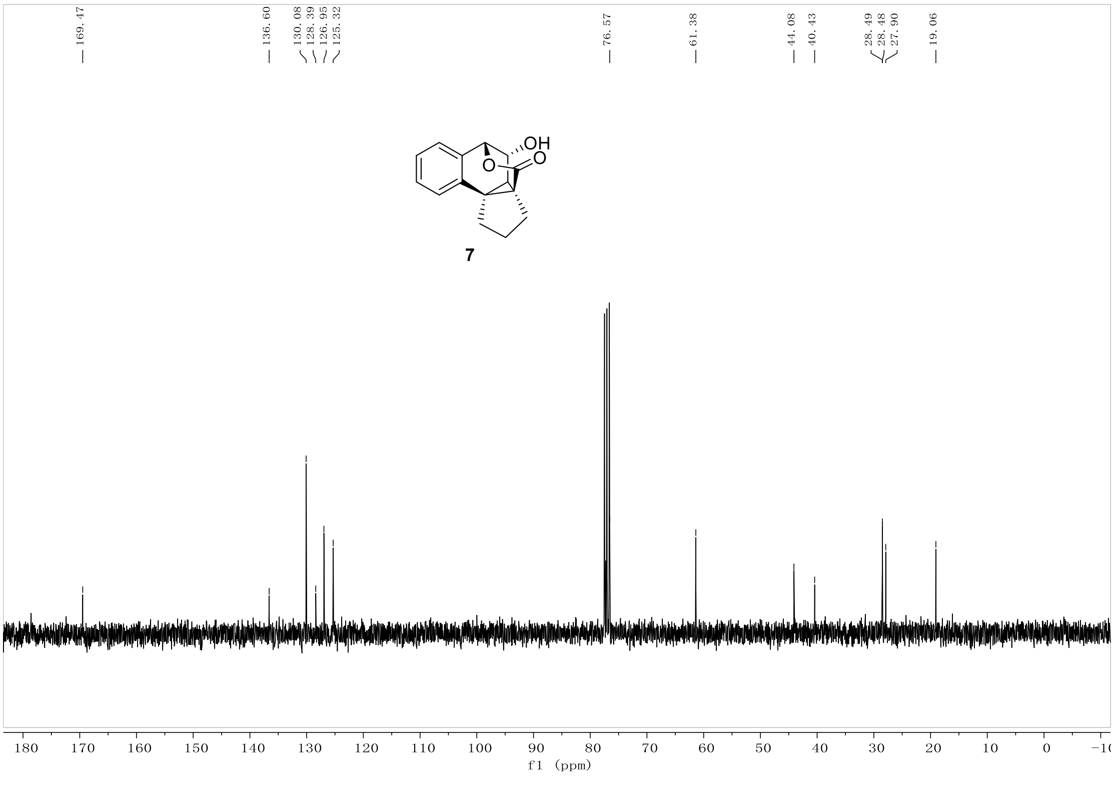

169.47
136.60
130.08
128.39
126.95
125.32
76.57
61.38
44.08
40.43
28.49
28.48
27.90
19.06
OH
O
O
7
f1 (ppm)

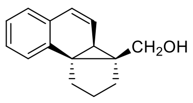

8

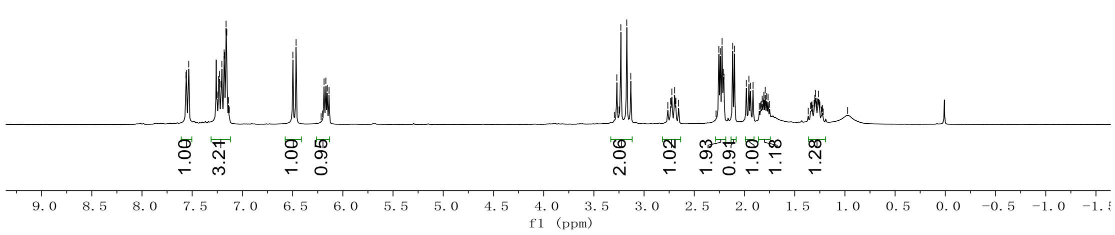

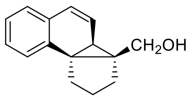

8

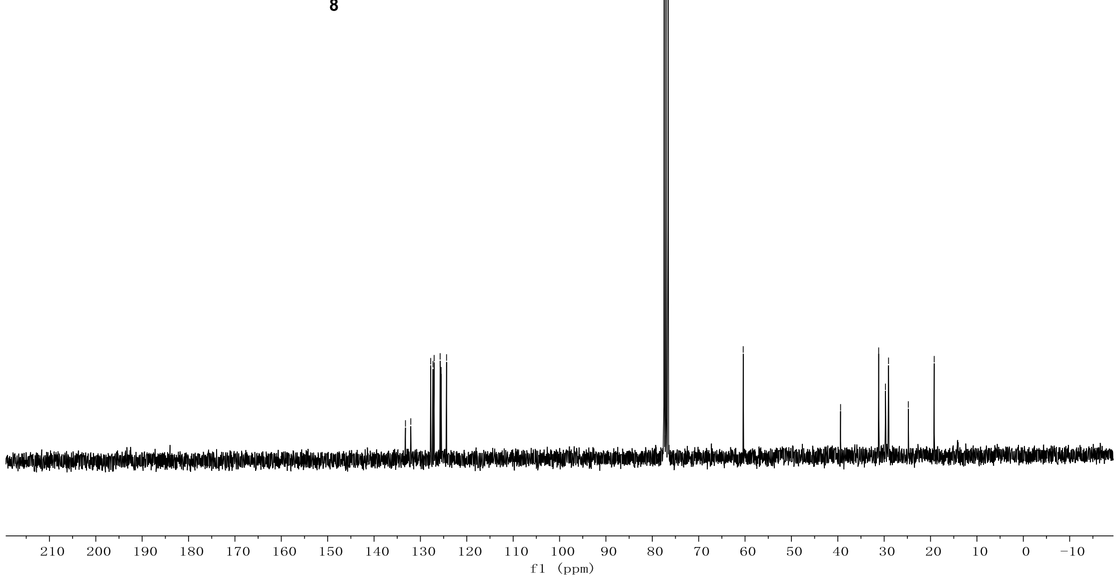

6. HPLC spectra of products 2

Chiral HPLC spectrum of racemic **2a**

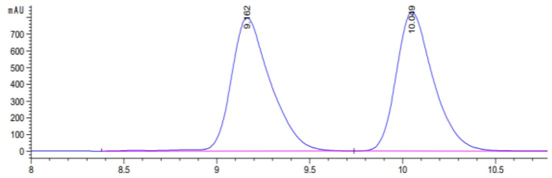

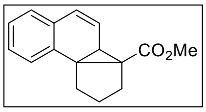

峰 #	保留时间 [min]	类型	峰宽 [min]	峰面积 [mAU*s]	峰高 [mAU]	峰面积 %
1	9.162	VV	0.2167	1.16504e4	796.38782	50.1602
2	10.049	VB	0.2086	1.15760e4	830.50977	49.8398

Chiral HPLC spectrum of **2a**

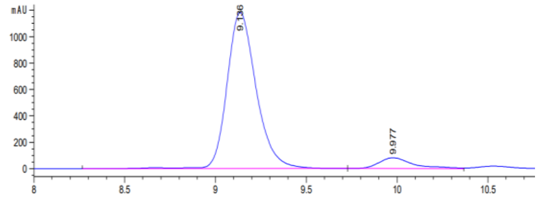

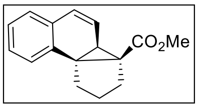

峰 #	保留时间 [min]	类型	峰宽 [min]	峰面积 [mAU*s]	峰高 [mAU]	峰面积 %
1	9.136	VV	0.1690	1.31090e4	1185.70947	92.3435
2	9.977	VV	0.1960	1086.91162	82.33852	7.6565

Chiral HPLC spectrum of racemic **2b**

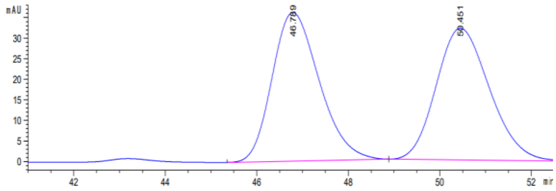

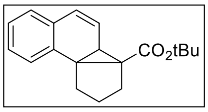

峰 #	保留时间 [min]	类型	峰宽 [min]	峰面积 [mAU*s]	峰高 [mAU]	峰面积 %
1	45.612	BV	1.0606	2636.27368	38.41492	49.9549
2	48.602	VB	1.2196	2641.02930	33.57762	50.0451

Chiral HPLC spectrum of **2b**

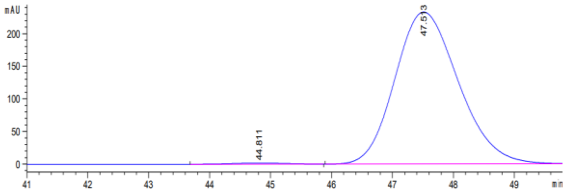

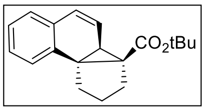

峰 #	保留时间 [min]	类型	峰宽 [min]	峰面积 [mAU*s]	峰高 [mAU]	峰面积 %
1	44.811	BV	0.9266	118.46732	1.93406	0.6916
2	47.513	VBA	1.1324	1.70119e4	233.39944	99.3084

Chiral HPLC spectrum of racemic **2c**

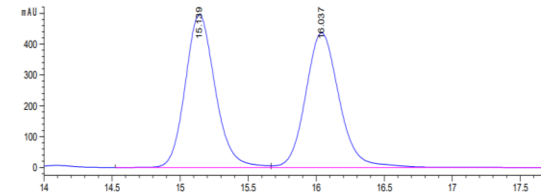

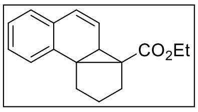

峰 #	保留时间 [min]	类型	峰宽 [min]	峰面积 [mAU*s]	峰高 [mAU]	峰面积 %
1	15.139	BV	0.2344	7587.61230	496.13843	50.3800
2	16.037	VB	0.2636	7473.14795	436.79535	49.6200

Chiral HPLC spectrum of **2c**

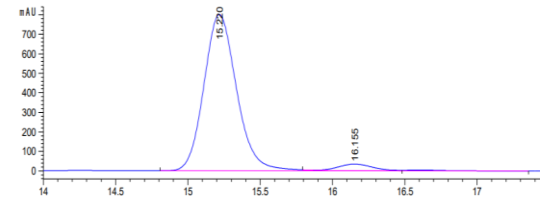

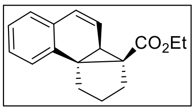

峰 #	保留时间 [min]	类型	峰宽 [min]	峰面积 [mAU*s]	峰高 [mAU]	峰面积 %
1	15.220	VV	0.2436	1.26107e4	801.22510	95.4415
2	16.155	VV	0.2627	602.30969	35.00448	4.5585

Chiral HPLC spectrum of racemic **2d**

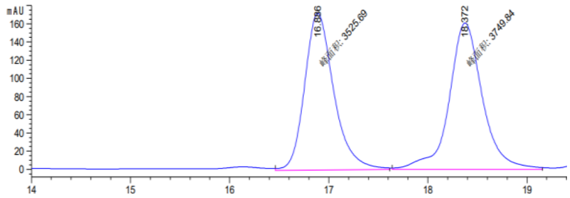

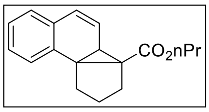

峰 #	保留时间 [min]	类型	峰宽 [min]	峰面积 [mAU*s]	峰高 [mAU]	峰面积 %
1	16.887	VV	0.3092	1.90921e4	939.18213	47.8963
2	18.373	VV	0.3520	2.07692e4	877.78296	52.1037

Chiral HPLC spectrum of **2d**

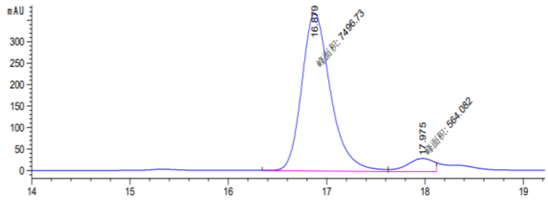

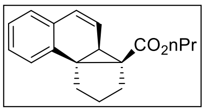

峰 #	保留时间 [min]	类型	峰宽 [min]	峰面积 [mAU*s]	峰高 [mAU]	峰面积 %
1	16.880	VV	0.3053	4.01681e4	1991.45142	93.8312
2	17.975	MM	0.3127	2640.79956	140.75777	6.1688

Chiral HPLC spectrum of racemic **2e**

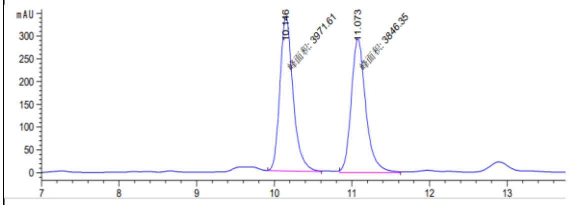

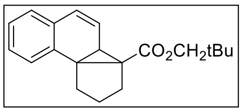

峰 #	保留时间 [min]	类型	峰宽 [min]	峰面积 [mAU*s]	峰高 [mAU]	峰面积 %
1	10.146	VV	0.1881	2.53436e4	2052.57300	54.4146
2	11.073	VV	0.2002	2.12314e4	1606.12585	45.5854

Chiral HPLC spectrum of **2e**

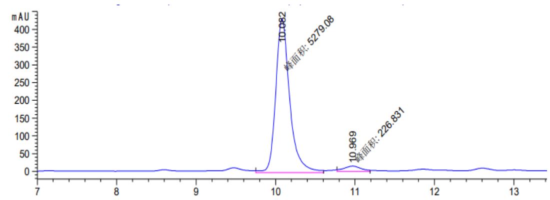

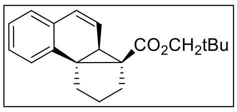

峰 #	保留时间 [min]	类型	峰宽 [min]	峰面积 [mAU*s]	峰高 [mAU]	峰面积 %
1	10.082	HV	0.1827	2.80360e4	2324.81177	95.9472
2	10.956	MM	0.2569	1184.24609	76.82060	4.0528

Chiral HPLC spectrum of racemic **2f**

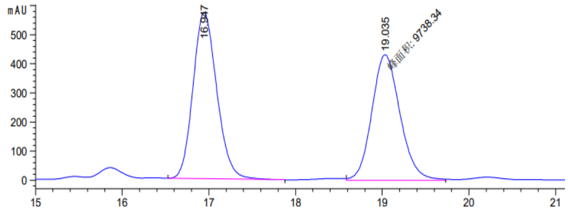

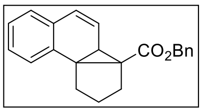

峰 #	保留时间 [min]	类型	峰宽 [min]	峰面积 [mAU*s]	峰高 [mAU]	峰面积 %
1	16.949	VV	0.3249	7.09231e4	3406.38452	50.9866
2	19.037	VV	0.3587	6.81783e4	2938.30444	49.0134

Chiral HPLC spectrum of **2f**

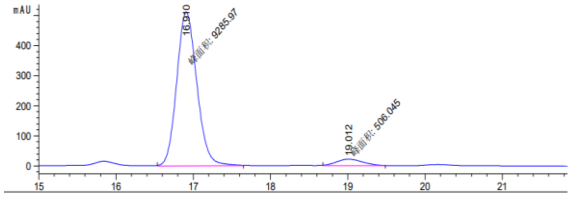

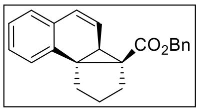

峰 #	保留时间 [min]	类型	峰宽 [min]	峰面积 [mAU*s]	峰高 [mAU]	峰面积 %
1	16.912	MM	0.3112	6.04140e4	3235.45996	95.2685
2	18.999	MM	0.3570	3000.48364	140.09404	4.7315

Chiral HPLC spectrum of racemic **2g**

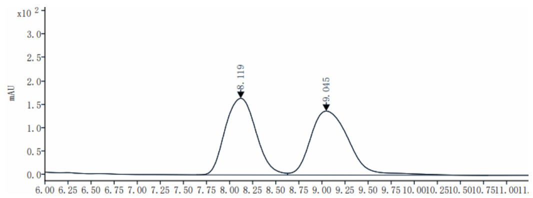

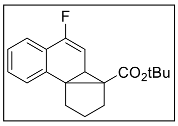

VWD1A, Wavelength=254 nm

Ret. Time	Area	Height	Area%
8.119	3798.21	163.59	49.15
9.045	3929.96	136.61	50.85

Chiral HPLC spectrum of **2g**

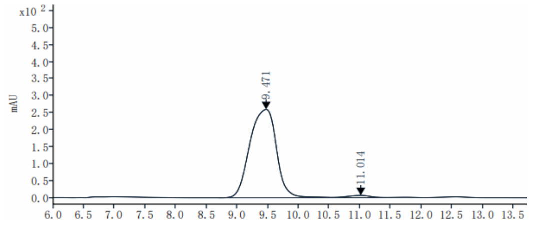

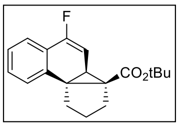

VWD1A, Wavelength=254 nm

Ret. Time	Area	Height	Area%
9.471	7833.37	259.77	97.94
11.014	164.97	6.56	2.06

Chiral HPLC spectrum of racemic **2h**

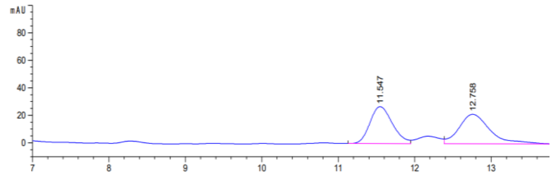

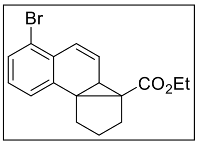

峰 #	保留时间 [min]	类型	峰宽 [min]	峰面积 [mAU*s]	峰高 [mAU]	峰面积 %
1	11.547	BV	0.3171	550.73297	26.87811	47.4429
2	12.758	VB	0.4272	610.09991	21.48967	52.5571

Chiral HPLC spectrum of **2h**

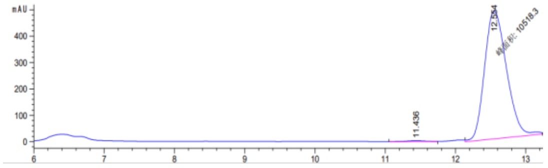

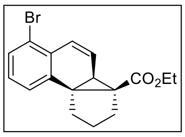

峰 #	保留时间 [min]	类型	峰宽 [min]	峰面积 [mAU*s]	峰高 [mAU]	峰面积 %
1	11.436	BB	0.2537	59.12213	3.59786	0.5764
2	12.554	MM	0.3547	1.01987e4	479.25613	99.4236

Chiral HPLC spectrum of racemic **2i**

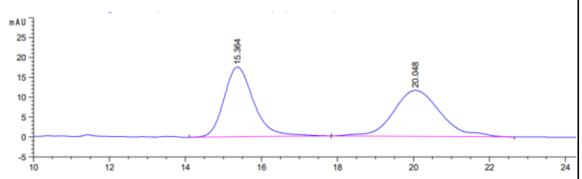

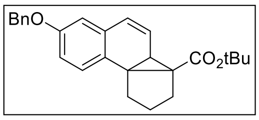

峰 #	保留时间 [min]	类型	峰宽 [min]	峰面积 [mAU*s]	峰高 [mAU]	峰面积 %
1	15.364	BB	0.8307	951.25275	17.54145	48.9338
2	20.048	BB	1.3170	992.70544	11.56989	51.0662

Chiral HPLC spectrum of **2i**

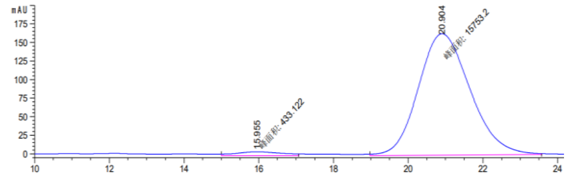

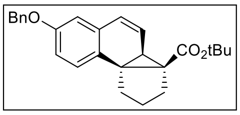

峰 #	保留时间 [min]	类型	峰宽 [min]	峰面积 [mAU*s]	峰高 [mAU]	峰面积 %
1	15.955	MM	1.4251	433.12244	5.06554	2.6758
2	20.904	MM	1.6057	1.57532e4	163.51404	97.3242

Chiral HPLC spectrum of racemic **2j**

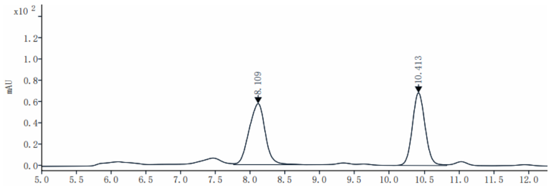

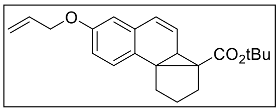

VWD1B, Wavelength=254 nm

Ret. Time	Area	Height	Area%
8.109	924.88	57.37	51.51
10.413	870.56	68.27	48.49

Chiral HPLC spectrum of **2j**

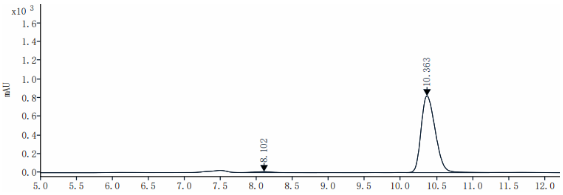

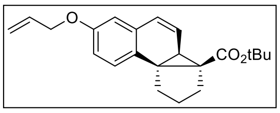

VWD1B, Wavelength=254 nm

Ret. Time	Area	Height	Area%
8.102	167.34	9.57	1.47
10.363	11216.56	821.17	98.53

Chiral HPLC spectrum of racemic **2k**

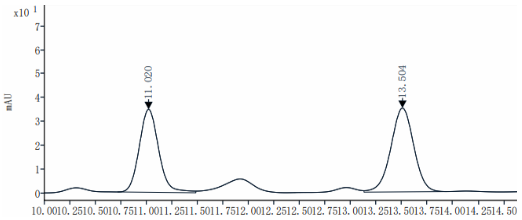

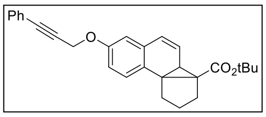

VWD1B, Wavelength=254 nm

Ret. Time	Area	Height	Area%
11.020	457.02	34.56	45.63
13.504	544.62	34.95	54.37

Chiral HPLC spectrum of **2k**

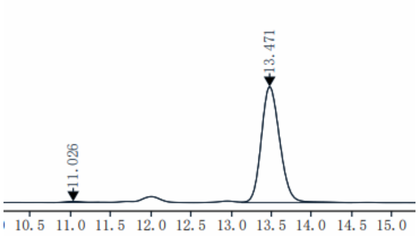

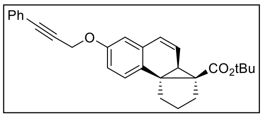

VWD1B, Wavelength=254 nm

Ret. Time	Area	Height	Area%
11.998	707.68	44.49	4.85
13.471	13874.55	866.35	95.15

Chiral HPLC spectrum of racemic **2l**

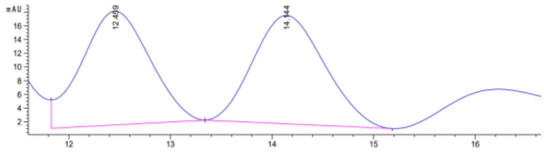

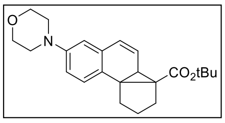

峰 #	保留时间 [min]	类型	峰宽 [min]	峰面积 [mAU*s]	峰高 [mAU]	峰面积 %
1	12.459	VB	0.7206	773.65588	16.57448	50.1974
2	14.144	BB	0.7672	767.57190	15.78792	49.8026

Chiral HPLC spectrum of **2l**

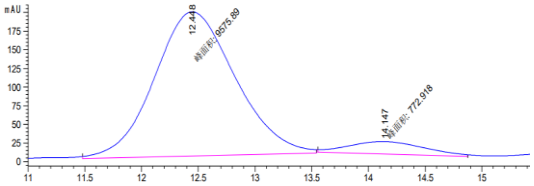

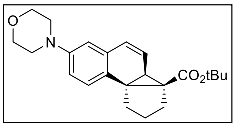

峰 #	保留时间 [min]	类型	峰宽 [min]	峰面积 [mAU*s]	峰高 [mAU]	峰面积 %
1	12.448	MM	0.7966	8978.66113	187.86050	92.6882
2	14.147	MM	0.7456	708.28729	15.83298	7.3118

Chiral HPLC spectrum of racemic **2m**

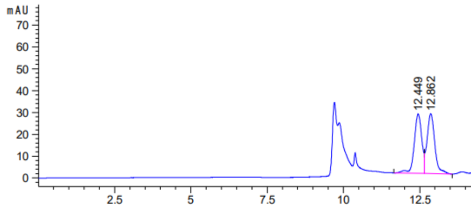

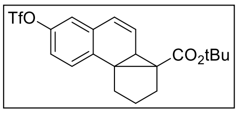

Peak #	RetTime [min]	Type	Width [min]	Area [mAU*s]	Height [mAU]	Area %
1	7.777	VV R	0.2124	280.22125	20.37611	49.5154
2	8.099	VB	0.2038	285.70569	21.12006	50.4846

Chiral HPLC spectrum of **2m**

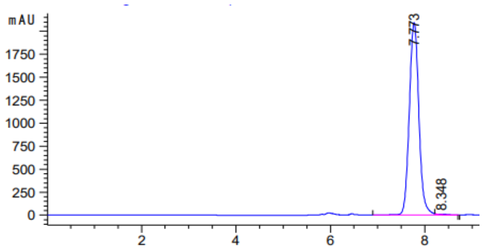

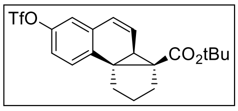

Peak #	RetTime [min]	Type	Width [min]	Area [mAU*s]	Height [mAU]	Area %
1	7.773	BV R	0.2186	2.95024e4	2091.47266	99.6921
2	8.348	VB E	0.2211	91.11530	6.28529	0.3079

Chiral HPLC spectrum of racemic **2n**

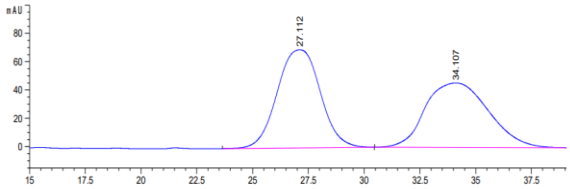

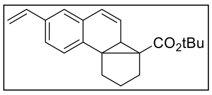

峰 #	保留时间 [min]	类型	峰宽 [min]	峰面积 [mAU*s]	峰高 [mAU]	峰面积 %
1	27.112	BB	2.0360	9291.29492	69.30683	50.9620
2	34.107	BB	3.0296	8940.52539	45.51733	49.0380

Chiral HPLC spectrum of **2n**

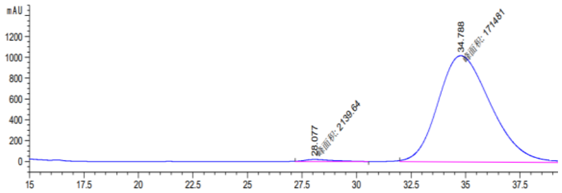

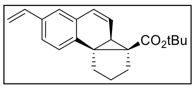

峰 #	保留时间 [min]	类型	峰宽 [min]	峰面积 [mAU*s]	峰高 [mAU]	峰面积 %
1	28.077	MM	1.6513	2139.63647	21.59529	1.2324
2	34.788	MM	2.8044	1.71481e5	1019.10260	98.7676

Chiral HPLC spectrum of racemic **2o**

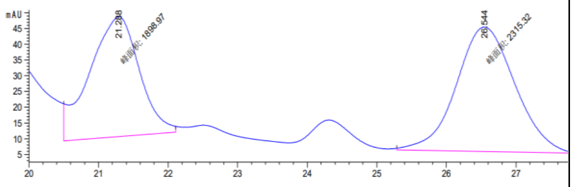

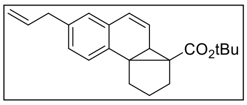

峰 #	保留时间 [min]	类型	峰宽 [min]	峰面积 [mAU*s]	峰高 [mAU]	峰面积 %
1	21.288	MM	0.8298	1898.96997	38.14236	45.0602
2	26.544	MM	0.9755	2315.32373	39.55942	54.9398

Chiral HPLC spectrum of **2o**

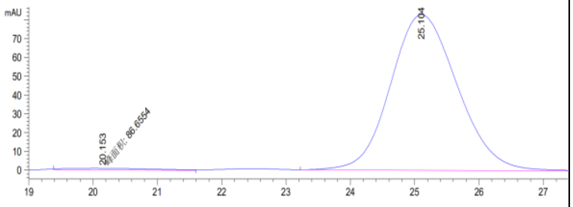

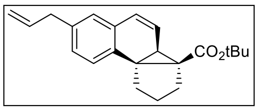

峰 #	保留时间 [min]	类型	峰宽 [min]	峰面积 [mAU*s]	峰高 [mAU]	峰面积 %
1	20.153	MM	1.6310	86.65545	8.85485e-1	1.4306
2	25.104	BBA	1.1258	5970.52783	82.74912	98.5694

Chiral HPLC spectrum of racemic **2p**

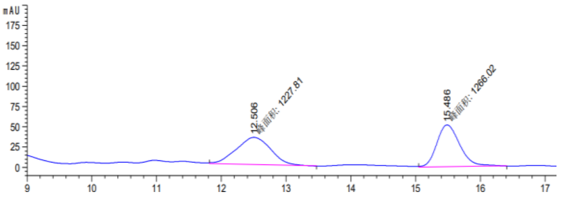

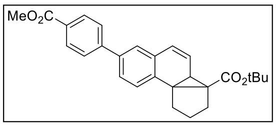

峰 #	保留时间 [min]	类型	峰宽 [min]	峰面积 [mAU*s]	峰高 [mAU]	峰面积 %
1	12.506	MM	0.6118	1227.80823	33.44891	49.2339
2	15.486	MM	0.4104	1266.01929	51.41250	50.7661

Chiral HPLC spectrum of **2p**

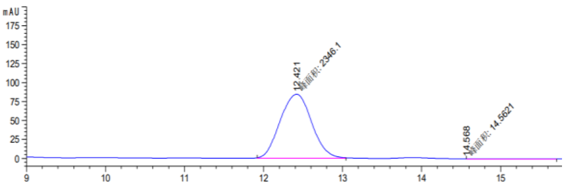

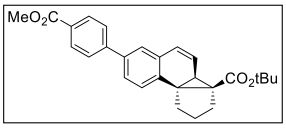

峰 #	保留时间 [min]	类型	峰宽 [min]	峰面积 [mAU*s]	峰高 [mAU]	峰面积 %
1	12.421	MM	0.4627	2346.10205	84.50029	99.3831
2	14.568	MM	0.5288	14.56210	3.22554e-1	0.6169

Chiral HPLC spectrum of racemic **2q**

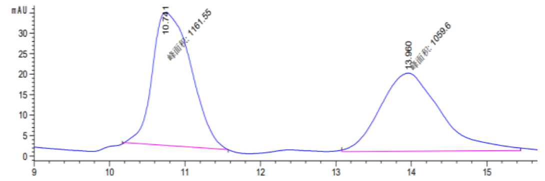

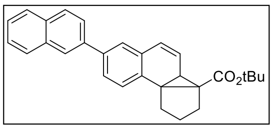

峰 #	保留时间 [min]	类型	峰宽 [min]	峰面积 [mAU*s]	峰高 [mAU]	峰面积 %
1	10.741	MM	0.5966	1161.55347	32.45079	52.2951
2	13.960	MM	0.9260	1059.59753	19.07189	47.7049

Chiral HPLC spectrum of **2q**

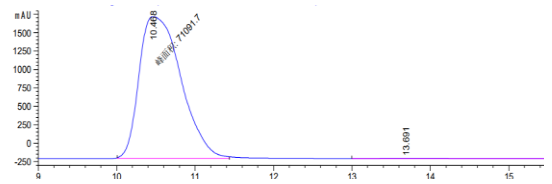

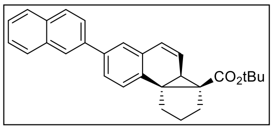

峰 #	保留时间 [min]	类型	峰宽 [min]	峰面积 [mAU*s]	峰高 [mAU]	峰面积 %
1	10.468	MM	0.6160	7.10917e4	1923.49426	99.5351
2	13.691	BBA	0.8872	332.03357	5.32697	0.4649

Chiral HPLC spectrum of racemic **2r**

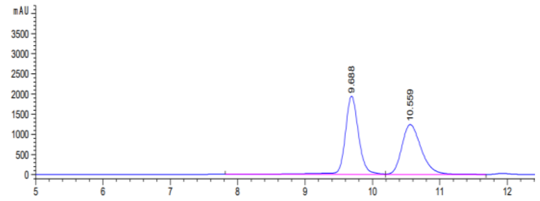

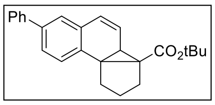

峰 #	保留时间 [min]	类型	峰宽 [min]	峰面积 [mAU*s]	峰高 [mAU]	峰面积 %
1	9.688	MM	0.2207	2.56247e4	1934.69299	50.3666
2	10.559	MM	0.3373	2.52517e4	1247.72913	49.6334

Chiral HPLC spectrum of **2r**

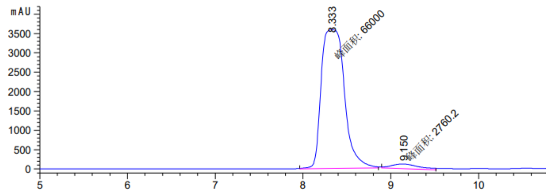

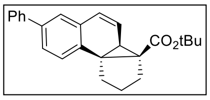

峰 #	保留时间 [min]	类型	峰宽 [min]	峰面积 [mAU*s]	峰高 [mAU]	峰面积 %
1	8.333	MM	0.3061	6.60000e4	3593.32935	95.9858
2	9.150	MM	0.3764	2760.20239	122.23000	4.0142

Chiral HPLC spectrum of racemic **2s**

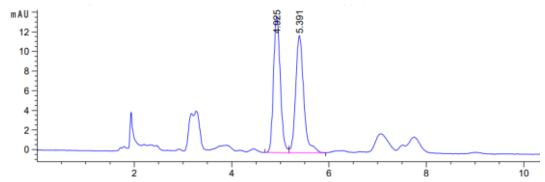

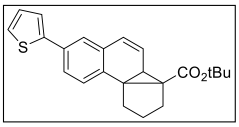

Peak #	RetTime [min]	Type	Width [min]	Area [mAU*s]	Height [mAU]	Area %
1	4.925	BV	0.1511	134.35776	13.86103	49.3840
2	5.391	VB	0.1789	137.70988	11.91117	50.6160

Chiral HPLC spectrum of **2s**

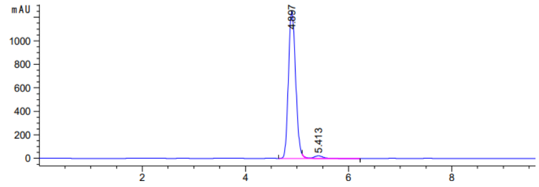

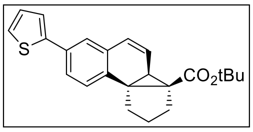

Peak #	RetTime [min]	Type	Width [min]	Area [mAU*s]	Height [mAU]	Area %
1	4.897	BV R	0.1537	9580.42480	983.17181	97.7921
2	5.413	VB E	0.1879	216.30586	17.30127	2.2079

Chiral HPLC spectrum of racemic **2t**

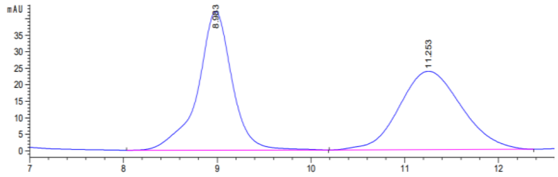

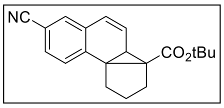

峰 #	保留时间 [min]	类型	峰宽 [min]	峰面积 [mAU*s]	峰高 [mAU]	峰面积 %
1	8.983	BB	0.3736	1064.50732	42.00721	49.3360
2	11.253	BB	0.7150	1093.16113	23.74871	50.6640

Chiral HPLC spectrum of **2t**

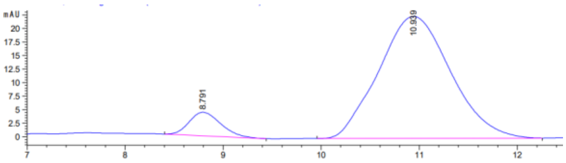

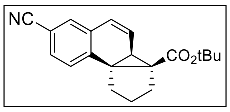

峰 #	保留时间 [min]	类型	峰宽 [min]	峰面积 [mAU*s]	峰高 [mAU]	峰面积 %
1	8.791	BB	0.3469	97.03662	4.33994	7.7956
2	10.939	BB	0.7764	1147.73108	22.52572	92.2044

Chiral HPLC spectrum of racemic **2u**

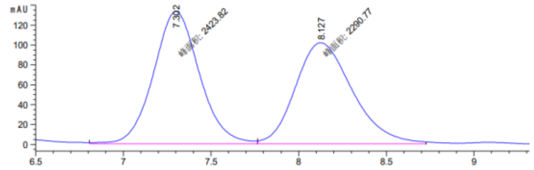

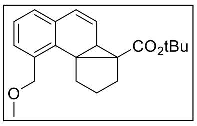

峰 #	保留时间 [min]	类型	峰宽 [min]	峰面积 [mAU*s]	峰高 [mAU]	峰面积 %
1	8.542	MM	0.2773	3794.42065	228.02844	50.9634
2	9.734	MM	0.3431	3650.95898	177.33154	49.0366

Chiral HPLC spectrum of racemic **2u**

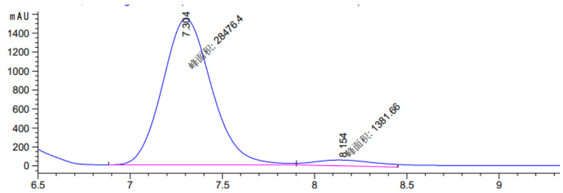

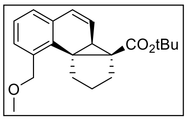

峰 #	保留时间 [min]	类型	峰宽 [min]	峰面积 [mAU*s]	峰高 [mAU]	峰面积 %
1	7.304	MM	0.3079	2.84764e4	1541.52722	95.3726
2	8.154	MM	0.3838	1381.65930	59.99681	4.6274

Chiral HPLC spectrum of racemic **2v**

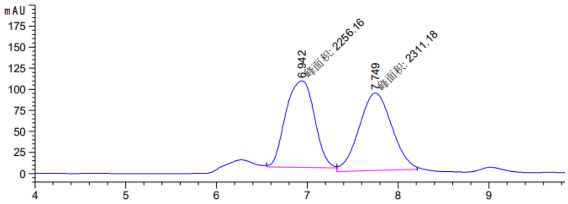

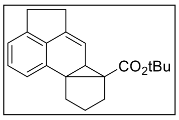

峰 #	保留时间 [min]	类型	峰宽 [min]	峰面积 [mAU*s]	峰高 [mAU]	峰面积 %
1	6.942	MM	0.3656	2256.16333	102.86256	49.3977
2	7.749	MM	0.4194	2311.17725	91.84441	50.6023

Chiral HPLC spectrum of **2v**

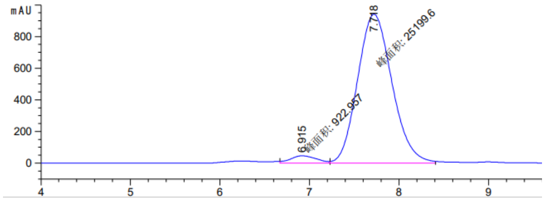

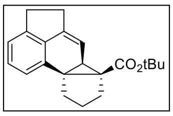

峰 #	保留时间 [min]	类型	峰宽 [min]	峰面积 [mAU*s]	峰高 [mAU]	峰面积 %
1	6.915	MM	0.3361	922.95709	45.76704	3.5332
2	7.718	MM	0.4461	2.51996e4	941.55273	96.4668

Chiral HPLC spectrum of racemic **5** derived from **4**

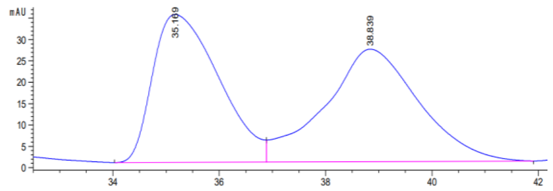

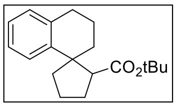

derived from

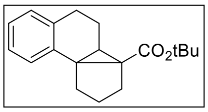

峰 #	保留时间 [min]	类型	峰宽 [min]	峰面积 [mAU*s]	峰高 [mAU]	峰面积 %
1	35.169	BV	1.3923	2993.69824	34.64013	48.0489
2	38.839	VB	1.7679	3236.82935	26.33630	51.9511

Chiral HPLC spectrum of **5** derived from **4**

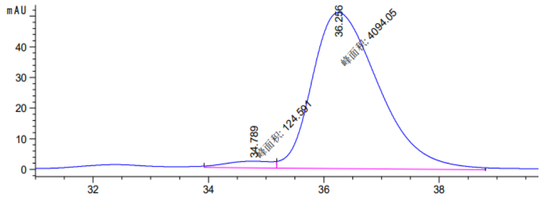

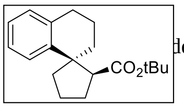

derived from

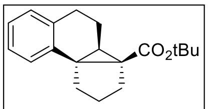

4

峰 #	保留时间 [min]	类型	峰宽 [min]	峰面积 [mAU*s]	峰高 [mAU]	峰面积 %
1	34.789	MF	0.9488	124.59115	2.18859	2.9534
2	36.256	FM	1.3398	4094.04688	50.92685	97.0466

Chiral HPLC spectrum of racemic **5**

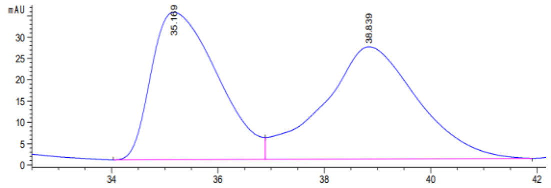

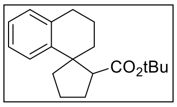

峰 #	保留时间 [min]	类型	峰宽 [min]	峰面积 [mAU*s]	峰高 [mAU]	峰面积 %
1	35.169	BV	1.3923	2993.69824	34.64013	48.0489
2	38.839	VB	1.7679	3236.82935	26.33630	51.9511

Chiral HPLC spectrum of **5**

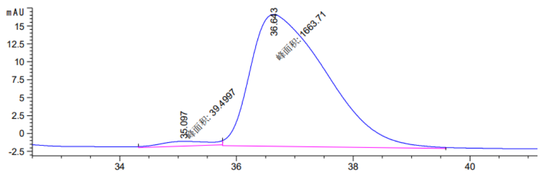

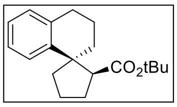

峰 #	保留时间 [min]	类型	峰宽 [min]	峰面积 [mAU*s]	峰高 [mAU]	峰面积 %
1	35.097	MM	1.0058	39.49966	6.54558e-1	2.3191
2	36.643	MM	1.5081	1663.70996	18.38666	97.6809

Chiral HPLC spectrum of racemic **6**

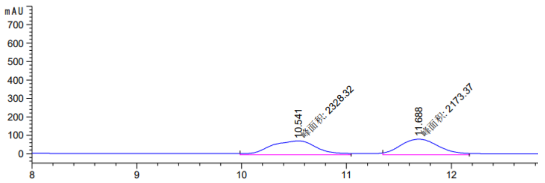

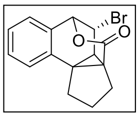

峰 #	保留时间 [min]	类型	峰宽 [min]	峰面积 [mAU*s]	峰高 [mAU]	峰面积 %
1	10.541	MM	0.5149	2328.31958	75.37010	51.7210
2	11.688	MM	0.4287	2173.37036	84.49004	48.2790

Chiral HPLC spectrum of **6**

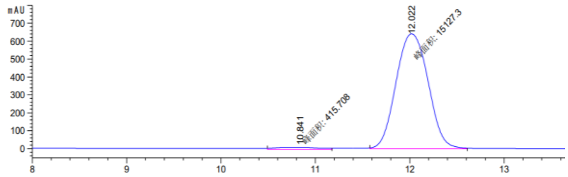

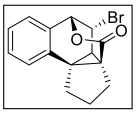

峰 #	保留时间 [min]	类型	峰宽 [min]	峰面积 [mAU*s]	峰高 [mAU]	峰面积 %
1	10.841	MM	0.5399	415.70773	12.83328	2.6746
2	12.022	MM	0.3935	1.51273e4	640.70142	97.3254

Chiral HPLC spectrum of racemic **7**

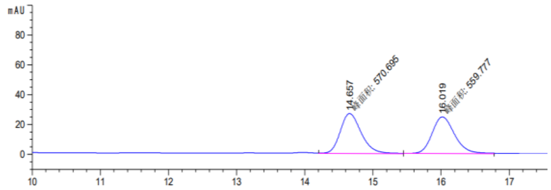

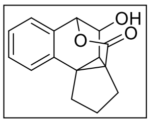

峰 #	保留时间 [min]	类型	峰宽 [min]	峰面积 [mAU*s]	峰高 [mAU]	峰面积 %
1	14.657	MM	0.3554	570.69476	26.76101	50.4829
2	16.019	MM	0.3806	559.77728	24.51354	49.5171

Chiral HPLC spectrum of **7**

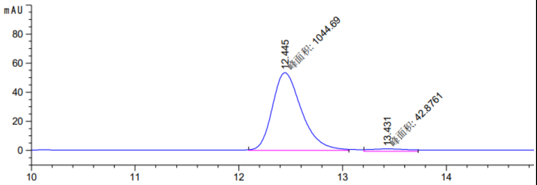

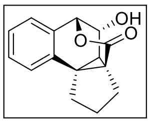

峰 #	保留时间 [min]	类型	峰宽 [min]	峰面积 [mAU*s]	峰高 [mAU]	峰面积 %
1	14.000	MM	0.3917	1008.15668	42.89618	98.1380
2	15.419	MM	0.4564	19.12853	6.98463e-1	1.8620

Chiral HPLC spectrum of racemic **8**

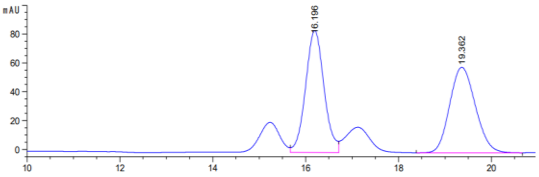

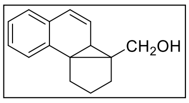

峰 #	保留时间 [min]	类型	峰宽 [min]	峰面积 [mAU*s]	峰高 [mAU]	峰面积 %
1	16.196	VV	0.4240	2334.55640	84.58681	50.5660
2	19.362	BB	0.6028	2282.29395	59.33413	49.4340

Chiral HPLC spectrum of **8**

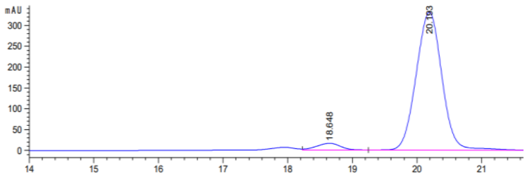

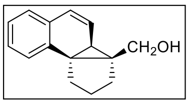

峰 #	保留时间 [min]	类型	峰宽 [min]	峰面积 [mAU*s]	峰高 [mAU]	峰面积 %
1	18.648	VB	0.4001	413.30954	16.17311	4.3257
2	20.193	BB	0.4323	9141.40430	330.85654	95.6743
